# THE CONCISE GUIDE TO PHARMACOLOGY 2019/20: Transporters

**DOI:** 10.1111/bph.14753

**Published:** 2019-11-11

**Authors:** Stephen PH Alexander, Eamonn Kelly, Alistair Mathie, John A Peters, Emma L Veale, Jane F Armstrong, Elena Faccenda, Simon D Harding, Adam J Pawson, Joanna L Sharman, Christopher Southan, Jamie A Davies, CGTP Collaborators, Catriona MH Anderson, Stefan Bröer, Paul Dawson, Bruno Hagenbuch, James R Hammond, Jules Hancox, Ken‐ichi Inui, Yoshikatsu Kanai, Stephan Kemp, David T Thwaites, Tiziano Verri

**Affiliations:** ^1^ School of Life Sciences University of Nottingham Medical School Nottingham NG7 2UH UK; ^2^ School of Physiology, Pharmacology and Neuroscience University of Bristol Bristol BS8 1TD UK; ^3^ Medway School of Pharmacy The Universities of Greenwich and Kent at Medway Anson Building, Central Avenue, Chatham Maritime Chatham Kent ME4 4TB UK; ^4^ Neuroscience Division, Medical Education Institute, Ninewells Hospital and Medical School University of Dundee Dundee DD1 9SY UK; ^5^ Centre for Discovery Brain Sciences University of Edinburgh Edinburgh EH8 9XD UK; ^6^ Newcastle upon Tyne UK; ^7^ Canberra Australia; ^8^ Atlanta USA; ^9^ Kansas City USA; ^10^ Edmonton Canada; ^11^ Bristol UK; ^12^ Kyoto Japan; ^13^ Osaka Japan; ^14^ Amsterdam The Netherlands; ^15^ Lecce Italy

## Abstract

The Concise Guide to PHARMACOLOGY 2019/20 is the fourth in this series of biennial publications. The Concise Guide provides concise overviews of the key properties of nearly 1800 human drug targets with an emphasis on selective pharmacology (where available), plus links to the open access knowledgebase source of drug targets and their ligands (http://www.guidetopharmacology.org), which provides more detailed views of target and ligand properties. Although the Concise Guide represents approximately 400 pages, the material presented is substantially reduced compared to information and links presented on the website. It provides a permanent, citable, point‐in‐time record that will survive database updates. The full contents of this section can be found at http://onlinelibrary.wiley.com/doi/10.1111/bph.14753. Transporters are one of the six major pharmacological targets into which the Guide is divided, with the others being: G protein‐coupled receptors, ion channels, nuclear hormone receptors, catalytic receptors and enzymes. These are presented with nomenclature guidance and summary information on the best available pharmacological tools, alongside key references and suggestions for further reading. The landscape format of the Concise Guide is designed to facilitate comparison of related targets from material contemporary to mid‐2019, and supersedes data presented in the 2017/18, 2015/16 and 2013/14 Concise Guides and previous Guides to Receptors and Channels. It is produced in close conjunction with the International Union of Basic and Clinical Pharmacology Committee on Receptor Nomenclature and Drug Classification (NC‐IUPHAR), therefore, providing official IUPHAR classification and nomenclature for human drug targets, where appropriate.

1

### Conflict of interest

The authors state that there are no conflicts of interest to disclose.

### Overview

The majority of biological solutes are charged organic or inorganic molecules. Cellular membranes are hydrophobic and, therefore, effective barriers to separate them allowing the formation of gradients, which can be exploited, for example, in the generation of energy. Membrane transporters carry solutes across cell membranes, which would otherwise be impermeable to them. The energy required for active transport processes is obtained from ATP turnover or by exploiting ion gradients.

ATP‐driven transporters can be divided into three major classes: P‐type ATPases; F‐type or V‐type ATPases and ATP‐binding cassette transporters. The first of these, P‐type ATPases, are multimeric proteins, which transport (primarily) inorganic cations. The second, F‐type or V‐type ATPases, are proton‐coupled motors, which can function either as transporters or as motors. Last, are ATP‐binding cassette transporters, heavily involved in drug disposition as well as transporting endogenous solutes.

The second largest family of membrane proteins in the human genome, after the G protein‐coupled receptors, are the SLC solute carrier family. Within the solute carrier family, there are a great variety of solutes transported, from simple inorganic ions to amino acids and sugars to relatively complex organic molecules like haem. The solute carrier family includes 65 families of almost 400 members. Many of these overlap in terms of the solutes that they carry. For example, amino acids accumulation is mediated by members of the SLC1, SLC3/7, SLC6, SLC15, SLC16, SLC17, SLC32, SLC36, SLC38 and SLC43 families. Further members of the SLC superfamily regulate ion fluxes at the plasma membrane, or solute transport into and out of cellular organelles. Some SLC family members remain orphan transporters, in as much as a physiological function has yet to be dtermined. Within the SLC super‐family, there is an abundance in diversity of structure. Two families (SLC3 and SLC7) only generate functional transporters as heteromeric partners, where one partner is a single TM domain protein. Membrane topology predictions for other families suggest 3,4,6,7,8,9,10,11,12,13 or 14 TM domains. The SLC transporters include members which function as antiports, where solute movement in one direction is balanced by a solute moving in the reverse direction. Symports allow concentration gradients of one solute to allow co‐transport of a second solute across a membrane. A third, relatively small group are equilibrative transporters, which allow solutes to travel across membranes down their concentration gradients. A more complex family of transporters, the SLC27 fatty acid transporters also express enzymatic function. Many of the transporters also express electrogenic properties of ion channels.

### Family structure

S399 ATP‐binding cassette transporter family

S399 ABCA subfamily

S401 ABCB subfamily

S403 ABCC subfamily

S404 ABCD subfamily of peroxisomal ABC transporters

S405 ABCG subfamily

S406 F‐type and V‐type ATPases

S406 F‐type ATPase

S407 V‐type ATPase

S407 P‐type ATPases

S407 Na^+^/K^+^‐ATPases

S408 Ca^2+^‐ATPases

S408 H^+^/K^+^‐ATPases

S408 Cu^+^‐ATPases

S409 Phospholipid‐transporting ATPases

S409 SLC superfamily of solute carriers

S410 SLC1 family of amino acid transporters

S410 Glutamate transporter subfamily

S412 Alanine/serine/cysteine transporter subfamily

S413 SLC2 family of hexose and sugar alcohol transporters

S413 Class I transporters

S414 Class II transporters

S415 Proton‐coupled inositol transporter

S415 SLC3 and SLC7 families of heteromeric amino acid transporters (HATs)

S415 SLC3 family

S416 SLC7 family

S417 SLC4 family of bicarbonate transporters

S417 Anion exchangers

S418 Sodium‐dependent HCO_3_ ^‐^ transporters

S418 SLC5 family of sodium‐dependent glucose transporters

S419 Hexose transporter family

S420 Choline transporter

S421 Sodium iodide symporter, sodium‐dependent multivitamin transporter and sodium‐coupled monocarboxylate transporters

S422 Sodium *myo*‐inositol cotransporter transporters

S423 SLC6 neurotransmitter transporter family

S423 Monoamine transporter subfamily

S424 GABA transporter subfamily

S425 Glycine transporter subfamily

S427 Neutral amino acid transporter subfamily

S428 SLC8 family of sodium/calcium exchangers

S429 SLC9 family of sodium/hydrogen exchangers

S429 SLC10 family of sodium‐bile acid co‐transporters

S431 SLC11 family of proton‐coupled metal ion transporters

S431 SLC12 family of cation‐coupled chloride transporters

S433 SLC13 family of sodium‐dependent sulphate/carboxylate transporters

S434 SLC14 family of facilitative urea transporters

S435 SLC15 family of peptide transporters

S437 SLC16 family of monocarboxylate transporters

S438 SLC17 phosphate and organic anion transporter family

S438 Type I sodium‐phosphate co‐transporters

S439 Sialic acid transporter

S439 Vesicular glutamate transporters (VGLUTs)

S440 Vesicular nucleotide transporter

S440 SLC18 family of vesicular amine transporters

S442 SLC19 family of vitamin transporters

S443 SLC20 family of sodium‐dependent phosphate transporters

S443 SLC22 family of organic cation and anion transporters

S444 Organic cation transporters (OCT)

S445 Organic zwitterions/cation transporters (OCTN)

S446 Organic anion transporters (OATs)

S446 Urate transporter

– http://www.guidetopharmacology.org/GRAC/FamilyDisplayForward?familyId=200

S447 Atypical SLC22B subfamily

S448 SLC23 family of ascorbic acid transporters

S449 SLC24 family of sodium/potassium/calcium exchangers

S450 SLC25 family of mitochondrial transporters

S450 Mitochondrial di‐ and tri‐carboxylic acid transporter subfamily

S451 Mitochondrial amino acid transporter subfamily

S452 Mitochondrial phosphate transporters

S452 Mitochondrial nucleotide transporter subfamily

S453 Mitochondrial uncoupling proteins

S454 Miscellaneous SLC25 mitochondrial transporters

S454 SLC26 family of anion exchangers

S454 Selective sulphate transporters

S455 Chloride/bicarbonate exchangers

S455 Anion channels

S456 Other SLC26 anion exchangers

S457 SLC27 family of fatty acid transporters

S458 SLC28 and SLC29 families of nucleoside transporters

S458 SLC28 family

S459 SLC29 family

S461 SLC30 zinc transporter family

S461 SLC31 family of copper transporters

S462 SLC32 vesicular inhibitory amino acid transporter

S463 SLC33 acetylCoA transporter

S464 SLC34 family of sodium phosphate co‐transporters

S465 SLC35 family of nucleotide sugar transporters

S466 SLC36 family of proton‐coupled amino acid transporters

S468 SLC37 family of phosphosugar/phosphate exchangers

S468 SLC38 family of sodium‐dependent neutral amino acid transporters

S469 System A‐like transporters

S469 System N‐like transporters

S470 Orphan SLC38 transporters

S470 SLC39 family of metal ion transporters

S471 SLC40 iron transporter

S472 SLC41 family of divalent cation transporters

S473 SLC42 family of Rhesus glycoprotein ammonium transporters

S473 SLC43 family of large neutral amino acid transporters

S474 SLC44 choline transporter‐like family

S475 SLC45 family of putative sugar transporters

S475 SLC46 family of folate transporters

S477 SLC47 family of multidrug and toxin extrusion transporters

S477 SLC48 heme transporter

S478 SLC49 family of FLVCR‐related heme transporters

S479 SLC50 sugar transporter

S479 SLC51 family of steroid‐derived molecule transporters

S480 SLC52 family of riboflavin transporters

S481 SLC53 Phosphate carriers

S481 SLC54 Mitochondrial pyruvate carriers

S482 SLC55 Mitochondrial cation/proton exchangers

S482 SLC56 Sideroflexins

S483 SLC57 NiPA‐like magnesium transporter family

S483 SLC58 MagT‐like magnesium transporter family

S484 SLC59 Sodium‐dependent lysophosphatidylcholine symporter family

S484 SLC60 Glucose transporters

S485 SLC61 Molybdate transporter family

S485 SLC62 Pyrophosphate transporters

S486 SLC63 Sphingosine‐phosphate transporters

S486 SLC64 Golgi Ca^2+^/H^+^ exchangers

S487 SLC65 NPC‐type cholesterol transporters

S488 SLCO family of organic anion transporting polypeptides

## http://www.guidetopharmacology.org/GRAC/FamilyDisplayForward?familyId=136

### Overview

ATP‐binding cassette transporters are ubiquitous membrane proteins characterized by active ATP‐dependent movement of a range of substrates, including ions, lipids, peptides, steroids. Individual subunits are typically made up of two groups of 6TM‐spanning domains, with two nucleotide‐binding domains (NBD). The majority of eukaryotic ABC transporters are ‘full’ transporters incorporating both TM and NBD entities. Some ABCs, notably the ABCD and ABCG families are half‐transporters with only a single membrane spanning domain and one NBD, and are only functional as homo‐ or heterodimers. Eukaryotic ABC transporters convey substrates from the cytoplasm, either out of the cell or into intracellular organelles. Their role in the efflux of exogenous compounds, notably chemotherapeutic agents, has led to considerable interest.

## http://www.guidetopharmacology.org/GRAC/FamilyDisplayForward?familyId=151

### Overview

To date, 12 members of the human ABCA subfamily are identified. They share a high degree of sequence conservation and have been mostly related with lipid trafficking in a wide range of body locations. Mutations in some of these genes have been described to cause severe hereditary diseases related with lipid transport, such as fatal surfactant deficiency or harlequin ichthyosis. In addition, most of them are hypothesized to participate in the subcellular sequestration of drugs, thereby being responsible for the resistance of several carcinoma cell lines against drug treatment [http://www.ncbi.nlm.nih.gov/pubmed/16586097?dopt=AbstractPlus].

**Table:** 

Nomenclature	http://www.guidetopharmacology.org/GRAC/ObjectDisplayForward?objectId=756	http://www.guidetopharmacology.org/GRAC/ObjectDisplayForward?objectId=758	http://www.guidetopharmacology.org/GRAC/ObjectDisplayForward?objectId=759
Common abbreviation	ABC1, CERP	ABC3, ABCC	ABCR
HGNC, UniProt	https://www.genenames.org/data/gene‐symbol‐report/#!/hgnc_id/HGNC:29, http://www.uniprot.org/uniprot/O95477	https://www.genenames.org/data/gene‐symbol‐report/#!/hgnc_id/HGNC:33, http://www.uniprot.org/uniprot/Q99758	https://www.genenames.org/data/gene‐symbol‐report/#!/hgnc_id/HGNC:34, http://www.uniprot.org/uniprot/P78363
Selective ligands	http://www.guidetopharmacology.org/GRAC/LigandDisplayForward?ligandId=9183 (Binding) [http://www.ncbi.nlm.nih.gov/pubmed/18805791?dopt=AbstractPlus]	–	–
Selective inhibitors	http://www.guidetopharmacology.org/GRAC/LigandDisplayForward?ligandId=7277 [http://www.ncbi.nlm.nih.gov/pubmed/15514211?dopt=AbstractPlus, http://www.ncbi.nlm.nih.gov/pubmed/15140889?dopt=AbstractPlus]	–	–
Comments	–	Loss‐of‐function mutations are associated with pulmonary surfactant deficiency	Retinal‐specific transporter of N‐retinylPE; loss‐of‐function mutations are associated with childhood‐onset Stargardt disease, a juvenile onset macular degenerative disease. The earlier onset disease is often associated with the more severe and deleterious *ABCA4* variants [http://www.ncbi.nlm.nih.gov/pubmed/25312043?dopt=AbstractPlus]. ABCA4 facilitates the clearance of all‐*trans*‐retinal from photoreceptor disc membranes following photoexcitation. ABCA4 can also transport N‐11‐*cis*‐retinylidene‐phosphatidylethanolamine, the Schiff‐base adduct of 11‐*cis*‐retinal; loss of function mutation cause a buildup of lipofuscin, atrophy of the central retina, and severe progressive loss in vision [http://www.ncbi.nlm.nih.gov/pubmed/24707049?dopt=AbstractPlus].

**Table:** 

Nomenclature	http://www.guidetopharmacology.org/GRAC/ObjectDisplayForward?objectId=760	http://www.guidetopharmacology.org/GRAC/ObjectDisplayForward?objectId=761	http://www.guidetopharmacology.org/GRAC/ObjectDisplayForward?objectId=762	http://www.guidetopharmacology.org/GRAC/ObjectDisplayForward?objectId=766
HGNC, UniProt	https://www.genenames.org/data/gene‐symbol‐report/#!/hgnc_id/HGNC:35, http://www.uniprot.org/uniprot/Q8WWZ7	https://www.genenames.org/data/gene‐symbol‐report/#!/hgnc_id/HGNC:36, http://www.uniprot.org/uniprot/Q8N139	https://www.genenames.org/data/gene‐symbol‐report/#!/hgnc_id/HGNC:37, http://www.uniprot.org/uniprot/Q8IZY2	https://www.genenames.org/data/gene‐symbol‐report/#!/hgnc_id/HGNC:14637, http://www.uniprot.org/uniprot/Q86UK0
Comments	ABCA5 is a lysosomal protein whose loss of function compromises integrity of lysosomes and leads to intra‐endolysosomal accumulation of cholesterol. It has recently been associated with Congenital Generalized Hypertrichosis Terminalis (CGHT), a hair overgrowth syndrome, in a patient with a mutation in *ABCA5* that significantly decreased its expression [http://www.ncbi.nlm.nih.gov/pubmed/24831815?dopt=AbstractPlus].	A recent genome wide association study identified an *ABCA6* variant associated with cholesterol levels [http://www.ncbi.nlm.nih.gov/pubmed/25751400?dopt=AbstractPlus].	Genome wide association studies identify *ABCA7* variants as associated with Alzheimer's Disease [http://www.ncbi.nlm.nih.gov/pubmed/21460840?dopt=AbstractPlus].	Reported to play a role in skin ceramide formation [http://www.ncbi.nlm.nih.gov/pubmed/18957418?dopt=AbstractPlus]. A recent study shows that *ABCA12* expression also impacts cholesterol efflux from macrophages. ABCA12 is postulated to associate with ABCA1 and LXR beta, and stabilize expression of *ABCA1*. ABCA12 deficiency causes decreased expression of *Abca1*, *Abcg1* and *Nr1h2* [http://www.ncbi.nlm.nih.gov/pubmed/23931754?dopt=AbstractPlus].

### Comments

A number of structural analogues are not found in man: Abca14 (http://www.ensembl.org/Mus_musculus/Gene/Summary?g=ENSMUSG00000062017;r=7:127347475‐127468866); Abca15 (http://www.ensembl.org/Mus_musculus/Gene/Summary?g=ENSMUSG00000054746;r=7:127472198‐127551201); Abca16 (http://www.ensembl.org/Mus_musculus/Gene/Summary?g=ENSMUSG00000051900;r=7:127553161‐127688327) and Abca17 (http://www.ensembl.org/Mus_musculus/Gene/Summary?g=ENSMUSG00000035435;r=17:24401204‐24487974).

## http://www.guidetopharmacology.org/GRAC/FamilyDisplayForward?familyId=152

### Overview

The ABCB subfamily is composed of four full transporters and two half transporters. This is the only human subfamily to have both half and full types of transporters. ABCB1 was discovered as a protein overexpressed in certain drug resistant tumor cells. It is expressed primarily in the blood brain barrier and liver and is thought to be involved in protecting cells from toxins. Cells that overexpress this protein exhibit multi‐drug resistance [http://www.ncbi.nlm.nih.gov/pubmed/11441126?dopt=AbstractPlus].

**Table:** 

Nomenclature	http://www.guidetopharmacology.org/GRAC/ObjectDisplayForward?objectId=768	http://www.guidetopharmacology.org/GRAC/ObjectDisplayForward?objectId=769	http://www.guidetopharmacology.org/GRAC/ObjectDisplayForward?objectId=770
Common abbreviation	MDR1, PGP1	TAP1	TAP2
HGNC, UniProt	https://www.genenames.org/data/gene‐symbol‐report/#!/hgnc_id/HGNC:40, http://www.uniprot.org/uniprot/P08183	https://www.genenames.org/data/gene‐symbol‐report/#!/hgnc_id/HGNC:43, http://www.uniprot.org/uniprot/Q03518	https://www.genenames.org/data/gene‐symbol‐report/#!/hgnc_id/HGNC:44, http://www.uniprot.org/uniprot/Q03519
Comments	Responsible for the cellular export of many therapeutic drugs. The mouse and rat have two Abcb1 genes (gene names; *Abcb1a* and *Abcb1b*) while the human has only the one gene, *ABCB1*.	Endoplasmic reticulum peptide transporter is a hetero‐dimer composed of the two half‐transporters, TAP1 (ABCB2) and TAP2 (ABCB3). The transporter shuttles peptides into the endoplasmic reticulum where they are loaded onto major histocompatibility complex class I (MHCI) molecules via the macromoldecular peptide‐loading complex and are eventually presented at the cell surface, attributing to TAP an important role in the adaptive immune response [http://www.ncbi.nlm.nih.gov/pubmed/24923865?dopt=AbstractPlus].	

**Table:** 

Nomenclature	http://www.guidetopharmacology.org/GRAC/ObjectDisplayForward?objectId=771	http://www.guidetopharmacology.org/GRAC/ObjectDisplayForward?objectId=772	http://www.guidetopharmacology.org/GRAC/ObjectDisplayForward?objectId=773
Common abbreviation	PGY3	–	MTABC3
HGNC, UniProt	https://www.genenames.org/data/gene‐symbol‐report/#!/hgnc_id/HGNC:45, http://www.uniprot.org/uniprot/P21439	https://www.genenames.org/data/gene‐symbol‐report/#!/hgnc_id/HGNC:46, http://www.uniprot.org/uniprot/Q2M3G0	https://www.genenames.org/data/gene‐symbol‐report/#!/hgnc_id/HGNC:47, http://www.uniprot.org/uniprot/Q9NP58
Comments	Transports phosphatidylcholinefrom intracellular to extracellular face of the hepatocyte canalicular membrane [http://www.ncbi.nlm.nih.gov/pubmed/16622704?dopt=AbstractPlus]. Heterozygous *ABCB4* variants contribute to mild cholestatic phenotypes, while homozygous deficiency leads to Progressive Intrahepatic Familial Cholestasis (PFIC) Type 3, and increased risk of cholesterol gallstones [http://www.ncbi.nlm.nih.gov/pubmed/23583734?dopt=AbstractPlus].	A drug efflux transporter that has been shown to identify cancer stem‐like cells in diverse human malignancies, and is also identified as a limbal stem cell that is required for corneal development and repair [http://www.ncbi.nlm.nih.gov/pubmed/25030174?dopt=AbstractPlus, http://www.ncbi.nlm.nih.gov/pubmed/24934811?dopt=AbstractPlus].	Putative mitochondrial porphyrin transporter [http://www.ncbi.nlm.nih.gov/pubmed/17006453?dopt=AbstractPlus]; other subcellular localizations are possible, such as the plasma membrane, as a specific determinant of the Langereis blood group system [http://www.ncbi.nlm.nih.gov/pubmed/22246506?dopt=AbstractPlus]. Loss of *Abcb6* expression in mice leads to decreased expression and activity of CYP450 [http://www.ncbi.nlm.nih.gov/pubmed/25623066?dopt=AbstractPlus].

**Table:** 

Nomenclature	http://www.guidetopharmacology.org/GRAC/ObjectDisplayForward?objectId=774	http://www.guidetopharmacology.org/GRAC/ObjectDisplayForward?objectId=775	http://www.guidetopharmacology.org/GRAC/ObjectDisplayForward?objectId=776
Common abbreviation	ABC7	MABC1	TAPL
HGNC, UniProt	https://www.genenames.org/data/gene‐symbol‐report/#!/hgnc_id/HGNC:48, http://www.uniprot.org/uniprot/O75027	https://www.genenames.org/data/gene‐symbol‐report/#!/hgnc_id/HGNC:49, http://www.uniprot.org/uniprot/Q9NUT2	https://www.genenames.org/data/gene‐symbol‐report/#!/hgnc_id/HGNC:50, http://www.uniprot.org/uniprot/Q9NP78
Comments	Mitochondrial; reportedly essential for haematopoiesis [http://www.ncbi.nlm.nih.gov/pubmed/17192398?dopt=AbstractPlus]. Deletion studies in mice demonstrate that *Abcb7* is essential in mammals and substantiate a role for mitochondria in cytosolic Fe‐S cluster assembly [http://www.ncbi.nlm.nih.gov/pubmed/16467350?dopt=AbstractPlus].	Mitochondrial; suggested to play a role in chemoresistance of melanoma [http://www.ncbi.nlm.nih.gov/pubmed/19147539?dopt=AbstractPlus]. Cardiac specific deletion of *Abcb8* leads to cardiomyopathy and accumulation of mitochondrial iron, and is thus thought to modulate mitochondrial iron export [http://www.ncbi.nlm.nih.gov/pubmed/22375032?dopt=AbstractPlus].	A homodimeric transport complex that translocates cytosolic peptides into the lumen of lysosome for degradation [http://www.ncbi.nlm.nih.gov/pubmed/22641697?dopt=AbstractPlus].

**Table:** 

Nomenclature	http://www.guidetopharmacology.org/GRAC/ObjectDisplayForward?objectId=777	http://www.guidetopharmacology.org/GRAC/ObjectDisplayForward?objectId=778
Common abbreviation	MTABC2	ABC16
HGNC, UniProt	https://www.genenames.org/data/gene‐symbol‐report/#!/hgnc_id/HGNC:41, http://www.uniprot.org/uniprot/Q9NRK6	https://www.genenames.org/data/gene‐symbol‐report/#!/hgnc_id/HGNC:42, http://www.uniprot.org/uniprot/O95342
Ligands	–	http://www.guidetopharmacology.org/GRAC/LigandDisplayForward?ligandId=4545 (Binding) (p*K*_i_ 5.2) [http://www.ncbi.nlm.nih.gov/pubmed/12404239?dopt=AbstractPlus]
Comments	Mitochondrial location; the first human ABC transporter to have a crystal structure reported [http://www.ncbi.nlm.nih.gov/pubmed/23716676?dopt=AbstractPlus]. ABCB10 is important in early steps of heme synthesis in the heart and is required for normal red blood cell development [http://www.ncbi.nlm.nih.gov/pubmed/23720443?dopt=AbstractPlus, http://www.ncbi.nlm.nih.gov/pubmed/22085049?dopt=AbstractPlus].	Loss‐of‐function mutations are associated with progressive familial intrahepatic cholestasis type 2 [http://www.ncbi.nlm.nih.gov/pubmed/19684528?dopt=AbstractPlus]. ATP‐dependent transport of bile acids into the confines of the canalicular space by ABCB11 (BSEP) generates an osmotic gradient and thereby, bile flow. Mutations in BSEP that decrease its function or expression cause Progressive Familial Cholestasis Type 2 (PFIC2), which in severe cases, can be fatal in the absence of a liver transplant. Drugs that inhibit BSEP function with IC50 values less than 25 *μ*M [http://www.ncbi.nlm.nih.gov/pubmed/20829430?dopt=AbstractPlus] or decrease its expression [http://www.ncbi.nlm.nih.gov/pubmed/24335466?dopt=AbstractPlus] can cause Drug‐Induced Liver Injury (DILI) in the form of cholestatic liver injury.

## http://www.guidetopharmacology.org/GRAC/FamilyDisplayForward?familyId=153

### Overview

Subfamily ABCC contains thirteen members and nine of these transporters are referred to as Multidrug Resistance Proteins (MRPs). MRP proteins are found throughout nature and mediate many important functions. They are known to be involved in ion transport, toxin secretion, and signal transduction [http://www.ncbi.nlm.nih.gov/pubmed/11441126?dopt=AbstractPlus].

**Table:** 

Nomenclature	http://www.guidetopharmacology.org/GRAC/ObjectDisplayForward?objectId=779	http://www.guidetopharmacology.org/GRAC/ObjectDisplayForward?objectId=780	http://www.guidetopharmacology.org/GRAC/ObjectDisplayForward?objectId=781
Common abbreviation	MRP1	MRP2, cMOAT	MRP3
HGNC, UniProt	https://www.genenames.org/data/gene‐symbol‐report/#!/hgnc_id/HGNC:51, http://www.uniprot.org/uniprot/P33527	https://www.genenames.org/data/gene‐symbol‐report/#!/hgnc_id/HGNC:53, http://www.uniprot.org/uniprot/Q92887	https://www.genenames.org/data/gene‐symbol‐report/#!/hgnc_id/HGNC:54, http://www.uniprot.org/uniprot/O15438
Inhibitors	http://www.guidetopharmacology.org/GRAC/LigandDisplayForward?ligandId=8762 (p*K*_i_ 7.2) [http://www.ncbi.nlm.nih.gov/pubmed/9647783?dopt=AbstractPlus]	http://www.guidetopharmacology.org/GRAC/LigandDisplayForward?ligandId=8776 (p*K*_i_ 5.4) [http://www.ncbi.nlm.nih.gov/pubmed/10570049?dopt=AbstractPlus]	–
Comments	Exhibits a broad substrate specificity [http://www.ncbi.nlm.nih.gov/pubmed/17187268?dopt=AbstractPlus], including http://www.guidetopharmacology.org/GRAC/LigandDisplayForward?ligandId=3354 (K_m_ 97 nM [http://www.ncbi.nlm.nih.gov/pubmed/7961706?dopt=AbstractPlus]) and estradiol‐17β‐glucuronide [http://www.ncbi.nlm.nih.gov/pubmed/9875554?dopt=AbstractPlus].	Loss‐of‐function mutations are associated with Dubin‐Johnson syndrome, in which plasma levels of conjugated bilirubin are elevated (http://omim.org/entry/237500).	Transports conjugates of glutathione, sulfate or glucuronide [http://www.ncbi.nlm.nih.gov/pubmed/16586096?dopt=AbstractPlus]

**Table:** 

Nomenclature	http://www.guidetopharmacology.org/GRAC/ObjectDisplayForward?objectId=782	http://www.guidetopharmacology.org/GRAC/ObjectDisplayForward?objectId=783	http://www.guidetopharmacology.org/GRAC/ObjectDisplayForward?objectId=784
Common abbreviation	MRP4	MRP5	MRP6
HGNC, UniProt	https://www.genenames.org/data/gene‐symbol‐report/#!/hgnc_id/HGNC:55, http://www.uniprot.org/uniprot/O15439	https://www.genenames.org/data/gene‐symbol‐report/#!/hgnc_id/HGNC:56, http://www.uniprot.org/uniprot/O15440	https://www.genenames.org/data/gene‐symbol‐report/#!/hgnc_id/HGNC:57, http://www.uniprot.org/uniprot/O95255
Inhibitors	http://www.guidetopharmacology.org/GRAC/LigandDisplayForward?ligandId=8802 (pIC_50_ 6.7) [http://www.ncbi.nlm.nih.gov/pubmed/12523936?dopt=AbstractPlus]	http://www.guidetopharmacology.org/GRAC/LigandDisplayForward?ligandId=8829 (p*K*_i_ 7.2) [http://www.ncbi.nlm.nih.gov/pubmed/22380603?dopt=AbstractPlus], http://www.guidetopharmacology.org/GRAC/LigandDisplayForward?ligandId=4743 (p*K*_i_ 5.9) [http://www.ncbi.nlm.nih.gov/pubmed/22380603?dopt=AbstractPlus]	–
Comments	Although reported to facilitate cellular cyclic nucleotide export, this role has been questioned [http://www.ncbi.nlm.nih.gov/pubmed/16586096?dopt=AbstractPlus]; reported to export prostaglandins in a manner sensitive to NSAIDS [http://www.ncbi.nlm.nih.gov/pubmed/12835412?dopt=AbstractPlus]	Although reported to facilitate cellular cyclic nucleotide export, this role has been questioned [http://www.ncbi.nlm.nih.gov/pubmed/16586096?dopt=AbstractPlus]	Loss‐of‐function mutations in ABCC6 are associated with pseudoxanthoma elasticum (http://omim.org/entry/264800).

**Table:** 

Nomenclature	http://www.guidetopharmacology.org/GRAC/ObjectDisplayForward?objectId=2594	http://www.guidetopharmacology.org/GRAC/ObjectDisplayForward?objectId=2746	http://www.guidetopharmacology.org/GRAC/ObjectDisplayForward?objectId=786
Systematic nomenclature	ABCC8	–	–
Common abbreviation	SUR1	SUR2	MRP8
HGNC, UniProt	https://www.genenames.org/data/gene‐symbol‐report/#!/hgnc_id/HGNC:59, http://www.uniprot.org/uniprot/Q09428	https://www.genenames.org/data/gene‐symbol‐report/#!/hgnc_id/HGNC:60, http://www.uniprot.org/uniprot/O60706	https://www.genenames.org/data/gene‐symbol‐report/#!/hgnc_id/HGNC:14639, http://www.uniprot.org/uniprot/Q96J66
Selective inhibitors	http://www.guidetopharmacology.org/GRAC/LigandDisplayForward?ligandId=6841 (pIC_50_ 7) [http://www.ncbi.nlm.nih.gov/pubmed/15380228?dopt=AbstractPlus]	–	–
Comments	The sulfonyurea drugs (acetohexamide, tolbutamide and glibenclamide) appear to bind sulfonylurea receptors and it has been shown experimentally that tritiated glibenclamide can be used to pull out a 140 kDa protein identified as SUR1 (now known as ABCC8) [http://www.ncbi.nlm.nih.gov/pubmed/22260657?dopt=AbstractPlus]. SUR2 (ABCC9) has also been identified [http://www.ncbi.nlm.nih.gov/pubmed/7502040?dopt=AbstractPlus]. However, this is not the full mechanism of action and the functional channel has been characterised as a hetero‐octamer formed by four SUR and four K_ir_6.2 subunits, with the K_ir_6.2 subunits forming the core ion pore and the SUR subunits providing the regulatory properties [http://www.ncbi.nlm.nih.gov/pubmed/10194514?dopt=AbstractPlus]. Co‐expression of K_ir_6.2 with SUR1, reconstitutes the ATP‐dependent K^+^ conductivity inhibited by the sulfonyureas [http://www.ncbi.nlm.nih.gov/pubmed/7502040?dopt=AbstractPlus].	Associated with familial atrial fibrillation, Cantu syndrome and familial isolated dilated cardiomyopathy.	Single nucleotide polymorphisms distinguish wet vs. dry earwax (http://omim.org/entry/117800); an association between earwax allele and breast cancer risk is reported in Japanese but not European populations.

### Comments

ABCC7 (also known as http://www.guidetopharmacology.org/GRAC/ObjectDisplayForward?objectId=707, a 12TM ABC transporter‐type protein, is a cAMP‐regulated epithelial cell membrane Cl^‐^ channel involved in normal fluid transport across various epithelia and can be viewed in the http://www.guidetopharmacology.org/GRAC/FamilyDisplayForward?familyId=120 section of the Guide. ABCC8 (http://www.ensembl.org/Homo_sapiens/Gene/Summary?g=ENSG00000006071;r=11:17414432‐17498449, also known as SUR1, sulfonylurea receptor 1) and ABCC9 (http://www.ensembl.org/Homo_sapiens/Gene/Summary?g=ENSG00000069431;r=12:21950335‐22094336, also known as SUR2, sulfonylurea receptor 2) are unusual in that they lack transport capacity but regulate the activity of particular K^+^ channels (http://www.guidetopharmacology.org/GRAC/FamilyDisplayForward?familyId=74), conferring nucleotide sensitivity to these channels to generate the canonical K_ATP_ channels. ABCC13 (http://www.ensembl.org/Homo_sapiens/Gene/Summary?g=ENSG00000243064;r=21:15646120‐15735075) is a possible pseudogene.

## http://www.guidetopharmacology.org/GRAC/FamilyDisplayForward?familyId=154

### Overview

Peroxisomes are indispensable organelles in higher eukaryotes. They are essential for the oxidation of a wide variety of metabolites, which include: saturated, monounsaturated and polyunsaturated fatty acids, branched‐chain fatty acids, bile acids and dicarboxylic acids [http://www.ncbi.nlm.nih.gov/pubmed/21488864?dopt=AbstractPlus]. However, the peroxisomal membrane forms an impermeable barrier to these metabolites. The mammalian peroxisomal membrane harbours three ATP‐binding cassette (ABC) half‐transporters, which act as homo‐ and/or heterodimers to transport these metabolites across the peroxisomal membrane.

**Table:** 

Nomenclature	http://www.guidetopharmacology.org/GRAC/ObjectDisplayForward?objectId=788	http://www.guidetopharmacology.org/GRAC/ObjectDisplayForward?objectId=789	http://www.guidetopharmacology.org/GRAC/ObjectDisplayForward?objectId=790
Common abbreviation	ALDP	ALDR	PMP70
HGNC, UniProt	https://www.genenames.org/data/gene‐symbol‐report/#!/hgnc_id/HGNC:61, http://www.uniprot.org/uniprot/P33897	https://www.genenames.org/data/gene‐symbol‐report/#!/hgnc_id/HGNC:66, http://www.uniprot.org/uniprot/Q9UBJ2	https://www.genenames.org/data/gene‐symbol‐report/#!/hgnc_id/HGNC:67, http://www.uniprot.org/uniprot/P28288
Comments	Transports coenzyme A esters of very long chain fatty acids [http://www.ncbi.nlm.nih.gov/pubmed/18757502?dopt=AbstractPlus, http://www.ncbi.nlm.nih.gov/pubmed/21145416?dopt=AbstractPlus]. Loss‐of‐function mutations in *ABCD1* (mutation registry held by the Adrenoleukodystrophy database; https://adrenoleukodystrophy.info/) result in adrenoleukodystrophy (http://omim.org/entry/300100) [http://www.ncbi.nlm.nih.gov/pubmed/27312864?dopt=AbstractPlus].	*In vitro* experiments indicate that ABCD2 has overlapping substrate specificity with ABCD1 towards saturated and monounsaturated very long‐chain fatty acids, albeit at much lower specificity. ABCD2 has affinity for the polyunsaturated fatty acids C22:6‐CoA and C24:6‐CoA. However, *in vivo* proof for its true function is still lacking. No disease has yet been linked to a deficiency of ABCD2.	Transports long‐chain dicarboxylic acids, branched‐chain fatty acids and C27 bile acids DHC‐CoA and THC‐CoA [http://www.ncbi.nlm.nih.gov/pubmed/25168382?dopt=AbstractPlus]. In mitochondrial fatty acid deficient cells and mice, ABCD3 accepts medium and long‐chain fatty acids

### Comments

ABCD4 (http://www.ensembl.org/Homo_sapiens/Gene/Summary?g=ENSG00000119688;r=14:74752126‐74769759, also known as PMP69, PXMP1‐L or P70R) is located at the lysosome and is involved in the transport of vitamin B12 (cobalamin) from lysosomes into the cytosol [http://www.ncbi.nlm.nih.gov/pubmed/22922874?dopt=AbstractPlus].

## http://www.guidetopharmacology.org/GRAC/FamilyDisplayForward?familyId=155

### Overview

This family of ‘half‐transporters’ act as homo‐ or heterodimers; particularly ABCG5 and ABCG8 are thought to be obligate heterodimers. The ABCG5/ABCG heterodimer sterol transporter structure has been determined [http://www.ncbi.nlm.nih.gov/pubmed/27144356?dopt=AbstractPlus], suggesting an extensive intracellular nucleotide binding domain linked to the transmembrane domains by a fold in the primary sequence. The functional ABCG2 transporter appears to be a homodimer with structural similarities to the ABCG5/ABCG8 heterodimer [http://www.ncbi.nlm.nih.gov/pubmed/28554189?dopt=AbstractPlus].

**Table:** 

Nomenclature	http://www.guidetopharmacology.org/GRAC/ObjectDisplayForward?objectId=791	http://www.guidetopharmacology.org/GRAC/ObjectDisplayForward?objectId=792	http://www.guidetopharmacology.org/GRAC/ObjectDisplayForward?objectId=793	http://www.guidetopharmacology.org/GRAC/ObjectDisplayForward?objectId=794	http://www.guidetopharmacology.org/GRAC/ObjectDisplayForward?objectId=795
Common abbreviation	ABC8	ABCP	–	–	–
HGNC, UniProt	https://www.genenames.org/data/gene‐symbol‐report/#!/hgnc_id/HGNC:73, http://www.uniprot.org/uniprot/P45844	https://www.genenames.org/data/gene‐symbol‐report/#!/hgnc_id/HGNC:74, http://www.uniprot.org/uniprot/Q9UNQ0	https://www.genenames.org/data/gene‐symbol‐report/#!/hgnc_id/HGNC:13884, http://www.uniprot.org/uniprot/Q9H172	https://www.genenames.org/data/gene‐symbol‐report/#!/hgnc_id/HGNC:13886, http://www.uniprot.org/uniprot/Q9H222	https://www.genenames.org/data/gene‐symbol‐report/#!/hgnc_id/HGNC:13887, http://www.uniprot.org/uniprot/Q9H221
Inhibitors	–	http://www.guidetopharmacology.org/GRAC/LigandDisplayForward?ligandId=1024 (p*K*_i_ 6.3) [http://www.ncbi.nlm.nih.gov/pubmed/11437380?dopt=AbstractPlus]	–	–	–
Comments	Transports sterols and choline phospholipids [http://www.ncbi.nlm.nih.gov/pubmed/21175590?dopt=AbstractPlus]	Exhibits a broad substrate specificity, including urate and haem, as well as multiple synthetic compounds [http://www.ncbi.nlm.nih.gov/pubmed/21175590?dopt=AbstractPlus].	Putative functional dependence on ABCG1	The ABCG5/ABCG8 heterodimer transports phytosterols and cholesterol [http://www.ncbi.nlm.nih.gov/pubmed/27144356?dopt=AbstractPlus]. Loss‐of‐function mutations in ABCG5 or ABCG8 are associated with sitosterolemia (http://omim.org/entry/210250).	The ABCG5/ABCG8 heterodimer transports phytosterols and cholesterol [http://www.ncbi.nlm.nih.gov/pubmed/27144356?dopt=AbstractPlus]. Loss‐of‐function mutations in ABCG5 or ABCG8 are associated with sitosterolemia (http://omim.org/entry/210250).

### Comments on ATP‐binding cassette transporter family

A further group of ABC transporter‐like proteins have been identified to lack membrane spanning regions and are not believed to be functional transporters, but appear to have a role in protein translation [http://www.ncbi.nlm.nih.gov/pubmed/16421098?dopt=AbstractPlus, http://www.ncbi.nlm.nih.gov/pubmed/19570978?dopt=AbstractPlus]: https://www.genenames.org/data/gene‐symbol‐report/#!/hgnc_id/HGNC:69 (http://www.uniprot.org/uniprot/P61221, also known as OABP or 2′‐5′ oligoadenylate‐binding protein); https://www.genenames.org/data/gene‐symbol‐report/#!/hgnc_id/HGNC:70 (http://www.uniprot.org/uniprot/Q8NE71, also known as ABC50 or TNF‐a‐stimulated ABC protein); https://www.genenames.org/data/gene‐symbol‐report/#!/hgnc_id/HGNC:71 (http://www.uniprot.org/uniprot/Q9UG63, also known as iron‐inhibited ABC transporter 2) and https://www.genenames.org/data/gene‐symbol‐report/#!/hgnc_id/HGNC:72 (http://www.uniprot.org/uniprot/Q9NUQ8).

### Further reading on ATP‐binding cassette transporter family

Baker A *et al*. (2015) Peroxisomal ABC transporters: functions and mechanism. *Biochem. Soc. Trans*. **43**: 959‐65 https://www.ncbi.nlm.nih.gov/pubmed/26517910?dopt=AbstractPlus

Beis K. (2015) Structural basis for the mechanism of ABC transporters. *Biochem. Soc. Trans*. **43**: 889‐93 https://www.ncbi.nlm.nih.gov/pubmed/26517899?dopt=AbstractPlus

Chen Z *et al*. (2016) Mammalian drug efflux transporters of the ATP binding cassette (ABC) family in multidrug resistance: A review of the past decade. *Cancer Lett*. **370**: 153‐64 https://www.ncbi.nlm.nih.gov/pubmed/26499806?dopt=AbstractPlus

Kemp S *et al*. (2011) Mammalian peroxisomal ABC transporters: from endogenous substrates to pathology and clinical significance. *Br. J. Pharmacol*. **164**: 1753‐66 https://www.ncbi.nlm.nih.gov/pubmed/21488864?dopt=AbstractPlus

Kerr ID *et al*. (2011) The ABCG family of membrane‐associated transporters: you don't have to be big to be mighty. *Br. J. Pharmacol*. **164**: 1767‐79 https://www.ncbi.nlm.nih.gov/pubmed/21175590?dopt=AbstractPlus

Kloudova A *et al*. (2017) The Role of Oxysterols in Human Cancer. *Trends Endocrinol. Metab*. **28**: 485‐496 https://www.ncbi.nlm.nih.gov/pubmed/28410994?dopt=AbstractPlus

López‐Marqués RL *et al*. (2015) Structure and mechanism of ATP‐dependent phospholipid transporters. *Biochim. Biophys. Acta* **1850**: 461‐475 https://www.ncbi.nlm.nih.gov/pubmed/24746984?dopt=AbstractPlus

Neul C *etal*. (2016) Impact of Membrane Drug Transporters on Resistance to Small‐Molecule Tyrosine Kinase Inhibitors. *Trends Pharmacol. Sci*. **37**: 904‐932 https://www.ncbi.nlm.nih.gov/pubmed/27659854?dopt=AbstractPlus

Peña‐Solórzano D *et al*. (2017) ABCG2/BCRP: Specific and Nonspecific Modulators. *Med Res Rev* **37**: 987‐1050 https://www.ncbi.nlm.nih.gov/pubmed/28005280?dopt=AbstractPlus

Robey RW *et al*. (2018) Revisiting the role of ABC transporters in multidrug‐resistant cancer. *Nat Rev Cancer* **18**: 452‐464 [https://www.ncbi.nlm.nih.gov/pubmed/29643473]

Vauthier V *et al*. (2017) Targeted pharmacotherapies for defective ABC transporters. *Biochem. Pharmacol*. **136**: 1‐11 https://www.ncbi.nlm.nih.gov/pubmed/28245962?dopt=AbstractPlus

## http://www.guidetopharmacology.org/GRAC/FamilyDisplayForward?familyId=137

### Overview

The F‐type (ATP synthase) and the V‐type (vacuolar or vesicular proton pump) ATPases, although having distinct sub‐cellular locations and roles, exhibit marked similarities in subunit structure and mechanism. They are both composed of a ‘soluble’ complex (termed F_1_ or V_1_) and a membrane complex (F_o_ or V_o_). Within each ATPase complex, the two individual sectors appear to function as connected opposing rotary motors, coupling catalysis of http://www.guidetopharmacology.org/GRAC/LigandDisplayForward?ligandId=1713 synthesis or hydrolysis to proton transport. Both the F‐type and V‐type ATPases have been assigned enzyme commission number http://www.genome.jp/kegg‐bin/search_brite?option=‐a&search_string=3.6.3.14

## http://www.guidetopharmacology.org/GRAC/FamilyDisplayForward?familyId=156

### Overview

The F‐type ATPase, also known as ATP synthase or ATP phosphohydrolase (H^+^‐transporting), is a mitochondrial membrane‐associated multimeric complex consisting of two domains, an F_0_ channel domain in the membrane and an F_1_ domain extending into the lumen. Proton transport across the inner mitochondrial membrane is used to drive the synthesis of http://www.guidetopharmacology.org/GRAC/LigandDisplayForward?ligandId=1713, although it is also possible for the enzyme to function as an AT‐Pase. The ATP5O subunit (oligomycin sensitivity‐conferring protein, https://www.genenames.org/data/gene‐symbol‐report/#!/hgnc_id/HGNC:850, (http://www.uniprot.org/uniprot/P48047)), acts as a connector between F_1_ and F_0_ motors.

The **F_1_ motor**, responsible for http://www.guidetopharmacology.org/GRAC/LigandDisplayForward?ligandId=1713 turnover, has the subunit composition α3β3γδε.

The **F_0_ motor**, responsible for ion translocation, is complex in mammals, with probably nine subunits centring on A, B, and C subunits in the membrane, together with D, E, F2, F6, G2 and 8 subunits. Multiple pseudogenes for the F_0_ motor proteins have been defined in the human genome.

Information on members of this family may be found in the http://www.guidetopharmacology.org/GRAC/FamilyDisplayForward?familyId=156.

## http://www.guidetopharmacology.org/GRAC/FamilyDisplayForward?familyId=157

### Overview

The V‐type ATPase is most prominently associated with lysosomes in mammals, but also appears to be expressed on the plasma membrane and neuronal synaptic vesicles.

The **V_1_ motor**, for http://www.guidetopharmacology.org/GRAC/LigandDisplayForward?ligandId=1713 turnover, has subunits with a of A‐H.

The**V_0_ motor**, responsible for ion translocation, has six subunits (a‐e).

Information on members of this family may be found in the http://www.guidetopharmacology.org/GRAC/FamilyDisplayForward?familyId=157.

### Further reading on F‐type and V‐type ATPases

Brandt K *et al*. (2015) Hybrid rotors in F1F(o) ATP synthases: subunit composition, distribution, and physiological significance. *Biol. Chem*. **396**: 1031–42 https://www.ncbi.nlm.nih.gov/pubmed/25838297?dopt=AbstractPlus

Krah A. (2015) Linking structural features from mitochondrial and bacterial F‐type ATP synthases to their distinct mechanisms of ATPase inhibition. *Prog. Biophys. Mol. Biol*. **119**: 94–102 https://www.ncbi.nlm.nih.gov/pubmed/26140992?dopt=AbstractPlus

Marshansky V *et al*. (2014) Eukaryotic V‐ATPase: novel structural findings and functional insights. *Biochim. Biophys. Acta* **1837**: 857–79 https://www.ncbi.nlm.nih.gov/pubmed/24508215?dopt=AbstractPlus

Noji H *et al*. (2017) Catalytic robustness and torque generation of the F_1‐ATPase. *Biophys Rev* **9**: 103–118 https://www.ncbi.nlm.nih.gov/pubmed/28424741?dopt=AbstractPlus

Okuno D *et al*. (2013) Single‐molecule analysis of the rotation of F_1‐ATPase under high hydrostatic pressure. *Biophys. J*. **105**: 1635–42 https://www.ncbi.nlm.nih.gov/pubmed/24094404?dopt=AbstractPlus

## http://www.guidetopharmacology.org/GRAC/FamilyDisplayForward?familyId=138

### Overview

Phosphorylation‐type ATPases (EC 3.6.3.‐) are associated with membranes and the transport of ions or phospholipids. Characteristics of the family are the transient phosphorylation of the transporters at an aspartate residue and the interconversion between E1 and E2 conformations in the activity cycle of the transporters, taken to represent ‘half‐channels’ facing the cytoplasm and extracellular/luminal side of the membrane, respectively.

Sequence analysis across multiple species allows the definition of five subfamilies, P1‐P5. The P1 subfamily includes heavy metal pumps, such as the copper ATPases. The P2 subfamily includes calcium, sodium/potassium and proton/potassium pumps. The P4 and P5 subfamilies include putative phospholipid flippases.

## http://www.guidetopharmacology.org/GRAC/FamilyDisplayForward?familyId=158

### Overview

The cell‐surface Na^+^/K^+^‐ATPase is an integral membrane protein which regulates the membrane potential of the cell by maintaining gradients of Na^+^ and K^+^ ions across the plasma membrane, also making a small, direct contribution to membrane potential, particularly in cardiac cells. For every molecule of ATP hydrolysed, the Na^+^/K^+^‐ATPase extrudes three Na^+^ ions and imports two K^+^ ions. The active transporter is a heteromultimer with incompletely defined stoichiometry, possibly as tetramers of heterodimers, each consisting of one of four large, ten TM domain catalytic α subunits and one of three smaller, single TM domain glycoprotein β‐subunits. Additional protein partners known as FXYD proteins (*e.g*. https://www.genenames.org/data/gene‐symbol‐report/#!/hgnc_id/HGNC:4026, http://www.uniprot.org/uniprot/P54710) appear to associate with and regulate the activity of the pump.

Information on members of this family may be found in the http://www.guidetopharmacology.org/GRAC/FamilyDisplayForward?familyId=158.

### Comments

Na^+^/K^+^‐ATPases are inhibited by http://www.guidetopharmacology.org/GRAC/LigandDisplayForward?ligandId=4826 and cardiac glycosides, such as http://www.guidetopharmacology.org/GRAC/LigandDisplayForward?ligandId=4726, as well as potentially endogenous cardiotonic steroids [http://www.ncbi.nlm.nih.gov/pubmed/19325075?dopt=AbstractPlus].

## http://www.guidetopharmacology.org/GRAC/FamilyDisplayForward?familyId=159

### Overview

The sarcoplasmic/endoplasmic reticulum Ca^2^+‐ATPase (SERCA) is an intracellular membrane‐associated pump for sequestering calcium from the cytosol into intracellular organelles, usually associated with the recovery phase following excitation of muscle and nerves.

The plasma membrane Ca^2+^‐ATPase (PMCA) is a cell‐surface pump for extruding calcium from the cytosol, usually associated with the recovery phase following excitation of cells. The active pump is a homodimer, each subunit of which is made up of ten TM segments, with cytosolic C‐ and N‐termini and two large intracellular loops.

Secretory pathway Ca^2+^‐ATPases (SPCA) allow accumulation of calcium and manganese in the Golgi apparatus.

Information on members of this family may be found in the http://www.guidetopharmacology.org/GRAC/FamilyDisplayForward?familyId=159.

### Comments

The fungal toxin http://www.guidetopharmacology.org/GRAC/LigandDisplayForward?ligandId=4672 has been described to activate SERCA in kidney microsomes [http://www.ncbi.nlm.nih.gov/pubmed/1417961?dopt=AbstractPlus]. http://www.guidetopharmacology.org/GRAC/LigandDisplayForward?ligandId=5350 [http://www.ncbi.nlm.nih.gov/pubmed/2530215?dopt=AbstractPlus], http://www.guidetopharmacology.org/GRAC/LigandDisplayForward?ligandId=5351 [http://www.ncbi.nlm.nih.gov/pubmed/1832668?dopt=AbstractPlus] and http://www.guidetopharmacology.org/GRAC/LigandDisplayForward?ligandId=5486are widely employed to block SERCA. Thapsigargin has also been described to block the TRPV1 vanilloid receptor [http://www.ncbi.nlm.nih.gov/pubmed/12054538?dopt=AbstractPlus].

The stoichiometry of flux through the PMCA differs from SERCA, with the PMCA transporting 1 Ca^2+^ while SERCA transports 2 Ca^2+^.

Loss‐of‐function mutations in SPCA1 appear to underlie Hailey‐Hailey disease [http://www.ncbi.nlm.nih.gov/pubmed/10615129?dopt=AbstractPlus].

## http://www.guidetopharmacology.org/GRAC/FamilyDisplayForward?familyId=160

### Overview

The H^+^/K^+^ ATPase is a heterodimeric protein, made up of α and β subunits. The α subunit has 10 TM domains and exhibits catalytic and pore functions, while the β subunit has a single TM domain, which appears to be required for intracellular trafficking and stabilising the α subunit. The ATP4A and ATP4B subunits are expressed together, while the ATP12A subunit is suggested to be expressed with the β1 (ATP1B1) subunit of the Na^+^/K^+^‐ATPase [http://www.ncbi.nlm.nih.gov/pubmed/16525125?dopt=AbstractPlus].

Information on members of this family may be found in the http://www.guidetopharmacology.org/GRAC/FamilyDisplayForward?familyId=160.

### Comments

The gastric H^+^/K^+^‐ATPase is inhibited by proton pump inhibitors used for treating excessive gastric acid secretion, including http://www.guidetopharmacology.org/GRAC/LigandDisplayForward?ligandId=5487 and a metabolite of http://www.guidetopharmacology.org/GRAC/LigandDisplayForward?ligandId=5488.

## http://www.guidetopharmacology.org/GRAC/FamilyDisplayForward?familyId=161

### Overview

Copper‐transporting ATPases convey copper ions across cell‐surface and intracellular membranes. They consist of eight TM domains and associate with multiple copper chaperone proteins (*e.g*. https://www.genenames.org/data/gene‐symbol‐report/#!/hgnc_id/HGNC:798, http://www.uniprot.org/uniprot/O00244).

Information on members of this family may be found in the http://www.guidetopharmacology.org/GRAC/FamilyDisplayForward?familyId=161.

## http://www.guidetopharmacology.org/GRAC/FamilyDisplayForward?familyId=162

### Overview

These transporters are thought to translocate the aminophospholipids phosphatidylserine and phosphatidylethanolamine from one side of the phospholipid bilayer to the other to generate asymmetric membranes. They are also proposed to be involved in the generation of vesicles from intracellular and cell‐surface membranes.

Information on members of this family may be found in the http://www.guidetopharmacology.org/GRAC/FamilyDisplayForward?familyId=162.

### Comments

Loss‐of‐functionmutations in ATP8B1 are associated with type I familial intrahepatic cholestasis.

A further series of structurally‐related proteins have been identified in the human genome, with as yet undefined function, including https://www.genenames.org/data/gene‐symbol‐report/#!/hgnc_id/HGNC:24215 (http://www.uniprot.org/uniprot/Q9HD20), https://www.genenames.org/data/gene‐symbol‐report/#!/hgnc_id/HGNC:30213 (http://www.uniprot.org/uniprot/Q9NQ11), https://www.genenames.org/data/gene‐symbol‐report/#!/hgnc_id/HGNC:24113 (http://www.uniprot.org/uniprot/Q9H7F0), https://www.genenames.org/data/gene‐symbol‐report/#!/hgnc_id/HGNC:25422 (http://www.uniprot.org/uniprot/Q4VNC1) and https://www.genenames.org/data/gene‐symbol‐report/#!/hgnc_id/HGNC:31789 (http://www.uniprot.org/uniprot/Q4VNC0).

### Further reading on P‐type ATPases

Aperia A *et al*. (2016) Na+‐K+‐ATPase, a new class of plasma membrane receptors. *Am. J. Physiol*., Cell Physiol. **310**: C491–5 https://www.ncbi.nlm.nih.gov/pubmed/26791490?dopt=AbstractPlus

Brini M *et al*. (2017) The plasma membrane calcium pumps: focus on the role in (neuro)pathology. *Biochem. Biophys. Res. Commun*. **483**: 1116–1124 https://www.ncbi.nlm.nih.gov/pubmed/27480928?dopt=AbstractPlus

Bruce JIE. (2018) Metabolic regulation of the PMCA: Role in cell death and survival. *Cell Calcium* **69**: 28–36 https://www.ncbi.nlm.nih.gov/pubmed/27553475?dopt=AbstractPlus

Diederich M *et al*. (2017) Cardiac glycosides: From molecular targets to immunogenic cell death. *Biochem. Pharmacol*. **125**: 1–11 https://www.ncbi.nlm.nih.gov/pubmed/27553475?dopt=AbstractPlus

Dubois C *et al*. (2016) The calcium‐signaling toolkit: Updates needed. *Biochim. Biophys. Acta* **1863**: 1337–43 https://www.ncbi.nlm.nih.gov/pubmed/26658643?dopt=AbstractPlus

Krebs J. (2015) The plethora of PMCA isoforms: Alternative splicing and differential expression. *Biochim. Biophys. Acta* **1853**: 2018–24 https://www.ncbi.nlm.nih.gov/pubmed/25535949?dopt=AbstractPlus

Little R *et al*. (2016) Plasma membrane calcium ATPases (PMCAs) as potential targets for the treatment of essential hypertension. *Pharmacol. Ther*. **159**: 23–34 https://www.ncbi.nlm.nih.gov/pubmed/26820758?dopt=AbstractPlus

López‐Marqués RL *et al*. (2015) Structure and mechanism of ATP‐dependent phospholipid transporters. *Biochim. Biophys. Acta* **1850**: 461–475 https://www.ncbi.nlm.nih.gov/pubmed/24746984?dopt=AbstractPlus

Migocka M. (2015) Copper‐transporting ATPases: The evolutionarily conserved machineries for balancing copper in living systems. *IUBMB Life* **67**: 737–45 https://www.ncbi.nlm.nih.gov/pubmed/26422816?dopt=AbstractPlus

Padányi R *et al*. (2016) Multifaceted plasmamembrane Ca(2+) pumps: From structure to intracellular Ca(2+) handling and cancer. *Biochim. Biophys. Acta* **1863**: 1351–63 https://www.ncbi.nlm.nih.gov/pubmed/26707182?dopt=AbstractPlus

Pomorski TG *et al*. (2016) Lipid somersaults: Uncovering the mechanisms of protein‐mediated lipid flipping. *Prog. Lipid Res*. **64**: 69–84 https://www.ncbi.nlm.nih.gov/pubmed/27528189?dopt=AbstractPlus

Retamales‐Ortega R *et al*. (2016) P2C‐Type ATPases and Their Regulation. *Mol. Neurobiol*. **53**: 1343–54 https://www.ncbi.nlm.nih.gov/pubmed/25631710?dopt=AbstractPlus

Tadini‐Buoninsegni F *et al*. (2017) Mechanisms of charge transfer in human copper ATPases ATP7A and ATP7B. *IUBMB Life* **69**: 218–225 https://www.ncbi.nlm.nih.gov/pubmed/28164426?dopt=AbstractPlus

## http://www.guidetopharmacology.org/GRAC/FamilyDisplayForward?familyId=863

### Overview

The SLC superfamily of solute carriers is the second largest family of membrane proteins after G protein‐coupled receptors, but with a great deal fewer therapeutic drugs that exploit them. As with the ABC transporters, however, they play a major role in drug disposition and so can be hugely influential in determining the clinical efficacy of particular drugs.

48 families are identified on the basis of sequence similarities, but many of them overlap in terms of the solutes that they carry. For example, amino acid accumulation is mediated by members of the SLC1, SLC3/7, SLC6, SLC15, SLC16, SLC17, SLC32, SLC36, SLC38 and SLC43. Further members of the SLC superfamily regulate ion fluxes at the plasma membrane, or solute transport into and out of cellular organelles.

Within the SLC superfamily, there is an abundance in diversity of structure. Two families (SLC3 and SLC7) only generate functional transporters as heteromeric partners, where one partner is a single TMdomain protein. Membrane topology predictions for other families suggest 3, 4 6, 7, 8, 9, 10, 11, 12, 13, or 14 TM domains.

Functionally, members may be divided into those dependent on gradients of ions (particularly sodium, chloride or protons), exchange of solutes or simple equilibrative gating. For many members, the stoichiometry of transport is not yet established. Furthermore, one family of transporters also possess enzymatic activity (SLC27), while many members function as ion channels (e.g. SLC1A7/EAAT5), which increases the complexity of function of the SLC superfamily.

## http://www.guidetopharmacology.org/GRAC/FamilyDisplayForward?familyId=139

### Overview

The SLC1 family of sodium dependent transporters includes the plasma membrane located glutamate transporters and the neutral amino acid transporters ASCT1 and ASCT2 [http://www.ncbi.nlm.nih.gov/pubmed/8103691?dopt=AbstractPlus, http://www.ncbi.nlm.nih.gov/pubmed/17088867?dopt=AbstractPlus, http://www.ncbi.nlm.nih.gov/pubmed/14530974?dopt=AbstractPlus, http://www.ncbi.nlm.nih.gov/pubmed/14612154?dopt=AbstractPlus, http://www.ncbi.nlm.nih.gov/pubmed/9790568?dopt=AbstractPlus].

## http://www.guidetopharmacology.org/GRAC/FamilyDisplayForward?familyId=163

### Overview

Glutamate transporters present the unusual structural motif of 8TM segments and 2 re‐entrant loops [http://www.ncbi.nlm.nih.gov/pubmed/10734120?dopt=AbstractPlus]. The crystal structure of a glutamate transporter homologue (GltPh) from *Pyrococcus horikoshii* supports this topology and indicates that the transporter assembles as a trimer, where each monomer is a functional unit capable of substrate permeation [http://www.ncbi.nlm.nih.gov/pubmed/17230192?dopt=AbstractPlus, http://www.ncbi.nlm.nih.gov/pubmed/19924125?dopt=AbstractPlus, http://www.ncbi.nlm.nih.gov/pubmed/15483603?dopt=AbstractPlus] reviewed by [http://www.ncbi.nlm.nih.gov/pubmed/20708631?dopt=AbstractPlus]). This structural data is in agreementwith the proposed quaternary structure for EAAT2 [http://www.ncbi.nlm.nih.gov/pubmed/15265858?dopt=AbstractPlus] and several functional studies that propose the monomer is the functional unit [http://www.ncbi.nlm.nih.gov/pubmed/16128593?dopt=AbstractPlus, http://www.ncbi.nlm.nih.gov/pubmed/17360917?dopt=AbstractPlus, http://www.ncbi.nlm.nih.gov/pubmed/17360916?dopt=AbstractPlus, http://www.ncbi.nlm.nih.gov/pubmed/14982939?dopt=AbstractPlus]. Recent evidence suggests that EAAT3 and EAAT4 may assemble as heterotrimers [http://www.ncbi.nlm.nih.gov/pubmed/21127051?dopt=AbstractPlus]. The activity of glutamate transporters located upon both neurones (predominantly EAAT3, 4 and 5) and glia (predominantly EAAT 1 and 2) serves, dependent upon their location, to regulate excitatory neurotransmission, maintain low ambient extracellular concentrations of glutamate (protecting against excitotoxicity) and provide glutamate for metabolism including the glutamate‐glutamine cycle. The http://www.guidetopharmacology.org/GRAC/FamilyDisplayForward?familyId=138 that maintains the ion gradients that drive transport has been demonstrated to co‐assemble with EAAT1 and EAAT2 [http://www.ncbi.nlm.nih.gov/pubmed/19553454?dopt=AbstractPlus]. Recent evidence supports altered glutamate transport and novel roles in brain for splice variants of EAAT1 and EAAT2 [http://www.ncbi.nlm.nih.gov/pubmed/20688910?dopt=AbstractPlus, http://www.ncbi.nlm.nih.gov/pubmed/20883814?dopt=AbstractPlus]. Three patients with dicarboxylic aminoaciduria (DA) were recently found to have loss‐of‐function mutations in EAAT3 [http://www.ncbi.nlm.nih.gov/pubmed/21123949?dopt=AbstractPlus]. DA is characterized by excessive excretion of the acidic amino acids glutamate and aspartate and EAAT3 is the predominant glutamate/aspartate transporter in the kidney. Enhanced expression of EAAT2 resulting fromadministration of ß‐lactam antibiotics (e.g. http://www.guidetopharmacology.org/GRAC/LigandDisplayForward?ligandId=5326) is neuroprotective and occurs through NF‐?B‐mediated EAAT2 promoter activation [http://www.ncbi.nlm.nih.gov/pubmed/16274998?dopt=AbstractPlus, http://www.ncbi.nlm.nih.gov/pubmed/18326497?dopt=AbstractPlus, http://www.ncbi.nlm.nih.gov/pubmed/15635412?dopt=AbstractPlus] reviewed by [http://www.ncbi.nlm.nih.gov/pubmed/21792905?dopt=AbstractPlus]). http://www.guidetopharmacology.org/GRAC/FamilyDisplayForward?familyId=86 activation (*e.g*. by http://www.guidetopharmacology.org/GRAC/LigandDisplayForward?ligandId=1056) also leads to enhanced expression of EAAT though promoter activation [http://www.ncbi.nlm.nih.gov/pubmed/17213861?dopt=AbstractPlus]. In addition, several translational activators of EAAT2 have recently been described [http://www.ncbi.nlm.nih.gov/pubmed/20508255?dopt=AbstractPlus] along with treatments that increase the surface expression of EAAT2 (*e.g*. [http://www.ncbi.nlm.nih.gov/pubmed/21309758?dopt=AbstractPlus, http://www.ncbi.nlm.nih.gov/pubmed/21426345?dopt=AbstractPlus]), or prevent its down‐regulation (*e.g*. [http://www.ncbi.nlm.nih.gov/pubmed/21730107?dopt=AbstractPlus]). A thermodynamically uncoupled Cl‐ flux, activated by Na^+^ and glutamate [http://www.ncbi.nlm.nih.gov/pubmed/15834685?dopt=AbstractPlus, http://www.ncbi.nlm.nih.gov/pubmed/14612154?dopt=AbstractPlus, http://www.ncbi.nlm.nih.gov/pubmed/21572047?dopt=AbstractPlus] (Na+ and aspartate in the case of GltPh [http://www.ncbi.nlm.nih.gov/pubmed/21730107?dopt=AbstractPlus]), is sufficiently large, in the instances of EAAT4 and EAAT5, to influence neuronal excitability [http://www.ncbi.nlm.nih.gov/pubmed/17908688?dopt=AbstractPlus, http://www.ncbi.nlm.nih.gov/pubmed/17041592?dopt=AbstractPlus]. Indeed, it has recently been suggested that the primary function of EAAT5 is as a slow anion channel gated by glutamate, rather than a glutamate transporter [http://www.ncbi.nlm.nih.gov/pubmed/21641307?dopt=AbstractPlus].

**Table:** 

Nomenclature	http://www.guidetopharmacology.org/GRAC/ObjectDisplayForward?objectId=868	http://www.guidetopharmacology.org/GRAC/ObjectDisplayForward?objectId=869	http://www.guidetopharmacology.org/GRAC/ObjectDisplayForward?objectId=870	http://www.guidetopharmacology.org/GRAC/ObjectDisplayForward?objectId=871	http://www.guidetopharmacology.org/GRAC/ObjectDisplayForward?objectId=872
Systematic nomenclature	SLC1A3	SLC1A2	SLC1A1	SLC1A6	SLC1A7
Common abbreviation	EAAT1	EAAT2	EAAT3	EAAT4	EAAT5
HGNC, UniProt	https://www.genenames.org/data/gene‐symbol‐report/#!/hgnc_id/HGNC:10941, http://www.uniprot.org/uniprot/P43003	https://www.genenames.org/data/gene‐symbol‐report/#!/hgnc_id/HGNC:10940, http://www.uniprot.org/uniprot/P43004	https://www.genenames.org/data/gene‐symbol‐report/#!/hgnc_id/HGNC:10939, http://www.uniprot.org/uniprot/P43005	https://www.genenames.org/data/gene‐symbol‐report/#!/hgnc_id/HGNC:10944, http://www.uniprot.org/uniprot/P48664	https://www.genenames.org/data/gene‐symbol‐report/#!/hgnc_id/HGNC:10945, http://www.uniprot.org/uniprot/O00341
Substrates	http://www.guidetopharmacology.org/GRAC/LigandDisplayForward?ligandId=4497 (*K*_i_ 5.8×10^‐5^M) [http://www.ncbi.nlm.nih.gov/pubmed/9463476?dopt=AbstractPlus], http://www.guidetopharmacology.org/GRAC/LigandDisplayForward?ligandId=6511, http://www.guidetopharmacology.org/GRAC/LigandDisplayForward?ligandId=4516	http://www.guidetopharmacology.org/GRAC/LigandDisplayForward?ligandId=6511, http://www.guidetopharmacology.org/GRAC/LigandDisplayForward?ligandId=4497, http://www.guidetopharmacology.org/GRAC/LigandDisplayForward?ligandId=4516 [http://www.ncbi.nlm.nih.gov/pubmed/10570036?dopt=AbstractPlus]	http://www.guidetopharmacology.org/GRAC/LigandDisplayForward?ligandId=4516, http://www.guidetopharmacology.org/GRAC/LigandDisplayForward?ligandId=4497, http://www.guidetopharmacology.org/GRAC/LigandDisplayForward?ligandId=6511	http://www.guidetopharmacology.org/GRAC/LigandDisplayForward?ligandId=6511, http://www.guidetopharmacology.org/GRAC/LigandDisplayForward?ligandId=4497, http://www.guidetopharmacology.org/GRAC/LigandDisplayForward?ligandId=4516	http://www.guidetopharmacology.org/GRAC/LigandDisplayForward?ligandId=6511, http://www.guidetopharmacology.org/GRAC/LigandDisplayForward?ligandId=4516, http://www.guidetopharmacology.org/GRAC/LigandDisplayForward?ligandId=4497
Endogenous substrates	http://www.guidetopharmacology.org/GRAC/LigandDisplayForward?ligandId=3309, http://www.guidetopharmacology.org/GRAC/LigandDisplayForward?ligandId=1369	http://www.guidetopharmacology.org/GRAC/LigandDisplayForward?ligandId=1369, http://www.guidetopharmacology.org/GRAC/LigandDisplayForward?ligandId=3309	http://www.guidetopharmacology.org/GRAC/LigandDisplayForward?ligandId=3309, http://www.guidetopharmacology.org/GRAC/LigandDisplayForward?ligandId=4782 [http://www.ncbi.nlm.nih.gov/pubmed/8782106?dopt=AbstractPlus], http://www.guidetopharmacology.org/GRAC/LigandDisplayForward?ligandId=1369	http://www.guidetopharmacology.org/GRAC/LigandDisplayForward?ligandId=1369, http://www.guidetopharmacology.org/GRAC/LigandDisplayForward?ligandId=3309	http://www.guidetopharmacology.org/GRAC/LigandDisplayForward?ligandId=3309, http://www.guidetopharmacology.org/GRAC/LigandDisplayForward?ligandId=1369
Stoichiometry	Probably 3 Na^+^: 1 H^+^: 1 glutamate (in): 1 K^+^ (out)	3 Na^+^: 1 H^+^: 1 glutamate (in): 1 K^+^ (out) [http://www.ncbi.nlm.nih.gov/pubmed/9822723?dopt=AbstractPlus]	3 Na^+^: 1 H^+^: 1 glutamate (in): 1 K^+^ (out) [http://www.ncbi.nlm.nih.gov/pubmed/8857541?dopt=AbstractPlus]	Probably 3 Na^+^: 1 H^+^: 1 glutamate (in): 1 K^+^(out)	Probably 3 Na^+^: 1 H^+^: 1 glutamate (in): 1 K^+^ (out)
Inhibitors	http://www.guidetopharmacology.org/GRAC/LigandDisplayForward?ligandId=4609 (membrane potential assay) (pIC_50_ 6.9) [http://www.ncbi.nlm.nih.gov/pubmed/19161278?dopt=AbstractPlus], http://www.guidetopharmacology.org/GRAC/LigandDisplayForward?ligandId=4631 (p*K*_B_ 5) [http://www.ncbi.nlm.nih.gov/pubmed/9463476?dopt=AbstractPlus]	http://www.guidetopharmacology.org/GRAC/LigandDisplayForward?ligandId=4531 (pIC_50_ 7.1) [http://www.ncbi.nlm.nih.gov/pubmed/16014807?dopt=AbstractPlus], http://www.guidetopharmacology.org/GRAC/LigandDisplayForward?ligandId=4631 (p*K*_B_ 6.9) [http://www.ncbi.nlm.nih.gov/pubmed/9463476?dopt=AbstractPlus], http://www.guidetopharmacology.org/GRAC/LigandDisplayForward?ligandId=4317 (p*K*_B_ 5.5) [http://www.ncbi.nlm.nih.gov/pubmed/9145919?dopt=AbstractPlus], http://www.guidetopharmacology.org/GRAC/LigandDisplayForward?ligandId=4571 (p*K*_B_ 5), http://www.guidetopharmacology.org/GRAC/LigandDisplayForward?ligandId=4573 (p*K*_B_ 4.7) [http://www.ncbi.nlm.nih.gov/pubmed/9145919?dopt=AbstractPlus]	http://www.guidetopharmacology.org/GRAC/LigandDisplayForward?ligandId=4626 (pIC50 7.1) [http://www.ncbi.nlm.nih.gov/pubmed/17017964?dopt=AbstractPlus], http://www.guidetopharmacology.org/GRAC/LigandDisplayForward?ligandId=4625 ([^3^H]D‐aspartate uptake assay) (p*K*_i_ 6.1) [http://www.ncbi.nlm.nih.gov/pubmed/16183084?dopt=AbstractPlus], http://www.guidetopharmacology.org/GRAC/LigandDisplayForward?ligandId=4631 (pIC_50_ 5.1) [http://www.ncbi.nlm.nih.gov/pubmed/11078189?dopt=AbstractPlus]	http://www.guidetopharmacology.org/GRAC/LigandDisplayForward?ligandId=4631 (p*K*_i_ 5.4) [http://www.ncbi.nlm.nih.gov/pubmed/11677257?dopt=AbstractPlus], http://www.guidetopharmacology.org/GRAC/LigandDisplayForward?ligandId=4573 (p*K*_i_ 4.3) [http://www.ncbi.nlm.nih.gov/pubmed/11299317?dopt=AbstractPlus]	http://www.guidetopharmacology.org/GRAC/LigandDisplayForward?ligandId=4631 (p*K*_i_ 5.5) [http://www.ncbi.nlm.nih.gov/pubmed/11677257?dopt=AbstractPlus]
Labelled ligands	http://www.guidetopharmacology.org/GRAC/LigandDisplayForward?ligandId=4492 (Binding) (p*K*_d_ 7.8) [http://www.ncbi.nlm.nih.gov/pubmed/17047096?dopt=AbstractPlus] – Rat, http://www.guidetopharmacology.org/GRAC/LigandDisplayForward?ligandId=4698, http://www.guidetopharmacology.org/GRAC/LigandDisplayForward?ligandId=4534, http://www.guidetopharmacology.org/GRAC/LigandDisplayForward?ligandId=4075	http://www.guidetopharmacology.org/GRAC/LigandDisplayForward?ligandId=4492 (Binding) (p*K*_d_ 7.8) [http://www.ncbi.nlm.nih.gov/pubmed/17047096?dopt=AbstractPlus] – Rat, http://www.guidetopharmacology.org/GRAC/LigandDisplayForward?ligandId=4534, http://www.guidetopharmacology.org/GRAC/LigandDisplayForward?ligandId=4534, http://www.guidetopharmacology.org/GRAC/LigandDisplayForward?ligandId=4075	http://www.guidetopharmacology.org/GRAC/LigandDisplayForward?ligandId=4492 (Binding) (p*K*_d_ 6.5) [http://www.ncbi.nlm.nih.gov/pubmed/17047096?dopt=AbstractPlus] – Rat, http://www.guidetopharmacology.org/GRAC/LigandDisplayForward?ligandId=4698, http://www.guidetopharmacology.org/GRAC/LigandDisplayForward?ligandId=4534	http://www.guidetopharmacology.org/GRAC/LigandDisplayForward?ligandId=4492 (Binding) (p*K*_d_ 7.9) [http://www.ncbi.nlm.nih.gov/pubmed/17047096?dopt=AbstractPlus] – Rat, http://www.guidetopharmacology.org/GRAC/LigandDisplayForward?ligandId=4698, http://www.guidetopharmacology.org/GRAC/LigandDisplayForward?ligandId=4534	http://www.guidetopharmacology.org/GRAC/LigandDisplayForward?ligandId=4492 (Binding) (p*K*_d_ 7.6) [http://www.ncbi.nlm.nih.gov/pubmed/17047096?dopt=AbstractPlus] – Rat, http://www.guidetopharmacology.org/GRAC/LigandDisplayForward?ligandId=4698, http://www.guidetopharmacology.org/GRAC/LigandDisplayForward?ligandId=4534

### Comments

The K_B_ (or K_i_) values reported, unless indicated otherwise, are derived from transporter currents mediated by EAATs expressed in voltage‐clamped *Xenopus* laevis oocytes [http://www.ncbi.nlm.nih.gov/pubmed/11299317?dopt=AbstractPlus, http://www.ncbi.nlm.nih.gov/pubmed/11677257?dopt=AbstractPlus, http://www.ncbi.nlm.nih.gov/pubmed/9463476?dopt=AbstractPlus, http://www.ncbi.nlm.nih.gov/pubmed/9145919?dopt=AbstractPlus]. K_B_ (or K_i_) values derived in uptake assays are generally higher (*e.g*. [http://www.ncbi.nlm.nih.gov/pubmed/9463476?dopt=AbstractPlus]). In addition to acting as a poorly transportable inhibitor of EAAT2, (2S,4R)‐4‐methylglutamate, also known as http://www.guidetopharmacology.org/GRAC/LigandDisplayForward?ligandId=4317, is a competitive substrate for EAAT1 (K_M_ = 54μM; [http://www.ncbi.nlm.nih.gov/pubmed/19074430?dopt=AbstractPlus, http://www.ncbi.nlm.nih.gov/pubmed/9145919?dopt=AbstractPlus]) and additionally is a potent kainate receptor agonist [http://www.ncbi.nlm.nih.gov/pubmed/8996224?dopt=AbstractPlus] which renders the compound unsuitable for autoradiographic localisation of EAATs [http://www.ncbi.nlm.nih.gov/pubmed/17590480?dopt=AbstractPlus]. Similarly, at concentrations that inhibit EAAT2, dihydrokainate binds to kainate receptors [http://www.ncbi.nlm.nih.gov/pubmed/9463476?dopt=AbstractPlus]. http://www.guidetopharmacology.org/GRAC/LigandDisplayForward?ligandId=5327 and http://www.guidetopharmacology.org/GRAC/LigandDisplayForward?ligandId=4531 are both non‐substrate inhibitors with a preference for EAAT2 over EAAT3 and EAAT1 [http://www.ncbi.nlm.nih.gov/pubmed/14517179?dopt=AbstractPlus, http://www.ncbi.nlm.nih.gov/pubmed/16014807?dopt=AbstractPlus]. http://www.guidetopharmacology.org/GRAC/LigandDisplayForward?ligandId=4626 is a non‐substrate inhibitor with modest selectivity for EAAT3 over EAAT1 (>10‐fold) and EAAT2 (5‐fold) [126, http://www.ncbi.nlm.nih.gov/pubmed/16368269?dopt=AbstractPlus]. Analogously, L‐β‐*threo*‐benzyl‐aspartate (http://www.guidetopharmacology.org/GRAC/LigandDisplayForward?ligandId=4625) is a competitive nonsubstrate inhibitor that preferentially blocks EAAT3 versus EAAT1, or EAAT2 [http://www.ncbi.nlm.nih.gov/pubmed/16183084?dopt=AbstractPlus]. http://www.guidetopharmacology.org/GRAC/LigandDisplayForward?ligandId=4075 demonstrates low affinity binding (K_D_≅6.0 μM) to EAAT1 and EAAT2 in rat brain homogenates [http://www.ncbi.nlm.nih.gov/pubmed/11389172?dopt=AbstractPlus] and EAAT1 in murine astrocyte membranes [http://www.ncbi.nlm.nih.gov/pubmed/14994336?dopt=AbstractPlus], whereas http://www.guidetopharmacology.org/GRAC/LigandDisplayForward?ligandId=4492 binds with high affinity to all EAATs other than EAAT3 [http://www.ncbi.nlm.nih.gov/pubmed/17047096?dopt=AbstractPlus]. The novel isoxazole derivative http://www.guidetopharmacology.org/GRAC/LigandDisplayForward?ligandId=5328 may interact at the same site as TBOA and preferentially inhibit reverse transport of glutamate [http://www.ncbi.nlm.nih.gov/pubmed/18451317?dopt=AbstractPlus]. http://www.guidetopharmacology.org/GRAC/LigandDisplayForward?ligandId=4573 induces substrate‐like currents at EAAT4, but does not elicit heteroexchange of [^3^H]‐aspartate in synaptosome preparations, inconsistentwith the behaviour of a substrate inhibitor [http://www.ncbi.nlm.nih.gov/pubmed/11299317?dopt=AbstractPlus]. http://www.guidetopharmacology.org/GRAC/LigandDisplayForward?ligandId=5352, a compound isolated from the venom from the spider *Parawixia bistriata* is a selective enhancer of the glutamate uptake through EAAT2 but not through EAAT1 or EAAT3 [http://www.ncbi.nlm.nih.gov/pubmed/17646426?dopt=AbstractPlus, http://www.ncbi.nlm.nih.gov/pubmed/12890709?dopt=AbstractPlus]. In addition to the agents listed in the table, http://www.guidetopharmacology.org/GRAC/LigandDisplayForward?ligandId=4497 and http://www.guidetopharmacology.org/GRAC/LigandDisplayForward?ligandId=4516 act as non‐selective competitive substrate inhibitors of all EAATs. Zn^2+^ and http://www.guidetopharmacology.org/GRAC/LigandDisplayForward?ligandId=2391 are putative endogenous modulators of EAATs with actions that differ across transporter subtypes (reviewed by [http://www.ncbi.nlm.nih.gov/pubmed/15324920?dopt=AbstractPlus]).

## http://www.guidetopharmacology.org/GRAC/FamilyDisplayForward?familyId=164

### Overview

ASC transporters mediate Na^+^‐dependent exchange of small neutral amino acids such as Ala, Ser, Cys and Thr and their structure is predicted to be similar to that of the glutamate transporters [http://www.ncbi.nlm.nih.gov/pubmed/8101838?dopt=AbstractPlus, http://www.ncbi.nlm.nih.gov/pubmed/8662767?dopt=AbstractPlus]. ASCT1 and ASCT2 also exhibit thermodynamically uncoupled chloride channel activity associated with substrate transport [http://www.ncbi.nlm.nih.gov/pubmed/10698697?dopt=AbstractPlus, http://www.ncbi.nlm.nih.gov/pubmed/8910405?dopt=AbstractPlus]. Whereas EAATs counter‐transport K^+^ (see above) ASCTs do not and their function is independent of the intracellular concentration of K^+^ [http://www.ncbi.nlm.nih.gov/pubmed/8910405?dopt=AbstractPlus].

**Table:** 

Nomenclature	http://www.guidetopharmacology.org/GRAC/ObjectDisplayForward?objectId=873	http://www.guidetopharmacology.org/GRAC/ObjectDisplayForward?objectId=874
Systematic nomenclature	SLC1A4	SLC1A5
Common abbreviation	ASCT1	ASCT2
HGNC, UniProt	https://www.genenames.org/data/gene‐symbol‐report/#!/hgnc_id/HGNC:10942, http://www.uniprot.org/uniprot/P43007	https://www.genenames.org/data/gene‐symbol‐report/#!/hgnc_id/HGNC:10943, http://www.uniprot.org/uniprot/Q15758
Endogenous substrates	http://www.guidetopharmacology.org/GRAC/LigandDisplayForward?ligandId=4782 > http://www.guidetopharmacology.org/GRAC/LigandDisplayForward?ligandId=720 = http://www.guidetopharmacology.org/GRAC/LigandDisplayForward?ligandId=726 > http://www.guidetopharmacology.org/GRAC/LigandDisplayForward?ligandId=4785	http://www.guidetopharmacology.org/GRAC/LigandDisplayForward?ligandId=720 = http://www.guidetopharmacology.org/GRAC/LigandDisplayForward?ligandId=726 = http://www.guidetopharmacology.org/GRAC/LigandDisplayForward?ligandId=4782 (low Vmax) = http://www.guidetopharmacology.org/GRAC/LigandDisplayForward?ligandId=4785 = http://www.guidetopharmacology.org/GRAC/LigandDisplayForward?ligandId=723 = http://www.guidetopharmacology.org/GRAC/LigandDisplayForward?ligandId=4533 ≫ http://www.guidetopharmacology.org/GRAC/LigandDisplayForward?ligandId=4814 ≅ http://www.guidetopharmacology.org/GRAC/LigandDisplayForward?ligandId=727 ≅ http://www.guidetopharmacology.org/GRAC/LigandDisplayForward?ligandId=3312 > http://www.guidetopharmacology.org/GRAC/LigandDisplayForward?ligandId=4794 > http://www.guidetopharmacology.org/GRAC/LigandDisplayForward?ligandId=1369 (enhanced at low pH)
Stoichiometry	1 Na^+^: 1 amino acid (in): 1 Na^+^: 1 amino acid (out); (homo‐, or hetero‐exchange; [http://www.ncbi.nlm.nih.gov/pubmed/8857541?dopt=AbstractPlus])	1 Na^+^: 1 amino acid (in): 1 Na^+^: 1 amino acid (out); (homo‐, or hetero‐exchange; [http://www.ncbi.nlm.nih.gov/pubmed/10537079?dopt=AbstractPlus])
Inhibitors	–	http://www.guidetopharmacology.org/GRAC/LigandDisplayForward?ligandId=4508 (p*K*_i_ 4.3) [http://www.ncbi.nlm.nih.gov/pubmed/15670919?dopt=AbstractPlus] – Rat, http://www.guidetopharmacology.org/GRAC/LigandDisplayForward?ligandId=4501 (p*K*_i_ 3.1) [http://www.ncbi.nlm.nih.gov/pubmed/15107471?dopt=AbstractPlus], http://www.guidetopharmacology.org/GRAC/LigandDisplayForward?ligandId=4500 (p*K*_i_ 3) [http://www.ncbi.nlm.nih.gov/pubmed/15107471?dopt=AbstractPlus]

### Comments

The substrate specificity of ASCT1 may extend to http://www.guidetopharmacology.org/GRAC/LigandDisplayForward?ligandId=3314 and http://www.guidetopharmacology.org/GRAC/LigandDisplayForward?ligandId=4704 [http://www.ncbi.nlm.nih.gov/pubmed/14502423?dopt=AbstractPlus]. At low pH (5.5) both ASCT1 and ASCT2 are able to exchange acidic amino acids such as http://www.guidetopharmacology.org/GRAC/LigandDisplayForward?ligandId=5329 and glutamate [http://www.ncbi.nlm.nih.gov/pubmed/8603078?dopt=AbstractPlus, http://www.ncbi.nlm.nih.gov/pubmed/8662767?dopt=AbstractPlus]. In addition to the inhibitors tabulated above, HgCl_2_, methylmercury and http://www.guidetopharmacology.org/GRAC/LigandDisplayForward?ligandId=5331, at low micromolar concentrations, non‐competitively inhibit ASCT2 by covalent modificiation of cysteine residues [http://www.ncbi.nlm.nih.gov/pubmed/20599776?dopt=AbstractPlus].

### Further reading on SLC1 family of amino acid transporters

Beart PM *et al*. (2007) Transporters for L‐glutamate: an update on their molecular pharmacology and pathological involvement. *Br. J. Pharmacol*. **150**: 5–17 https://www.ncbi.nlm.nih.gov/pubmed/17088867?dopt=AbstractPlus

Bjrn‐Yoshimoto WE *et al*. (2016) The importance of the excitatory amino acid transporter 3 (EAAT3). *Neurochem. Int*. **98**: 4–18 https://www.ncbi.nlm.nih.gov/pubmed/27233497?dopt=AbstractPlus

Fahlke C *et al*. (2016) Molecular physiology of EAAT anion channels. *Pflugers Arch*. **468**: 491–502 https://www.ncbi.nlm.nih.gov/pubmed/26687113?dopt=AbstractPlus

Fontana AC. (2015) Current approaches to enhance glutamate transporter function and expression. *J. Neurochem*. **134**: 982–1007 https://www.ncbi.nlm.nih.gov/pubmed/26096891?dopt=AbstractPlus

Grewer C *et al*. (2014) SLC1 glutamate transporters. *Pflugers Arch*. **466**: 3–24 https://www.ncbi.nlm.nih.gov/pubmed/24240778?dopt=AbstractPlus

Jensen AA *et al*. (2015) Excitatory amino acid transporters: recent insights into molecular mechanisms, novel modes of modulation and new therapeutic possibilities. *Curr Opin Pharmacol* **20**: 116–23 https://www.ncbi.nlm.nih.gov/pubmed/25466154?dopt=AbstractPlus

Kanai Y *et al*. (2013) The SLC1 high‐affinity glutamate and neutral amino acid transporter family. *Mol. Aspects Med*. **34**: 108–20 https://www.ncbi.nlm.nih.gov/pubmed/23506861?dopt=AbstractPlus

Takahashi K *et al*. (2015) Glutamate transporter EAAT2: regulation, function, and potential as a therapeutic target for neurological and psychiatric disease. *Cell. Mol. Life Sci*. **72**: 3489–506 https://www.ncbi.nlm.nih.gov/pubmed/26033496?dopt=AbstractPlus

## http://www.guidetopharmacology.org/GRAC/FamilyDisplayForward?familyId=140

### Overview

The SLC2 family transports http://www.guidetopharmacology.org/GRAC/LigandDisplayForward?ligandId=4536, http://www.guidetopharmacology.org/GRAC/LigandDisplayForward?ligandId=4654, inositol (*e.g*. http://www.guidetopharmacology.org/GRAC/LigandDisplayForward?ligandId=4495) and related hexoses. Three classes of glucose transporter can be identified, separating GLUT1‐4 and 14, GLUT6, 8, 10 and 12; and GLUT5, 7, 9 and 11. Modelling suggests a 12 TM membrane topology, with intracellular termini, with functional transporters acting as homodimers or homotetramers.

## http://www.guidetopharmacology.org/GRAC/FamilyDisplayForward?familyId=165

### Overview

Class I transporters are able to transport http://www.guidetopharmacology.org/GRAC/LigandDisplayForward?ligandId=4536, but not http://www.guidetopharmacology.org/GRAC/LigandDisplayForward?ligandId=4654, in the direction of the concentration gradient and may be inhibited non‐selectively by http://www.guidetopharmacology.org/GRAC/LigandDisplayForward?ligandId=4285 and http://www.guidetopharmacology.org/GRAC/LigandDisplayForward?ligandId=5334. GLUT1 is the major glucose transporter in brain, placenta and erythrocytes, GLUT2 is found in the pancreas, liver and kidneys, GLUT3 is neuronal and placental, while GLUT4 is the insulin‐responsive transporter found in skeletal muscle, heart and adipose tissue. GLUT14 appears to result from gene duplication of GLUT3 and is expressed in the testes [http://www.ncbi.nlm.nih.gov/pubmed/12504846?dopt=AbstractPlus].

**Table:** 

Nomenclature	http://www.guidetopharmacology.org/GRAC/ObjectDisplayForward?objectId=875	http://www.guidetopharmacology.org/GRAC/ObjectDisplayForward?objectId=876	http://www.guidetopharmacology.org/GRAC/ObjectDisplayForward?objectId=877	http://www.guidetopharmacology.org/GRAC/ObjectDisplayForward?objectId=878	http://www.guidetopharmacology.org/GRAC/ObjectDisplayForward?objectId=879
Systematic nomenclature	SLC2A1	SLC2A2	SLC2A3	SLC2A4	SLC2A14
Common abbreviation	GLUT1	GLUT2	GLUT3	GLUT4	GLUT14
HGNC, UniProt	https://www.genenames.org/data/gene‐symbol‐report/#!/hgnc_id/HGNC:11005, http://www.uniprot.org/uniprot/P11166	https://www.genenames.org/data/gene‐symbol‐report/#!/hgnc_id/HGNC:11006, http://www.uniprot.org/uniprot/P11168	https://www.genenames.org/data/gene‐symbol‐report/#!/hgnc_id/HGNC:11007, http://www.uniprot.org/uniprot/P11169	https://www.genenames.org/data/gene‐symbol‐report/#!/hgnc_id/HGNC:11009, http://www.uniprot.org/uniprot/P14672	https://www.genenames.org/data/gene‐symbol‐report/#!/hgnc_id/HGNC:18301, http://www.uniprot.org/uniprot/Q8TDB8
Substrates	http://www.guidetopharmacology.org/GRAC/LigandDisplayForward?ligandId=4535 (D‐glucose = D‐glucosamine) [http://www.ncbi.nlm.nih.gov/pubmed/12135767?dopt=AbstractPlus], http://www.guidetopharmacology.org/GRAC/LigandDisplayForward?ligandId=4733 [http://www.ncbi.nlm.nih.gov/pubmed/3945643?dopt=AbstractPlus], http://www.guidetopharmacology.org/GRAC/LigandDisplayForward?ligandId=4536 (D‐glucose = D‐glucosamine) [http://www.ncbi.nlm.nih.gov/pubmed/12135767?dopt=AbstractPlus]	http://www.guidetopharmacology.org/GRAC/LigandDisplayForward?ligandId=4535 (D‐glucosamine > D‐glucose) [http://www.ncbi.nlm.nih.gov/pubmed/12135767?dopt=AbstractPlus], http://www.guidetopharmacology.org/GRAC/LigandDisplayForward?ligandId=4536 (D‐glucosamine > D‐glucose) [http://www.ncbi.nlm.nih.gov/pubmed/12135767?dopt=AbstractPlus]	http://www.guidetopharmacology.org/GRAC/LigandDisplayForward?ligandId=4536	http://www.guidetopharmacology.org/GRAC/LigandDisplayForward?ligandId=4535 (D‐glucosamine ≥ D‐glucose) [http://www.ncbi.nlm.nih.gov/pubmed/12135767?dopt=AbstractPlus], http://www.guidetopharmacology.org/GRAC/LigandDisplayForward?ligandId=4536 (D‐glucosamine ≥ D‐glucose) [http://www.ncbi.nlm.nih.gov/pubmed/12135767?dopt=AbstractPlus]	–
Labelled ligands	http://www.guidetopharmacology.org/GRAC/LigandDisplayForward?ligandId=4643	http://www.guidetopharmacology.org/GRAC/LigandDisplayForward?ligandId=4643	http://www.guidetopharmacology.org/GRAC/LigandDisplayForward?ligandId=4643	http://www.guidetopharmacology.org/GRAC/LigandDisplayForward?ligandId=4643	–
Comments	GLUT1 is a class I facilitative sugar transporter. GLUT1 functions to maintain basal glucose import which is required for cellular respiration.	–	–	–	–

## http://www.guidetopharmacology.org/GRAC/FamilyDisplayForward?familyId=166

### Overview

Class II transporters transport http://www.guidetopharmacology.org/GRAC/LigandDisplayForward?ligandId=4654 and appear to be insensitive to http://www.guidetopharmacology.org/GRAC/LigandDisplayForward?ligandId=5334. Class II transporters appear to be predominantly intracellularly located.

**Table:** 

Nomenclature	http://www.guidetopharmacology.org/GRAC/ObjectDisplayForward?objectId=880	http://www.guidetopharmacology.org/GRAC/ObjectDisplayForward?objectId=881	http://www.guidetopharmacology.org/GRAC/ObjectDisplayForward?objectId=882
Systematic nomenclature	SLC2A5	SLC2A7	SLC2A9
Common abbreviation	GLUT5	GLUT7	GLUT9
HGNC, UniProt	https://www.genenames.org/data/gene‐symbol‐report/#!/hgnc_id/HGNC:11010, http://www.uniprot.org/uniprot/P22732	https://www.genenames.org/data/gene‐symbol‐report/#!/hgnc_id/HGNC:13445, http://www.uniprot.org/uniprot/Q6PXP3	https://www.genenames.org/data/gene‐symbol‐report/#!/hgnc_id/HGNC:13446, http://www.uniprot.org/uniprot/Q9NRM0
Substrates	http://www.guidetopharmacology.org/GRAC/LigandDisplayForward?ligandId=4654 (D‐fructose > D‐glucose) [http://www.ncbi.nlm.nih.gov/pubmed/1634504?dopt=AbstractPlus], http://www.guidetopharmacology.org/GRAC/LigandDisplayForward?ligandId=4536 (D‐fructose > D‐glucose) [http://www.ncbi.nlm.nih.gov/pubmed/1634504?dopt=AbstractPlus]	http://www.guidetopharmacology.org/GRAC/LigandDisplayForward?ligandId=4654 [http://www.ncbi.nlm.nih.gov/pubmed/18477702?dopt=AbstractPlus], http://www.guidetopharmacology.org/GRAC/LigandDisplayForward?ligandId=4536 [http://www.ncbi.nlm.nih.gov/pubmed/18477702?dopt=AbstractPlus]	http://www.guidetopharmacology.org/GRAC/LigandDisplayForward?ligandId=4654 [http://www.ncbi.nlm.nih.gov/pubmed/18842065?dopt=AbstractPlus], http://www.guidetopharmacology.org/GRAC/LigandDisplayForward?ligandId=4731 [http://www.ncbi.nlm.nih.gov/pubmed/18842065?dopt=AbstractPlus]

**Table:** 

Nomenclature	http://www.guidetopharmacology.org/GRAC/ObjectDisplayForward?objectId=883	http://www.guidetopharmacology.org/GRAC/ObjectDisplayForward?objectId=884	http://www.guidetopharmacology.org/GRAC/ObjectDisplayForward?objectId=885	http://www.guidetopharmacology.org/GRAC/ObjectDisplayForward?objectId=886	http://www.guidetopharmacology.org/GRAC/ObjectDisplayForward?objectId=887
Systematic nomenclature	SLC2A11	SLC2A6	SLC2A8	SLC2A10	SLC2A12
Common abbreviation	GLUT11	GLUT6	GLUT8	GLUT10	GLUT12
HGNC, UniProt	https://www.genenames.org/data/gene‐symbol‐report/#!/hgnc_id/HGNC:14239, http://www.uniprot.org/uniprot/Q9BYW1	https://www.genenames.org/data/gene‐symbol‐report/#!/hgnc_id/HGNC:11011, http://www.uniprot.org/uniprot/Q9UGQ3	https://www.genenames.org/data/gene‐symbol‐report/#!/hgnc_id/HGNC:13812, http://www.uniprot.org/uniprot/Q9NY64	https://www.genenames.org/data/gene‐symbol‐report/#!/hgnc_id/HGNC:13444, http://www.uniprot.org/uniprot/O95528	https://www.genenames.org/data/gene‐symbol‐report/#!/hgnc_id/HGNC:18067, http://www.uniprot.org/uniprot/Q8TD20
Substrates	http://www.guidetopharmacology.org/GRAC/LigandDisplayForward?ligandId=4654 [http://www.ncbi.nlm.nih.gov/pubmed/17710649?dopt=AbstractPlus], http://www.guidetopharmacology.org/GRAC/LigandDisplayForward?ligandId=4536 [http://www.ncbi.nlm.nih.gov/pubmed/11583593?dopt=AbstractPlus]	–	http://www.guidetopharmacology.org/GRAC/LigandDisplayForward?ligandId=4536 [http://www.ncbi.nlm.nih.gov/pubmed/10671487?dopt=AbstractPlus]	http://www.guidetopharmacology.org/GRAC/LigandDisplayForward?ligandId=4733 [http://www.ncbi.nlm.nih.gov/pubmed/20639396?dopt=AbstractPlus], http://www.guidetopharmacology.org/GRAC/LigandDisplayForward?ligandId=4536 [http://www.ncbi.nlm.nih.gov/pubmed/20639396?dopt=AbstractPlus]	http://www.guidetopharmacology.org/GRAC/LigandDisplayForward?ligandId=4536 [http://www.ncbi.nlm.nih.gov/pubmed/12914765?dopt=AbstractPlus]

## http://www.guidetopharmacology.org/GRAC/FamilyDisplayForward?familyId=167

### Overview

Proton‐coupled inositol transporters are expressed predominantly in the brain and can be inhibited by http://www.guidetopharmacology.org/GRAC/LigandDisplayForward?ligandId=4285 and http://www.guidetopharmacology.org/GRAC/LigandDisplayForward?ligandId=5334 [http://www.ncbi.nlm.nih.gov/pubmed/12135767?dopt=AbstractPlus].

**Table:** 

Nomenclature	http://www.guidetopharmacology.org/GRAC/ObjectDisplayForward?objectId=888
Systematic nomenclature	SLC2A13
Common abbreviation	HMIT
HGNC, UniProt	https://www.genenames.org/data/gene‐symbol‐report/#!/hgnc_id/HGNC:15956, http://www.uniprot.org/uniprot/Q96QE2
Substrates	http://www.guidetopharmacology.org/GRAC/LigandDisplayForward?ligandId=4645 [http://www.ncbi.nlm.nih.gov/pubmed/12135767?dopt=AbstractPlus], http://www.guidetopharmacology.org/GRAC/LigandDisplayForward?ligandId=4495 [http://www.ncbi.nlm.nih.gov/pubmed/12135767?dopt=AbstractPlus], http://www.guidetopharmacology.org/GRAC/LigandDisplayForward?ligandId=4649 [http://www.ncbi.nlm.nih.gov/pubmed/12135767?dopt=AbstractPlus], http://www.guidetopharmacology.org/GRAC/LigandDisplayForward?ligandId=4648 [http://www.ncbi.nlm.nih.gov/pubmed/12135767?dopt=AbstractPlus]
Stoichiometry	1 H^+^: 1 inositol (in) [http://www.ncbi.nlm.nih.gov/pubmed/19607714?dopt=AbstractPlus]

### Further reading on SLC2 family of hexose and sugar alcohol transporters

Augustin R. (2010) The protein family of glucose transport facilitators: It's not only about glucose after all. *IUBMB Life* **62**: 315–33 https://www.ncbi.nlm.nih.gov/pubmed/20209635?dopt=AbstractPlus

Klip A *et al*. (2014) Signal transduction meets vesicle traffic: the software and hardware of GLUT4 translocation. *Am. J. Physiol., Cell Physiol*. **306**: C879–86 https://www.ncbi.nlm.nih.gov/pubmed/24598362?dopt=AbstractPlus

Leney SE *et al*. (2009) Themolecular basis of insulin‐stimulated glucose uptake: signalling, trafficking and potential drug targets. *J. Endocrinol*. **203**: 1–18 https://www.ncbi.nlm.nih.gov/pubmed/19389739?dopt=AbstractPlus

Mueckler M *et al*. (2013) The SLC2 (GLUT) family of membrane transporters. Mol. Aspects Med. 34: 121‐38 https://www.ncbi.nlm.nih.gov/pubmed/23506862?dopt=AbstractPlus

Uldry M *et al*. (2004) The SLC2 family of facilitated hexose and polyol transporters. *Pflugers Arch*. 447: 480‐9 https://www.ncbi.nlm.nih.gov/pubmed/12750891?dopt=AbstractPlus

## http://www.guidetopharmacology.org/GRAC/FamilyDisplayForward?familyId=141

### Overview

The SLC3 and SLC7 families combine to generate functional transporters, where the subunit composition is a disulphide‐linked combination of a heavy chain (SLC3 family) with a light chain (SLC7 family).

## http://www.guidetopharmacology.org/GRAC/FamilyDisplayForward?familyId=168

### Overview

SLC3 family members are single TM proteins with extensive glycosylation of the exterior C‐terminus, which heterodimerize with SLC7 family members in the endoplasmic reticulum and assist in the plasma membrane localization of the transporter.

Information on members of this family may be found in the http://www.guidetopharmacology.org/GRAC/FamilyDisplayForward?familyId=168.

## http://www.guidetopharmacology.org/GRAC/FamilyDisplayForward?familyId=169

### Overview

SLC7 family members may be divided into two major groups: cationic amino acid transporters (CATs) and glycoprotein‐associated amino acid transporters (gpaATs). Cationic amino acid transporters are 14 TM proteins, which mediate pH^‐^ and sodium‐independent transport of cationic amino acids (system y^+^), apparently as an exchange mechanism. These transporters are sensitive to inhibition by http://www.guidetopharmacology.org/GRAC/LigandDisplayForward?ligandId=5335.

**Table:** 

Nomenclature	http://www.guidetopharmacology.org/GRAC/ObjectDisplayForward?objectId=891	http://www.guidetopharmacology.org/GRAC/ObjectDisplayForward?objectId=892	http://www.guidetopharmacology.org/GRAC/ObjectDisplayForward?objectId=893	http://www.guidetopharmacology.org/GRAC/ObjectDisplayForward?objectId=896	http://www.guidetopharmacology.org/GRAC/ObjectDisplayForward?objectId=897
Systematic nomenclature	SLC7A1	SLC7A2	SLC7A3	SLC7A5	SLC7A8
Common abbreviation	CAT1	CAT2	CAT3	LAT1	LAT2
HGNC, UniProt	https://www.genenames.org/data/gene‐symbol‐report/#!/hgnc_id/HGNC:11057, http://www.uniprot.org/uniprot/P30825	https://www.genenames.org/data/gene‐symbol‐report/#!/hgnc_id/HGNC:11060, http://www.uniprot.org/uniprot/P52569	https://www.genenames.org/data/gene‐symbol‐report/#!/hgnc_id/HGNC:11061, http://www.uniprot.org/uniprot/Q8WY07	https://www.genenames.org/data/gene‐symbol‐report/#!/hgnc_id/HGNC:11063, http://www.uniprot.org/uniprot/Q01650	https://www.genenames.org/data/gene‐symbol‐report/#!/hgnc_id/HGNC:11066, http://www.uniprot.org/uniprot/Q9UHI5
Substrates	http://www.guidetopharmacology.org/GRAC/LigandDisplayForward?ligandId=725, http://www.guidetopharmacology.org/GRAC/LigandDisplayForward?ligandId=721, http://www.guidetopharmacology.org/GRAC/LigandDisplayForward?ligandId=724, http://www.guidetopharmacology.org/GRAC/LigandDisplayForward?ligandId=3310	http://www.guidetopharmacology.org/GRAC/LigandDisplayForward?ligandId=725, http://www.guidetopharmacology.org/GRAC/LigandDisplayForward?ligandId=721, http://www.guidetopharmacology.org/GRAC/LigandDisplayForward?ligandId=724, http://www.guidetopharmacology.org/GRAC/LigandDisplayForward?ligandId=3310	http://www.guidetopharmacology.org/GRAC/LigandDisplayForward?ligandId=725, http://www.guidetopharmacology.org/GRAC/LigandDisplayForward?ligandId=721, http://www.guidetopharmacology.org/GRAC/LigandDisplayForward?ligandId=724	–	–
Selective inhibitors	–	–	–	http://www.guidetopharmacology.org/GRAC/LigandDisplayForward?ligandId=9347 [http://www.ncbi.nlm.nih.gov/pubmed/19900191?dopt=AbstractPlus]	–

**Table:** 

Nomenclature	http://www.guidetopharmacology.org/GRAC/ObjectDisplayForward?objectId=898	http://www.guidetopharmacology.org/GRAC/ObjectDisplayForward?objectId=899	http://www.guidetopharmacology.org/GRAC/ObjectDisplayForward?objectId=900	http://www.guidetopharmacology.org/GRAC/ObjectDisplayForward?objectId=901	http://www.guidetopharmacology.org/GRAC/ObjectDisplayForward?objectId=902	http://www.guidetopharmacology.org/GRAC/ObjectDisplayForward?objectId=903
Systematic nomenclature	SLC7A7	SLC7A6	SLC7A9	SLC7A10	SLC7A11	SLC7A13
Common abbreviation	y+LAT1	y+LAT2	b^0,+^AT	Asc‐1	xCT	–
HGNC, UniProt	https://www.genenames.org/data/gene‐symbol‐report/#!/hgnc_id/HGNC:11065, http://www.uniprot.org/uniprot/Q9UM01	https://www.genenames.org/data/gene‐symbol‐report/#!/hgnc_id/HGNC:11064, http://www.uniprot.org/uniprot/Q92536	https://www.genenames.org/data/gene‐symbol‐report/#!/hgnc_id/HGNC:11067, http://www.uniprot.org/uniprot/P82251	https://www.genenames.org/data/gene‐symbol‐report/#!/hgnc_id/HGNC:11058, http://www.uniprot.org/uniprot/Q9NS82	https://www.genenames.org/data/gene‐symbol‐report/#!/hgnc_id/HGNC:11059, http://www.uniprot.org/uniprot/Q9UPY5	https://www.genenames.org/data/gene‐symbol‐report/#!/hgnc_id/HGNC:23092, http://www.uniprot.org/uniprot/Q8TCU3
Inhibitors	–	–	–	–	http://www.guidetopharmacology.org/GRAC/LigandDisplayForward?ligandId=1372 (pIC50 5.3) [http://www.ncbi.nlm.nih.gov/pubmed/20303751?dopt=AbstractPlus]	–

### Comments

CAT4 appears to be non‐functional in heterologous expression [http://www.ncbi.nlm.nih.gov/pubmed/12049641?dopt=AbstractPlus], while SLC7A14 has yet to be characterized. Glycoprotein‐associated amino acid transporters are 12 TM proteins, which heterodimerize with members of the SLC3 family to act as cell‐surface amino acid exchangers.

Heterodimers between 4F2hc and LAT1 or LAT2 generate sodium‐independent system L transporters. LAT1 transports large neutral amino acids including branched‐chain and aromatic amino acids as well as http://www.guidetopharmacology.org/GRAC/LigandDisplayForward?ligandId=4841, whereas LAT2 transports most of the neutral amino acids.

Heterodimers between 4F2hc and y^+^LAT1 or y^+^LAT2 generate transporters similar to the system y^+^L , which transport cationic (http://www.guidetopharmacology.org/GRAC/LigandDisplayForward?ligandId=721, http://www.guidetopharmacology.org/GRAC/LigandDisplayForward?ligandId=724, http://www.guidetopharmacology.org/GRAC/LigandDisplayForward?ligandId=725) amino acids independent of sodium and neutral (http://www.guidetopharmacology.org/GRAC/LigandDisplayForward?ligandId=3312, http://www.guidetopharmacology.org/GRAC/LigandDisplayForward?ligandId=3311, http://www.guidetopharmacology.org/GRAC/LigandDisplayForward?ligandId=4814, http://www.guidetopharmacology.org/GRAC/LigandDisplayForward?ligandId=723) amino acids in a partially sodium‐dependent manner. These transporters are http://www.guidetopharmacology.org/GRAC/LigandDisplayForward?ligandId=5335‐insensitive. Heterodimers between rBAT and b^0,+^AT appear to mediate sodium‐independent system b^0,+^ transport of most of the neutral amino acids and cationic amino acids (http://www.guidetopharmacology.org/GRAC/LigandDisplayForward?ligandId=721, http://www.guidetopharmacology.org/GRAC/LigandDisplayForward?ligandId=724 and http://www.guidetopharmacology.org/GRAC/LigandDisplayForward?ligandId=725).

Asc‐1 appears to heterodimerize with 4F2hc to allow the transport of small neutral amino acids (such as http://www.guidetopharmacology.org/GRAC/LigandDisplayForward?ligandId=720, http://www.guidetopharmacology.org/GRAC/LigandDisplayForward?ligandId=726, http://www.guidetopharmacology.org/GRAC/LigandDisplayForward?ligandId=4785, http://www.guidetopharmacology.org/GRAC/LigandDisplayForward?ligandId=723 and http://www.guidetopharmacology.org/GRAC/LigandDisplayForward?ligandId=727), as well as http://www.guidetopharmacology.org/GRAC/LigandDisplayForward?ligandId=4171, in a sodium‐independent manner.

xCT generates a heterodimer with 4F2hc for a system x^‐^_e‐c_ transporter that mediates the sodium‐independent exchange of http://www.guidetopharmacology.org/GRAC/LigandDisplayForward?ligandId=5413 and http://www.guidetopharmacology.org/GRAC/LigandDisplayForward?ligandId=1369.

AGT has been conjugated with SLC3 members as fusion proteins to generate functional transporters, but the identity of a native heterodimer has yet to be ascertained.

### Further reading on SLC3 and SLC7 families of heteromeric amino acid transporters (HATs)

Bhutia YD *et al*. (2015) Amino Acid transporters in cancer and their relevance to "glutamine addiction": novel targets for the design of a new class of anticancer drugs. *Cancer Res*. **75**: 1782–8 https://www.ncbi.nlm.nih.gov/pubmed/25855379?dopt=AbstractPlus

Fotiadis D *et al*. (2013) The SLC3 and SLC7 families of amino acid transporters. *Mol. Aspects Med*. **34**: 139–58 https://www.ncbi.nlm.nih.gov/pubmed/23506863?dopt=AbstractPlus

Palacín M *et al*. (2004) The ancillary proteins of HATs: SLC3 family of amino acid transporters. *Pflugers Arch*. **447**: 490–4 https://www.ncbi.nlm.nih.gov/pubmed/14770309?dopt=AbstractPlus

Palacín M *et al*. (2005) The genetics of heteromeric amino acid transporters. *Physiology (Bethesda)* **20**: 112–24 https://www.ncbi.nlm.nih.gov/pubmed/15772300?dopt=AbstractPlus

Verrey F *et al*. (2004) CATs and HATs: the SLC7 family of amino acid transporters. *Pflugers Arch*. **447**: 532–42 https://www.ncbi.nlm.nih.gov/pubmed/14770310?dopt=AbstractPlus

## http://www.guidetopharmacology.org/GRAC/FamilyDisplayForward?familyId=142

### Overview

Together with the SLC26 family, the SLC4 family of transporters subserve anion exchange, principally of chloride and bicarbonate (HCO_3_^‐^), but also carbonate and hydrogen sulphate (HSO_4_^‐^). SLC4 family members regulate bicarbonate fluxes as part of carbon dioxidemovement, chyme neutralization and reabsorption in the kidney.

Within the family, subgroups of transporters are identifiable: the electroneutral sodium‐independent Cl^‐^/HCO_3_^‐^ transporters (AE1, AE2 and AE3), the electrogenic sodium‐dependent HCO_3_^‐^ transporters (NBCe1 and NBCe2) and the electroneutral HCO_3_^‐^ transporters (NBCn1 and NBCn2). Topographical information derives mainly from study of AE1, abundant in erythrocytes, which suggests a dimeric or tetrameric arrangement, with subunits made up of 13 TM domains and re‐entrant loops at TM9/10 and TM11/12. The N terminus exhibits sites for interaction with multiple proteins, including glycolytic enzymes, haemoglobin and cytoskeletal elements.

## http://www.guidetopharmacology.org/GRAC/FamilyDisplayForward?familyId=170

**Table:** 

Nomenclature	http://www.guidetopharmacology.org/GRAC/ObjectDisplayForward?objectId=904	http://www.guidetopharmacology.org/GRAC/ObjectDisplayForward?objectId=905	http://www.guidetopharmacology.org/GRAC/ObjectDisplayForward?objectId=906	http://www.guidetopharmacology.org/GRAC/ObjectDisplayForward?objectId=907
Systematic nomenclature	SLC4A1	SLC4A2	SLC4A3	SLC4A9
Common abbreviation	AE1	AE2	AE3	AE4
HGNC, UniProt	https://www.genenames.org/data/gene‐symbol‐report/#!/hgnc_id/HGNC:11027, http://www.uniprot.org/uniprot/P02730	https://www.genenames.org/data/gene‐symbol‐report/#!/hgnc_id/HGNC:11028, http://www.uniprot.org/uniprot/P04920	https://www.genenames.org/data/gene‐symbol‐report/#!/hgnc_id/HGNC:11029, http://www.uniprot.org/uniprot/P48751	https://www.genenames.org/data/gene‐symbol‐report/#!/hgnc_id/HGNC:11035, http://www.uniprot.org/uniprot/Q96Q91
Endogenous substrates	HCO_3_^‐^, http://www.guidetopharmacology.org/GRAC/LigandDisplayForward?ligandId=2339	http://www.guidetopharmacology.org/GRAC/LigandDisplayForward?ligandId=2339, HCO_3_^‐^	http://www.guidetopharmacology.org/GRAC/LigandDisplayForward?ligandId=2339, HCO_3_^‐^	–
Stoichiometry	1 Cl^‐^ (in) : 1 HCO_3_^‐^ (out)	1 Cl^‐^ (in) : 1 HCO_3_^‐^ (out)	1 Cl^‐^ (in) : 1 HCO_3_^‐^ (out)	–

## http://www.guidetopharmacology.org/GRAC/FamilyDisplayForward?familyId=171

**Table:** 

Nomenclature	http://www.guidetopharmacology.org/GRAC/ObjectDisplayForward?objectId=908	http://www.guidetopharmacology.org/GRAC/ObjectDisplayForward?objectId=909	http://www.guidetopharmacology.org/GRAC/ObjectDisplayForward?objectId=910	http://www.guidetopharmacology.org/GRAC/ObjectDisplayForward?objectId=911	http://www.guidetopharmacology.org/GRAC/ObjectDisplayForward?objectId=912	http://www.guidetopharmacology.org/GRAC/ObjectDisplayForward?objectId=913
Systematic nomenclature	SLC4A4	SLC4A5	SLC4A7	SLC4A10	SLC4A8	SLC4A11
Common abbreviation	NBCe1	NBCe2	NBCn1	NBCn2	NDCBE	BTR1
HGNC, UniProt	https://www.genenames.org/data/gene‐symbol‐report/#!/hgnc_id/HGNC:11030, http://www.uniprot.org/uniprot/Q9Y6R1	https://www.genenames.org/data/gene‐symbol‐report/#!/hgnc_id/HGNC:18168, http://www.uniprot.org/uniprot/Q9BY07	https://www.genenames.org/data/gene‐symbol‐report/#!/hgnc_id/HGNC:11033, http://www.uniprot.org/uniprot/Q9Y6M7	https://www.genenames.org/data/gene‐symbol‐report/#!/hgnc_id/HGNC:13811, http://www.uniprot.org/uniprot/Q6U841	https://www.genenames.org/data/gene‐symbol‐report/#!/hgnc_id/HGNC:11034, http://www.uniprot.org/uniprot/Q2Y0W8	https://www.genenames.org/data/gene‐symbol‐report/#!/hgnc_id/HGNC:16438, http://www.uniprot.org/uniprot/Q8NBS3
Endogenous substrates	http://www.guidetopharmacology.org/GRAC/LigandDisplayForward?ligandId=4507	http://www.guidetopharmacology.org/GRAC/LigandDisplayForward?ligandId=4507	http://www.guidetopharmacology.org/GRAC/LigandDisplayForward?ligandId=4507	http://www.guidetopharmacology.org/GRAC/LigandDisplayForward?ligandId=4507	http://www.guidetopharmacology.org/GRAC/LigandDisplayForward?ligandId=4507, http://www.guidetopharmacology.org/GRAC/LigandDisplayForward?ligandId=2339	http://www.guidetopharmacology.org/GRAC/LigandDisplayForward?ligandId=2339, http://www.guidetopharmacology.org/GRAC/LigandDisplayForward?ligandId=4507
Stoichiometry	1 Na^+^ : 2/3 HCO_3_^‐^ (out) or 1 Na^+^ : CO_3_^2*^	1 Na^+^ : 2/3 HCO_3_^‐^ (out) or 1 Na^+^ : CO_3_^2*^	1 Na^+^ : 1 HCO_3_^‐^ (out) or 1 Na^+^ : CO_3_^2*^	1 Na^+^ : 1 HCO_3_^‐^ (out) or 1 Na : CO_3_^2*^	1 Na^+^ : 2HCO_3_^‐^ (in) : 1 Cl^‐^ (out)	–

### Further reading on SLC4 family of bicarbonate transporters

Majumdar D *et al*. (2010) Na‐coupled bicarbonate transporters of the solute carrier 4 family in the nervous system: function, localization, and relevance to neurologic function. *Neuroscience* **171**: 951‐72 [https://www.ncbi.nlm.nih.gov/pubmed/20884330?dopt=AbstractPlus]

Parker MD *et al*. (2013) The divergence, actions, roles, and relatives of sodium‐coupled bicarbonate transporters. *Physiol. Rev*. **93**: 803‐959 [https://www.ncbi.nlm.nih.gov/pubmed/23589833?dopt=AbstractPlus]

Reithmeier RA *et al*. (2016) Band 3, the human red cell chloride/bicarbonate anion exchanger (AE1, SLC4A1), in a structural context. *Biochim. Biophys. Acta* **1858**: 1507‐32 [https://www.ncbi.nlm.nih.gov/pubmed/27058983?dopt=AbstractPlus]

Romero MF *et al*. (2013) The SLC4 family of bicarbonate (HCO_3^‐^) transporters. *Mol. Aspects Med*. **34**: 159‐82 [https://www.ncbi.nlm.nih.gov/pubmed/23506864?dopt=AbstractPlus]

Thornell IM *et al*. (2015) Regulators of Slc4 bicarbonate transporter activity. *Front Physiol* **6**: 166 [https://www.ncbi.nlm.nih.gov/pubmed/26124722?dopt=AbstractPlus]

## http://www.guidetopharmacology.org/GRAC/FamilyDisplayForward?familyId=143

### Overview

The SLC5 family of sodium‐dependent glucose transporters includes, in mammals, the Na^+^/substrate co‐transporters for glucose (*e.g*. http://www.guidetopharmacology.org/GRAC/LigandDisplayForward?ligandId=4551), http://www.guidetopharmacology.org/GRAC/LigandDisplayForward?ligandId=4536, monocarboxylates, http://www.guidetopharmacology.org/GRAC/LigandDisplayForward?ligandId=4495 and I^‐^ [http://www.ncbi.nlm.nih.gov/pubmed/14993474?dopt=AbstractPlus, http://www.ncbi.nlm.nih.gov/pubmed/18446519?dopt=AbstractPlus, http://www.ncbi.nlm.nih.gov/pubmed/21527736?dopt=AbstractPlus, http://www.ncbi.nlm.nih.gov/pubmed/12748858?dopt=AbstractPlus]. Members of the SLC5 and SLC6 families, along with other unrelated Na^+^ cotransporters (*i.e*. Mhp1 and BetP), share a common structural core that contains an inverted repeat of 5TM α‐helical domains [http://www.ncbi.nlm.nih.gov/pubmed/19631523?dopt=AbstractPlus].

## http://www.guidetopharmacology.org/GRAC/FamilyDisplayForward?familyId=173

### Overview

Detailed characterisation of members of the hexose transporter family is limited to SGLT1, 2 and 3, which are all inhibited in a competitive manner by http://www.guidetopharmacology.org/GRAC/LigandDisplayForward?ligandId=4757, a natural dihydrocholine glucoside, that exhibits modest selectivity towards SGLT2 (see [http://www.ncbi.nlm.nih.gov/pubmed/21527736?dopt=AbstractPlus] for an extensive review). SGLT1 is predominantly expressed in the small intestine, mediating the absorption of glucose (*e.g*. http://www.guidetopharmacology.org/GRAC/LigandDisplayForward?ligandId=4536), but also occurs in the brain, heart and in the late proximal straight tubule of the kidney. The expression of SGLT2 is almost exclusively restricted to the early proximal convoluted tubule of the kidney, where it is largely responsible for the renal reabsorption of glucose. SGLT3 is not a transporter but instead acts as a glucosensor generating an inwardly directed flux of Na^+^ that causes membrane depolarization [http://www.ncbi.nlm.nih.gov/pubmed/13130073?dopt=AbstractPlus].

**Table:** 

Nomenclature	http://www.guidetopharmacology.org/GRAC/ObjectDisplayForward?objectId=915	http://www.guidetopharmacology.org/GRAC/ObjectDisplayForward?objectId=916	http://www.guidetopharmacology.org/GRAC/ObjectDisplayForward?objectId=917	http://www.guidetopharmacology.org/GRAC/ObjectDisplayForward?objectId=918	http://www.guidetopharmacology.org/GRAC/ObjectDisplayForward?objectId=919
Systematic nomenclature	SLC5A1	SLC5A2	SLC5A4	SLC5A9	SLC5A10
Common abbreviation	SGLT1	SGLT2	SGLT3	SGLT4	SGLT5
HGNC, UniProt	https://www.genenames.org/data/gene‐symbol‐report/#!/hgnc_id/HGNC:11036, http://www.uniprot.org/uniprot/P13866	https://www.genenames.org/data/gene‐symbol‐report/#!/hgnc_id/HGNC:11037, http://www.uniprot.org/uniprot/P31639	https://www.genenames.org/data/gene‐symbol‐report/#!/hgnc_id/HGNC:11039, http://www.uniprot.org/uniprot/Q9NY91	https://www.genenames.org/data/gene‐symbol‐report/#!/hgnc_id/HGNC:22146, http://www.uniprot.org/uniprot/Q2M3M2	https://www.genenames.org/data/gene‐symbol‐report/#!/hgnc_id/HGNC:23155, http://www.uniprot.org/uniprot/A0PJK1
Substrates	http://www.guidetopharmacology.org/GRAC/LigandDisplayForward?ligandId=4646 [http://www.ncbi.nlm.nih.gov/pubmed/17110502?dopt=AbstractPlus], http://www.guidetopharmacology.org/GRAC/LigandDisplayForward?ligandId=4640 [http://www.ncbi.nlm.nih.gov/pubmed/17110502?dopt=AbstractPlus], http://www.guidetopharmacology.org/GRAC/LigandDisplayForward?ligandId=4536 [http://www.ncbi.nlm.nih.gov/pubmed/17110502?dopt=AbstractPlus]	http://www.guidetopharmacology.org/GRAC/LigandDisplayForward?ligandId=4640, http://www.guidetopharmacology.org/GRAC/LigandDisplayForward?ligandId=4536	http://www.guidetopharmacology.org/GRAC/LigandDisplayForward?ligandId=4536 [http://www.ncbi.nlm.nih.gov/pubmed/17110502?dopt=AbstractPlus], http://www.guidetopharmacology.org/GRAC/LigandDisplayForward?ligandId=4744 [http://www.ncbi.nlm.nih.gov/pubmed/17110502?dopt=AbstractPlus], http://www.guidetopharmacology.org/GRAC/LigandDisplayForward?ligandId=4586 [http://www.ncbi.nlm.nih.gov/pubmed/17110502?dopt=AbstractPlus], http://www.guidetopharmacology.org/GRAC/LigandDisplayForward?ligandId=4841 [http://www.ncbi.nlm.nih.gov/pubmed/17110502?dopt=AbstractPlus], http://www.guidetopharmacology.org/GRAC/LigandDisplayForward?ligandId=4842 [http://www.ncbi.nlm.nih.gov/pubmed/17110502?dopt=AbstractPlus], http://www.guidetopharmacology.org/GRAC/LigandDisplayForward?ligandId=4642 [http://www.ncbi.nlm.nih.gov/pubmed/17110502?dopt=AbstractPlus]	http://www.guidetopharmacology.org/GRAC/LigandDisplayForward?ligandId=4536, http://www.guidetopharmacology.org/GRAC/LigandDisplayForward?ligandId=4650, http://www.guidetopharmacology.org/GRAC/LigandDisplayForward?ligandId=4640	http://www.guidetopharmacology.org/GRAC/LigandDisplayForward?ligandId=4646, http://www.guidetopharmacology.org/GRAC/LigandDisplayForward?ligandId=4536
Stoichiometry	2 Na^+^ : 1 glucose [http://www.ncbi.nlm.nih.gov/pubmed/8282810?dopt=AbstractPlus]	1 Na^+^ : 1 glucose [http://www.ncbi.nlm.nih.gov/pubmed/20980548?dopt=AbstractPlus]	–	–	–
Selective inhibitors	http://www.guidetopharmacology.org/GRAC/LigandDisplayForward?ligandId=9547 (p*K*_i_ 7.6) [http://www.ncbi.nlm.nih.gov/pubmed/28410751?dopt=AbstractPlus]	http://www.guidetopharmacology.org/GRAC/LigandDisplayForward?ligandId=4594 (pIC_50_ 9.3) [http://www.ncbi.nlm.nih.gov/pubmed/20637636?dopt=AbstractPlus]	–	–	–
Comments	–	–	SGLT3 acts as a glucosensor.	–	–

### Comments

Recognition and transport of substrate by SGLTs requires that the sugar is a pyranose. De‐oxyglucose derivatives have reduced affinity for SGLT1, but the replacement of the sugar equatorial hydroxyl group by fluorine at some positions, excepting C2 and C3, is tolerated (see [http://www.ncbi.nlm.nih.gov/pubmed/21527736?dopt=AbstractPlus] for a detailed quantification). Although SGLT1 and SGLT2 have been described as high‐ and lowaffinity sodium glucose co‐transporters, respectively, recent work suggests that they have a similar affinity for glucose under physiological conditions [http://www.ncbi.nlm.nih.gov/pubmed/20980548?dopt=AbstractPlus]. Selective blockers of SGLT2, and thus blocking 50% of renal glucose reabsorption, are in development for the treatment of diabetes (*e.g*. [http://www.ncbi.nlm.nih.gov/pubmed/20508640?dopt=AbstractPlus]).

## http://www.guidetopharmacology.org/GRAC/FamilyDisplayForward?familyId=172

### Overview

The high affinity, hemicholinium‐3‐sensitive, choline transporter (CHT) is expressed mainly in cholinergic neurones on nerve cell terminals and synaptic vesicles (keratinocytes being an additional location). In autonomic neurones, expression of CHT requires an activity‐dependent retrograde signal from postsynaptic neurones [http://www.ncbi.nlm.nih.gov/pubmed/19186169?dopt=AbstractPlus]. Through recapture of http://www.guidetopharmacology.org/GRAC/LigandDisplayForward?ligandId=4551 generated by the hydrolysis of ACh by http://www.guidetopharmacology.org/GRAC/LigandDisplayForward?ligandId=294sterase, CHT serves to maintain acetylcholine synthesis within the presynaptic terminal [http://www.ncbi.nlm.nih.gov/pubmed/14993474?dopt=AbstractPlus]. Homozygous mice engineered to lack CHT die within one hour of birth as a result of hypoxia arising from failure of transmission at the neuromuscular junction of the skeletal muscles that support respiration [http://www.ncbi.nlm.nih.gov/pubmed/15173594?dopt=AbstractPlus]. A low affinity choline uptake mechanism that remains to be identified at the molecular level may involve multiple transporters. In addition, a family of choline transporter‐like (CTL) proteins, (which are members of the SLC44 family) with weak Na^+^ dependence have been described [http://www.ncbi.nlm.nih.gov/pubmed/15715662?dopt=AbstractPlus].

**Table:** 

Nomenclature	http://www.guidetopharmacology.org/GRAC/ObjectDisplayForward?objectId=914
Systematic nomenclature	SLC5A7
HGNC, UniProt	https://www.genenames.org/data/gene‐symbol‐report/#!/hgnc_id/HGNC:14025, http://www.uniprot.org/uniprot/Q9GZV3
Substrates	http://www.guidetopharmacology.org/GRAC/LigandDisplayForward?ligandId=4760
Endogenous substrates	http://www.guidetopharmacology.org/GRAC/LigandDisplayForward?ligandId=4551
Stoichiometry	Na^+^ : choline (variable stoichimetry); modulated by extracellular Cl^‐^ [http://www.ncbi.nlm.nih.gov/pubmed/17005849?dopt=AbstractPlus]
Selective inhibitors	http://www.guidetopharmacology.org/GRAC/LigandDisplayForward?ligandId=4494 (p*K*_i_ 7–8) [http://www.ncbi.nlm.nih.gov/pubmed/12675135?dopt=AbstractPlus]
Labelled ligands	http://www.guidetopharmacology.org/GRAC/LigandDisplayForward?ligandId=4493 (p*K*_d_ 8.2–8.4)

### Comments

K_i_ and K_D_ values for http://www.guidetopharmacology.org/GRAC/LigandDisplayForward?ligandId=4494 listed in the table are for human CHT expressed in *Xenopus laevis* oocytes [http://www.ncbi.nlm.nih.gov/pubmed/11068039?dopt=AbstractPlus], or COS‐7 cells [http://www.ncbi.nlm.nih.gov/pubmed/11027560?dopt=AbstractPlus]. http://www.guidetopharmacology.org/GRAC/LigandDisplayForward?ligandId=5502 is a substrate for CHT that causes covalent modification and irreversible inactivation of the transporter. Several exogenous substances (*e.g*. http://www.guidetopharmacology.org/GRAC/LigandDisplayForward?ligandId=4760) that are substrates for CHT act as precursors to cholinergic false transmitters.

## http://www.guidetopharmacology.org/GRAC/FamilyDisplayForward?familyId=174

### Overview

The sodium‐iodide symporter (NIS) is an iodide transporter found principally in the thyroid gland where it mediates the accumulation of I^‐^ within thyrocytes. Transport of I^‐^ by NIS from the blood across the basolateral membrane followed by apical efflux into the colloidal lumen, mediated at least in part by pendrin (SLC22A4), and most likely not SMCT1 (SLC5A8) as once thought, provides the I^‐^ required for the synthesis of the thyroid hormones triiodothyronine (http://www.guidetopharmacology.org/GRAC/LigandDisplayForward?ligandId=2634) and thyroxine (http://www.guidetopharmacology.org/GRAC/LigandDisplayForward?ligandId=2635) [http://www.ncbi.nlm.nih.gov/pubmed/19196800?dopt=AbstractPlus]. NIS is also expressed in the salivary glands, gastric mucosa, intestinal enterocytes and lactating breast. NIS mediates I^‐^ absorption in the intestine and I^‐^ secretion into the milk. SMVT is expressed on the apical membrane of intestinal enterocytes and colonocytes and is the main system responsible for http://www.guidetopharmacology.org/GRAC/LigandDisplayForward?ligandId=4787 (vitamin H) and http://www.guidetopharmacology.org/GRAC/LigandDisplayForward?ligandId=4668 (vitamin B_5_) uptake in humans [http://www.ncbi.nlm.nih.gov/pubmed/19056639?dopt=AbstractPlus]. SMVT located in kidney proximal tubule epithelial cells mediates the reabsorption of http://www.guidetopharmacology.org/GRAC/LigandDisplayForward?ligandId=4787 and http://www.guidetopharmacology.org/GRAC/LigandDisplayForward?ligandId=4668. SMCT1 (SLC5A8), which transports a wide range of monocarboxylates, is expressed in the apical membrane of epithelia of the small intestine, colon, kidney, brain neurones and the retinal pigment epithelium [http://www.ncbi.nlm.nih.gov/pubmed/18446519?dopt=AbstractPlus]. SMCT2 (SLC5A12) also localises to the apical membrane of kidney, intestine, and colon, but in the brain and retina is restricted to astrocytes and Müller cells, respectively [http://www.ncbi.nlm.nih.gov/pubmed/18446519?dopt=AbstractPlus]. SMCT1 is a high‐affinity transporter whereas SMCT2 is a lowaffinity transporter. The physiological substrates for SMCT1 and SMCT2 are lactate (http://www.guidetopharmacology.org/GRAC/LigandDisplayForward?ligandId=2932 and http://www.guidetopharmacology.org/GRAC/LigandDisplayForward?ligandId=2934), http://www.guidetopharmacology.org/GRAC/LigandDisplayForward?ligandId=4809, http://www.guidetopharmacology.org/GRAC/LigandDisplayForward?ligandId=1062, and http://www.guidetopharmacology.org/GRAC/LigandDisplayForward?ligandId=1588 in non‐colonic tissues such as the kidney. SMCT1 is also likely to be the principal transporter for the absorption of http://www.guidetopharmacology.org/GRAC/LigandDisplayForward?ligandId=1588 (vitamin B3) in the intestine and kidney [http://www.ncbi.nlm.nih.gov/pubmed/15651982?dopt=AbstractPlus]. In the small intestine and colon, the physiological substrates for these transporters are http://www.guidetopharmacology.org/GRAC/LigandDisplayForward?ligandId=1588 and the shortchain fatty acids http://www.guidetopharmacology.org/GRAC/LigandDisplayForward?ligandId=1058, http://www.guidetopharmacology.org/GRAC/LigandDisplayForward?ligandId=1062, and http://www.guidetopharmacology.org/GRAC/LigandDisplayForward?ligandId=1059 that are produced by bacterial fermentation of dietary fiber [http://www.ncbi.nlm.nih.gov/pubmed/14966140?dopt=AbstractPlus]. In the kidney, SMCT2 is responsible for the bulk absorption of lactate because of its low‐affinity/high‐capacity nature. Absence of both transporters in the kidney leads to massive excretion of lactate in urine and consequently drastic decrease in the circulating levels of lactate in blood [http://www.ncbi.nlm.nih.gov/pubmed/16873376?dopt=AbstractPlus]. SMCT1 also functions as a tumour suppressor in the colon as well as in various other non‐colonic tissues [http://www.ncbi.nlm.nih.gov/pubmed/18992769?dopt=AbstractPlus]. The tumour‐suppressive function of SMCT1 is based on its ability to transport http://www.guidetopharmacology.org/GRAC/LigandDisplayForward?ligandId=4809, an inhibitor of histone deacetylases, into cells in non‐colonic tissues [http://www.ncbi.nlm.nih.gov/pubmed/17178845?dopt=AbstractPlus]; in the colon, the ability of SMCT1 to transport http://www.guidetopharmacology.org/GRAC/LigandDisplayForward?ligandId=1059 and http://www.guidetopharmacology.org/GRAC/LigandDisplayForward?ligandId=1062, also inhibitors of histone deacetylases, underlies the tumour‐suppressive function of this transporter [http://www.ncbi.nlm.nih.gov/pubmed/18446519?dopt=AbstractPlus, http://www.ncbi.nlm.nih.gov/pubmed/18992769?dopt=AbstractPlus, http://www.ncbi.nlm.nih.gov/pubmed/16375929?dopt=AbstractPlus]. The ability of SMCT1 to promote histone acetylase inhibition through accumulation of http://www.guidetopharmacology.org/GRAC/LigandDisplayForward?ligandId=1059 and http://www.guidetopharmacology.org/GRAC/LigandDisplayForward?ligandId=1062 in immune cells is also responsible for suppression of dendritic cell development in the colon [http://www.ncbi.nlm.nih.gov/pubmed/20601425?dopt=AbstractPlus].

**Table:** 

Nomenclature	http://www.guidetopharmacology.org/GRAC/ObjectDisplayForward?objectId=920	http://www.guidetopharmacology.org/GRAC/ObjectDisplayForward?objectId=921	http://www.guidetopharmacology.org/GRAC/ObjectDisplayForward?objectId=922	http://www.guidetopharmacology.org/GRAC/ObjectDisplayForward?objectId=923
Systematic nomenclature	SLC5A5	SLC5A6	SLC5A8	SLC5A12
HGNC, UniProt	https://www.genenames.org/data/gene‐symbol‐report/#!/hgnc_id/HGNC:11040, http://www.uniprot.org/uniprot/Q92911	https://www.genenames.org/data/gene‐symbol‐report/#!/hgnc_id/HGNC:11041, http://www.uniprot.org/uniprot/Q9Y289	https://www.genenames.org/data/gene‐symbol‐report/#!/hgnc_id/HGNC:19119, http://www.uniprot.org/uniprot/Q8N695	https://www.genenames.org/data/gene‐symbol‐report/#!/hgnc_id/HGNC:28750, http://www.uniprot.org/uniprot/Q1EHB4
Substrates	http://www.guidetopharmacology.org/GRAC/LigandDisplayForward?ligandId=4524, http://www.guidetopharmacology.org/GRAC/LigandDisplayForward?ligandId=4529, I^‐^, NO_3_^‐^, http://www.guidetopharmacology.org/GRAC/LigandDisplayForward?ligandId=4515	http://www.guidetopharmacology.org/GRAC/LigandDisplayForward?ligandId=4822 [http://www.ncbi.nlm.nih.gov/pubmed/20980265?dopt=AbstractPlus], http://www.guidetopharmacology.org/GRAC/LigandDisplayForward?ligandId=4668 [http://www.ncbi.nlm.nih.gov/pubmed/20980265?dopt=AbstractPlus], I^‐^ [http://www.ncbi.nlm.nih.gov/pubmed/20980265?dopt=AbstractPlus], http://www.guidetopharmacology.org/GRAC/LigandDisplayForward?ligandId=4787 [http://www.ncbi.nlm.nih.gov/pubmed/20980265?dopt=AbstractPlus]	http://www.guidetopharmacology.org/GRAC/LigandDisplayForward?ligandId=1062, http://www.guidetopharmacology.org/GRAC/LigandDisplayForward?ligandId=4517, http://www.guidetopharmacology.org/GRAC/LigandDisplayForward?ligandId=4703, http://www.guidetopharmacology.org/GRAC/LigandDisplayForward?ligandId=1588, http://www.guidetopharmacology.org/GRAC/LigandDisplayForward?ligandId=2934, http://www.guidetopharmacology.org/GRAC/LigandDisplayForward?ligandId=1593, http://www.guidetopharmacology.org/GRAC/LigandDisplayForward?ligandId=2932, http://www.guidetopharmacology.org/GRAC/LigandDisplayForward?ligandId=4306, http://www.guidetopharmacology.org/GRAC/LigandDisplayForward?ligandId=4518, http://www.guidetopharmacology.org/GRAC/LigandDisplayForward?ligandId=1059, http://www.guidetopharmacology.org/GRAC/LigandDisplayForward?ligandId=4656, http://www.guidetopharmacology.org/GRAC/LigandDisplayForward?ligandId=4809, http://www.guidetopharmacology.org/GRAC/LigandDisplayForward?ligandId=4522, http://www.guidetopharmacology.org/GRAC/LigandDisplayForward?ligandId=4565, http://www.guidetopharmacology.org/GRAC/LigandDisplayForward?ligandId=4711, http://www.guidetopharmacology.org/GRAC/LigandDisplayForward?ligandId=4521, http://www.guidetopharmacology.org/GRAC/LigandDisplayForward?ligandId=1058, http://www.guidetopharmacology.org/GRAC/LigandDisplayForward?ligandId=4699, http://www.guidetopharmacology.org/GRAC/LigandDisplayForward?ligandId=4655	http://www.guidetopharmacology.org/GRAC/LigandDisplayForward?ligandId=4809, http://www.guidetopharmacology.org/GRAC/LigandDisplayForward?ligandId=2932, http://www.guidetopharmacology.org/GRAC/LigandDisplayForward?ligandId=1588
Stoichiometry	2Na^+^ : 1I^‐^ [http://www.ncbi.nlm.nih.gov/pubmed/9341168?dopt=AbstractPlus]; 1Na^+^ : 1 ClO_4_^‐^ [http://www.ncbi.nlm.nih.gov/pubmed/18077370?dopt=AbstractPlus]	2Na^+^ : 1 biotin (or pantothenic acid) [http://www.ncbi.nlm.nih.gov/pubmed/10772912?dopt=AbstractPlus]	2Na^+^ : 1 monocarboxylate [http://www.ncbi.nlm.nih.gov/pubmed/17526579?dopt=AbstractPlus]	–
Inhibitors	–	–	http://www.guidetopharmacology.org/GRAC/LigandDisplayForward?ligandId=4820 (pIC_50_ 4.6) [http://www.ncbi.nlm.nih.gov/pubmed/16729224?dopt=AbstractPlus], http://www.guidetopharmacology.org/GRAC/LigandDisplayForward?ligandId=2713 (pIC_50_ 4.2) [http://www.ncbi.nlm.nih.gov/pubmed/16729224?dopt=AbstractPlus], http://www.guidetopharmacology.org/GRAC/LigandDisplayForward?ligandId=4795 (pIC_50_ 3.9) [http://www.ncbi.nlm.nih.gov/pubmed/16729224?dopt=AbstractPlus]	–

### Comments

I^‐^, http://www.guidetopharmacology.org/GRAC/LigandDisplayForward?ligandId=4524, thiocyanate and NO_3_^‐^ are competitive substrate inhibitors of NIS [http://www.ncbi.nlm.nih.gov/pubmed/18077370?dopt=AbstractPlus]. http://www.guidetopharmacology.org/GRAC/LigandDisplayForward?ligandId=4822 appears to act as a competitive substrate inhibitor of SMVT [http://www.ncbi.nlm.nih.gov/pubmed/10329687?dopt=AbstractPlus] and the anticonvulsant drugs http://www.guidetopharmacology.org/GRAC/LigandDisplayForward?ligandId=5338 and http://www.guidetopharmacology.org/GRAC/LigandDisplayForward?ligandId=5339 competitively block the transport of http://www.guidetopharmacology.org/GRAC/LigandDisplayForward?ligandId=4787 by brush border vesicles prepared from human intestine [http://www.ncbi.nlm.nih.gov/pubmed/2911998?dopt=AbstractPlus].

## http://www.guidetopharmacology.org/GRAC/FamilyDisplayForward?familyId=175

### Overview

Three different mammalian myo‐inositol cotransporters are currently known; two are the Na^+^‐coupled SMIT1 and SMIT2 tabulated below and the third is proton‐coupled HMIT (SLC2A13). SMIT1 and SMIT2 have a widespread and overlapping tissue location but in polarized cells, such as the Madin‐ Darby canine kidney cell line, they segregate to the basolateral and apical membranes, respectively [http://www.ncbi.nlm.nih.gov/pubmed/15181167?dopt=AbstractPlus]. In the nephron, SMIT1 mediates http://www.guidetopharmacology.org/GRAC/LigandDisplayForward?ligandId=4495 uptake as a ‘compatible osmolyte’ when inner medullary tubules are exposed to increases in extracellular osmolality, whilst SMIT2 mediates the reabsorption of http://www.guidetopharmacology.org/GRAC/LigandDisplayForward?ligandId=4495 from the filtrate. In some species (*e.g.* rat, but not rabbit) apically located SMIT2 is responsible for the uptake of http://www.guidetopharmacology.org/GRAC/LigandDisplayForward?ligandId=4495 from the intestinal lumen [http://www.ncbi.nlm.nih.gov/pubmed/17932225?dopt=AbstractPlus].

**Table:** 

Nomenclature	http://www.guidetopharmacology.org/GRAC/ObjectDisplayForward?objectId=924	http://www.guidetopharmacology.org/GRAC/ObjectDisplayForward?objectId=925
Systematic nomenclature	SLC5A3	SLC5A11
Common abbreviation	SMIT1	SMIT2
HGNC, UniProt	https://www.genenames.org/data/gene‐symbol‐report/#!/hgnc_id/HGNC:11038, http://www.uniprot.org/uniprot/P53794	https://www.genenames.org/data/gene‐symbol‐report/#!/hgnc_id/HGNC:23091, http://www.uniprot.org/uniprot/Q8WWX8
Substrates	http://www.guidetopharmacology.org/GRAC/LigandDisplayForward?ligandId=4495, http://www.guidetopharmacology.org/GRAC/LigandDisplayForward?ligandId=4649 > http://www.guidetopharmacology.org/GRAC/LigandDisplayForward?ligandId=4721 > http://www.guidetopharmacology.org/GRAC/LigandDisplayForward?ligandId=4720 > http://www.guidetopharmacology.org/GRAC/LigandDisplayForward?ligandId=4719, http://www.guidetopharmacology.org/GRAC/LigandDisplayForward?ligandId=4536, http://www.guidetopharmacology.org/GRAC/LigandDisplayForward?ligandId=4640 > http://www.guidetopharmacology.org/GRAC/LigandDisplayForward?ligandId=4646, http://www.guidetopharmacology.org/GRAC/LigandDisplayForward?ligandId=4722 > http://www.guidetopharmacology.org/GRAC/LigandDisplayForward?ligandId=4724 [http://www.ncbi.nlm.nih.gov/pubmed/7537337?dopt=AbstractPlus]	http://www.guidetopharmacology.org/GRAC/LigandDisplayForward?ligandId=4495 = http://www.guidetopharmacology.org/GRAC/LigandDisplayForward?ligandId=4645 > http://www.guidetopharmacology.org/GRAC/LigandDisplayForward?ligandId=4536 > http://www.guidetopharmacology.org/GRAC/LigandDisplayForward?ligandId=4724 > http://www.guidetopharmacology.org/GRAC/LigandDisplayForward?ligandId=4720 [http://www.ncbi.nlm.nih.gov/pubmed/12133831?dopt=AbstractPlus]
Stoichiometry	2 Na^+^ :1 *myo*‐inositol [http://www.ncbi.nlm.nih.gov/pubmed/7537337?dopt=AbstractPlus]	2Na^+^ :1 *myo*‐inositol [http://www.ncbi.nlm.nih.gov/pubmed/15613375?dopt=AbstractPlus]
Inhibitors	http://www.guidetopharmacology.org/GRAC/LigandDisplayForward?ligandId=4757 [http://www.ncbi.nlm.nih.gov/pubmed/12133831?dopt=AbstractPlus]	http://www.guidetopharmacology.org/GRAC/LigandDisplayForward?ligandId=4757 (p*K*_i_ 4.1) [http://www.ncbi.nlm.nih.gov/pubmed/12133831?dopt=AbstractPlus]

### Comments

The data tabulated are those for dog SMIT1 and rabbit SMIT2. SMIT2 transports http://www.guidetopharmacology.org/GRAC/LigandDisplayForward?ligandId=4645, but SMIT1 does not. In addition, whereas SMIT1 transports both http://www.guidetopharmacology.org/GRAC/LigandDisplayForward?ligandId=4724 and http://www.guidetopharmacology.org/GRAC/LigandDisplayForward?ligandId=4720 and http://www.guidetopharmacology.org/GRAC/LigandDisplayForward?ligandId=4722 and http://www.guidetopharmacology.org/GRAC/LigandDisplayForward?ligandId=4721, SMIT2 transports only the D‐isomers of these sugars [http://www.ncbi.nlm.nih.gov/pubmed/12133831?dopt=AbstractPlus, http://www.ncbi.nlm.nih.gov/pubmed/7537337?dopt=AbstractPlus]. Thus the substrate specificities of SMIT1 (for http://www.guidetopharmacology.org/GRAC/LigandDisplayForward?ligandId=4721) and SMIT2 (for http://www.guidetopharmacology.org/GRAC/LigandDisplayForward?ligandId=4645) allow discrimination between the two SMITs. Human SMIT2 appears not to transport glucose [http://www.ncbi.nlm.nih.gov/pubmed/19032932?dopt=AbstractPlus].

### Further reading on SLC5 family of sodium‐dependent glucose transporters

DeFronzo RA *et al*. (2017) Renal, metabolic and cardiovascular considerations of SGLT2 inhibition. *Nat Rev Nephrol* **13**: 11‐26 https://www.ncbi.nlm.nih.gov/pubmed/27941935?dopt=AbstractPlus

Koepsell H. (2017) The Na^+^‐D‐glucose cotransporters SGLT1 and SGLT2 are targets for the treatment of diabetes and cancer. *Pharmacol. Ther*. **170**: 148‐165 https://www.ncbi.nlm.nih.gov/pubmed/27773781?dopt=AbstractPlus

Lehmann A *et al*. (2016) Intestinal SGLT1 inmetabolic health and disease. *Am. J. Physiol. Gastrointest. Liver Physiol*. **310**: G887‐98 https://www.ncbi.nlm.nih.gov/pubmed/27012770?dopt=AbstractPlus

Wright EM. (2013) Glucose transport families SLC5 and SLC50. *Mol. Aspects Med*. **34**: 183‐96 https://www.ncbi.nlm.nih.gov/pubmed/23506865?dopt=AbstractPlus

Wright EM *et al*. (2011) Biology of human sodium glucose transporters. *Physiol. Rev*. **91**: 733‐94 https://www.ncbi.nlm.nih.gov/pubmed/21527736?dopt=AbstractPlus

## http://www.guidetopharmacology.org/GRAC/FamilyDisplayForward?familyId=144

### Overview

Members of the solute carrier family 6 (SLC6) of sodium‐ and (sometimes chloride‐) dependent neurotransmitter transporters [http://www.ncbi.nlm.nih.gov/pubmed/16540203?dopt=AbstractPlus, http://www.ncbi.nlm.nih.gov/pubmed/12719981?dopt=AbstractPlus, http://www.ncbi.nlm.nih.gov/pubmed/21752877?dopt=AbstractPlus] are primarily plasma membrane located and may be divided into four subfamilies that transport monoamines, http://www.guidetopharmacology.org/GRAC/LigandDisplayForward?ligandId=1067, http://www.guidetopharmacology.org/GRAC/LigandDisplayForward?ligandId=727 and neutral amino acids, plus the related bacterial NSS transporters [http://www.ncbi.nlm.nih.gov/pubmed/19022853?dopt=AbstractPlus]. The members of this superfamily share a structural motif of 10 TM segments that has been observed in crystal structures of the NSS bacterial homolog LeuT_Aa_, a Na^+^‐dependent amino acid transporter from *Aquiflex aeolicus* [http://www.ncbi.nlm.nih.gov/pubmed/16041361?dopt=AbstractPlus] and in several other transporter families structurally related to LeuT [http://www.ncbi.nlm.nih.gov/pubmed/19996368?dopt=AbstractPlus].

## http://www.guidetopharmacology.org/GRAC/FamilyDisplayForward?familyId=176

### Overview

Monoamine neurotransmission is limited by perisynaptic transporters. Presynapticmonoamine transporters allow recycling of synaptically released http://www.guidetopharmacology.org/GRAC/LigandDisplayForward?ligandId=484, http://www.guidetopharmacology.org/GRAC/LigandDisplayForward?ligandId=940 and http://www.guidetopharmacology.org/GRAC/LigandDisplayForward?ligandId=5.

**Table:** 

Nomenclature	http://www.guidetopharmacology.org/GRAC/ObjectDisplayForward?objectId=926	http://www.guidetopharmacology.org/GRAC/ObjectDisplayForward?objectId=927	http://www.guidetopharmacology.org/GRAC/ObjectDisplayForward?objectId=928
Systematic nomenclature	SLC6A2	SLC6A3	SLC6A4
HGNC, UniProt	https://www.genenames.org/data/gene‐symbol‐report/#!/hgnc_id/HGNC:11048, http://www.uniprot.org/uniprot/P23975	https://www.genenames.org/data/gene‐symbol‐report/#!/hgnc_id/HGNC:11049, http://www.uniprot.org/uniprot/Q01959	https://www.genenames.org/data/gene‐symbol‐report/#!/hgnc_id/HGNC:11050, http://www.uniprot.org/uniprot/P31645
Substrates	http://www.guidetopharmacology.org/GRAC/LigandDisplayForward?ligandId=4568, http://www.guidetopharmacology.org/GRAC/LigandDisplayForward?ligandId=4803, http://www.guidetopharmacology.org/GRAC/LigandDisplayForward?ligandId=4804	http://www.guidetopharmacology.org/GRAC/LigandDisplayForward?ligandId=4568, http://www.guidetopharmacology.org/GRAC/LigandDisplayForward?ligandId=4803, http://www.guidetopharmacology.org/GRAC/LigandDisplayForward?ligandId=4804	http://www.guidetopharmacology.org/GRAC/LigandDisplayForward?ligandId=4574, http://www.guidetopharmacology.org/GRAC/LigandDisplayForward?ligandId=4592
Endogenous substrates	http://www.guidetopharmacology.org/GRAC/LigandDisplayForward?ligandId=940, http://www.guidetopharmacology.org/GRAC/LigandDisplayForward?ligandId=479, http://www.guidetopharmacology.org/GRAC/LigandDisplayForward?ligandId=505	http://www.guidetopharmacology.org/GRAC/LigandDisplayForward?ligandId=940, http://www.guidetopharmacology.org/GRAC/LigandDisplayForward?ligandId=479, http://www.guidetopharmacology.org/GRAC/LigandDisplayForward?ligandId=505	http://www.guidetopharmacology.org/GRAC/LigandDisplayForward?ligandId=5
Stoichiometry	1 noradrenaline: 1 Na^+^:1 Cl^‐^ [http://www.ncbi.nlm.nih.gov/pubmed/8636118?dopt=AbstractPlus]	1 dopamine: 1^∪^2 Na^+^: 1 Cl^‐^ [http://www.ncbi.nlm.nih.gov/pubmed/8125921?dopt=AbstractPlus]	1 5‐HT:1 Na^+^:1 Cl^‐^ (in), + 1 K^+^ (out) [http://www.ncbi.nlm.nih.gov/pubmed/6853478?dopt=AbstractPlus]
Inhibitors	http://www.guidetopharmacology.org/GRAC/LigandDisplayForward?ligandId=9880 (p*K*_i_ 8.2) [http://www.ncbi.nlm.nih.gov/pubmed/29615471?dopt=AbstractPlus] – Rat	–	http://www.guidetopharmacology.org/GRAC/LigandDisplayForward?ligandId=9880 (p*K*_i_ 8.3) [http://www.ncbi.nlm.nih.gov/pubmed/29615471?dopt=AbstractPlus] – Rat
Sub/family‐selective inhibitors	http://www.guidetopharmacology.org/GRAC/LigandDisplayForward?ligandId=2586 (p*K*_i_ 5.2) [31]	http://www.guidetopharmacology.org/GRAC/LigandDisplayForward?ligandId=2586 (p*K*_i_ 6.3) [31]	http://www.guidetopharmacology.org/GRAC/LigandDisplayForward?ligandId=2586 (p*K*_i_ 6) [31]
Selective inhibitors	http://www.guidetopharmacology.org/GRAC/LigandDisplayForward?ligandId=4797 (p*K*_i_ 8.9), http://www.guidetopharmacology.org/GRAC/LigandDisplayForward?ligandId=7285 (pIC_50_ 8.8) [http://www.ncbi.nlm.nih.gov/pubmed/22420844?dopt=AbstractPlus], http://www.guidetopharmacology.org/GRAC/LigandDisplayForward?ligandId=4637 (p*K*_i_ 8.4), http://www.guidetopharmacology.org/GRAC/LigandDisplayForward?ligandId=7285 (p*K*_i_ 8.2) [http://www.ncbi.nlm.nih.gov/pubmed/18983139?dopt=AbstractPlus], http://www.guidetopharmacology.org/GRAC/LigandDisplayForward?ligandId=4792 (p*K*_i_ 8.1), http://www.guidetopharmacology.org/GRAC/LigandDisplayForward?ligandId=4808 (p*K*_i_ 8) [http://www.ncbi.nlm.nih.gov/pubmed/10812041?dopt=AbstractPlus]	http://www.guidetopharmacology.org/GRAC/LigandDisplayForward?ligandId=4797 (p*K*_i_ 8), http://www.guidetopharmacology.org/GRAC/LigandDisplayForward?ligandId=4606 (p*K*_i_ 7.9) [http://www.ncbi.nlm.nih.gov/pubmed/8878059?dopt=AbstractPlus], http://www.guidetopharmacology.org/GRAC/LigandDisplayForward?ligandId=4639 (p*K*_i_ 7.6), http://www.guidetopharmacology.org/GRAC/LigandDisplayForward?ligandId=7554 (p*K*_i_ 7.6) [http://www.ncbi.nlm.nih.gov/pubmed/21129986?dopt=AbstractPluss], http://www.guidetopharmacology.org/GRAC/LigandDisplayForward?ligandId=7236 (pIC_50_ 7.1) [http://www.ncbi.nlm.nih.gov/pubmed/17228864?dopt=AbstractPlus]	http://www.guidetopharmacology.org/GRAC/LigandDisplayForward?ligandId=2398 (p*K*_i_ 9.7) [http://www.ncbi.nlm.nih.gov/pubmed/9537821?dopt=AbstractPlus], http://www.guidetopharmacology.org/GRAC/LigandDisplayForward?ligandId=4790 (p*K*_i_ 9.6) [http://www.ncbi.nlm.nih.gov/pubmed/9537821?dopt=AbstractPlus], http://www.guidetopharmacology.org/GRAC/LigandDisplayForward?ligandId=2398 (p*K*_d_ 9.6) [http://www.ncbi.nlm.nih.gov/pubmed/9537821?dopt=AbstractPlus], http://www.guidetopharmacology.org/GRAC/LigandDisplayForward?ligandId=4798 (p*K*_i_ 9.1), http://www.guidetopharmacology.org/GRAC/LigandDisplayForward?ligandId=7177 (pIC_50_ 9) [http://www.ncbi.nlm.nih.gov/pubmed/19720528?dopt=AbstractPlus], http://www.guidetopharmacology.org/GRAC/LigandDisplayForward?ligandId=7901 (pIC_50_ 8.9) [233], http://www.guidetopharmacology.org/GRAC/LigandDisplayForward?ligandId=7189 (p*K*_d_ 8.7) [http://www.ncbi.nlm.nih.gov/pubmed/9537821?dopt=AbstractPlus], http://www.guidetopharmacology.org/GRAC/LigandDisplayForward?ligandId=203 (p*K*_i_ 8.5) [http://www.ncbi.nlm.nih.gov/pubmed/9537821?dopt=AbstractPlus], http://www.guidetopharmacology.org/GRAC/LigandDisplayForward?ligandId=7547 (p*K*_i_ 8.4) [http://www.ncbi.nlm.nih.gov/pubmed/18487050?dopt=AbstractPlus]
Labelled ligands	http://www.guidetopharmacology.org/GRAC/LigandDisplayForward?ligandId=4591 (Inhibitor) (p*K*_d_ 9.3) [http://www.ncbi.nlm.nih.gov/pubmed/15300361?dopt=AbstractPlus] – Rat, http://www.guidetopharmacology.org/GRAC/LigandDisplayForward?ligandId=4636 (Inhibitor) (p*K*_d_ 8.4)	http://www.guidetopharmacology.org/GRAC/LigandDisplayForward?ligandId=4638 (Inhibitor) (p*K*_d_ 8.5) [http://www.ncbi.nlm.nih.gov/pubmed/8302271?dopt=AbstractPlus], http://www.guidetopharmacology.org/GRAC/LigandDisplayForward?ligandId=4605 (Inhibitor) (p*K*_d_ 8) [http://www.ncbi.nlm.nih.gov/pubmed/8302271?dopt=AbstractPlus]	http://www.guidetopharmacology.org/GRAC/LigandDisplayForward?ligandId=4561 (Inhibitor) (p*K*_d_ 9.7), http://www.guidetopharmacology.org/GRAC/LigandDisplayForward?ligandId=4621 (Inhibitor) (p*K*_d_ 8.3)

### Comments

http://www.guidetopharmacology.org/GRAC/LigandDisplayForward?ligandId=5409 labels all three monoamine transporters (NET, DAT and SERT) with affinities between 0.5 and 5 nM. http://www.guidetopharmacology.org/GRAC/LigandDisplayForward?ligandId=2286 is an inhibitor of all three transporters with *p*K_i_ values between 6.5 and 7.2. Potential alternative splicing sites in non‐coding regions of SERT and NET have been identified. A bacterial homologue of SERT shows allosteric modulation by selected anti‐depressants [http://www.ncbi.nlm.nih.gov/pubmed/17687333?dopt=AbstractPlus].

## http://www.guidetopharmacology.org/GRAC/FamilyDisplayForward?familyId=177

### Overview

The activity of GABA‐transporters located predominantly upon neurones (GAT‐1), glia (GAT‐3) or both (GAT‐2, BGT‐ 1) serves to terminate phasic GABA‐ergic transmission, maintain low ambient extracellular concentrations of GABA, and recycle GABA for reuse by neurones. Nonetheless, ambient concentrations of http://www.guidetopharmacology.org/GRAC/LigandDisplayForward?ligandId=1067 are sufficient to sustain tonic inhibition mediated by high affinity GABAA receptors in certain neuronal populations [http://www.ncbi.nlm.nih.gov/pubmed/15111008?dopt=AbstractPlus]. GAT1 is the predominant GABA transporter in the brain and occurs primarily upon the terminals of presynaptic neurones and to a much lesser extent upon distal astocytic processes that are in proximity to axons terminals. GAT3 resides predominantly on distal astrocytic terminals that are close to the GABAergic synapse. By contrast, BGT1 occupies an extrasynaptic location possibly along with GAT2 which has limited expression in the brain [http://www.ncbi.nlm.nih.gov/pubmed/20026354?dopt=AbstractPlus]. TauT is a high affinity taurine transporter involved in osmotic balance that occurs in the brain and non‐neuronal tissues, such as the kidney, brush border membrane of the intestine and blood brain barrier [http://www.ncbi.nlm.nih.gov/pubmed/12719981?dopt=AbstractPlus, http://www.ncbi.nlm.nih.gov/pubmed/16734743?dopt=AbstractPlus]. CT1, which transports http://www.guidetopharmacology.org/GRAC/LigandDisplayForward?ligandId=4496, has a ubiquitous expression pattern, often co‐localizing with creatine kinase [http://www.ncbi.nlm.nih.gov/pubmed/12719981?dopt=AbstractPlus].

**Table:** 

Nomenclature	http://www.guidetopharmacology.org/GRAC/ObjectDisplayForward?objectId=929	http://www.guidetopharmacology.org/GRAC/ObjectDisplayForward?objectId=930	http://www.guidetopharmacology.org/GRAC/ObjectDisplayForward?objectId=931	http://www.guidetopharmacology.org/GRAC/ObjectDisplayForward?objectId=932	http://www.guidetopharmacology.org/GRAC/ObjectDisplayForward?objectId=933	http://www.guidetopharmacology.org/GRAC/ObjectDisplayForward?objectId=934
Systematic nomenclature	SLC6A1	SLC6A13	SLC6A11	SLC6A12	SLC6A6	SLC6A8
HGNC, UniProt	https://www.genenames.org/data/gene‐symbol‐report/#!/hgnc_id/HGNC:11042, http://www.uniprot.org/uniprot/P30531	https://www.genenames.org/data/gene‐symbol‐report/#!/hgnc_id/HGNC:11046, http://www.uniprot.org/uniprot/Q9NSD5	https://www.genenames.org/data/gene‐symbol‐report/#!/hgnc_id/HGNC:11044, http://www.uniprot.org/uniprot/P48066	https://www.genenames.org/data/gene‐symbol‐report/#!/hgnc_id/HGNC:11045, http://www.uniprot.org/uniprot/P48065	https://www.genenames.org/data/gene‐symbol‐report/#!/hgnc_id/HGNC:11052, http://www.uniprot.org/uniprot/P31641	https://www.genenames.org/data/gene‐symbol‐report/#!/hgnc_id/HGNC:11055, http://www.uniprot.org/uniprot/P48029
Substrates	http://www.guidetopharmacology.org/GRAC/LigandDisplayForward?ligandId=4564, http://www.guidetopharmacology.org/GRAC/LigandDisplayForward?ligandId=4691	http://www.guidetopharmacology.org/GRAC/LigandDisplayForward?ligandId=4564, http://www.guidetopharmacology.org/GRAC/LigandDisplayForward?ligandId=4691	http://www.guidetopharmacology.org/GRAC/LigandDisplayForward?ligandId=4691, http://www.guidetopharmacology.org/GRAC/LigandDisplayForward?ligandId=4564	–	–	–
Endogenous substrates	http://www.guidetopharmacology.org/GRAC/LigandDisplayForward?ligandId=1067	http://www.guidetopharmacology.org/GRAC/LigandDisplayForward?ligandId=2365, http://www.guidetopharmacology.org/GRAC/LigandDisplayForward?ligandId=1067	http://www.guidetopharmacology.org/GRAC/LigandDisplayForward?ligandId=2365, http://www.guidetopharmacology.org/GRAC/LigandDisplayForward?ligandId=1067	http://www.guidetopharmacology.org/GRAC/LigandDisplayForward?ligandId=1067, http://www.guidetopharmacology.org/GRAC/LigandDisplayForward?ligandId=4550	http://www.guidetopharmacology.org/GRAC/LigandDisplayForward?ligandId=2365, http://www.guidetopharmacology.org/GRAC/LigandDisplayForward?ligandId=2379, http://www.guidetopharmacology.org/GRAC/LigandDisplayForward?ligandId=1067 [http://www.ncbi.nlm.nih.gov/pubmed/19074966?dopt=AbstractPlus]	http://www.guidetopharmacology.org/GRAC/LigandDisplayForward?ligandId=4496
Stoichiometry	2Na^+^: 1Cl^‐^: 1GABA	2Na^+^: 1Cl^‐^: 1GABA	≥ 2Na^+^: 2 Cl^‐^: 1GABA	3Na^+^: 1 (or 2) Cl^‐^: 1GABA	2Na^+^: 1Cl^‐^: 1 taurine	Probably 2Na^+^: 1Cl^‐^: 1 creatine
Selective inhibitors	http://www.guidetopharmacology.org/GRAC/LigandDisplayForward?ligandId=4669 (pIC_50_ 7.4) [http://www.ncbi.nlm.nih.gov/pubmed/7851497?dopt=AbstractPlus], http://www.guidetopharmacology.org/GRAC/LigandDisplayForward?ligandId=4818 (pIC_50_ 7.2) [http://www.ncbi.nlm.nih.gov/pubmed/7851497?dopt=AbstractPlus], http://www.guidetopharmacology.org/GRAC/LigandDisplayForward?ligandId=4705 (pIC_50_ 6.9) [149], http://www.guidetopharmacology.org/GRAC/LigandDisplayForward?ligandId=4612 (pIC_50_ 6.6) [http://www.ncbi.nlm.nih.gov/pubmed/7851497?dopt=AbstractPlus], http://www.guidetopharmacology.org/GRAC/LigandDisplayForward?ligandId=4583 (pIC_50_ 4.9–5.7), http://www.guidetopharmacology.org/GRAC/LigandDisplayForward?ligandId=6512 (pIC_50_ 5.1–5.4), http://www.guidetopharmacology.org/GRAC/LigandDisplayForward?ligandId=4756 (pIC_50_ 5.4) [http://www.ncbi.nlm.nih.gov/pubmed/15550575?dopt=AbstractPlus] – Mouse, http://www.guidetopharmacology.org/GRAC/LigandDisplayForward?ligandId=6513 (pIC_50_ 3.6–3.9)	http://www.guidetopharmacology.org/GRAC/LigandDisplayForward?ligandId=4677 (pIC_50_ 4.7) [http://www.ncbi.nlm.nih.gov/pubmed/7874447?dopt=AbstractPlus] – Rat	http://www.guidetopharmacology.org/GRAC/LigandDisplayForward?ligandId=4677 (pIC_50_ 5.2) [http://www.ncbi.nlm.nih.gov/pubmed/7874447?dopt=AbstractPlus]	http://www.guidetopharmacology.org/GRAC/LigandDisplayForward?ligandId=4610 (p*K*_i_ 5.9) [http://www.ncbi.nlm.nih.gov/pubmed/9134205?dopt=AbstractPlus] – Mouse, http://www.guidetopharmacology.org/GRAC/LigandDisplayForward?ligandId=4583 (pIC_50_ 4.9), http://www.guidetopharmacology.org/GRAC/LigandDisplayForward?ligandId=6512 (pIC_50_ 3.7–4.7), http://www.guidetopharmacology.org/GRAC/LigandDisplayForward?ligandId=6513 (pIC_50_ 3.6–4.5), http://www.guidetopharmacology.org/GRAC/LigandDisplayForward?ligandId=4756 (pIC_50_ 4) [http://www.ncbi.nlm.nih.gov/pubmed/15550575?dopt=AbstractPlus] – Mouse	–	–
Labelled ligands	http://www.guidetopharmacology.org/GRAC/LigandDisplayForward?ligandId=4685 (Inhibitor)	–	–	–	–	–

### Comments

The IC50 values for GAT1‐4 reported in the table reflect the range reported in the literature from studies of both human and mouse transporters. There is a tendency towards lower IC_50_ values for the human orthologue [http://www.ncbi.nlm.nih.gov/pubmed/19275529?dopt=AbstractPlus]. http://www.guidetopharmacology.org/GRAC/LigandDisplayForward?ligandId=4677 is only weakly selective for GAT 2 and GAT3, with IC_50_ values in the range 22 to >30 *μ*M at GAT1 and BGT1, whereas http://www.guidetopharmacology.org/GRAC/LigandDisplayForward?ligandId=4610 has at least an order of magnitude selectivity for BGT1 [see [http://www.ncbi.nlm.nih.gov/pubmed/17175818?dopt=AbstractPlus, http://www.ncbi.nlm.nih.gov/pubmed/15451399?dopt=AbstractPlus] for reviews]. http://www.guidetopharmacology.org/GRAC/LigandDisplayForward?ligandId=5489 is a recently described compound that displays 20‐fold selectivity for GAT3 over GAT1 [http://www.ncbi.nlm.nih.gov/pubmed/16766089?dopt=AbstractPlus]. In addition to the inhibitors listed, http://www.guidetopharmacology.org/GRAC/LigandDisplayForward?ligandId=5490 is a moderately potent, though non‐selective, inhibitor of all cloned GABA transporters (IC_50_ = 26‐46 *μ*M; [http://www.ncbi.nlm.nih.gov/pubmed/8057281?dopt=AbstractPlus]). Diaryloxime and diarylvinyl ether derivatives of http://www.guidetopharmacology.org/GRAC/LigandDisplayForward?ligandId=4564 and http://www.guidetopharmacology.org/GRAC/LigandDisplayForward?ligandId=4691 that potently inhibit the uptake of http://www.guidetopharmacology.org/GRAC/LigandDisplayForward?ligandId=5410 into rat synaptosomes have been described [http://www.ncbi.nlm.nih.gov/pubmed/10479278?dopt=AbstractPlus]. Several derivatives of http://www.guidetopharmacology.org/GRAC/LigandDisplayForward?ligandId=5418 (*e.g.* http://www.guidetopharmacology.org/GRAC/LigandDisplayForward?ligandId=5419 and http://www.guidetopharmacology.org/GRAC/LigandDisplayForward?ligandId=5420) demonstrate selectivity as blockers of astroglial, versus neuronal, uptake of http://www.guidetopharmacology.org/GRAC/LigandDisplayForward?ligandId=1067 [see [http://www.ncbi.nlm.nih.gov/pubmed/17175818?dopt=AbstractPlus, http://www.ncbi.nlm.nih.gov/pubmed/21428813?dopt=AbstractPlus] for reviews]. GAT3 is inhibited by physiologically relevant concentrations of Zn^2+^ [http://www.ncbi.nlm.nih.gov/pubmed/15829583?dopt=AbstractPlus]. Taut transports http://www.guidetopharmacology.org/GRAC/LigandDisplayForward?ligandId=1067, but with low affinity, but CT1 does not, although it can be engineered to do so by mutagenesis guided by LeuT as a structural template [http://www.ncbi.nlm.nih.gov/pubmed/17400549?dopt=AbstractPlus]. Although inhibitors of http://www.guidetopharmacology.org/GRAC/LigandDisplayForward?ligandId=4496 transport by CT1 (*e.g*. http://www.guidetopharmacology.org/GRAC/LigandDisplayForward?ligandId=4707, http://www.guidetopharmacology.org/GRAC/LigandDisplayForward?ligandId=5491, http://www.guidetopharmacology.org/GRAC/LigandDisplayForward?ligandId=5492) are known (*e.g*. [http://www.ncbi.nlm.nih.gov/pubmed/9882430?dopt=AbstractPlus]) they insufficiently characterized to be included in the table.

## http://www.guidetopharmacology.org/GRAC/FamilyDisplayForward?familyId=178

### Overview

Two gene products, GlyT1 and GlyT2, are known that give rise to transporters that are predominantly located on glia and neurones, respectively. Five variants of GlyT1 (a,b,c,d & e) differing in their N‐ and C‐termini are generated by alternative promoter usage and splicing, and three splice variants of GlyT2 (a,b & c) have also been identified (see [http://www.ncbi.nlm.nih.gov/pubmed/16417482?dopt=AbstractPlus, http://www.ncbi.nlm.nih.gov/pubmed/15950877?dopt=AbstractPlus, http://www.ncbi.nlm.nih.gov/pubmed/16722246?dopt=AbstractPlus, http://www.ncbi.nlm.nih.gov/pubmed/12354619?dopt=AbstractPlus] for reviews). GlyT1 transporter isoforms expressed in glia surrounding glutamatergic synapses regulate synaptic http://www.guidetopharmacology.org/GRAC/LigandDisplayForward?ligandId=727 concentrations influencing NMDA receptor‐mediated neurotransmission [http://www.ncbi.nlm.nih.gov/pubmed/9861038?dopt=AbstractPlus, http://www.ncbi.nlm.nih.gov/pubmed/15555781?dopt=AbstractPlus], but also are important, in early neonatal life, for regulating glycine concentrations at inhibitory glycinergic synapses [http://www.ncbi.nlm.nih.gov/pubmed/14622582?dopt=AbstractPlus]. Homozygous mice engineered to totally lack GlyT1 exhibit severe respiratory and motor deficiencies due to hyperactive glycinergic signalling and die within the first postnatal day [http://www.ncbi.nlm.nih.gov/pubmed/14622582?dopt=AbstractPlus, http://www.ncbi.nlm.nih.gov/pubmed/15159536?dopt=AbstractPlus]. Disruption of GlyT1 restricted to forebrain neurones is associated with enhancement of EPSCs mediated by NMDA receptors and behaviours that are suggestive of a promnesic action [http://www.ncbi.nlm.nih.gov/pubmed/16554468?dopt=AbstractPlus]. GlyT2 transporters localised on the axons and boutons of glycinergic neurones appear crucial for efficient transmitter loading of synaptic vesicles but may not be essential for the termination of inhibitory neurotransmission [http://www.ncbi.nlm.nih.gov/pubmed/14622583?dopt=AbstractPlus, http://www.ncbi.nlm.nih.gov/pubmed/18815261?dopt=AbstractPlus]. Mice in which GlyT2 has been deleted develop a fatal hyperekplexia phenotype during the second postnatal week [http://www.ncbi.nlm.nih.gov/pubmed/14622583?dopt=AbstractPlus] and mutations in the human gene encoding GlyT2 (SLC6A5) have been identified in patients with hyperekplexia (reviewed by [http://www.ncbi.nlm.nih.gov/pubmed/18707791?dopt=AbstractPlus]). ATB^0+^ (SLC6A14) is a transporter for numerous dipolar and cationic amino acids and thus has a much broader substrate specificity than the glycine transporters alongside which it is grouped on the basis of structural similarity [http://www.ncbi.nlm.nih.gov/pubmed/12719981?dopt=AbstractPlus]. ATB^0+^ is expressed in various peripheral tissues [http://www.ncbi.nlm.nih.gov/pubmed/12719981?dopt=AbstractPlus]. By contrast PROT (SLC6A7), which is expressed only in brain in association with a subset of excitatory nerve terminals, shows specificity for the transport of http://www.guidetopharmacology.org/GRAC/LigandDisplayForward?ligandId=3314.

**Table:** 

Nomenclature	http://www.guidetopharmacology.org/GRAC/ObjectDisplayForward?objectId=935	http://www.guidetopharmacology.org/GRAC/ObjectDisplayForward?objectId=936	http://www.guidetopharmacology.org/GRAC/ObjectDisplayForward?objectId=937	http://www.guidetopharmacology.org/GRAC/ObjectDisplayForward?objectId=938
Systematic nomenclature	SLC6A9	SLC6A5	SLC6A14	SLC6A7
HGNC, UniProt	https://www.genenames.org/data/gene‐symbol‐report/#!/hgnc_id/HGNC:11056, http://www.uniprot.org/uniprot/P48067	https://www.genenames.org/data/gene‐symbol‐report/#!/hgnc_id/HGNC:11051, http://www.uniprot.org/uniprot/Q9Y345	https://www.genenames.org/data/gene‐symbol‐report/#!/hgnc_id/HGNC:11047, http://www.uniprot.org/uniprot/Q9UN76	https://www.genenames.org/data/gene‐symbol‐report/#!/hgnc_id/HGNC:11054, http://www.uniprot.org/uniprot/Q99884
Substrates	–	–	http://www.guidetopharmacology.org/GRAC/LigandDisplayForward?ligandId=4700, zwitterionic or cationic NOS inhibitors [http://www.ncbi.nlm.nih.gov/pubmed/11306607?dopt=AbstractPlus], http://www.guidetopharmacology.org/GRAC/LigandDisplayForward?ligandId=4694 [http://www.ncbi.nlm.nih.gov/pubmed/18522536?dopt=AbstractPlus], http://www.guidetopharmacology.org/GRAC/LigandDisplayForward?ligandId=4716 [http://www.ncbi.nlm.nih.gov/pubmed/15290873?dopt=AbstractPlus]	–
Endogenous substrates	http://www.guidetopharmacology.org/GRAC/LigandDisplayForward?ligandId=4713, http://www.guidetopharmacology.org/GRAC/LigandDisplayForward?ligandId=727	http://www.guidetopharmacology.org/GRAC/LigandDisplayForward?ligandId=727	http://www.guidetopharmacology.org/GRAC/LigandDisplayForward?ligandId=2365 [http://www.ncbi.nlm.nih.gov/pubmed/18599538?dopt=AbstractPlus, http://www.ncbi.nlm.nih.gov/pubmed/19074966?dopt=AbstractPlus] http://www.guidetopharmacology.org/GRAC/LigandDisplayForward?ligandId=3311 > http://www.guidetopharmacology.org/GRAC/LigandDisplayForward?ligandId=3312, http://www.guidetopharmacology.org/GRAC/LigandDisplayForward?ligandId=4814 > http://www.guidetopharmacology.org/GRAC/LigandDisplayForward?ligandId=3313 > http://www.guidetopharmacology.org/GRAC/LigandDisplayForward?ligandId=717 > http://www.guidetopharmacology.org/GRAC/LigandDisplayForward?ligandId=4794 > http://www.guidetopharmacology.org/GRAC/LigandDisplayForward?ligandId=726 [http://www.ncbi.nlm.nih.gov/pubmed/10446133?dopt=AbstractPlus]	http://www.guidetopharmacology.org/GRAC/LigandDisplayForward?ligandId=3314
Stoichiometry	2 Na^+^: 1 Cl^‐^: 1 glycine	3 Na^+^: 1 Cl^‐^: 1 glycine	2‐3 Na^+^: 1 Cl^‐^: 1 amino acid [http://www.ncbi.nlm.nih.gov/pubmed/10446133?dopt=AbstractPlus]	Probably 2 Na^+^: 1 Cl^‐^: 1 L‐proline
Inhibitors	http://www.guidetopharmacology.org/GRAC/LigandDisplayForward?ligandId=9057 (p*K*_i_ 7.9) [http://www.ncbi.nlm.nih.gov/pubmed/19410451?dopt=AbstractPlus]	http://www.guidetopharmacology.org/GRAC/LigandDisplayForward?ligandId=7546 (pEC_50_ <4.5) [http://www.ncbi.nlm.nih.gov/pubmed/20491477?dopt=AbstractPlus]	–	–
Selective inhibitors	http://www.guidetopharmacology.org/GRAC/LigandDisplayForward?ligandId=4618 (pIC_50_ 8.5–9.1), http://www.guidetopharmacology.org/GRAC/LigandDisplayForward?ligandId=4778 (pIC_50_ 8.7) [http://www.ncbi.nlm.nih.gov/pubmed/18621075?dopt=AbstractPlus], http://www.guidetopharmacology.org/GRAC/LigandDisplayForward?ligandId=4604 (pIC_50_ 8.6), http://www.guidetopharmacology.org/GRAC/LigandDisplayForward?ligandId=4712 (pIC_50_ 7.8), http://www.guidetopharmacology.org/GRAC/LigandDisplayForward?ligandId=4598 (pIC_50_ 7.6), http://www.guidetopharmacology.org/GRAC/LigandDisplayForward?ligandId=7546 (pEC_50_ 7.5) [http://www.ncbi.nlm.nih.gov/pubmed/20491477?dopt=AbstractPlus]	http://www.guidetopharmacology.org/GRAC/LigandDisplayForward?ligandId=4667 (pIC_50_ 7.8) [http://www.ncbi.nlm.nih.gov/pubmed/11495577?dopt=AbstractPlus], http://www.guidetopharmacology.org/GRAC/LigandDisplayForward?ligandId=4622, http://www.guidetopharmacology.org/GRAC/LigandDisplayForward?ligandId=4767	http://www.guidetopharmacology.org/GRAC/LigandDisplayForward?ligandId=4693 (pIC_50_ 3.6) [http://www.ncbi.nlm.nih.gov/pubmed/18522536?dopt=AbstractPlus]	http://www.guidetopharmacology.org/GRAC/LigandDisplayForward?ligandId=8520 (pIC_50_ 7.7) [http://www.ncbi.nlm.nih.gov/pubmed/25037917?dopt=AbstractPlus], http://www.guidetopharmacology.org/GRAC/LigandDisplayForward?ligandId=4576 (pIC_50_ 7) [http://www.ncbi.nlm.nih.gov/pubmed/19159658?dopt=AbstractPlus]
Labelled ligands	http://www.guidetopharmacology.org/GRAC/LigandDisplayForward?ligandId=4602 (Binding) (p*K*_d_ 9) [http://www.ncbi.nlm.nih.gov/pubmed/12657266?dopt=AbstractPlus], http://www.guidetopharmacology.org/GRAC/LigandDisplayForward?ligandId=4490 (Binding) (p*K*_d_ 8.8) [http://www.ncbi.nlm.nih.gov/pubmed/20691713?dopt=AbstractPlus], http://www.guidetopharmacology.org/GRAC/LigandDisplayForward?ligandId=4584 (Binding) (p*K*_d_ 8.7) [http://www.ncbi.nlm.nih.gov/pubmed/18355687?dopt=AbstractPlus], http://www.guidetopharmacology.org/GRAC/LigandDisplayForward?ligandId=4491 (Binding) (p*K*_d_ 8.7) [http://www.ncbi.nlm.nih.gov/pubmed/20691713?dopt=AbstractPlus], http://www.guidetopharmacology.org/GRAC/LigandDisplayForward?ligandId=4603 (p*K*_d_ 8.1–8.5), http://www.guidetopharmacology.org/GRAC/LigandDisplayForward?ligandId=4619 (p*K*_d_ 7.7–8.2)	–	–	–
Comments	–	N‐Oleoyl‐L‐carnitine (0.3*μ*M, [http://www.ncbi.nlm.nih.gov/pubmed/22978602?dopt=AbstractPlus]) and and N‐arachidonoylglycine (IC_50_ 5‐8 *μ*M, [http://www.ncbi.nlm.nih.gov/pubmed/16899062?dopt=AbstractPlus) have been described as potential endogenous selective GlyT2 inhibitors	–	–

### Comments

http://www.guidetopharmacology.org/GRAC/LigandDisplayForward?ligandId=4713 is a selective transportable inhibitor of GlyT1 and also a weak agonist at the http://www.guidetopharmacology.org/GRAC/LigandDisplayForward?ligandId=727 binding site of the NMDA receptor [http://www.ncbi.nlm.nih.gov/pubmed/19433577?dopt=AbstractPlus], but has no effect on GlyT2. This difference has been attributed to a single glycine residue in TM6 (serine residue in GlyT2) [http://www.ncbi.nlm.nih.gov/pubmed/17383967?dopt=AbstractPlus]. Inhibition of GLYT1 by the sarcosine derivatives http://www.guidetopharmacology.org/GRAC/LigandDisplayForward?ligandId=4620, http://www.guidetopharmacology.org/GRAC/LigandDisplayForward?ligandId=4601 and http://www.guidetopharmacology.org/GRAC/LigandDisplayForward?ligandId=4600 is non‐competitive [http://www.ncbi.nlm.nih.gov/pubmed/12941372?dopt=AbstractPlus, http://www.ncbi.nlm.nih.gov/pubmed/18815213?dopt=AbstractPlus]. IC_50_ values for http://www.guidetopharmacology.org/GRAC/LigandDisplayForward?ligandId=4600 reported in the literature vary, most likely due to differences in assay conditions [http://www.ncbi.nlm.nih.gov/pubmed/11454468?dopt=AbstractPlus, http://www.ncbi.nlm.nih.gov/pubmed/12941372?dopt=AbstractPlus]. The tricyclic antidepressant http://www.guidetopharmacology.org/GRAC/LigandDisplayForward?ligandId=201 weakly inhibits GlyT2 (IC_50_ 92 *μ*M) with approximately 10‐fold selectivity over GlyT1 [http://www.ncbi.nlm.nih.gov/pubmed/10694221?dopt=AbstractPlus]. The endogenous lipids http://www.guidetopharmacology.org/GRAC/LigandDisplayForward?ligandId=2391 and http://www.guidetopharmacology.org/GRAC/LigandDisplayForward?ligandId=2364 exert opposing effects upon GlyT1a, inhibiting (IC_50_ 2 *μ*M) and potentiating (EC_50_ 13 *μ*M) transport currents, respectively [http://www.ncbi.nlm.nih.gov/pubmed/12558979?dopt=AbstractPlus]. http://www.guidetopharmacology.org/GRAC/LigandDisplayForward?ligandId=5493, http://www.guidetopharmacology.org/GRAC/LigandDisplayForward?ligandId=5494 and http://www.guidetopharmacology.org/GRAC/LigandDisplayForward?ligandId=5495 have been described as endogenous non‐competitive inhibitors of GlyT2a, but not GlyT1b [http://www.ncbi.nlm.nih.gov/pubmed/19875446?dopt=AbstractPlus, http://www.ncbi.nlm.nih.gov/pubmed/20860669?dopt=AbstractPlus, http://www.ncbi.nlm.nih.gov/pubmed/16899062?dopt=AbstractPlus]. Protons [http://www.ncbi.nlm.nih.gov/pubmed/10860934?dopt=AbstractPlus] and Zn^2+^ [http://www.ncbi.nlm.nih.gov/pubmed/15031290?dopt=AbstractPlus] act as non‐competitive inhibitors of GlyT1b, with IC_50_ values of 100 nM and 10 *μ*M respectively, but neither ion affects GlyT2 (reviewed by [http://www.ncbi.nlm.nih.gov/pubmed/15324920?dopt=AbstractPlus]). Glycine transport by GLYT1 is inhibited by http://www.guidetopharmacology.org/GRAC/LigandDisplayForward?ligandId=5212, whereas GLYT2 transport is stimulated (both in the presence of Na^+^) [http://www.ncbi.nlm.nih.gov/pubmed/21574997?dopt=AbstractPlus].

## http://www.guidetopharmacology.org/GRAC/FamilyDisplayForward?familyId=179

### Overview

Certain members of neutral amino acid transport family are expressed upon the apical surface of epithelial cells and are important for the absorption of amino acids from the duodenum, jejunum and ileum and their reabsorption within the proximal tubule of the nephron (i.e. B^0^AT1 (SLC6A19), SLC6A18, SLC6A20). Others may function as transporters for neurotransmitters or their precursors (i.e. B^0^AT2, SLC6A17) [http://www.ncbi.nlm.nih.gov/pubmed/18400692?dopt=AbstractPlus]. B^0^AT1 has been proposed as a drug target to treat phenylketonuria [http://www.ncbi.nlm.nih.gov/pubmed/30046012?dopt=AbstractPlus].

**Table:** 

Nomenclature	http://www.guidetopharmacology.org/GRAC/ObjectDisplayForward?objectId=939	http://www.guidetopharmacology.org/GRAC/ObjectDisplayForward?objectId=940	http://www.guidetopharmacology.org/GRAC/ObjectDisplayForward?objectId=941	http://www.guidetopharmacology.org/GRAC/ObjectDisplayForward?objectId=942	http://www.guidetopharmacology.org/GRAC/ObjectDisplayForward?objectId=943	http://www.guidetopharmacology.org/GRAC/ObjectDisplayForward?objectId=944
Systematic nomenclature	SLC6A19	SLC6A15	SLC6A18	SLC6A16	SLC6A17	SLC6A20
HGNC, UniProt	https://www.genenames.org/data/gene‐symbol‐report/#!/hgnc_id/HGNC:27960, http://www.uniprot.org/uniprot/Q695T7	https://www.genenames.org/data/gene‐symbol‐report/#!/hgnc_id/HGNC:13621, http://www.uniprot.org/uniprot/Q9H2J7	https://www.genenames.org/data/gene‐symbol‐report/#!/hgnc_id/HGNC:26441, http://www.uniprot.org/uniprot/Q96N87	https://www.genenames.org/data/gene‐symbol‐report/#!/hgnc_id/HGNC:13622, http://www.uniprot.org/uniprot/Q9GZN6	https://www.genenames.org/data/gene‐symbol‐report/#!/hgnc_id/HGNC:31399, http://www.uniprot.org/uniprot/Q9H1V8	https://www.genenames.org/data/gene‐symbol‐report/#!/hgnc_id/HGNC:30927, http://www.uniprot.org/uniprot/Q9NP91
Endogenous substrates	http://www.guidetopharmacology.org/GRAC/LigandDisplayForward?ligandId=3312, http://www.guidetopharmacology.org/GRAC/LigandDisplayForward?ligandId=4814, http://www.guidetopharmacology.org/GRAC/LigandDisplayForward?ligandId=3311, http://www.guidetopharmacology.org/GRAC/LigandDisplayForward?ligandId=4794 > http://www.guidetopharmacology.org/GRAC/LigandDisplayForward?ligandId=4533, http://www.guidetopharmacology.org/GRAC/LigandDisplayForward?ligandId=3313, http://www.guidetopharmacology.org/GRAC/LigandDisplayForward?ligandId=720, http://www.guidetopharmacology.org/GRAC/LigandDisplayForward?ligandId=726 > http://www.guidetopharmacology.org/GRAC/LigandDisplayForward?ligandId=4785, http://www.guidetopharmacology.org/GRAC/LigandDisplayForward?ligandId=727, http://www.guidetopharmacology.org/GRAC/LigandDisplayForward?ligandId=3314 [http://www.ncbi.nlm.nih.gov/pubmed/16540203?dopt=AbstractPlus]	http://www.guidetopharmacology.org/GRAC/LigandDisplayForward?ligandId=3314 > http://www.guidetopharmacology.org/GRAC/LigandDisplayForward?ligandId=720, http://www.guidetopharmacology.org/GRAC/LigandDisplayForward?ligandId=4794, http://www.guidetopharmacology.org/GRAC/LigandDisplayForward?ligandId=4814, http://www.guidetopharmacology.org/GRAC/LigandDisplayForward?ligandId=3312 > http://www.guidetopharmacology.org/GRAC/LigandDisplayForward?ligandId=3311, http://www.guidetopharmacology.org/GRAC/LigandDisplayForward?ligandId=4785, http://www.guidetopharmacology.org/GRAC/LigandDisplayForward?ligandId=4533, http://www.guidetopharmacology.org/GRAC/LigandDisplayForward?ligandId=726, http://www.guidetopharmacology.org/GRAC/LigandDisplayForward?ligandId=3313 > http://www.guidetopharmacology.org/GRAC/LigandDisplayForward?ligandId=727 [http://www.ncbi.nlm.nih.gov/pubmed/16540203?dopt=AbstractPlus]	http://www.guidetopharmacology.org/GRAC/LigandDisplayForward?ligandId=720, http://www.guidetopharmacology.org/GRAC/LigandDisplayForward?ligandId=727 > http://www.guidetopharmacology.org/GRAC/LigandDisplayForward?ligandId=4814, http://www.guidetopharmacology.org/GRAC/LigandDisplayForward?ligandId=3313, http://www.guidetopharmacology.org/GRAC/LigandDisplayForward?ligandId=3312, http://www.guidetopharmacology.org/GRAC/LigandDisplayForward?ligandId=3310, http://www.guidetopharmacology.org/GRAC/LigandDisplayForward?ligandId=723 [http://www.ncbi.nlm.nih.gov/pubmed/20377526?dopt=AbstractPlus]	–	http://www.guidetopharmacology.org/GRAC/LigandDisplayForward?ligandId=3312, http://www.guidetopharmacology.org/GRAC/LigandDisplayForward?ligandId=4814, http://www.guidetopharmacology.org/GRAC/LigandDisplayForward?ligandId=3314 > http://www.guidetopharmacology.org/GRAC/LigandDisplayForward?ligandId=4782, http://www.guidetopharmacology.org/GRAC/LigandDisplayForward?ligandId=720, http://www.guidetopharmacology.org/GRAC/LigandDisplayForward?ligandId=723, http://www.guidetopharmacology.org/GRAC/LigandDisplayForward?ligandId=726 > http://www.guidetopharmacology.org/GRAC/LigandDisplayForward?ligandId=3310, http://www.guidetopharmacology.org/GRAC/LigandDisplayForward?ligandId=727 [http://www.ncbi.nlm.nih.gov/pubmed/19147495?dopt=AbstractPlus]	http://www.guidetopharmacology.org/GRAC/LigandDisplayForward?ligandId=3314
Stoichiometry	1 Na^+^: 1 amino acid [http://www.ncbi.nlm.nih.gov/pubmed/15804236?dopt=AbstractPlus]	1Na^+^ : 1 amino acid [http://www.ncbi.nlm.nih.gov/pubmed/16185194?dopt=AbstractPlus]	Na^+^‐ and Cl^‐^ ‐dependent transport [http://www.ncbi.nlm.nih.gov/pubmed/19478081?dopt=AbstractPlus]	–	Na^+^‐dependent, Cl^‐^‐independent transport [http://www.ncbi.nlm.nih.gov/pubmed/19147495?dopt=AbstractPlus]	2 Na^+^: 1 Cl^‐^: 1 imino acid [http://www.ncbi.nlm.nih.gov/pubmed/19657969?dopt=AbstractPlus]
Inhibitors	http://www.guidetopharmacology.org/GRAC/LigandDisplayForward?ligandId=10256 (pIC_50_ 6.4) [http://www.ncbi.nlm.nih.gov/pubmed/30589598?dopt=AbstractPlus], http://www.guidetopharmacology.org/GRAC/LigandDisplayForward?ligandId=7401 (pIC_50_ 4.6) [http://www.ncbi.nlm.nih.gov/pubmed/24704252?dopt=AbstractPlus] – Rat, http://www.guidetopharmacology.org/GRAC/LigandDisplayForward?ligandId=7601 (pIC_50_ 4.4) [http://www.ncbi.nlm.nih.gov/pubmed/28176326?dopt=AbstractPlus]	–	–	–	–	–
Selective inhibitors	–	http://www.guidetopharmacology.org/GRAC/LigandDisplayForward?ligandId=7216 (pIC_50_ 5.4) [http://www.ncbi.nlm.nih.gov/pubmed/25318072?dopt=AbstractPlus]	–	–	–	–
Comments	Mutations in B^0^AT1 are associated with Hartnup disorder	–	SLC6A18 is a functional transporter in mouse, but not in humans.	–	–	–

### Further reading on SLC6 neurotransmitter transporter family

Bermingham DP *et al*. (2016) Kinase‐dependent Regulation of Monoamine Neurotransmitter Transporters. *Pharmacol. Rev*. **68**: 888‐953 [https://www.ncbi.nlm.nih.gov/pubmed/27591044?dopt=AbstractPlus]

Bröer S *et al*. (2012) The solute carrier 6 family of transporters. *Br. J. Pharmacol*. **167**: 256‐78 [https://www.ncbi.nlm.nih.gov/pubmed/22519513?dopt=AbstractPlus]

Joncquel‐Chevalier Curt M *et al*. (2015) Creatine biosynthesis and transport in health and disease. *Biochimie* **119**: 146‐65 [https://www.ncbi.nlm.nih.gov/pubmed/26542286?dopt=AbstractPlus]

Lohr KM *et al*. (2017) Membrane transporters as mediators of synaptic dopamine dynamics: implications for disease. *Eur. J. Neurosci*. **45**: 20‐33 [https://www.ncbi.nlm.nih.gov/pubmed/27520881?dopt=AbstractPlus]

## http://www.guidetopharmacology.org/GRAC/FamilyDisplayForward?familyId=180

### Overview

The sodium/calcium exchangers (NCX) use the extracellular sodium concentration to facilitate the extrusion of calcium out of the cell. Alongside the plasma membrane Ca^2+^‐ATPase (http://www.guidetopharmacology.org/GRAC/FamilyDisplayForward?familyId=138#159_overview) and sarcoplasmic/endoplasmic reticulum Ca^2+^‐ATPase (http://www.guidetopharmacology.org/GRAC/FamilyDisplayForward?familyId=138), as well as the sodium/potassium/calcium exchangers (NKCX, http://www.guidetopharmacology.org/GRAC/FamilyDisplayForward?familyId=202), NCX allow recovery of intracellular calcium back to basal levels after cellular stimulation. When intracellular sodium ion levels rise, for example, following depolarisation, these transporters can operate in the reverse direction to allow calcium influx and sodium efflux, as an electrogenic mechanism. Structural modelling suggests the presence of 9 TM segments, with a large intracellular loop between the fifth and sixth TM segments.

**Table:** 

Nomenclature	http://www.guidetopharmacology.org/GRAC/ObjectDisplayForward?objectId=945	http://www.guidetopharmacology.org/GRAC/ObjectDisplayForward?objectId=946	http://www.guidetopharmacology.org/GRAC/ObjectDisplayForward?objectId=947
Systematic nomenclature	SLC8A1	SLC8A2	SLC8A3
Common abbreviation	NCX1	NCX2	NCX3
HGNC, UniProt	https://www.genenames.org/data/gene‐symbol‐report/#!/hgnc_id/HGNC:11068, http://www.uniprot.org/uniprot/P32418	https://www.genenames.org/data/gene‐symbol‐report/#!/hgnc_id/HGNC:11069, http://www.uniprot.org/uniprot/Q9UPR5	https://www.genenames.org/data/gene‐symbol‐report/#!/hgnc_id/HGNC:11070, http://www.uniprot.org/uniprot/P57103
Stoichiometry	3 Na^+^ (in) : 1 Ca^2+^ (out) or 4 Na^+^ (in) : 1 Ca^2+^ (out) [http://www.ncbi.nlm.nih.gov/pubmed/11916852?dopt=AbstractPlus]; Reverse mode 1 Ca^2+^ (in): 1 Na^+^ (out)	–	–
Selective inhibitors	–	–	http://www.guidetopharmacology.org/GRAC/LigandDisplayForward?ligandId=9484 (pIC_50_ 7.7) [http://www.ncbi.nlm.nih.gov/pubmed/16973719?dopt=AbstractPlus]

### Comments

Although subtype‐selective inhibitors of NCX function are not widely available, http://www.guidetopharmacology.org/GRAC/LigandDisplayForward?ligandId=4597 and http://www.guidetopharmacology.org/GRAC/LigandDisplayForward?ligandId=4593 act as non‐selective NCX inhibitors, while http://www.guidetopharmacology.org/GRAC/LigandDisplayForward?ligandId=4617, http://www.guidetopharmacology.org/GRAC/LigandDisplayForward?ligandId=4232, http://www.guidetopharmacology.org/GRAC/LigandDisplayForward?ligandId=4666, and http://www.guidetopharmacology.org/GRAC/LigandDisplayForward?ligandId=6481 [http://www.ncbi.nlm.nih.gov/pubmed/23647096?dopt=AbstractPlus] act to inhibit NCX function with varying degrees of selectivity. http://www.guidetopharmacology.org/GRAC/LigandDisplayForward?ligandId=8438 is a selective NCX3 inhibitor [http://www.ncbi.nlm.nih.gov/pubmed/25942323?dopt=AbstractPlus] and and http://www.guidetopharmacology.org/GRAC/LigandDisplayForward?ligandId=9484 inhibits NCX3 preferentially over other isoforms [http://www.ncbi.nlm.nih.gov/pubmed/16973719?dopt=AbstractPlus, http://www.ncbi.nlm.nih.gov/pubmed/27480939?dopt=AbstractPlus].

### Further reading on SLC8 family of sodium/calcium exchangers

Giladi M *et al*. (2016) Structure‐Functional Basis of Ion Transport in Sodium‐Calcium Exchanger (NCX) Proteins. *Int J Mol Sci* **17**: [https://www.ncbi.nlm.nih.gov/pubmed/27879668?dopt=AbstractPlus]

Khananshvili D. (2013) The SLC8 gene family of sodium‐calcium exchangers (NCX) ‐ structure, function, and regulation in health and disease. *Mol. Aspects Med*. **34**: 220‐35 [https://www.ncbi.nlm.nih.gov/pubmed/23506867?dopt=AbstractPlus]

Sekler I. (2015) Standing of giants shoulders the story of the mitochondrial Na(+)Ca(2+) exchanger. *Biochem. Biophys. Res. Commun*. **460**: 50‐2 [https://www.ncbi.nlm.nih.gov/pubmed/25998733?dopt=AbstractPlus]

## http://www.guidetopharmacology.org/GRAC/FamilyDisplayForward?familyId=181

### Overview

Sodium/hydrogen exchangers or sodium/proton antiports are a family of transporters that maintain cellular pH by utilising the sodium gradient across the plasma membrane to extrude protons produced by metabolism, in a stoichiometry of 1 Na^+^ (in) : 1 H^+^ (out). Several isoforms, NHE6, NHE7, NHE8 and NHE9 appear to locate on intracellularmembranes [http://www.ncbi.nlm.nih.gov/pubmed/11641397?dopt=AbstractPlus, http://www.ncbi.nlm.nih.gov/pubmed/15522866?dopt=AbstractPlus, http://www.ncbi.nlm.nih.gov/pubmed/11279194?dopt=AbstractPlus]. Li^+^ and NH_4_^+^, but not K^+^, ions may also be transported by some isoforms. Modelling of the topology of these transporters indicates 12 TM regions with an extended intracellular C‐terminus containing multiple regulatory sites. NHE1 is considered to be a ubiquitously‐expressed ‘housekeeping’ transporter. NHE3 is highly expressed in the intestine and kidneys and regulate sodium movements in those tissues. NHE10 is present in sperm [http://www.ncbi.nlm.nih.gov/pubmed/14634667?dopt=AbstractPlus] and osteoclasts [http://www.ncbi.nlm.nih.gov/pubmed/18269914?dopt=AbstractPlus]; gene disruption results in infertile male mice [http://www.ncbi.nlm.nih.gov/pubmed/14634667?dopt=AbstractPlus].

Information on members of this family may be found in the http://www.guidetopharmacology.org/GRAC/FamilyDisplayForward?familyId=181.

### Comments

Analogues of the non‐selective cation transport inhibitor amiloride appear to inhibit NHE function through competitive inhibition of the extracellular Na^+^ binding site. The more selective amiloride analogues http://www.guidetopharmacology.org/GRAC/LigandDisplayForward?ligandId=4595 and http://www.guidetopharmacology.org/GRAC/LigandDisplayForward?ligandId=4186 exhibit a rank order of affinity of inhibition of NHE1 > NHE2 > NHE3 [http://www.ncbi.nlm.nih.gov/pubmed/8246907?dopt=AbstractPlus, http://www.ncbi.nlm.nih.gov/pubmed/8415663?dopt=AbstractPlus, http://www.ncbi.nlm.nih.gov/pubmed/7685025?dopt=AbstractPlus].

### Further reading on SLC9 family of sodium/hydrogen exchangers

Donowitz M *et al*. (2013) SLC9/NHE gene family, a plasma membrane and organellar family of Na^+^/H^+^ exchangers. *Mol. Aspects Med*. **34**: 236‐51 [https://www.ncbi.nlm.nih.gov/pubmed/23506868?dopt=AbstractPlus]

Kato A *et al*. (2011) Regulation of electroneutral NaCl absorption by the small intestine. *Annu. Rev. Physiol*. **73**: 261‐81 [https://www.ncbi.nlm.nih.gov/pubmed/21054167?dopt=AbstractPlus]

Ohgaki R *et al*. (2011) Organellar Na+/H+ exchangers: novel players in organelle pH regulation and their emerging functions. *Biochemistry* **50**: 443‐50 [https://www.ncbi.nlm.nih.gov/pubmed/21171650?dopt=AbstractPlus]

Parker MD *et al*. (2015) Na+‐H+ exchanger‐1 (NHE1) regulation in kidney proximal tubule. *Cell. Mol. Life Sci*. **72**: 2061‐74 [https://www.ncbi.nlm.nih.gov/pubmed/25680790?dopt=AbstractPlus]

Ruffin VA *et al*. (2014) Intracellular pH regulation by acid‐base transporters in mammalian neurons. *Front Physiol* **5**: 43 [https://www.ncbi.nlm.nih.gov/pubmed/24592239?dopt=AbstractPlus]

## http://www.guidetopharmacology.org/GRAC/FamilyDisplayForward?familyId=182

### Overview

The SLC10 family transport bile acids, sulphated solutes, and other xenobiotics in a sodium‐dependent manner. The founding members, SLC10A1 (NTCP) and SLC10A2 (ASBT) function, along with members of the ABC transporter family (MDR1/ABCB1, BSEP/ABCB11 andMRP2/ABCC2) and the organic solute transporter obligate heterodimer OSTa:OSTß (SLC51), to maintain the enterohepatic circulation of bile acids [http://www.ncbi.nlm.nih.gov/pubmed/19498215?dopt=AbstractPlus, http://www.ncbi.nlm.nih.gov/pubmed/20103563?dopt=AbstractPlus]. SLC10A6 (SOAT) functions as a sodium‐dependent transporter of sulphated solutes including sulfphated steroids and bile acids [http://www.ncbi.nlm.nih.gov/pubmed/17491011?dopt=AbstractPlus, http://www.ncbi.nlm.nih.gov/pubmed/15020217?dopt=AbstractPlus]. Transport function has not yet been demonstrated for the 4 remaining members of the SLC10 family, SLC10A3 (P3), SLC10A4 (P4), SLC10A5 (P5), and SLC10A7 (P7), and the identity of their endogenous substrates remain unknown [http://www.ncbi.nlm.nih.gov/pubmed/17632081?dopt=AbstractPlus, http://www.ncbi.nlm.nih.gov/pubmed/15020217?dopt=AbstractPlus, http://www.ncbi.nlm.nih.gov/pubmed/17628207?dopt=AbstractPlus, http://www.ncbi.nlm.nih.gov/pubmed/19682536?dopt=AbstractPlus]. Members of the SLC10 family are predicted to have seven transmembrane domains with an extracellular N‐terminus and cytoplasmic C‐terminus [http://www.ncbi.nlm.nih.gov/pubmed/16411770?dopt=AbstractPlus, http://www.ncbi.nlm.nih.gov/pubmed/10471288?dopt=AbstractPlus].

**Table:** 

Nomenclature	http://www.guidetopharmacology.org/GRAC/ObjectDisplayForward?objectId=959	http://www.guidetopharmacology.org/GRAC/ObjectDisplayForward?objectId=960	http://www.guidetopharmacology.org/GRAC/ObjectDisplayForward?objectId=961
Systematic nomenclature	SLC10A1	SLC10A2	SLC10A6
Common abbreviation	NTCP	ASBT	SOAT
HGNC, UniProt	https://www.genenames.org/data/gene‐symbol‐report/#!/hgnc_id/HGNC:10905, http://www.uniprot.org/uniprot/Q14973	https://www.genenames.org/data/gene‐symbol‐report/#!/hgnc_id/HGNC:10906, http://www.uniprot.org/uniprot/Q12908	https://www.genenames.org/data/gene‐symbol‐report/#!/hgnc_id/HGNC:30603, http://www.uniprot.org/uniprot/Q3KNW5
Substrates	–	http://www.guidetopharmacology.org/GRAC/LigandDisplayForward?ligandId=4714 > http://www.guidetopharmacology.org/GRAC/LigandDisplayForward?ligandId=4715, http://www.guidetopharmacology.org/GRAC/LigandDisplayForward?ligandId=4545 > http://www.guidetopharmacology.org/GRAC/LigandDisplayForward?ligandId=4547 http://www.guidetopharmacology.org/GRAC/LigandDisplayForward?ligandId=609 > cholic acid [http://www.ncbi.nlm.nih.gov/pubmed/9458785?dopt=AbstractPlus]	http://www.guidetopharmacology.org/GRAC/LigandDisplayForward?ligandId=4290 [http://www.ncbi.nlm.nih.gov/pubmed/17491011?dopt=AbstractPlus], http://www.guidetopharmacology.org/GRAC/LigandDisplayForward?ligandId=4749, http://www.guidetopharmacology.org/GRAC/LigandDisplayForward?ligandId=4528 [http://www.ncbi.nlm.nih.gov/pubmed/15020217?dopt=AbstractPlus], http://www.guidetopharmacology.org/GRAC/LigandDisplayForward?ligandId=4548
Endogenous substrates	http://www.guidetopharmacology.org/GRAC/LigandDisplayForward?ligandId=4528 [http://www.ncbi.nlm.nih.gov/pubmed/9458785?dopt=AbstractPlus, http://www.ncbi.nlm.nih.gov/pubmed/17632081?dopt=AbstractPlus, http://www.ncbi.nlm.nih.gov/pubmed/9398014?dopt=AbstractPlus], http://www.guidetopharmacology.org/GRAC/LigandDisplayForward?ligandId=4749, iodothyronine sulphates [http://www.ncbi.nlm.nih.gov/pubmed/19682536?dopt=AbstractPlus] http://www.guidetopharmacology.org/GRAC/LigandDisplayForward?ligandId=4746, http://www.guidetopharmacology.org/GRAC/LigandDisplayForward?ligandId=4547, http://www.guidetopharmacology.org/GRAC/LigandDisplayForward?ligandId=4747 > http://www.guidetopharmacology.org/GRAC/LigandDisplayForward?ligandId=4544 > http://www.guidetopharmacology.org/GRAC/LigandDisplayForward?ligandId=609 [http://www.ncbi.nlm.nih.gov/pubmed/9398014?dopt=AbstractPlus]	–	–
Stoichiometry	2 Na^+^: 1 bile acid [http://www.ncbi.nlm.nih.gov/pubmed/16411770?dopt=AbstractPlus, http://www.ncbi.nlm.nih.gov/pubmed/17491011?dopt=AbstractPlus]	>1 Na^+^: 1 bile acid [http://www.ncbi.nlm.nih.gov/pubmed/9458785?dopt=AbstractPlus, http://www.ncbi.nlm.nih.gov/pubmed/9856990?dopt=AbstractPlus]	–
Inhibitors	http://www.guidetopharmacology.org/GRAC/LigandDisplayForward?ligandId=63 (pIC_50_ 8.2) [http://www.ncbi.nlm.nih.gov/pubmed/10565843?dopt=AbstractPlus], http://www.guidetopharmacology.org/GRAC/LigandDisplayForward?ligandId=1024 (pIC_50_ 6) [http://www.ncbi.nlm.nih.gov/pubmed/10565843?dopt=AbstractPlus], http://www.guidetopharmacology.org/GRAC/LigandDisplayForward?ligandId=7596 (pIC_50_ 5.3) [http://www.ncbi.nlm.nih.gov/pubmed/10565843?dopt=AbstractPlus], http://www.guidetopharmacology.org/GRAC/LigandDisplayForward?ligandId=1024 (p*K*_i_ 5.1) [http://www.ncbi.nlm.nih.gov/pubmed/23339484?dopt=AbstractPlus], http://www.guidetopharmacology.org/GRAC/LigandDisplayForward?ligandId=589 (p*K*_i_ 4.9) [http://www.ncbi.nlm.nih.gov/pubmed/23339484?dopt=AbstractPlus]	http://www.guidetopharmacology.org/GRAC/LigandDisplayForward?ligandId=9996t (pIC_50_ 8.9) [237], http://www.guidetopharmacology.org/GRAC/LigandDisplayForward?ligandId=6530 (pIC_50_ 8.8) [http://www.ncbi.nlm.nih.gov/pubmed/12810816?dopt=AbstractPlus], http://www.guidetopharmacology.org/GRAC/LigandDisplayForward?ligandId=6529 (pIC_50_ 7.3) [http://www.ncbi.nlm.nih.gov/pubmed/14552767?dopt=AbstractPlus, http://www.ncbi.nlm.nih.gov/pubmed/23678871?dopt=AbstractPlus]	–
Labelled ligands	–	http://www.guidetopharmacology.org/GRAC/LigandDisplayForward?ligandId=4546 [http://www.ncbi.nlm.nih.gov/pubmed/9458785?dopt=AbstractPlus]	–
Comments	–	http://www.guidetopharmacology.org/GRAC/LigandDisplayForward?ligandId=4770 is a fluorescent bile acid analogue used as a probe [http://www.ncbi.nlm.nih.gov/pubmed/9856990?dopt=AbstractPlus].	–

### Comments

Heterologously expressed SLC10A4 [http://www.ncbi.nlm.nih.gov/pubmed/18355966?dopt=AbstractPlus] or SLC10A7 [http://www.ncbi.nlm.nih.gov/pubmed/17628207?dopt=AbstractPlus] failed to exhibit significant transport of http://www.guidetopharmacology.org/GRAC/LigandDisplayForward?ligandId=4547, http://www.guidetopharmacology.org/GRAC/LigandDisplayForward?ligandId=4290, http://www.guidetopharmacology.org/GRAC/LigandDisplayForward?ligandId=4528 or http://www.guidetopharmacology.org/GRAC/LigandDisplayForward?ligandId=4551. SLC10A4 has recently been suggested to associate with neuronal vesicles [http://www.ncbi.nlm.nih.gov/pubmed/21742018?dopt=AbstractPlus].

### Further reading on SLC10 family of sodium‐bile acid co‐transporters

Anwer MS *et al*. (2014) Sodium‐dependent bile salt transporters of the SLC10A transporter family: more than solute transporters. *Pflugers Arch*. **466**: 77‐89 [https://www.ncbi.nlm.nih.gov/pubmed/24196564?dopt=AbstractPlus]

Claro da Silva T *et al*. (2013) The solute carrier family 10 (SLC10): beyond bile acid transport. *Mol. Aspects Med*. **34**: 252‐69 [https://www.ncbi.nlm.nih.gov/pubmed/23506869?dopt=AbstractPlus]

Dawson PA. (2017) Roles of Ileal ASBT and OSTa‐OSTß in Regulating Bile Acid Signaling. *Dig Dis* 35: 261‐266 [https://www.ncbi.nlm.nih.gov/pubmed/28249269?dopt=AbstractPlus]

Zwicker BL *et al*. (2013) Transport and biological activities of bile acids. *Int. J. Biochem. Cell Biol*. **45**: 1389‐98 [https://www.ncbi.nlm.nih.gov/pubmed/23603607?dopt=AbstractPlus]

## http://www.guidetopharmacology.org/GRAC/FamilyDisplayForward?familyId=183

### Overview

The family of proton‐coupled metal ion transporters are responsible for movements of divalent cations, particularly ferrous and manganese ions, across the cell membrane (SLC11A2/DMT1) and across endosomal (SLC11A2/DMT1) or lysosomal/phagosomal membranes (SLC11A1/NRAMP1), dependent on proton transport. Both proteins appear to have 12 TM regions and cytoplasmic N‐ and C‐ termini. NRAMP1 is involved in antimicrobial action in macrophages, although its precise mechanism is undefined. Facilitated diffusion of divalent cations into phagosomes may increase intravesicular free radicals to damage the pathogen. Alternatively, export of divalent cations from the phagosome may deprive the pathogen of essential enzyme cofactors. SLC11A2/DMT1 is more widely expressed and appears to assist in divalent cation assimilation from the diet, as well as in phagocytotic cells.

**Table:** 

Nomenclature	http://www.guidetopharmacology.org/GRAC/ObjectDisplayForward?objectId=966	http://www.guidetopharmacology.org/GRAC/ObjectDisplayForward?objectId=967
Systematic nomenclature	SLC11A1	SLC11A2
HGNC, UniProt	https://www.genenames.org/data/gene‐symbol‐report/#!/hgnc_id/HGNC:10907, http://www.uniprot.org/uniprot/P49279	https://www.genenames.org/data/gene‐symbol‐report/#!/hgnc_id/HGNC:10908, http://www.uniprot.org/uniprot/P49281
Endogenous substrates	Fe^2+^, Mn^2+^	http://www.guidetopharmacology.org/GRAC/LigandDisplayForward?ligandId=4164, http://www.guidetopharmacology.org/GRAC/LigandDisplayForward?ligandId=4160, http://www.guidetopharmacology.org/GRAC/LigandDisplayForward?ligandId=2440, Fe^2+^, Mn^2+^
Stoichiometry	1 H^+^ : 1 Fe^2+^ (out) or 1 Fe^2+^ (in) : 1 H^+^ (out)	1 H^+^ : 1 Fe^2+^ (out) [http://www.ncbi.nlm.nih.gov/pubmed/9242408?dopt=AbstractPlus]
Inhibitors	–	http://www.guidetopharmacology.org/GRAC/LigandDisplayForward?ligandId=8814 (pIC_50_ 7.1) [http://www.ncbi.nlm.nih.gov/pubmed/22749870?dopt=AbstractPlus]

### Comments

Loss‐of‐function mutations in NRAMP1 are associated with increased susceptibility to microbial infection (http://omim.org/entry/607948). Loss‐of‐function mutations in DMT1 are associated with microcytic anemia (http://omim.org/entry/206100).

### Further reading on SLC11 family of proton‐coupled metal ion transporters

Codazzi F *et al*. (2015) Iron entry in neurons and astrocytes: a link with synaptic activity. *Front Mol Neurosci* **8**: 18 [https://www.ncbi.nlm.nih.gov/pubmed/26089776?dopt=AbstractPlus]

Montalbetti N *et al*. (2013) Mammalian iron transporters: families SLC11 and SLC40. *Mol. Aspects Med*. **34**: 270‐87 [https://www.ncbi.nlm.nih.gov/pubmed/23506870?dopt=AbstractPlus]

Wessling‐Resnick M. (2015) Nramp1 and Other Transporters Involved in Metal Withholding during Infection. *J. Biol. Chem*. **290**: 18984‐90 [https://www.ncbi.nlm.nih.gov/pubmed/26055722?dopt=AbstractPlus]

Zheng W *et al*. (2012) Regulation of brain iron and copper homeostasis by brain barrier systems: implication in neurodegenerative diseases. *Pharmacol. Ther*. **133**: 177‐88 [https://www.ncbi.nlm.nih.gov/pubmed/22115751?dopt=AbstractPlus]

## http://www.guidetopharmacology.org/GRAC/FamilyDisplayForward?familyId=184

### Overview

The SLC12 family of chloride transporters contribute to ion fluxes across a variety of tissues, particularly in the kidney and choroid plexus of the brain. Within this family, further subfamilies are identifiable: NKCC1, NKCC2 and NCC constitute a group of therapeutically‐relevant transporters, targets for loop and thiazide diuretics. These 12 TM proteins exhibit cytoplasmic termini and an extended extracellular loop at TM7/8 and are kidneyspecific (NKCC2 and NCC) or show a more widespread distribution (NKCC1). A second family, the K‐Cl co‐transporters are also 12 TM domain proteins with cytoplasmic termini, but with an extended extracellular loop at TM 5/6. CCC6 exhibits structural similarities with the K‐Cl co‐transporters, while CCC9 is divergent, with 11 TM domains and a cytoplasmic N‐terminus and extracellular C‐terminus.

**Table:** 

Nomenclature	http://www.guidetopharmacology.org/GRAC/ObjectDisplayForward?objectId=968	http://www.guidetopharmacology.org/GRAC/ObjectDisplayForward?objectId=969	http://www.guidetopharmacology.org/GRAC/ObjectDisplayForward?objectId=970	http://www.guidetopharmacology.org/GRAC/ObjectDisplayForward?objectId=971	http://www.guidetopharmacology.org/GRAC/ObjectDisplayForward?objectId=972
Systematic nomenclature	SLC12A1	SLC12A2	SLC12A3	SLC12A4	SLC12A5
Common abbreviation	NKCC2	NKCC1	NCC	KCC1	KCC2
HGNC, UniProt	https://www.genenames.org/data/gene‐symbol‐report/#!/hgnc_id/HGNC:10910, http://www.uniprot.org/uniprot/Q13621	https://www.genenames.org/data/gene‐symbol‐report/#!/hgnc_id/HGNC:10911, http://www.uniprot.org/uniprot/P55011	https://www.genenames.org/data/gene‐symbol‐report/#!/hgnc_id/HGNC:10912, http://www.uniprot.org/uniprot/P55017	https://www.genenames.org/data/gene‐symbol‐report/#!/hgnc_id/HGNC:10913, http://www.uniprot.org/uniprot/Q9UP95	https://www.genenames.org/data/gene‐symbol‐report/#!/hgnc_id/HGNC:13818, http://www.uniprot.org/uniprot/Q9H2X9
Stoichiometry	1 Na^+^ : 1 K^+^ : 2 Cl^‐^ (in)	1 Na^+^ : 1 K^+^ : 2 Cl^‐^ (in)	1 Na^+^ : 1 Cl^‐^ (in)	1 K^+^ : 1 Cl^‐^ (out)	1 K^+^ : 1 Cl^‐^ (out)
Inhibitors	http://www.guidetopharmacology.org/GRAC/LigandDisplayForward?ligandId=4837 (pIC_50_ 6.5) [http://www.ncbi.nlm.nih.gov/pubmed/11882915?dopt=AbstractPlus], http://www.guidetopharmacology.org/GRAC/LigandDisplayForward?ligandId=4742 (pIC_50_ 6) [http://www.ncbi.nlm.nih.gov/pubmed/11882915?dopt=AbstractPlus], http://www.guidetopharmacology.org/GRAC/LigandDisplayForward?ligandId=4839 (pIC_50_ 5.2) [http://www.ncbi.nlm.nih.gov/pubmed/11882915?dopt=AbstractPlus]	http://www.guidetopharmacology.org/GRAC/LigandDisplayForward?ligandId=4742 (pIC_50_ 5.6) [http://www.ncbi.nlm.nih.gov/pubmed/11882915?dopt=AbstractPlus], http://www.guidetopharmacology.org/GRAC/LigandDisplayForward?ligandId=4837 (pIC_50_ 5.6) [http://www.ncbi.nlm.nih.gov/pubmed/11882915?dopt=AbstractPlus], http://www.guidetopharmacology.org/GRAC/LigandDisplayForward?ligandId=4839 (pIC_50_ 5.1) [http://www.ncbi.nlm.nih.gov/pubmed/11882915?dopt=AbstractPlus]	http://www.guidetopharmacology.org/GRAC/LigandDisplayForward?ligandId=4835, http://www.guidetopharmacology.org/GRAC/LigandDisplayForward?ligandId=4167, http://www.guidetopharmacology.org/GRAC/LigandDisplayForward?ligandId=4836, http://www.guidetopharmacology.org/GRAC/LigandDisplayForward?ligandId=4838	http://www.guidetopharmacology.org/GRAC/LigandDisplayForward?ligandId=4589	http://www.guidetopharmacology.org/GRAC/LigandDisplayForward?ligandId=4665 (pIC_50_ 6.2) [http://www.ncbi.nlm.nih.gov/pubmed/19279215?dopt=AbstractPlus],http://www.guidetopharmacology.org/GRAC/LigandDisplayForward?ligandId=4589

**Table:** 

Nomenclature	http://www.guidetopharmacology.org/GRAC/ObjectDisplayForward?objectId=973	http://www.guidetopharmacology.org/GRAC/ObjectDisplayForward?objectId=974	http://www.guidetopharmacology.org/GRAC/ObjectDisplayForward?objectId=975
Systematic nomenclature	SLC12A6	SLC12A7	SLC12A8
Common abbreviation	KCC3	KCC4	CCC9
HGNC, UniProt	https://www.genenames.org/data/gene‐symbol‐report/#!/hgnc_id/HGNC:10914, http://www.uniprot.org/uniprot/Q9UHW9	https://www.genenames.org/data/gene‐symbol‐report/#!/hgnc_id/HGNC:10915, http://www.uniprot.org/uniprot/Q9Y666	https://www.genenames.org/data/gene‐symbol‐report/#!/hgnc_id/HGNC:15595, http://www.uniprot.org/uniprot/A0AV02
Substrates	–	–	http://www.guidetopharmacology.org/GRAC/LigandDisplayForward?ligandId=1369, http://www.guidetopharmacology.org/GRAC/LigandDisplayForward?ligandId=710, http://www.guidetopharmacology.org/GRAC/LigandDisplayForward?ligandId=3309, http://www.guidetopharmacology.org/GRAC/LigandDisplayForward?ligandId=2390
Stoichiometry	1 K^+^ : 1 Cl^‐^ (out)	1 K^+^ : 1 Cl^‐^ (out)	Unknown
Inhibitors	http://www.guidetopharmacology.org/GRAC/LigandDisplayForward?ligandId=4589	http://www.guidetopharmacology.org/GRAC/LigandDisplayForward?ligandId=4589	–
Comments	–	–	In mouse studies Slc12a8 has been shown to transport the nicotinamide adenine dinucleotide (NAD+) precursor, nicotinamide mononucleotide (NMN) in to cells, and administration of NMN produces anti‐ageing effects *in vivo* [261].

### Comments

http://www.guidetopharmacology.org/GRAC/LigandDisplayForward?ligandId=4589 is able to differentiate KCC isoforms from NKCC and NCC transporters, but also inhibits CFTR [http://www.ncbi.nlm.nih.gov/pubmed/11527541?dopt=AbstractPlus].

### Further reading on SLC12 family of cation‐coupled chloride transporters

Arroyo JP *et al*. (2013) The SLC12 family of electroneutral cation‐coupled chloride cotransporters. *Mol. Aspects Med*. **34**: 288‐98 [https://www.ncbi.nlm.nih.gov/pubmed/23506871?dopt=AbstractPlus]

Bachmann S *et al*. (2017) Regulation of renal Na‐(K)‐Cl cotransporters by vasopressin. *Pflugers Arch*. **469**: 889‐897 [https://www.ncbi.nlm.nih.gov/pubmed/28577072?dopt=AbstractPlus]

Bazúa‐Valenti S *et al*. (2016) Physiological role of SLC12 family members in the kidney. *Am. J. Physiol. Renal Physiol*. **311**: F131‐44 [https://www.ncbi.nlm.nih.gov/pubmed/27097893?dopt=AbstractPlus]

Huang X *et al*. (2016) Everything we always wanted to know about furosemide but were afraid to ask. *Am. J. Physiol. Renal Physiol*. **310**: F958‐71 [https://www.ncbi.nlm.nih.gov/pubmed/26911852?dopt=AbstractPlus]

Kahle KT *et al*. (2015) K‐Cl cotransporters, cell volume homeostasis, and neurological disease. *Trends Mol Med* **21**: 513‐23 [https://www.ncbi.nlm.nih.gov/pubmed/26142773?dopt=AbstractPlus]

Martín‐Aragón Baudel MA *et al*. (2017) Chloride co‐transporters as possible therapeutic targets for stroke. *J. Neurochem*. **140**: 195‐209 [https://www.ncbi.nlm.nih.gov/pubmed/27861901?dopt=AbstractPlus]

## http://www.guidetopharmacology.org/GRAC/FamilyDisplayForward?familyId=185

### Overview

Within the SLC13 family, two groups of transporters may be differentiated on the basis of the substrates transported: NaS1 and NaS2 convey sulphate, while NaC1‐3 transport carboxylates. NaS1 and NaS2 transporters are made up of 13 TM domains, with an intracellular N terminus and are electrogenic with physiological roles in the intestine, kidney and placenta. NaC1, NaC2 and NaC3 are made up of 11 TM domains with an intracellular N terminus and are electrogenic, with physiological roles in the kidney and liver.

**Table:** 

Nomenclature	http://www.guidetopharmacology.org/GRAC/ObjectDisplayForward?objectId=977	http://www.guidetopharmacology.org/GRAC/ObjectDisplayForward?objectId=978	http://www.guidetopharmacology.org/GRAC/ObjectDisplayForward?objectId=979	http://www.guidetopharmacology.org/GRAC/ObjectDisplayForward?objectId=980	http://www.guidetopharmacology.org/GRAC/ObjectDisplayForward?objectId=981
Systematic nomenclature	SLC13A1	SLC13A2	SLC13A3	SLC13A4	SLC13A5
Common abbreviation	NaS1	NaC1	NaC3	NaS2	NaC2
HGNC, UniProt	https://www.genenames.org/data/gene‐symbol‐report/#!/hgnc_id/HGNC:10916, http://www.uniprot.org/uniprot/Q9BZW2	https://www.genenames.org/data/gene‐symbol‐report/#!/hgnc_id/HGNC:10917, http://www.uniprot.org/uniprot/Q13183	https://www.genenames.org/data/gene‐symbol‐report/#!/hgnc_id/HGNC:14430, http://www.uniprot.org/uniprot/Q8WWT9	https://www.genenames.org/data/gene‐symbol‐report/#!/hgnc_id/HGNC:15827, http://www.uniprot.org/uniprot/Q9UKG4	https://www.genenames.org/data/gene‐symbol‐report/#!/hgnc_id/HGNC:23089, http://www.uniprot.org/uniprot/Q86YT5
Endogenous substrates	SeO_4_^2‐^, SO_4_^2‐^, S_2_O_3_^2‐^	http://www.guidetopharmacology.org/GRAC/LigandDisplayForward?ligandId=2478, http://www.guidetopharmacology.org/GRAC/LigandDisplayForward?ligandId=3637	http://www.guidetopharmacology.org/GRAC/LigandDisplayForward?ligandId=2478, http://www.guidetopharmacology.org/GRAC/LigandDisplayForward?ligandId=3637	SO_4_^2‐^	http://www.guidetopharmacology.org/GRAC/LigandDisplayForward?ligandId=2478, http://www.guidetopharmacology.org/GRAC/LigandDisplayForward?ligandId=4809
Stoichiometry	3 Na^+^ : 1 SO_4_^2‐^ (in)	3 Na^+^ : 1 dicarboxylate^2‐^ (in)	Unknown	3 Na^+^ : SO_4_^2‐^ (in)	Unknown

### Further reading on SLC13 family of sodium‐dependent sulphate/carboxylate transporters

Bergeron MJ *et al*. (2013) SLC13 family of Na^+^‐coupled di‐ and tri‐carboxylate/sulfate transporters. *Mol. Aspects Med*. **34**: 299‐312 [https://www.ncbi.nlm.nih.gov/pubmed/23506872?dopt=AbstractPlus]

Markovich D. (2014) Na^+^‐sulfate cotransporter SLC13A1. *Pflugers Arch*. **466**: 131‐7 [https://www.ncbi.nlm.nih.gov/pubmed/24193406?dopt=AbstractPlus]

Pajor AM. (2014) Sodium‐coupled dicarboxylate and citrate transporters from the SLC13 family. *Pflugers Arch*. **466**: 119‐30 [https://www.ncbi.nlm.nih.gov/pubmed/24114175?dopt=AbstractPlus]

## http://www.guidetopharmacology.org/GRAC/FamilyDisplayForward?familyId=186

### Overview

As a product of protein catabolism, urea is moved around the body and through the kidneys for excretion. Although there is experimental evidence for concentrative urea transporters, these have not been defined at the molecular level. The SLC14 family are facilitative transporters, allowing http://www.guidetopharmacology.org/GRAC/LigandDisplayForward?ligandId=4539 movement down its concentration gradient. Multiple splice variants of these transporters have been identified; for UT‐A transporters, in particular, there is evidence for cell‐specific expression of these variants with functional impact [http://www.ncbi.nlm.nih.gov/pubmed/21449978?dopt=AbstractPlus]. Topographical modelling suggests that the majority of the variants of SLC14 transporters have 10 TM domains, with a glycosylated extracellular loop at TM5/6, and intracellular C‐ and N‐termini. The UT‐A1 splice variant, exceptionally, has 20 TMdomains, equivalent to a combination of theUT‐A2 and UT‐A3 splice variants.

**Table:** 

Nomenclature	http://www.guidetopharmacology.org/GRAC/ObjectDisplayForward?objectId=982	http://www.guidetopharmacology.org/GRAC/ObjectDisplayForward?objectId=983
Systematic nomenclature	SLC14A1	SLC14A2
Common abbreviation	UT‐B	UT‐A
HGNC, UniProt	https://www.genenames.org/data/gene‐symbol‐report/#!/hgnc_id/HGNC:10918, http://www.uniprot.org/uniprot/Q13336	https://www.genenames.org/data/gene‐symbol‐report/#!/hgnc_id/HGNC:10919, http://www.uniprot.org/uniprot/Q15849
Substrates	http://www.guidetopharmacology.org/GRAC/LigandDisplayForward?ligandId=4661 [http://www.ncbi.nlm.nih.gov/pubmed/17506977?dopt=AbstractPlus], http://www.guidetopharmacology.org/GRAC/LigandDisplayForward?ligandId=4553 [http://www.ncbi.nlm.nih.gov/pubmed/17506977?dopt=AbstractPlus], http://www.guidetopharmacology.org/GRAC/LigandDisplayForward?ligandId=4662 [http://www.ncbi.nlm.nih.gov/pubmed/17506977?dopt=AbstractPlus]	–
Endogenous substrates	http://www.guidetopharmacology.org/GRAC/LigandDisplayForward?ligandId=4509 [http://www.ncbi.nlm.nih.gov/pubmed/17506977?dopt=AbstractPlus], http://www.guidetopharmacology.org/GRAC/LigandDisplayForward?ligandId=4539 [http://www.ncbi.nlm.nih.gov/pubmed/17506977?dopt=AbstractPlus], http://www.guidetopharmacology.org/GRAC/LigandDisplayForward?ligandId=4739 [http://www.ncbi.nlm.nih.gov/pubmed/17506977?dopt=AbstractPlus]	http://www.guidetopharmacology.org/GRAC/LigandDisplayForward?ligandId=4539 [http://www.ncbi.nlm.nih.gov/pubmed/18256317?dopt=AbstractPlus]
Stoichiometry	Equilibrative	Equilibrative
Inhibitors	http://www.guidetopharmacology.org/GRAC/LigandDisplayForward?ligandId=8881 (pIC_50_ ~8) [http://www.ncbi.nlm.nih.gov/pubmed/23597791?dopt=AbstractPlus], http://www.guidetopharmacology.org/GRAC/LigandDisplayForward?ligandId=8881 (pIC_50_ 7.6) [http://www.ncbi.nlm.nih.gov/pubmed/23597791?dopt=AbstractPlus] –Mouse	–

### Further reading on SLC14 family of facilitative urea transporters

Esteva‐Font C *et al*. (2015) Urea transporter proteins as targets for small‐molecule diuretics. *Nat Rev Nephrol* **11**: 113‐23 [https://www.ncbi.nlm.nih.gov/pubmed/25488859?dopt=AbstractPlus]

LeMoine CM *et al*. (2015) Evolution of urea transporters in vertebrates: adaptation to urea's multiple roles and metabolic sources. *J. Exp. Biol*. **218**: 1936‐1945 [https://www.ncbi.nlm.nih.gov/pubmed/26085670?dopt=AbstractPlus]

Pannabecker TL. (2013) Comparative physiology and architecture associated with the mammalian urine concentrating mechanism: role of inner medullary water and urea transport pathways in the rodent medulla. *Am. J. Physiol. Regul. Integr. Comp. Physiol*. **304**: R488‐503 [https://www.ncbi.nlm.nih.gov/pubmed/23364530?dopt=AbstractPlus]

Shayakul C *et al*. (2013) The urea transporter family (SLC14): physiological, pathological and structural aspects. *Mol. Aspects Med*. **34**: 313‐22 [https://www.ncbi.nlm.nih.gov/pubmed/23506873?dopt=AbstractPlus]

Stewart G. (2011) The emerging physiological roles of the SLC14A family of urea transporters. *Br. J. Pharmacol*. **164**: 1780‐92 [https://www.ncbi.nlm.nih.gov/pubmed/21449978?dopt=AbstractPlus]

## http://www.guidetopharmacology.org/GRAC/FamilyDisplayForward?familyId=187

### Overview

The Solute Carrier 15 (SLC15) family of peptide transporters, alias H^+^‐coupled oligopeptide cotransporter family, is a group of membrane transporters known for their key role in the cellular uptake of di‐ and tripeptides (di/tripeptides). Of its members, SLC15A1 (PEPT1) chiefly mediates intestinal absorption of luminal di/tripeptides from overall dietary protein digestion, SLC15A2 (PEPT2) mainly allows renal tubular reuptake of di/tripeptides from ultrafiltration and brain‐to‐blood efflux of di/tripeptides in the choroid plexus, SLC15A3 (PHT2) and SLC15A4 (PHT1) interact with both di/tripeptides and histidine, e.g. in certain immune cells, and SLC15A5 has unknown physiological function. In addition, the SLC15 family of peptide transporters variably interacts with a very large number of peptidomimetics and peptide‐like drugs. It is conceivable, based on the currently acknowledged structural and functional differences, to divide the SLC15 family of peptide transporters into two subfamilies.

**Table:** 

Nomenclature	http://www.guidetopharmacology.org/GRAC/ObjectDisplayForward?objectId=984	http://www.guidetopharmacology.org/GRAC/ObjectDisplayForward?objectId=985
Systematic nomenclature	SLC15A1	SLC15A2
Common abbreviation	PEPT1	PEPT2
HGNC, UniProt	https://www.genenames.org/data/gene‐symbol‐report/#!/hgnc_id/HGNC:10920, http://www.uniprot.org/uniprot/P46059	https://www.genenames.org/data/gene‐symbol‐report/#!/hgnc_id/HGNC:10921, http://www.uniprot.org/uniprot/Q16348
Substrates	http://www.guidetopharmacology.org/GRAC/LigandDisplayForward?ligandId=1022 [http://www.ncbi.nlm.nih.gov/pubmed/14578196?dopt=AbstractPlus, http://www.ncbi.nlm.nih.gov/pubmed/16568107?dopt=AbstractPlus, http://www.ncbi.nlm.nih.gov/pubmed/11375948?dopt=AbstractPlus, http://www.ncbi.nlm.nih.gov/pubmed/9835627?dopt=AbstractPlus, http://www.ncbi.nlm.nih.gov/pubmed/23259992?dopt=AbstractPlus], http://www.guidetopharmacology.org/GRAC/LigandDisplayForward?ligandId=9502 [http://www.ncbi.nlm.nih.gov/pubmed/27543355?dopt=AbstractPlus], http://www.guidetopharmacology.org/GRAC/LigandDisplayForward?ligandId=5421 [http://www.ncbi.nlm.nih.gov/pubmed/28943923?dopt=AbstractPlus, http://www.ncbi.nlm.nih.gov/pubmed/11518682?dopt=AbstractPlus, http://www.ncbi.nlm.nih.gov/pubmed/24744852?dopt=AbstractPlus, http://www.ncbi.nlm.nih.gov/pubmed/22950754?dopt=AbstractPlus], http://www.guidetopharmacology.org/GRAC/LigandDisplayForward?ligandId=9501 [http://www.ncbi.nlm.nih.gov/pubmed/21366347?dopt=AbstractPlus, http://www.ncbi.nlm.nih.gov/pubmed/23822979?dopt=AbstractPlus, http://www.ncbi.nlm.nih.gov/pubmed/24744852?dopt=AbstractPlus], http://www.guidetopharmacology.org/GRAC/LigandDisplayForward?ligandId=9503 [http://www.ncbi.nlm.nih.gov/pubmed/15128310?dopt=AbstractPlus], http://www.guidetopharmacology.org/GRAC/LigandDisplayForward?ligandId=5024 [http://www.ncbi.nlm.nih.gov/pubmed/17487240?dopt=AbstractPlus, http://www.ncbi.nlm.nih.gov/pubmed/15521010?dopt=AbstractPlus]	http://www.guidetopharmacology.org/GRAC/LigandDisplayForward?ligandId=5024 [http://www.ncbi.nlm.nih.gov/pubmed/29784761?dopt=AbstractPlus, http://www.ncbi.nlm.nih.gov/pubmed/23442152?dopt=AbstractPlus], http://www.guidetopharmacology.org/GRAC/LigandDisplayForward?ligandId=9501 [http://www.ncbi.nlm.nih.gov/pubmed/21366347?dopt=AbstractPlus, http://www.ncbi.nlm.nih.gov/pubmed/9888294?dopt=AbstractPlus, http://www.ncbi.nlm.nih.gov/pubmed/24744852?dopt=AbstractPlus, http://www.ncbi.nlm.nih.gov/pubmed/19913073?dopt=AbstractPlus, http://www.ncbi.nlm.nih.gov/pubmed/16041713?dopt=AbstractPlus, http://www.ncbi.nlm.nih.gov/pubmed/23442152?dopt=AbstractPlus, http://www.ncbi.nlm.nih.gov/pubmed/20868728?dopt=AbstractPlus, http://www.ncbi.nlm.nih.gov/pubmed/19612975?dopt=AbstractPlus], http://www.guidetopharmacology.org/GRAC/LigandDisplayForward?ligandId=5421 [http://www.ncbi.nlm.nih.gov/pubmed/24744852?dopt=AbstractPlus, http://www.ncbi.nlm.nih.gov/pubmed/22950754?dopt=AbstractPlus, http://www.ncbi.nlm.nih.gov/pubmed/9843719?dopt=AbstractPlus], http://www.guidetopharmacology.org/GRAC/LigandDisplayForward?ligandId=9504 [http://www.ncbi.nlm.nih.gov/pubmed/23442152?dopt=AbstractPlus, http://www.ncbi.nlm.nih.gov/pubmed/18474668?dopt=AbstractPlus], http://www.guidetopharmacology.org/GRAC/LigandDisplayForward?ligandId=9503 [http://www.ncbi.nlm.nih.gov/pubmed/15128310?dopt=AbstractPlus]
Endogenous substrates	protons, dipeptides [http://www.ncbi.nlm.nih.gov/pubmed/9637710?dopt=AbstractPlus], http://www.guidetopharmacology.org/GRAC/LigandDisplayForward?ligandId=4784 [http://www.ncbi.nlm.nih.gov/pubmed/19789362?dopt=AbstractPlus, http://www.ncbi.nlm.nih.gov/pubmed/15901802?dopt=AbstractPlus, http://www.ncbi.nlm.nih.gov/pubmed/9637710?dopt=AbstractPlus, http://www.ncbi.nlm.nih.gov/pubmed/11454935?dopt=AbstractPlus, http://www.ncbi.nlm.nih.gov/pubmed/12649372?dopt=AbstractPlus, http://www.ncbi.nlm.nih.gov/pubmed/16627568?dopt=AbstractPlus], tripeptides [http://www.ncbi.nlm.nih.gov/pubmed/9637710?dopt=AbstractPlus]	dipeptides, protons, tripeptides, http://www.guidetopharmacology.org/GRAC/LigandDisplayForward?ligandId=4784 [http://www.ncbi.nlm.nih.gov/pubmed/9637710?dopt=AbstractPlus, http://www.ncbi.nlm.nih.gov/pubmed/11454935?dopt=AbstractPlus, http://www.ncbi.nlm.nih.gov/pubmed/17034769?dopt=AbstractPlus]
Stoichiometry	Transport is electrogenic and involves a variable proton‐to‐substrate stoichiometry for uptake of neutral and mono‐ or polyvalently charged peptides, as well as 5‐aminolevulic acid.	Transport is electrogenic and involves a variable proton‐to‐substrate stoichiometry for uptake of neutral and mono‐ or polyvalently charged peptides, as well as 5‐aminolevulic acid.
Inhibitors	http://www.guidetopharmacology.org/GRAC/LigandDisplayForward?ligandId=9486 (p*K*_i_ 5.7) [http://www.ncbi.nlm.nih.gov/pubmed/14706812?dopt=AbstractPlus], http://www.guidetopharmacology.org/GRAC/LigandDisplayForward?ligandId=4702 (p*K*_i_ 5.5) [http://www.ncbi.nlm.nih.gov/pubmed/15930458?dopt=AbstractPlus, http://www.ncbi.nlm.nih.gov/pubmed/9882198?dopt=AbstractPlus], http://www.guidetopharmacology.org/GRAC/LigandDisplayForward?ligandId=4498 (p*K*_i_ 5–5.3) [http://www.ncbi.nlm.nih.gov/pubmed/11284702?dopt=AbstractPlus]	http://www.guidetopharmacology.org/GRAC/LigandDisplayForward?ligandId=4499 (p*K*_i_ 8) [http://www.ncbi.nlm.nih.gov/pubmed/16868651?dopt=AbstractPlus, http://www.ncbi.nlm.nih.gov/pubmed/11752223?dopt=AbstractPlus], http://www.guidetopharmacology.org/GRAC/LigandDisplayForward?ligandId=4498
Labelled ligands	http://www.guidetopharmacology.org/GRAC/LigandDisplayForward?ligandId=4489 [http://www.ncbi.nlm.nih.gov/pubmed/18344442?dopt=AbstractPlus], http://www.guidetopharmacology.org/GRAC/LigandDisplayForward?ligandId=4599 [http://www.ncbi.nlm.nih.gov/pubmed/30135242?dopt=AbstractPlus, http://www.ncbi.nlm.nih.gov/pubmed/9753615?dopt=AbstractPlus, http://www.ncbi.nlm.nih.gov/pubmed/15974593?dopt=AbstractPlus, http://www.ncbi.nlm.nih.gov/pubmed/7592745?dopt=AbstractPlus, http://www.ncbi.nlm.nih.gov/pubmed/9610386?dopt=AbstractPlus, http://www.ncbi.nlm.nih.gov/pubmed/9092716?dopt=AbstractPlus, http://www.ncbi.nlm.nih.gov/pubmed/11454935?dopt=AbstractPlus, http://www.ncbi.nlm.nih.gov/pubmed/18824524?dopt=AbstractPlus, http://www.ncbi.nlm.nih.gov/pubmed/11284702?dopt=AbstractPlus, http://www.ncbi.nlm.nih.gov/pubmed/18713951?dopt=AbstractPlus, http://www.ncbi.nlm.nih.gov/pubmed/15567297?dopt=AbstractPlus, http://www.ncbi.nlm.nih.gov/pubmed/27543355?dopt=AbstractPlus, http://www.ncbi.nlm.nih.gov/pubmed/10578127?dopt=AbstractPlus, http://www.ncbi.nlm.nih.gov/pubmed/9379359?dopt=AbstractPlus, http://www.ncbi.nlm.nih.gov/pubmed/9374833?dopt=AbstractPlus, http://www.ncbi.nlm.nih.gov/pubmed/8843163?dopt=AbstractPlus, http://www.ncbi.nlm.nih.gov/pubmed/10748266?dopt=AbstractPlus], [http://www.guidetopharmacology.org/GRAC/LigandDisplayForward?ligandId=4659]http://www.guidetopharmacology.org/GRAC/LigandDisplayForward?ligandId=4659 [http://www.ncbi.nlm.nih.gov/pubmed/12538834?dopt=AbstractPlus, http://www.ncbi.nlm.nih.gov/pubmed/11714740?dopt=AbstractPlus, http://www.ncbi.nlm.nih.gov/pubmed/11602669?dopt=AbstractPlus, http://www.ncbi.nlm.nih.gov/pubmed/8956326?dopt=AbstractPlus, http://www.ncbi.nlm.nih.gov/pubmed/9706043?dopt=AbstractPlus, http://www.ncbi.nlm.nih.gov/pubmed/20660104?dopt=AbstractPlus, http://www.ncbi.nlm.nih.gov/pubmed/16434549?dopt=AbstractPlus, http://www.ncbi.nlm.nih.gov/pubmed/27903454?dopt=AbstractPlus, http://www.ncbi.nlm.nih.gov/pubmed/16627568?dopt=AbstractPlus, http://www.ncbi.nlm.nih.gov/pubmed/22950754?dopt=AbstractPlus]	http://www.guidetopharmacology.org/GRAC/LigandDisplayForward?ligandId=4489 [http://www.ncbi.nlm.nih.gov/pubmed/15781409?dopt=AbstractPlus], http://www.guidetopharmacology.org/GRAC/LigandDisplayForward?ligandId=4599 [http://www.ncbi.nlm.nih.gov/pubmed/14715149?dopt=AbstractPlus, http://www.ncbi.nlm.nih.gov/pubmed/7592745?dopt=AbstractPlus, http://www.ncbi.nlm.nih.gov/pubmed/9610386?dopt=AbstractPlus, http://www.ncbi.nlm.nih.gov/pubmed/9092716?dopt=AbstractPlus, http://www.ncbi.nlm.nih.gov/pubmed/24548120?dopt=AbstractPlus, http://www.ncbi.nlm.nih.gov/pubmed/11454935?dopt=AbstractPlus, http://www.ncbi.nlm.nih.gov/pubmed/18824524?dopt=AbstractPlus, http://www.ncbi.nlm.nih.gov/pubmed/18713951?dopt=AbstractPlus, http://www.ncbi.nlm.nih.gov/pubmed/7756356?dopt=AbstractPlus, http://www.ncbi.nlm.nih.gov/pubmed/15567297?dopt=AbstractPlus, http://www.ncbi.nlm.nih.gov/pubmed/10578127?dopt=AbstractPlus, http://www.ncbi.nlm.nih.gov/pubmed/9374833?dopt=AbstractPlus, http://www.ncbi.nlm.nih.gov/pubmed/10748266?dopt=AbstractPlus], http://www.guidetopharmacology.org/GRAC/LigandDisplayForward?ligandId=4659 [http://www.ncbi.nlm.nih.gov/pubmed/16434549?dopt=AbstractPlus, http://www.ncbi.nlm.nih.gov/pubmed/26494147?dopt=AbstractPlus, http://www.ncbi.nlm.nih.gov/pubmed/27903454?dopt=AbstractPlus, http://www.ncbi.nlm.nih.gov/pubmed/18367661?dopt=AbstractPlus, http://www.ncbi.nlm.nih.gov/pubmed/27836942?dopt=AbstractPlus, http://www.ncbi.nlm.nih.gov/pubmed/22950754?dopt=AbstractPlus]
Comments	A variety of dipeptides and drugs interact with PEPT1, including http://www.guidetopharmacology.org/GRAC/LigandDisplayForward?ligandId=4559 [http://www.ncbi.nlm.nih.gov/pubmed/15832510?dopt=AbstractPlus], D‐Phe‐Ala [http://www.ncbi.nlm.nih.gov/pubmed/9637710?dopt=AbstractPlus, http://www.ncbi.nlm.nih.gov/pubmed/11284702?dopt=AbstractPlus], D‐Phe‐Gln [http://www.ncbi.nlm.nih.gov/pubmed/19789362?dopt=AbstractPlus], http://www.guidetopharmacology.org/GRAC/LigandDisplayForward?ligandId=4784 [http://www.ncbi.nlm.nih.gov/pubmed/12649372?dopt=AbstractPlus, http://www.ncbi.nlm.nih.gov/pubmed/14600253?dopt=AbstractPlus, http://www.ncbi.nlm.nih.gov/pubmed/16632403?dopt=AbstractPlus], http://www.guidetopharmacology.org/GRAC/LigandDisplayForward?ligandId=6833 [http://www.ncbi.nlm.nih.gov/pubmed/10748266?dopt=AbstractPlus], http://www.guidetopharmacology.org/GRAC/LigandDisplayForward?ligandId=2414 [http://www.ncbi.nlm.nih.gov/pubmed/10578127?dopt=AbstractPlus], http://www.guidetopharmacology.org/GRAC/LigandDisplayForward?ligandId=4824 [http://www.ncbi.nlm.nih.gov/pubmed/11180195?dopt=AbstractPlus, http://www.ncbi.nlm.nih.gov/pubmed/9753615?dopt=AbstractPlus, http://www.ncbi.nlm.nih.gov/pubmed/15901802?dopt=AbstractPlus, http://www.ncbi.nlm.nih.gov/pubmed/10087037?dopt=AbstractPlus], http://www.guidetopharmacology.org/GRAC/LigandDisplayForward?ligandId=4831 [http://www.ncbi.nlm.nih.gov/pubmed/8627565?dopt=AbstractPlus], http://www.guidetopharmacology.org/GRAC/LigandDisplayForward?ligandId=4832 [http://www.ncbi.nlm.nih.gov/pubmed/7592745?dopt=AbstractPlus] and http://www.guidetopharmacology.org/GRAC/LigandDisplayForward?ligandId=4796 (benzylpenicillin) [http://www.ncbi.nlm.nih.gov/pubmed/15901802?dopt=AbstractPlus], as detected using [^3^H] and [^14^C] radio‐labelled probes.	Other high‐affinity (non‐transported) inhibitors: http://www.guidetopharmacology.org/GRAC/LigandDisplayForward?ligandId=9486l (K_i_ 2 *μ*M) [http://www.ncbi.nlm.nih.gov/pubmed/14706812?dopt=AbstractPlus, http://www.ncbi.nlm.nih.gov/pubmed/11284702?dopt=AbstractPlus], http://www.guidetopharmacology.org/GRAC/LigandDisplayForward?ligandId=4498 (K_i_ 3‐7 *μ*M) [http://www.ncbi.nlm.nih.gov/pubmed/14706812?dopt=AbstractPlus, http://www.ncbi.nlm.nih.gov/pubmed/11284702?dopt=AbstractPlus]; other low‐affinity, (non‐transported) inhibitors: http://www.guidetopharmacology.org/GRAC/LigandDisplayForward?ligandId=4702 (Ki 3 mM) [http://www.ncbi.nlm.nih.gov/pubmed/19789362?dopt=AbstractPlus, http://www.ncbi.nlm.nih.gov/pubmed/15930458?dopt=AbstractPlus, http://www.ncbi.nlm.nih.gov/pubmed/9882198?dopt=AbstractPlus]. Like PEPT1, PEPT2 interact with dipeptides and drugs including http://www.guidetopharmacology.org/GRAC/LigandDisplayForward?ligandId=4559 [http://www.ncbi.nlm.nih.gov/pubmed/17034769?dopt=AbstractPlus], D‐Phe‐Ala [http://www.ncbi.nlm.nih.gov/pubmed/9637710?dopt=AbstractPlus, http://www.ncbi.nlm.nih.gov/pubmed/11752223?dopt=AbstractPlus], http://www.guidetopharmacology.org/GRAC/LigandDisplayForward?ligandId=4784 [http://www.ncbi.nlm.nih.gov/pubmed/9637710?dopt=AbstractPlus, http://www.ncbi.nlm.nih.gov/pubmed/11454935?dopt=AbstractPlus, http://www.ncbi.nlm.nih.gov/pubmed/12649372?dopt=AbstractPlus, http://www.ncbi.nlm.nih.gov/pubmed/14600253?dopt=AbstractPlus, http://www.ncbi.nlm.nih.gov/pubmed/16632403?dopt=AbstractPlus], http://www.guidetopharmacology.org/GRAC/LigandDisplayForward?ligandId=6833 [http://www.ncbi.nlm.nih.gov/pubmed/10748266?dopt=AbstractPlus], http://www.guidetopharmacology.org/GRAC/LigandDisplayForward?ligandId=2414 [http://www.ncbi.nlm.nih.gov/pubmed/10578127?dopt=AbstractPlus], http://www.guidetopharmacology.org/GRAC/LigandDisplayForward?ligandId=4831 [http://www.ncbi.nlm.nih.gov/pubmed/14600253?dopt=AbstractPlus], http://www.guidetopharmacology.org/GRAC/LigandDisplayForward?ligandId=4832 [http://www.ncbi.nlm.nih.gov/pubmed/7592745?dopt=AbstractPlus] and http://www.guidetopharmacology.org/GRAC/LigandDisplayForward?ligandId=4796 (benzylpenicillin) [http://www.ncbi.nlm.nih.gov/pubmed/14600253?dopt=AbstractPlus].

**Table:** 

Nomenclature	http://www.guidetopharmacology.org/GRAC/ObjectDisplayForward?objectId=986	http://www.guidetopharmacology.org/GRAC/ObjectDisplayForward?objectId=987
Systematic nomenclature	SLC15A3	SLC15A4
Common abbreviation	PHT2	PHT1
HGNC, UniProt	https://www.genenames.org/data/gene‐symbol‐report/#!/hgnc_id/HGNC:18068, http://www.uniprot.org/uniprot/Q8IY34	https://www.genenames.org/data/gene‐symbol‐report/#!/hgnc_id/HGNC:23090, http://www.uniprot.org/uniprot/Q8N697
Substrates	http://www.guidetopharmacology.org/GRAC/LigandDisplayForward?ligandId=9506 [http://www.ncbi.nlm.nih.gov/pubmed/24695226?dopt=AbstractPlus], http://www.guidetopharmacology.org/GRAC/LigandDisplayForward?ligandId=5024 [http://www.ncbi.nlm.nih.gov/pubmed/24695226?dopt=AbstractPlus, http://www.ncbi.nlm.nih.gov/pubmed/29305856?dopt=AbstractPlus], http://www.guidetopharmacology.org/GRAC/LigandDisplayForward?ligandId=9507 [http://www.ncbi.nlm.nih.gov/pubmed/29305856?dopt=AbstractPlus]	http://www.guidetopharmacology.org/GRAC/LigandDisplayForward?ligandId=9502 [http://www.ncbi.nlm.nih.gov/pubmed/27543355?dopt=AbstractPlus], http://www.guidetopharmacology.org/GRAC/LigandDisplayForward?ligandId=9507 [http://www.ncbi.nlm.nih.gov/pubmed/19570976?dopt=AbstractPlus, http://www.ncbi.nlm.nih.gov/pubmed/21277849?dopt=AbstractPlus, http://www.ncbi.nlm.nih.gov/pubmed/29224352?dopt=AbstractPlus], http://www.guidetopharmacology.org/GRAC/LigandDisplayForward?ligandId=9508 [http://www.ncbi.nlm.nih.gov/pubmed/19570976?dopt=AbstractPlus], http://www.guidetopharmacology.org/GRAC/LigandDisplayForward?ligandId=9505 [http://www.ncbi.nlm.nih.gov/pubmed/16289537?dopt=AbstractPlus, http://www.ncbi.nlm.nih.gov/pubmed/24548120?dopt=AbstractPlus, http://www.ncbi.nlm.nih.gov/pubmed/29224352?dopt=AbstractPlus, http://www.ncbi.nlm.nih.gov/pubmed/22950754?dopt=AbstractPlus], http://www.guidetopharmacology.org/GRAC/LigandDisplayForward?ligandId=5024 [http://www.ncbi.nlm.nih.gov/pubmed/24695226?dopt=AbstractPlus, http://www.ncbi.nlm.nih.gov/pubmed/29224352?dopt=AbstractPlus], http://www.guidetopharmacology.org/GRAC/LigandDisplayForward?ligandId=4824 [http://www.ncbi.nlm.nih.gov/pubmed/16289537?dopt=AbstractPlus], http://www.guidetopharmacology.org/GRAC/LigandDisplayForward?ligandId=9506 [http://www.ncbi.nlm.nih.gov/pubmed/29784761?dopt=AbstractPlus, http://www.ncbi.nlm.nih.gov/pubmed/24695226?dopt=AbstractPlus]
Endogenous substrates	http://www.guidetopharmacology.org/GRAC/LigandDisplayForward?ligandId=3310 [http://www.ncbi.nlm.nih.gov/pubmed/11336635?dopt=AbstractPlus], dipeptides, tripeptides, protons	http://www.guidetopharmacology.org/GRAC/LigandDisplayForward?ligandId=3310 [http://www.ncbi.nlm.nih.gov/pubmed/16289537?dopt=AbstractPlus, http://www.ncbi.nlm.nih.gov/pubmed/25238095?dopt=AbstractPlus, http://www.ncbi.nlm.nih.gov/pubmed/27543355?dopt=AbstractPlus, http://www.ncbi.nlm.nih.gov/pubmed/27845049?dopt=AbstractPlus, http://www.ncbi.nlm.nih.gov/pubmed/9092568?dopt=AbstractPlus], http://www.guidetopharmacology.org/GRAC/LigandDisplayForward?ligandId=4559 [http://www.ncbi.nlm.nih.gov/pubmed/16289537?dopt=AbstractPlus, http://www.ncbi.nlm.nih.gov/pubmed/9092568?dopt=AbstractPlus]
Stoichiometry	PHT2 has not been analyzed systematically with respect to driving force, mode of transport, and substrate specificity. The pH dependence observed for transport of histidine [http://www.ncbi.nlm.nih.gov/pubmed/11336635?dopt=AbstractPlus] and the model peptides used, *i.e*., carnosine [http://www.ncbi.nlm.nih.gov/pubmed/11336635?dopt=AbstractPlus] and histidyl‐leucine [http://www.ncbi.nlm.nih.gov/pubmed/11336635?dopt=AbstractPlus], suggest a similar mode of operation as PEPT1 and PEPT2 proteins.	PHT1 has not been analyzed systematically with respect to driving force, mode of transport, and substrate specificity. The pH dependence observed for transport of histidine [http://www.ncbi.nlm.nih.gov/pubmed/16289537?dopt=AbstractPlus, http://www.ncbi.nlm.nih.gov/pubmed/25238095?dopt=AbstractPlus, http://www.ncbi.nlm.nih.gov/pubmed/27543355?dopt=AbstractPlus, http://www.ncbi.nlm.nih.gov/pubmed/27845049?dopt=AbstractPlus, http://www.ncbi.nlm.nih.gov/pubmed/9092568?dopt=AbstractPlus] and the model peptide used, *i.e*., carnosine [http://www.ncbi.nlm.nih.gov/pubmed/16289537?dopt=AbstractPlus, http://www.ncbi.nlm.nih.gov/pubmed/9092568?dopt=AbstractPlus], suggest a similar mode of operation as PEPT1 and PEPT2 proteins.
Labelled ligands	http://www.guidetopharmacology.org/GRAC/LigandDisplayForward?ligandId=4623 [http://www.ncbi.nlm.nih.gov/pubmed/11336635?dopt=AbstractPlus]	http://www.guidetopharmacology.org/GRAC/LigandDisplayForward?ligandId=4623 (Binding) [http://www.ncbi.nlm.nih.gov/pubmed/27845049?dopt=AbstractPlus, http://www.ncbi.nlm.nih.gov/pubmed/29305823?dopt=AbstractPlus, http://www.ncbi.nlm.nih.gov/pubmed/9092568?dopt=AbstractPlus], http://www.guidetopharmacology.org/GRAC/LigandDisplayForward?ligandId=4670 [http://www.ncbi.nlm.nih.gov/pubmed/16289537?dopt=AbstractPlus, http://www.ncbi.nlm.nih.gov/pubmed/27543355?dopt=AbstractPlus, http://www.ncbi.nlm.nih.gov/pubmed/22950754?dopt=AbstractPlus, http://www.ncbi.nlm.nih.gov/pubmed/27845049?dopt=AbstractPlus]
Comments	PHT2 interacts with [^3^H]carnosine [http://www.ncbi.nlm.nih.gov/pubmed/11336635?dopt=AbstractPlus].	Other PHT1 ligands include [^3^H]histidine [http://www.ncbi.nlm.nih.gov/pubmed/16289537?dopt=AbstractPlus, http://www.ncbi.nlm.nih.gov/pubmed/27543355?dopt=AbstractPlus, http://www.ncbi.nlm.nih.gov/pubmed/22950754?dopt=AbstractPlus, http://www.ncbi.nlm.nih.gov/pubmed/27845049?dopt=AbstractPlus], d3‐L‐histidine [http://www.ncbi.nlm.nih.gov/pubmed/29224352?dopt=AbstractPlus], [^3^H]carnosine [http://www.ncbi.nlm.nih.gov/pubmed/16289537?dopt=AbstractPlus, http://www.ncbi.nlm.nih.gov/pubmed/9092568?dopt=AbstractPlus] [^14^C]GlySar [http://www.ncbi.nlm.nih.gov/pubmed/24548120?dopt=AbstractPlus], [^3^H]GlySar [http://www.ncbi.nlm.nih.gov/pubmed/16289537?dopt=AbstractPlus, http://www.ncbi.nlm.nih.gov/pubmed/22950754?dopt=AbstractPlus] and [^3^H]valacyclovir [http://www.ncbi.nlm.nih.gov/pubmed/16289537?dopt=AbstractPlus].

### Comments

The members of the SLC15 family of peptide transporters are particularly promiscuous in the transport of di/tripeptides, and D‐amino acid containing peptides are also transported. While SLC15A3 and SLC15A4 transport histidine, none of them transport tetrapeptides. In addition, many molecules, among which beta‐lactam antibiotics, angiotensinconverting enzyme inhibitors and sartans, variably interact with the SLC15 family transporters. Known substrates include http://www.guidetopharmacology.org/GRAC/LigandDisplayForward?ligandId=4831, http://www.guidetopharmacology.org/GRAC/LigandDisplayForward?ligandId=4824, http://www.guidetopharmacology.org/GRAC/LigandDisplayForward?ligandId=4784, L‐Dopa prodrugs, gemcitabine prodrugs, floxuridine prodrugs, Maillard reaction products, JBP485, zanamivir, oseltamivir prodrugs, doxorubicin prodrugs, polymyxins, and didanosine prodrugs. Frequently used pharmaceutical excipients such as Tween®20, Tween®80, Solutol ®HS 15 and Cremophor EL®strongly inhibit cellular uptake of Gly‐Sar by SLC15A1 and/or SLC15A2 [http://www.ncbi.nlm.nih.gov/pubmed/27903454?dopt=AbstractPlus]. There is evidence to suggest the existence of a fifth member of this transporter family, https://www.genenames.org/data/gene‐symbol‐report/#!/hgnc_id/HGNC:33455 (http://www.uniprot.org/uniprot/A6NIM6; http://www.ensembl.org/Homo_sapiens/Gene/Summary?g=ENSG00000188991;r=12:16188485‐16277685;t=ENST00000344941), but to date there is no established biological function or reported pharmacology for this protein [http://www.ncbi.nlm.nih.gov/pubmed/21044875?dopt=AbstractPlus].

### Further reading on SLC15 family of peptide transporters

Anderson CM *et al*. (2010) Hijacking solute carriers for proton‐coupled drug transport. *Physiology (Bethesda)* **25**: 364‐77 [https://www.ncbi.nlm.nih.gov/pubmed/21186281?dopt=AbstractPlus]

Brandsch M. (2013) Drug transport via the intestinal peptide transporter PepT1. *Curr Opin Pharmacol* **13**: 881‐7 [https://www.ncbi.nlm.nih.gov/pubmed/24007794?dopt=AbstractPlus]

Brandsch M. (2009) Transport of drugs by proton‐coupled peptide transporters: pearls and pitfalls. *Expert Opin Drug Metab Toxicol* **5**: 887‐905 [https://www.ncbi.nlm.nih.gov/pubmed/19519280?dopt=AbstractPlus]

Fredriksson R *et al*. (2008) The solute carrier (SLC) complement of the human genome: phylogenetic classification reveals four major families. *FEBS Lett*. **582**: 3811‐6 [https://www.ncbi.nlm.nih.gov/pubmed/18948099?dopt=AbstractPlus]

Newstead S. (2015) Molecular insights into proton coupled peptide transport in the PTR family of oligopeptide transporters. *Biochim. Biophys. Acta* **1850**: 488‐499 [https://www.ncbi.nlm.nih.gov/pubmed/24859687?dopt=AbstractPlus]

Newstead S. (2017) Recent advances in understanding proton coupled peptide transport via the POT family. *Curr. Opin. Struct. Biol*. **45**: 17‐24 [https://www.ncbi.nlm.nih.gov/pubmed/27865112?dopt=AbstractPlus]

Newstead S. (2011) Towards a structural understanding of drug and peptide transport within the proton‐dependent oligopeptide transporter (POT) family. *Biochem. Soc. Trans*. **39**: 1353‐8 [https://www.ncbi.nlm.nih.gov/pubmed/21936814?dopt=AbstractPlus]

Smith DE *et al*. (2013) Proton‐coupled oligopeptide transporter family SLC15: physiological, pharmacological and pathological implications. *Mol. Aspects Med*. **34**: 323‐36 [https://www.ncbi.nlm.nih.gov/pubmed/23506874?dopt=AbstractPlus]

Thwaites DT *et al*. (2007) H+‐coupled nutrient, micronutrient and drug transporters in the mammalian small intestine. *Exp. Physiol*. **92**: 603‐19 [https://www.ncbi.nlm.nih.gov/pubmed/17468205?dopt=AbstractPlus]

## http://www.guidetopharmacology.org/GRAC/FamilyDisplayForward?familyId=188

### Overview

Members of the SLC16 family may be divided into subfamilies on the basis of substrate selectivities, particularly lactate (*e.g*. http://www.guidetopharmacology.org/GRAC/LigandDisplayForward?ligandId=2932), http://www.guidetopharmacology.org/GRAC/LigandDisplayForward?ligandId=4809 and ketone bodies, as well as aromatic amino acids. Topology modelling suggests 12 TM domains, with intracellular termini and an extended loop at TM 6/7.

The proton‐coupledmonocarboxylate transporters (monocarboxylate transporters 1, 4, 2 and 3) allowtransport of the products of cellularmetabolism, principally lactate (*e.g*. http://www.guidetopharmacology.org/GRAC/LigandDisplayForward?ligandId=2932) and http://www.guidetopharmacology.org/GRAC/LigandDisplayForward?ligandId=4809.

**Table:** 

Nomenclature	http://www.guidetopharmacology.org/GRAC/ObjectDisplayForward?objectId=988	http://www.guidetopharmacology.org/GRAC/ObjectDisplayForward?objectId=990	http://www.guidetopharmacology.org/GRAC/ObjectDisplayForward?objectId=991	http://www.guidetopharmacology.org/GRAC/ObjectDisplayForward?objectId=989	http://www.guidetopharmacology.org/GRAC/ObjectDisplayForward?objectId=995	http://www.guidetopharmacology.org/GRAC/ObjectDisplayForward?objectId=992	http://www.guidetopharmacology.org/GRAC/ObjectDisplayForward?objectId=993
Systematic nomenclature	SLC16A1	SLC16A7	SLC16A8	SLC16A3	SLC16A5	SLC16A2	SLC16A10
Common abbreviation	MCT1	MCT2	MCT3	MCT4	MCT6	MCT8	TAT1
HGNC, UniProt	https://www.genenames.org/data/gene‐symbol‐report/#!/hgnc_id/HGNC:10922, http://www.uniprot.org/uniprot/P53985	https://www.genenames.org/data/gene‐symbol‐report/#!/hgnc_id/HGNC:10928, http://www.uniprot.org/uniprot/O60669	https://www.genenames.org/data/gene‐symbol‐report/#!/hgnc_id/HGNC:16270, http://www.uniprot.org/uniprot/O95907	https://www.genenames.org/data/gene‐symbol‐report/#!/hgnc_id/HGNC:10924, http://www.uniprot.org/uniprot/O15427	https://www.genenames.org/data/gene‐symbol‐report/#!/hgnc_id/HGNC:10926, http://www.uniprot.org/uniprot/O15375	https://www.genenames.org/data/gene‐symbol‐report/#!/hgnc_id/HGNC:10923, http://www.uniprot.org/uniprot/P36021	https://www.genenames.org/data/gene‐symbol‐report/#!/hgnc_id/HGNC:17027, http://www.uniprot.org/uniprot/Q8TF71
Substrates	http://www.guidetopharmacology.org/GRAC/LigandDisplayForward?ligandId=4711 [http://www.ncbi.nlm.nih.gov/pubmed/16707723?dopt=AbstractPlus]	–	–	–	–	–	–
Endogenous substrates	http://www.guidetopharmacology.org/GRAC/LigandDisplayForward?ligandId=4809, http://www.guidetopharmacology.org/GRAC/LigandDisplayForward?ligandId=2932, http://www.guidetopharmacology.org/GRAC/LigandDisplayForward?ligandId=1593	http://www.guidetopharmacology.org/GRAC/LigandDisplayForward?ligandId=4809, http://www.guidetopharmacology.org/GRAC/LigandDisplayForward?ligandId=2932	http://www.guidetopharmacology.org/GRAC/LigandDisplayForward?ligandId=2932	http://www.guidetopharmacology.org/GRAC/LigandDisplayForward?ligandId=4809, http://www.guidetopharmacology.org/GRAC/LigandDisplayForward?ligandId=2932	–	http://www.guidetopharmacology.org/GRAC/LigandDisplayForward?ligandId=2634 [http://www.ncbi.nlm.nih.gov/pubmed/16887882?dopt=AbstractPlus], http://www.guidetopharmacology.org/GRAC/LigandDisplayForward?ligandId=2635_4_ [http://www.ncbi.nlm.nih.gov/pubmed/16887882?dopt=AbstractPlus]	http://www.guidetopharmacology.org/GRAC/LigandDisplayForward?ligandId=717, http://www.guidetopharmacology.org/GRAC/LigandDisplayForward?ligandId=3313, http://www.guidetopharmacology.org/GRAC/LigandDisplayForward?ligandId=3639, http://www.guidetopharmacology.org/GRAC/LigandDisplayForward?ligandId=4791
Stoichiometry	1 H^+^ : 1 monocarboxylate^‐^ (out)	1 H^+^ : 1 monocarboxylate^‐^ (out)	1 H^+^ : 1 monocarboxylate^‐^ (out)	1 H^+^ : 1 monocarboxylate^‐^ (out)	Unknown	Unknown	Unknown
Inhibitors	http://www.guidetopharmacology.org/GRAC/LigandDisplayForward?ligandId=8813	(Compound 30 is a channel blocker.) (pKi 8.3) [http://www.ncbi.nlm.nih.gov/pubmed/16455256?dopt=AbstractPlus]	http://www.guidetopharmacology.org/GRAC/LigandDisplayForward?ligandId=8818 (pIC_50_ 8) [http://www.ncbi.nlm.nih.gov/pubmed/24095010?dopt=AbstractPlus]	–	–	–	–
Comments	–	–	–	–	MCT6 has been reported to transport http://www.guidetopharmacology.org/GRAC/LigandDisplayForward?ligandId=4837, but not short chain fatty acids [http://www.ncbi.nlm.nih.gov/pubmed/16174808?dopt=AbstractPlus].	–	–

### Comments

MCT1 and MCT2, but not MCT3 and MCT4, are inhibited by CHC, which also inhibits members of the mitochondrial transporter family, http://www.guidetopharmacology.org/GRAC/FamilyDisplayForward?familyId=147.

MCT5‐MCT7, MCT9 and MCT11‐14 are regarded as orphan transporters.

### Further reading on SLC16 family of monocarboxylate transporters

Bernal J *et al*. (2015) Thyroid hormone transporters‐functions and clinical implications. *Nat Rev Endocrinol* **11**: 406‐417 https://www.ncbi.nlm.nih.gov/pubmed/25942657?dopt=AbstractPlus

Halestrap AP. (2013) The SLC16 gene family ‐ structure, role and regulation in health and disease. *Mol. Aspects Med*. **34**: 337‐49 https://www.ncbi.nlm.nih.gov/pubmed/23506875?dopt=AbstractPlus

Jones RS *et al*. (2016) Monocarboxylate Transporters: Therapeutic Targets and Prognostic Factors in Disease. *Clin. Pharmacol. Ther*. **100**: 454‐463 https://www.ncbi.nlm.nih.gov/pubmed/27351344?dopt=AbstractPlus

## http://www.guidetopharmacology.org/GRAC/FamilyDisplayForward?familyId=145

### Overview

The SLC17 family are sometimes referred to as Type I sodium‐phosphate co‐transporters, alongside Type II (http://www.guidetopharmacology.org/GRAC/FamilyDisplayForward?familyId=221) and Type III (http://www.guidetopharmacology.org/GRAC/FamilyDisplayForward?familyId=195) transporters. Within the SLC17 family, however, further subgroups of organic anion transporters may be defined, allowing the accumulation of http://www.guidetopharmacology.org/GRAC/LigandDisplayForward?ligandId=4644 in the endoplasmic reticulum and glutamate (*e.g*. http://www.guidetopharmacology.org/GRAC/LigandDisplayForward?ligandId=1369) or nucleotides in synaptic and secretory vesicles. Topology modelling suggests 12 TM domains.

## http://www.guidetopharmacology.org/GRAC/FamilyDisplayForward?familyId=189

### Overview

Type I sodium‐phosphate co‐transporters are expressed in the kidney and intestine.

**Table:** 

Nomenclature	http://www.guidetopharmacology.org/GRAC/ObjectDisplayForward?objectId=1002	http://www.guidetopharmacology.org/GRAC/ObjectDisplayForward?objectId=1003	http://www.guidetopharmacology.org/GRAC/ObjectDisplayForward?objectId=1004	http://www.guidetopharmacology.org/GRAC/ObjectDisplayForward?objectId=1005
Systematic nomenclature	SLC17A1	SLC17A2	SLC17A3	SLC17A4
Common abbreviation	NPT1	NPT3	NPT4	–
HGNC, UniProt	https://www.genenames.org/data/gene‐symbol‐report/#!/hgnc_id/HGNC:10929, http://www.uniprot.org/uniprot/Q14916	https://www.genenames.org/data/gene‐symbol‐report/#!/hgnc_id/HGNC:10930, http://www.uniprot.org/uniprot/O00624	https://www.genenames.org/data/gene‐symbol‐report/#!/hgnc_id/HGNC:10931, http://www.uniprot.org/uniprot/O00476	https://www.genenames.org/data/gene‐symbol‐report/#!/hgnc_id/HGNC:10932, http://www.uniprot.org/uniprot/Q9Y2C5
Substrates	http://www.guidetopharmacology.org/GRAC/LigandDisplayForward?ligandId=4357 [http://www.ncbi.nlm.nih.gov/pubmed/8643577?dopt=AbstractPlus], http://www.guidetopharmacology.org/GRAC/LigandDisplayForward?ligandId=4796 [http://www.ncbi.nlm.nih.gov/pubmed/8643577?dopt=AbstractPlus], http://www.guidetopharmacology.org/GRAC/LigandDisplayForward?ligandId=2339 [http://www.ncbi.nlm.nih.gov/pubmed/20566650?dopt=AbstractPlus], organic acids [306], http://www.guidetopharmacology.org/GRAC/LigandDisplayForward?ligandId=4731 [http://www.ncbi.nlm.nih.gov/pubmed/20566650?dopt=AbstractPlus], phosphate [http://www.ncbi.nlm.nih.gov/pubmed/20566650?dopt=AbstractPlus]	–	–	–
Stoichiometry	Unknown	Unknown	Unknown	Unknown

## http://www.guidetopharmacology.org/GRAC/FamilyDisplayForward?familyId=190

### Overview

The sialic acid transporter is expressed on both lysosomes and synaptic vesicles, where it appears to allow export of sialic acid and accumulation of acidic amino acids, respectively [446], driven by proton gradients. In lysosomes, degradation of glycoproteins generates amino acids and sugar residues, which are metabolized further following export from the lysosome.

**Table:** 

Nomenclature	http://www.guidetopharmacology.org/GRAC/ObjectDisplayForward?objectId=1006
Systematic nomenclature	SLC17A5
Common abbreviation	AST
HGNC, UniProt	https://www.genenames.org/data/gene‐symbol‐report/#!/hgnc_id/HGNC:10933, http://www.uniprot.org/uniprot/Q9NRA2
Endogenous substrates	http://www.guidetopharmacology.org/GRAC/LigandDisplayForward?ligandId=2932, http://www.guidetopharmacology.org/GRAC/LigandDisplayForward?ligandId=4519 (out), http://www.guidetopharmacology.org/GRAC/LigandDisplayForward?ligandId=1369 (in) [http://www.ncbi.nlm.nih.gov/pubmed/21781115?dopt=AbstractPlus], http://www.guidetopharmacology.org/GRAC/LigandDisplayForward?ligandId=4723, http://www.guidetopharmacology.org/GRAC/LigandDisplayForward?ligandId=3309 [http://www.ncbi.nlm.nih.gov/pubmed/21781115?dopt=AbstractPlus], http://www.guidetopharmacology.org/GRAC/LigandDisplayForward?ligandId=4644
Stoichiometry	1 H^+^ : 1 sialic acid (out)

### Comments

Loss‐of‐function mutations in sialin are associated with Salla disease (http://omim.org/entry/604369), an autosomal recessive neurodegenerative disorder associated with sialic acid storage disease [http://www.ncbi.nlm.nih.gov/pubmed/10581036?dopt=AbstractPlus].

## http://www.guidetopharmacology.org/GRAC/FamilyDisplayForward?familyId=191

### Overview

Vesicular glutamate transporters (VGLUTs) allow accumulation of glutamate into synaptic vesicles, as well as secretory vesicles in endocrine tissues. The roles of VGLUTs in kidney and liver are unclear. These transporters appear to utilize the proton gradient and also express a chloride conductance [http://www.ncbi.nlm.nih.gov/pubmed/10938000?dopt=AbstractPlus].

**Table:** 

Nomenclature	http://www.guidetopharmacology.org/GRAC/ObjectDisplayForward?objectId=1007	http://www.guidetopharmacology.org/GRAC/ObjectDisplayForward?objectId=1008	http://www.guidetopharmacology.org/GRAC/ObjectDisplayForward?objectId=1009
Systematic nomenclature	SLC17A7	SLC17A6	SLC17A8
Common abbreviation	VGLUT1	VGLUT2	VGLUT3
HGNC, UniProt	https://www.genenames.org/data/gene‐symbol‐report/#!/hgnc_id/HGNC:16704, http://www.uniprot.org/uniprot/Q9P2U7	https://www.genenames.org/data/gene‐symbol‐report/#!/hgnc_id/HGNC:16703, http://www.uniprot.org/uniprot/Q9P2U8	https://www.genenames.org/data/gene‐symbol‐report/#!/hgnc_id/HGNC:20151, http://www.uniprot.org/uniprot/Q8NDX2
Endogenous substrates	http://www.guidetopharmacology.org/GRAC/LigandDisplayForward?ligandId=1369 > http://www.guidetopharmacology.org/GRAC/LigandDisplayForward?ligandId=4708	http://www.guidetopharmacology.org/GRAC/LigandDisplayForward?ligandId=1369 > http://www.guidetopharmacology.org/GRAC/LigandDisplayForward?ligandId=4708	http://www.guidetopharmacology.org/GRAC/LigandDisplayForward?ligandId=1369 > http://www.guidetopharmacology.org/GRAC/LigandDisplayForward?ligandId=4708
Stoichiometry	Unknown	Unknown	Unknown

### Comments

Endogenous ketoacids produced during fasting have been proposed to regulate VGLUT function through blocking chloride ion‐mediated allosteric enhancement of transporter function [http://www.ncbi.nlm.nih.gov/pubmed/20920794?dopt=AbstractPlus].

## http://www.guidetopharmacology.org/GRAC/FamilyDisplayForward?familyId=192

### Overview

The vesicular nucleotide transporter is the most recent member of the SLC17 family to have an assigned function. Uptake of http://www.guidetopharmacology.org/GRAC/LigandDisplayForward?ligandId=1713 was independent of pH, but dependent on chloride ions and membrane potential [http://www.ncbi.nlm.nih.gov/pubmed/18375752?dopt=AbstractPlus].

**Table:** 

Nomenclature	http://www.guidetopharmacology.org/GRAC/ObjectDisplayForward?objectId=1010
Systematic nomenclature	SLC17A9
Common abbreviation	VNUT
HGNC, UniProt	https://www.genenames.org/data/gene‐symbol‐report/#!/hgnc_id/HGNC:16192, http://www.uniprot.org/uniprot/Q9BYT1
Endogenous substrates	http://www.guidetopharmacology.org/GRAC/LigandDisplayForward?ligandId=2410 [http://www.ncbi.nlm.nih.gov/pubmed/18375752?dopt=AbstractPlus], http://www.guidetopharmacology.org/GRAC/LigandDisplayForward?ligandId=1742 [http://www.ncbi.nlm.nih.gov/pubmed/18375752?dopt=AbstractPlus], http://www.guidetopharmacology.org/GRAC/LigandDisplayForward?ligandId=1713 [http://www.ncbi.nlm.nih.gov/pubmed/18375752?dopt=AbstractPlus]
Stoichiometry	Unknown
Selective inhibitors	http://www.guidetopharmacology.org/GRAC/LigandDisplayForward?ligandId=9605 (pIC_50_ 7.8) [346]

### Comments

VGLUTs and VNUT can be inhibited by http://www.guidetopharmacology.org/GRAC/LigandDisplayForward?ligandId=4177 and http://www.guidetopharmacology.org/GRAC/LigandDisplayForward?ligandId=4579.

### Further reading on SLC17 phosphate and organic anion transporter family

Moriyama Y *et al*. (2017) Vesicular nucleotide transporter (VNUT): appearance of an actress on the stage of purinergic signaling. *Purinergic Signal*. **13**: 387‐404 https://www.ncbi.nlm.nih.gov/pubmed/28616712?dopt=AbstractPlus

Omote H *et al*. (2016) Structure, Function, and Drug Interactions of Neurotransmitter Transporters in the Postgenomic Era. *Annu. Rev. Pharmacol. Toxicol*. **56**: 385‐402 https://www.ncbi.nlm.nih.gov/pubmed/26514205?dopt=AbstractPlus

Reimer RJ. (2013) SLC17: a functionally diverse family of organic anion transporters. *Mol. Aspects Med*. **34**: 350‐9 https://www.ncbi.nlm.nih.gov/pubmed/23506876?dopt=AbstractPlus

Takamori S. (2016) Vesicular glutamate transporters as anion channels? *Pflugers Arch*. **468**: 513‐8 https://www.ncbi.nlm.nih.gov/pubmed/26577586?dopt=AbstractPlus

## http://www.guidetopharmacology.org/GRAC/FamilyDisplayForward?familyId=193

### Overview

The vesicular amine transporters (VATs) are putative 12 TM domain proteins that function to transport singly positively charged amine neurotransmitters and hormones from the cytoplasm and concentrate them within secretory vesicles. They function as amine/proton antiporters driven by secondary active transport utilizing the proton gradient established by a multi‐subunit http://www.guidetopharmacology.org/GRAC/FamilyDisplayForward?familyId=137#V‐typeATPase that acidifies secretory vesicles (reviewed by [http://www.ncbi.nlm.nih.gov/pubmed/12827358?dopt=AbstractPlus]). The vesicular acetylcholine transporter (VAChT; [http://www.ncbi.nlm.nih.gov/pubmed/8071310?dopt=AbstractPlus]) localizes to cholinergic neurons, but non‐neuronal expression has also been claimed [http://www.ncbi.nlm.nih.gov/pubmed/21482687?dopt=AbstractPlus]. Vesicular monoamine transporter 1 (VMAT1, [http://www.ncbi.nlm.nih.gov/pubmed/8245983?dopt=AbstractPlus]) is mainly expressed in peripheral neuroendocrine cells, but most likely not in the CNS, whereas VMAT2 [http://www.ncbi.nlm.nih.gov/pubmed/8643547?dopt=AbstractPlus] distributes between both central and peripheral sympathetic monoaminergic neurones [http://www.ncbi.nlm.nih.gov/pubmed/21272013?dopt=AbstractPlus]. The vescular polyamine transporter (VPAT) is highly expressed in the lungs and placenta, with moderate expression in brain and testis, and with low expression in heart and skeletal muscle [http://www.ncbi.nlm.nih.gov/pubmed/25355561?dopt=AbstractPlus]. VPAT mediates vesicular accumulation of polyamines in mast cells [http://www.ncbi.nlm.nih.gov/pubmed/28082679?dopt=AbstractPlus].

**Table:** 

Nomenclature	http://www.guidetopharmacology.org/GRAC/ObjectDisplayForward?objectId=1011	http://www.guidetopharmacology.org/GRAC/ObjectDisplayForward?objectId=1012	http://www.guidetopharmacology.org/GRAC/ObjectDisplayForward?objectId=1013
Systematic nomenclature	SLC18A1	SLC18A2	SLC18A3
Common abbreviation	VMAT1	VMAT2	VAChT
HGNC, UniProt	https://www.genenames.org/data/gene‐symbol‐report/#!/hgnc_id/HGNC:10934, http://www.uniprot.org/uniprot/P54219	https://www.genenames.org/data/gene‐symbol‐report/#!/hgnc_id/HGNC:10935, http://www.uniprot.org/uniprot/Q05940	https://www.genenames.org/data/gene‐symbol‐report/#!/hgnc_id/HGNC:10936, http://www.uniprot.org/uniprot/Q16572
Substrates	http://www.guidetopharmacology.org/GRAC/LigandDisplayForward?ligandId=2147 (*K*_i_ 4.7×10^‐5^M) [http://www.ncbi.nlm.nih.gov/pubmed/8643547?dopt=AbstractPlus], http://www.guidetopharmacology.org/GRAC/LigandDisplayForward?ligandId=2144 (*K*_i_ 3.4×10^‐5^M) [http://www.ncbi.nlm.nih.gov/pubmed/8643547?dopt=AbstractPlus], http://www.guidetopharmacology.org/GRAC/LigandDisplayForward?ligandId=4613 (*K*_i_ 3.1×10^‐6^M) [http://www.ncbi.nlm.nih.gov/pubmed/8643547?dopt=AbstractPlus], http://www.guidetopharmacology.org/GRAC/LigandDisplayForward?ligandId=4568 (*K*_i_ 6.9×10^‐5^M) [http://www.ncbi.nlm.nih.gov/pubmed/8643547?dopt=AbstractPlus], http://www.guidetopharmacology.org/GRAC/LigandDisplayForward?ligandId=4574 (*K*_i_ 1.9×10^‐5^M) [http://www.ncbi.nlm.nih.gov/pubmed/8643547?dopt=AbstractPlus]	http://www.guidetopharmacology.org/GRAC/LigandDisplayForward?ligandId=2144 (*K*_i_ 3.7×10^‐6^M) [http://www.ncbi.nlm.nih.gov/pubmed/8643547?dopt=AbstractPlus], http://www.guidetopharmacology.org/GRAC/LigandDisplayForward?ligandId=2147 (*K*_i_ 2.1×10^‐6^M) [http://www.ncbi.nlm.nih.gov/pubmed/8643547?dopt=AbstractPlus], http://www.guidetopharmacology.org/GRAC/LigandDisplayForward?ligandId=4613 (*K*_i_ 5.1×10^‐6^M) [http://www.ncbi.nlm.nih.gov/pubmed/8643547?dopt=AbstractPlus], http://www.guidetopharmacology.org/GRAC/LigandDisplayForward?ligandId=4568 (*K*_i_ 8.9×10^‐6^M) [http://www.ncbi.nlm.nih.gov/pubmed/8643547?dopt=AbstractPlus], MDMA (*K*_i_ 6.9×10^‐6^M) [http://www.ncbi.nlm.nih.gov/pubmed/8643547?dopt=AbstractPlus]	http://www.guidetopharmacology.org/GRAC/LigandDisplayForward?ligandId=4558 [http://www.ncbi.nlm.nih.gov/pubmed/15979764?dopt=AbstractPlus], http://www.guidetopharmacology.org/GRAC/LigandDisplayForward?ligandId=4569 [http://www.ncbi.nlm.nih.gov/pubmed/15979764?dopt=AbstractPlus], http://www.guidetopharmacology.org/GRAC/LigandDisplayForward?ligandId=4652 [http://www.ncbi.nlm.nih.gov/pubmed/15979764?dopt=AbstractPlus], http://www.guidetopharmacology.org/GRAC/LigandDisplayForward?ligandId=4773 [http://www.ncbi.nlm.nih.gov/pubmed/15979764?dopt=AbstractPlus]
Endogenous substrates	http://www.guidetopharmacology.org/GRAC/LigandDisplayForward?ligandId=1204 (*K*_i_ 4.6×10^‐3^M) [http://www.ncbi.nlm.nih.gov/pubmed/8643547?dopt=AbstractPlus], http://www.guidetopharmacology.org/GRAC/LigandDisplayForward?ligandId=5 (*K*_i_ 1.4×10^‐6^M) [http://www.ncbi.nlm.nih.gov/pubmed/8643547?dopt=AbstractPlus], http://www.guidetopharmacology.org/GRAC/LigandDisplayForward?ligandId=940 (*K*_i_ 3.8×10^‐6^M) [http://www.ncbi.nlm.nih.gov/pubmed/8643547?dopt=AbstractPlus], http://www.guidetopharmacology.org/GRAC/LigandDisplayForward?ligandId=505 (*K*_i_ 1.3×10^‐5^M) [http://www.ncbi.nlm.nih.gov/pubmed/8643547?dopt=AbstractPlus], http://www.guidetopharmacology.org/GRAC/LigandDisplayForward?ligandId=479 (*K*_i_ 5.5×10^‐6^M) [http://www.ncbi.nlm.nih.gov/pubmed/8643547?dopt=AbstractPlus]	http://www.guidetopharmacology.org/GRAC/LigandDisplayForward?ligandId=1204 (*K*_i_ 1.4×10^‐4^M) [http://www.ncbi.nlm.nih.gov/pubmed/8643547?dopt=AbstractPlus], http://www.guidetopharmacology.org/GRAC/LigandDisplayForward?ligandId=940 (*K*_i_ 1.4×10^‐6^M) [http://www.ncbi.nlm.nih.gov/pubmed/8643547?dopt=AbstractPlus], http://www.guidetopharmacology.org/GRAC/LigandDisplayForward?ligandId=5 (*K*_i_ 9×10^‐7^M) [http://www.ncbi.nlm.nih.gov/pubmed/8643547?dopt=AbstractPlus], http://www.guidetopharmacology.org/GRAC/LigandDisplayForward?ligandId=505 (*K*_i_ 3.4×10^‐6^M) [http://www.ncbi.nlm.nih.gov/pubmed/8643547?dopt=AbstractPlus], http://www.guidetopharmacology.org/GRAC/LigandDisplayForward?ligandId=479 (*K*_i_ 1.9×10^‐6^M) [http://www.ncbi.nlm.nih.gov/pubmed/8643547?dopt=AbstractPlus]	http://www.guidetopharmacology.org/GRAC/LigandDisplayForward?ligandId=294 (*K*_i_ 7.9×10^‐4^M) [http://www.ncbi.nlm.nih.gov/pubmed/15485505?dopt=AbstractPlus, http://www.ncbi.nlm.nih.gov/pubmed/20225888?dopt=AbstractPlus], choline (*K*_i_ 5×10^‐3^M) [http://www.ncbi.nlm.nih.gov/pubmed/15485505?dopt=AbstractPlus, http://www.ncbi.nlm.nih.gov/pubmed/20225888?dopt=AbstractPlus]
Stoichiometry	1 amine (in): 2H^+^ (out)	1 amine (in): 2H^+^ (out)	1 amine (in): 2H^+^ (out)
Inhibitors	http://www.guidetopharmacology.org/GRAC/LigandDisplayForward?ligandId=4823 (p*K*_i_ 7.5) [http://www.ncbi.nlm.nih.gov/pubmed/8643547?dopt=AbstractPlus], http://www.guidetopharmacology.org/GRAC/LigandDisplayForward?ligandId=88 (p*K*_i_ 5.8) [http://www.ncbi.nlm.nih.gov/pubmed/8643547?dopt=AbstractPlus], http://www.guidetopharmacology.org/GRAC/LigandDisplayForward?ligandId=4834 (p*K*_i_ 4.7) [http://www.ncbi.nlm.nih.gov/pubmed/8643547?dopt=AbstractPlus]	http://www.guidetopharmacology.org/GRAC/LigandDisplayForward?ligandId=4823 (p*K*_i_ 7.9) [http://www.ncbi.nlm.nih.gov/pubmed/8643547?dopt=AbstractPlus], http://www.guidetopharmacology.org/GRAC/LigandDisplayForward?ligandId=4834 (p*K*_i_ 7) [http://www.ncbi.nlm.nih.gov/pubmed/8643547?dopt=AbstractPlus], http://www.guidetopharmacology.org/GRAC/LigandDisplayForward?ligandId=88 (p*K*_i_ 6.3) [http://www.ncbi.nlm.nih.gov/pubmed/8643547?dopt=AbstractPlus]	http://www.guidetopharmacology.org/GRAC/LigandDisplayForward?ligandId=4768 (p*K*_i_ 10.9) [http://www.ncbi.nlm.nih.gov/pubmed/7702637?dopt=AbstractPlus], http://www.guidetopharmacology.org/GRAC/LigandDisplayForward?ligandId=4759 (p*K*_i_ 8.7) [http://www.ncbi.nlm.nih.gov/pubmed/7702637?dopt=AbstractPlus]
Labelled ligands	–	http://www.guidetopharmacology.org/GRAC/LigandDisplayForward?ligandId=4766 (Inhibitor) (p*K*_d_ 8.2) [http://www.ncbi.nlm.nih.gov/pubmed/8910293?dopt=AbstractPlus], http://www.guidetopharmacology.org/GRAC/LigandDisplayForward?ligandId=4764 (Inhibitor) (p*K*_d_ 8.1) [http://www.ncbi.nlm.nih.gov/pubmed/7855735?dopt=AbstractPlus], http://www.guidetopharmacology.org/GRAC/LigandDisplayForward?ligandId=4608 (Inhibitor),http://www.guidetopharmacology.org/GRAC/LigandDisplayForward?ligandId=4763 (Inhibitor) [http://www.ncbi.nlm.nih.gov/pubmed/9325342?dopt=AbstractPlus]	http://www.guidetopharmacology.org/GRAC/LigandDisplayForward?ligandId=4758 (p*K*_d_ 8.4) [http://www.ncbi.nlm.nih.gov/pubmed/8910293?dopt=AbstractPlus], http://www.guidetopharmacology.org/GRAC/LigandDisplayForward?ligandId=4762

### Comments

*p*K_i_ values for endogenous and synthetic substrate inhibitors of human VMAT1 and VMAT2 are for inhibition of http://www.guidetopharmacology.org/GRAC/LigandDisplayForward?ligandId=3248 uptake in transfected and permeabilised CV‐1 cells as detailed by [http://www.ncbi.nlm.nih.gov/pubmed/8643547?dopt=AbstractPlus]. In addition to the monoamines listed in the table, the trace amines http://www.guidetopharmacology.org/GRAC/LigandDisplayForward?ligandId=2150 and http://www.guidetopharmacology.org/GRAC/LigandDisplayForward?ligandId=2144 are probable substrates for VMAT2 [http://www.ncbi.nlm.nih.gov/pubmed/21272013?dopt=AbstractPlus]. Probes listed in the table are those currently employed; additional agents have been synthesized (*e.g*. [http://www.ncbi.nlm.nih.gov/pubmed/19632829?dopt=AbstractPlus]).

### Further reading on SLC18 family of vesicular amine transporters

German CL *et al*. (2015) Regulation of the Dopamine and Vesicular Monoamine Transporters: Pharmacological Targets and Implications for Disease. *Pharmacol. Rev*. **67**: 1005‐24 https://www.ncbi.nlm.nih.gov/pubmed/26408528?dopt=AbstractPlus

Lohr KM *et al*. (2017) Membrane transporters as mediators of synaptic dopamine dynamics: implications for disease. *Eur. J. Neurosci*. **45**: 20‐33 https://www.ncbi.nlm.nih.gov/pubmed/27520881?dopt=AbstractPlus

Omote H *et al*. (2016) Structure, Function, and Drug Interactions of Neurotransmitter Transporters in the Postgenomic Era. *Annu. Rev. Pharmacol. Toxicol*. **56**: 385‐402 https://www.ncbi.nlm.nih.gov/pubmed/26514205?dopt=AbstractPlus

Sitte HH *et al*. (2015) Amphetamines, new psychoactive drugs and the monoamine transporter cycle. *Trends Pharmacol. Sci*. **36**: 41‐50 https://www.ncbi.nlm.nih.gov/pubmed/25542076?dopt=AbstractPlus

Wimalasena K. (2011) Vesicular monoamine transporters: structure‐function, pharmacology, and medicinal chemistry. *Med Res Rev* **31**: 483‐519 https://www.ncbi.nlm.nih.gov/pubmed/20135628?dopt=AbstractPlus

## http://www.guidetopharmacology.org/GRAC/FamilyDisplayForward?familyId=194

### Overview

The B vitamins http://www.guidetopharmacology.org/GRAC/LigandDisplayForward?ligandId=4563 and http://www.guidetopharmacology.org/GRAC/LigandDisplayForward?ligandId=4629 are transported across the cell membrane, particularly in the intestine, kidneys and placenta, using pH differences as driving forces. Topological modelling suggests the transporters have 12 TM domains.

**Table:** 

Nomenclature	http://www.guidetopharmacology.org/GRAC/ObjectDisplayForward?objectId=1014	http://www.guidetopharmacology.org/GRAC/ObjectDisplayForward?objectId=1015	http://www.guidetopharmacology.org/GRAC/ObjectDisplayForward?objectId=1016
Systematic nomenclature	SLC19A1	SLC19A2	SLC19A3
Common abbreviation	FOLT	ThTr1	ThTr2
HGNC, UniProt	https://www.genenames.org/data/gene‐symbol‐report/#!/hgnc_id/HGNC:10937, http://www.uniprot.org/uniprot/P41440	https://www.genenames.org/data/gene‐symbol‐report/#!/hgnc_id/HGNC:10938, http://www.uniprot.org/uniprot/O60779	https://www.genenames.org/data/gene‐symbol‐report/#!/hgnc_id/HGNC:16266, http://www.uniprot.org/uniprot/Q9BZV2
Substrates	N^5^‐formyltetrahydrofolate, http://www.guidetopharmacology.org/GRAC/LigandDisplayForward?ligandId=4816, http://www.guidetopharmacology.org/GRAC/LigandDisplayForward?ligandId=4815, http://www.guidetopharmacology.org/GRAC/LigandDisplayForward?ligandId=4563 [http://www.ncbi.nlm.nih.gov/pubmed/7826387?dopt=AbstractPlus]	–	–
Endogenous substrates	Other tetrahydrofolate‐cofactors, Organic phosphates; in particular, adenine nucleotides, http://www.guidetopharmacology.org/GRAC/LigandDisplayForward?ligandId=4675 [http://www.ncbi.nlm.nih.gov/pubmed/7826387?dopt=AbstractPlus], http://www.guidetopharmacology.org/GRAC/LigandDisplayForward?ligandId=4684 [http://www.ncbi.nlm.nih.gov/pubmed/7826387?dopt=AbstractPlus], http://www.guidetopharmacology.org/GRAC/LigandDisplayForward?ligandId=4580 [http://www.ncbi.nlm.nih.gov/pubmed/11997266?dopt=AbstractPlus]	http://www.guidetopharmacology.org/GRAC/LigandDisplayForward?ligandId=4629	http://www.guidetopharmacology.org/GRAC/LigandDisplayForward?ligandId=4629
Stoichiometry	Folate (in) : organic phosphate (out), precise stoichiometry unknown	A facilitative carrier not known to be coupled to an inorganic or organic ion gradient	A facilitative carrier not known to be coupled to an inorganic or organic ion gradient
Inhibitors	http://www.guidetopharmacology.org/GRAC/LigandDisplayForward?ligandId=8890 (p*K*_i_ 6.6) [http://www.ncbi.nlm.nih.gov/pubmed/15615544?dopt=AbstractPlus], http://www.guidetopharmacology.org/GRAC/LigandDisplayForward?ligandId=4815 (p*K*_i_ 5.3) [http://www.ncbi.nlm.nih.gov/pubmed/15615544?dopt=AbstractPlus]	–	–
Labelled ligands	http://www.guidetopharmacology.org/GRAC/LigandDisplayForward?ligandId=4562 [http://www.ncbi.nlm.nih.gov/pubmed/9525913?dopt=AbstractPlus], http://www.guidetopharmacology.org/GRAC/LigandDisplayForward?ligandId=4674 [http://www.ncbi.nlm.nih.gov/pubmed/9525913?dopt=AbstractPlus]	http://www.guidetopharmacology.org/GRAC/LigandDisplayForward?ligandId=4628 [http://www.ncbi.nlm.nih.gov/pubmed/10542220?dopt=AbstractPlus]	http://www.guidetopharmacology.org/GRAC/LigandDisplayForward?ligandId=4628 [http://www.ncbi.nlm.nih.gov/pubmed/11731220?dopt=AbstractPlus]

### Comments

Loss‐of‐function mutations in ThTr1 underlie thiamine‐responsive megaloblastic anemia syndrome [http://www.ncbi.nlm.nih.gov/pubmed/10391223?dopt=AbstractPlus].

### Further reading on SLC19 family of vitamin transporters

Matherly LH *et al*. (2014) The major facilitative folate transporters solute carrier 19A1 and solute carrier 46A1: biology and role in antifolate chemotherapy of cancer. *Drug Metab. Dispos*. **42**: 632‐49 https://www.ncbi.nlm.nih.gov/pubmed/24396145?dopt=AbstractPlus

Zhao R *et al*. (2013) Folate and thiamine transporters mediated by facilitative carriers (SLC19A1‐3 and SLC46A1) and folate receptors. *Mol. Aspects Med*. **34**: 373‐85 https://www.ncbi.nlm.nih.gov/pubmed/23506878?dopt=AbstractPlus

## http://www.guidetopharmacology.org/GRAC/FamilyDisplayForward?familyId=195

### Overview

The SLC20 family is looked upon not only as ion transporters, but also as retroviral receptors. As ion transporters, they are sometimes referred to as Type III sodium‐phosphate co‐transporters, alongside Type I (http://www.guidetopharmacology.org/GRAC/FamilyDisplayForward?familyId=145) and Type II (http://www.guidetopharmacology.org/GRAC/FamilyDisplayForward?familyId=221). PiTs are cell‐surface transporters, composed of ten TM domains with extracellular C‐ and N‐termini. PiT1 is a focus for dietary phosphate and http://www.guidetopharmacology.org/GRAC/FamilyDisplayForward?familyId=90 regulation of parathyroid hormone secretion from the parathyroid gland. PiT2 appears to be involved in intestinal absorption of dietary phosphate.

**Table:** 

Nomenclature	http://www.guidetopharmacology.org/GRAC/ObjectDisplayForward?objectId=1017	http://www.guidetopharmacology.org/GRAC/ObjectDisplayForward?objectId=1018
Systematic nomenclature	SLC20A1	SLC20A2
Common abbreviation	PiT1	PiT2
HGNC, UniProt	https://www.genenames.org/data/gene‐symbol‐report/#!/hgnc_id/HGNC:10946, http://www.uniprot.org/uniprot/Q8WUM9	https://www.genenames.org/data/gene‐symbol‐report/#!/hgnc_id/HGNC:10947, http://www.uniprot.org/uniprot/Q08357
Substrates	AsO_4_^3‐^ [http://www.ncbi.nlm.nih.gov/pubmed/17494632?dopt=AbstractPlus], phosphate [http://www.ncbi.nlm.nih.gov/pubmed/17494632?dopt=AbstractPlus]	phosphate [http://www.ncbi.nlm.nih.gov/pubmed/17494632?dopt=AbstractPlus]
Stoichiometry	>1 Na^+^ : 1 HPO_4_^2‐^ (in)	>1 Na^+^ : 1 HPO_4_^2‐^ (in)

### Further reading on SLC20 family of sodium‐dependent phosphate transporters

Biber J *et al*. (2013) Phosphate transporters and their function. *Annu. Rev. Physiol*. **75**: 535‐50 https://www.ncbi.nlm.nih.gov/pubmed/23398154?dopt=AbstractPlus

Forster IC *et al*. (2013) Phosphate transporters of the SLC20 and SLC34 families. *Mol. Aspects Med*. **34**: 386‐95 https://www.ncbi.nlm.nih.gov/pubmed/23506879?dopt=AbstractPlus

Shobeiri N *et al*. (2014) Phosphate: an old bone molecule but new cardiovascular risk factor. *Br J Clin Pharmacol* **77**: 39‐54 https://www.ncbi.nlm.nih.gov/pubmed/23506202?dopt=AbstractPlus

## http://www.guidetopharmacology.org/GRAC/FamilyDisplayForward?familyId=146

### Overview

The SLC22 family of transporters is mostly composed of non‐selective transporters, which are expressed highly in liver, kidney and intestine, playing a major role in drug disposition. The family may be divided into three subfamilies based on the nature of the substrate transported: organic cations (OCTs), organic anions (OATs) and organic zwiterrion/cations (OCTN). Membrane topology is predicted to contain 12 TM domains with intracellular termini, and an extended extracellular loop at TM 1/2.

## http://www.guidetopharmacology.org/GRAC/FamilyDisplayForward?familyId=196

### Overview

Organic cation transporters (OCT) are electrogenic, Na^+^‐independent and reversible.

**Table:** 

Nomenclature	http://www.guidetopharmacology.org/GRAC/ObjectDisplayForward?objectId=1019	http://www.guidetopharmacology.org/GRAC/ObjectDisplayForward?objectId=1020	http://www.guidetopharmacology.org/GRAC/ObjectDisplayForward?objectId=1021
Systematic nomenclature	SLC22A1	SLC22A2	SLC22A3
Common abbreviation	OCT1	OCT2	OCT3
HGNC, UniProt	https://www.genenames.org/data/gene‐symbol‐report/#!/hgnc_id/HGNC:10963, http://www.uniprot.org/uniprot/O15245	https://www.genenames.org/data/gene‐symbol‐report/#!/hgnc_id/HGNC:10966, http://www.uniprot.org/uniprot/O15244	https://www.genenames.org/data/gene‐symbol‐report/#!/hgnc_id/HGNC:10967, http://www.uniprot.org/uniprot/O75751
Substrates	http://www.guidetopharmacology.org/GRAC/LigandDisplayForward?ligandId=4568, http://www.guidetopharmacology.org/GRAC/LigandDisplayForward?ligandId=2343, http://www.guidetopharmacology.org/GRAC/LigandDisplayForward?ligandId=2399, http://www.guidetopharmacology.org/GRAC/LigandDisplayForward?ligandId=4779 [http://www.ncbi.nlm.nih.gov/pubmed/17476361?dopt=AbstractPlus], http://www.guidetopharmacology.org/GRAC/LigandDisplayForward?ligandId=4829	http://www.guidetopharmacology.org/GRAC/LigandDisplayForward?ligandId=4568 [http://www.ncbi.nlm.nih.gov/pubmed/9260930?dopt=AbstractPlus], http://www.guidetopharmacology.org/GRAC/LigandDisplayForward?ligandId=4001 [http://www.ncbi.nlm.nih.gov/pubmed/9260930?dopt=AbstractPlus], http://www.guidetopharmacology.org/GRAC/LigandDisplayForward?ligandId=2343 [http://www.ncbi.nlm.nih.gov/pubmed/9260930?dopt=AbstractPlus], http://www.guidetopharmacology.org/GRAC/LigandDisplayForward?ligandId=2294 [http://www.ncbi.nlm.nih.gov/pubmed/9260930?dopt=AbstractPlus], http://www.guidetopharmacology.org/GRAC/LigandDisplayForward?ligandId=5343 [http://www.ncbi.nlm.nih.gov/pubmed/23506881?dopt=AbstractPlus], http://www.guidetopharmacology.org/GRAC/LigandDisplayForward?ligandId=4779 [http://www.ncbi.nlm.nih.gov/pubmed/23506881?dopt=AbstractPlus]	http://www.guidetopharmacology.org/GRAC/LigandDisplayForward?ligandId=4568, http://www.guidetopharmacology.org/GRAC/LigandDisplayForward?ligandId=2343, http://www.guidetopharmacology.org/GRAC/LigandDisplayForward?ligandId=2342, http://www.guidetopharmacology.org/GRAC/LigandDisplayForward?ligandId=4779 [http://www.ncbi.nlm.nih.gov/pubmed/23506881?dopt=AbstractPlus]
Endogenous substrates	http://www.guidetopharmacology.org/GRAC/LigandDisplayForward?ligandId=1884, http://www.guidetopharmacology.org/GRAC/LigandDisplayForward?ligandId=4551, http://www.guidetopharmacology.org/GRAC/LigandDisplayForward?ligandId=1883, http://www.guidetopharmacology.org/GRAC/LigandDisplayForward?ligandId=5	http://www.guidetopharmacology.org/GRAC/LigandDisplayForward?ligandId=1883 [http://www.ncbi.nlm.nih.gov/pubmed/11907186?dopt=AbstractPlus], http://www.guidetopharmacology.org/GRAC/LigandDisplayForward?ligandId=940 [http://www.ncbi.nlm.nih.gov/pubmed/10385678?dopt=AbstractPlus], http://www.guidetopharmacology.org/GRAC/LigandDisplayForward?ligandId=1204 [http://www.ncbi.nlm.nih.gov/pubmed/10385678?dopt=AbstractPlus]	http://www.guidetopharmacology.org/GRAC/LigandDisplayForward?ligandId=505 [http://www.ncbi.nlm.nih.gov/pubmed/20402963?dopt=AbstractPlus], http://www.guidetopharmacology.org/GRAC/LigandDisplayForward?ligandId=5 [http://www.ncbi.nlm.nih.gov/pubmed/20402963?dopt=AbstractPlus], http://www.guidetopharmacology.org/GRAC/LigandDisplayForward?ligandId=940 [http://www.ncbi.nlm.nih.gov/pubmed/20402963?dopt=AbstractPlus]
Stoichiometry	Unknown	Unknown	Unknown
Inhibitors	http://www.guidetopharmacology.org/GRAC/LigandDisplayForward?ligandId=516 (p*K*_i_ 6.3) [http://www.ncbi.nlm.nih.gov/pubmed/9655880?dopt=AbstractPlus]	http://www.guidetopharmacology.org/GRAC/LigandDisplayForward?ligandId=8482 (p*K*_i_ 7) [http://www.ncbi.nlm.nih.gov/pubmed/9260930?dopt=AbstractPlus]	http://www.guidetopharmacology.org/GRAC/LigandDisplayForward?ligandId=8826 (p*K*_i_ 7.8) [http://www.ncbi.nlm.nih.gov/pubmed/10196521?dopt=AbstractPlus]

### Comments

http://www.guidetopharmacology.org/GRAC/LigandDisplayForward?ligandId=2869 and http://www.guidetopharmacology.org/GRAC/LigandDisplayForward?ligandId=2510 are able to inhibit all three organic cation transporters.

### Further reading on Organic cation transporters (OCT)

Koepsell H. (2013) The SLC22 family with transporters of organic cations, anions and zwitterions. *Mol. Aspects Med*. **34**: 413‐35 https://www.ncbi.nlm.nih.gov/pubmed/23506881?dopt=AbstractPlus

Lozano E *et al*. (2013) Role of the plasma membrane transporter of organic cations OCT1 and its genetic variants in modern liver pharmacology. *Biomed Res Int* **2013**: 692071 https://www.ncbi.nlm.nih.gov/pubmed/23984399?dopt=AbstractPlus

Pelis RM *et al*. (2014) SLC22, SLC44, and SLC47 transporters–organic anion and cation transporters: molecular and cellular properties. *Curr Top Membr* **73**: 233‐61 https://www.ncbi.nlm.nih.gov/pubmed/24745985?dopt=AbstractPlus

Yin J *et al*. (2016) Renal drug transporters and their significance in drug‐drug interactions. *Acta Pharm Sin B* **6**: 363‐373 https://www.ncbi.nlm.nih.gov/pubmed/27709005?dopt=AbstractPlus

## http://www.guidetopharmacology.org/GRAC/FamilyDisplayForward?familyId=197

### Overview

Organic zwitterions/cation transporters (OCTN) function as organic cation uniporters, organic cation/proton exchangers or sodium/http://www.guidetopharmacology.org/GRAC/LigandDisplayForward?ligandId=4780 co‐transporters.

**Table:** 

Nomenclature	http://www.guidetopharmacology.org/GRAC/ObjectDisplayForward?objectId=1022	http://www.guidetopharmacology.org/GRAC/ObjectDisplayForward?objectId=1023	http://www.guidetopharmacology.org/GRAC/ObjectDisplayForward?objectId=1024
Systematic nomenclature	SLC22A4	SLC22A5	SLC22A16
Common abbreviation	OCTN1	OCTN2	CT2
HGNC, UniProt	https://www.genenames.org/data/gene‐symbol‐report/#!/hgnc_id/HGNC:10968, http://www.uniprot.org/uniprot/Q9H015	https://www.genenames.org/data/gene‐symbol‐report/#!/hgnc_id/HGNC:10969, http://www.uniprot.org/uniprot/O76082	https://www.genenames.org/data/gene‐symbol‐report/#!/hgnc_id/HGNC:20302, http://www.uniprot.org/uniprot/Q86VW1
Substrates	http://www.guidetopharmacology.org/GRAC/LigandDisplayForward?ligandId=2406, http://www.guidetopharmacology.org/GRAC/LigandDisplayForward?ligandId=1227, http://www.guidetopharmacology.org/GRAC/LigandDisplayForward?ligandId=2343, http://www.guidetopharmacology.org/GRAC/LigandDisplayForward?ligandId=4568	http://www.guidetopharmacology.org/GRAC/LigandDisplayForward?ligandId=2406, http://www.guidetopharmacology.org/GRAC/LigandDisplayForward?ligandId=2343, http://www.guidetopharmacology.org/GRAC/LigandDisplayForward?ligandId=4568, http://www.guidetopharmacology.org/GRAC/LigandDisplayForward?ligandId=1227	–
Endogenous substrates	http://www.guidetopharmacology.org/GRAC/LigandDisplayForward?ligandId=4780	http://www.guidetopharmacology.org/GRAC/LigandDisplayForward?ligandId=4780, http://www.guidetopharmacology.org/GRAC/LigandDisplayForward?ligandId=4520	http://www.guidetopharmacology.org/GRAC/LigandDisplayForward?ligandId=4780
Stoichiometry	Unknown	Unknown	Unknown

### Comments

Mutations in the SLC22A5 gene lead to primary carnitine deficiency [http://www.ncbi.nlm.nih.gov/pubmed/26828774?dopt=AbstractPlus].

### Further reading on Organic zwitterions/cation transporters (OCTN)

Pochini L *et al*. (2013) OCTN cation transporters in health and disease: role as drug targets and assay development. *J Biomol Screen* **18**: 851‐67 https://www.ncbi.nlm.nih.gov/pubmed/23771822?dopt=AbstractPlus

Tamai I. (2013) Pharmacological and pathophysiological roles of carnitine/organic cation transporters (OCTNs: SLC22A4, SLC22A5 and Slc22a21). *Biopharm Drug Dispos* **34**: 29‐44 https://www.ncbi.nlm.nih.gov/pubmed/22952014?dopt=AbstractPlus

Yin J *et al*. (2016) Renal drug transporters and their significance in drug‐drug interactions. *Acta Pharm Sin B* **6**: 363‐373 https://www.ncbi.nlm.nih.gov/pubmed/27709005?dopt=AbstractPlus

## http://www.guidetopharmacology.org/GRAC/FamilyDisplayForward?familyId=198

### Overview

Organic anion transporters (OATs) are non‐selective transporters prominent in the kidney, placenta and blood‐brain barrier.

**Table:** 

Nomenclature	http://www.guidetopharmacology.org/GRAC/ObjectDisplayForward?objectId=1025	http://www.guidetopharmacology.org/GRAC/ObjectDisplayForward?objectId=1026	http://www.guidetopharmacology.org/GRAC/ObjectDisplayForward?objectId=1027	http://www.guidetopharmacology.org/GRAC/ObjectDisplayForward?objectId=1030	http://www.guidetopharmacology.org/GRAC/ObjectDisplayForward?objectId=1029	http://www.guidetopharmacology.org/GRAC/ObjectDisplayForward?objectId=1028
Systematic nomenclature	SLC22A6	SLC22A7	SLC22A8	SLC22A11	SLC22A10	SLC22A9
Common abbreviation	OAT1	OAT2	OAT3	–	OAT5	OAT4
HGNC, UniProt	https://www.genenames.org/data/gene‐symbol‐report/#!/hgnc_id/HGNC:10970, http://www.uniprot.org/uniprot/Q4U2R8	https://www.genenames.org/data/gene‐symbol‐report/#!/hgnc_id/HGNC:10971, http://www.uniprot.org/uniprot/Q9Y694	https://www.genenames.org/data/gene‐symbol‐report/#!/hgnc_id/HGNC:10972, http://www.uniprot.org/uniprot/Q8TCC7	https://www.genenames.org/data/gene‐symbol‐report/#!/hgnc_id/HGNC:18120, http://www.uniprot.org/uniprot/Q9NSA0	https://www.genenames.org/data/gene‐symbol‐report/#!/hgnc_id/HGNC:18057, http://www.uniprot.org/uniprot/Q63ZE4	https://www.genenames.org/data/gene‐symbol‐report/#!/hgnc_id/HGNC:16261, http://www.uniprot.org/uniprot/Q8IVM8
Substrates	http://www.guidetopharmacology.org/GRAC/LigandDisplayForward?ligandId=4810, non‐steroidal anti‐inflammatory drugs	http://www.guidetopharmacology.org/GRAC/LigandDisplayForward?ligandId=4810, http://www.guidetopharmacology.org/GRAC/LigandDisplayForward?ligandId=1883, non‐steroidal anti‐inflammatory drugs	http://www.guidetopharmacology.org/GRAC/LigandDisplayForward?ligandId=4731 [http://www.ncbi.nlm.nih.gov/pubmed/29847376?dopt=AbstractPlus], http://www.guidetopharmacology.org/GRAC/LigandDisplayForward?ligandId=4749 [http://www.ncbi.nlm.nih.gov/pubmed/10224140?dopt=AbstractPlus], http://www.guidetopharmacology.org/GRAC/LigandDisplayForward?ligandId=4810 [http://www.ncbi.nlm.nih.gov/pubmed/10224140?dopt=AbstractPlus], http://www.guidetopharmacology.org/GRAC/LigandDisplayForward?ligandId=1231 [http://www.ncbi.nlm.nih.gov/pubmed/10224140?dopt=AbstractPlus], http://www.guidetopharmacology.org/GRAC/LigandDisplayForward?ligandId=4672 [http://www.ncbi.nlm.nih.gov/pubmed/10224140?dopt=AbstractPlus]	http://www.guidetopharmacology.org/GRAC/LigandDisplayForward?ligandId=4731 [http://www.ncbi.nlm.nih.gov/pubmed/29847376?dopt=AbstractPlus], http://www.guidetopharmacology.org/GRAC/LigandDisplayForward?ligandId=4528 [http://www.ncbi.nlm.nih.gov/pubmed/10660625?dopt=AbstractPlus], http://www.guidetopharmacology.org/GRAC/LigandDisplayForward?ligandId=4749 [http://www.ncbi.nlm.nih.gov/pubmed/10660625?dopt=AbstractPlus], http://www.guidetopharmacology.org/GRAC/LigandDisplayForward?ligandId=4672 [http://www.ncbi.nlm.nih.gov/pubmed/10660625?dopt=AbstractPlus]	http://www.guidetopharmacology.org/GRAC/LigandDisplayForward?ligandId=4672 [http://www.ncbi.nlm.nih.gov/pubmed/15068970?dopt=AbstractPlus]	–
Stoichiometry	Unknown	Unknown	Unknown	Unknown	Unknown	Unknown
Inhibitors	http://www.guidetopharmacology.org/GRAC/LigandDisplayForward?ligandId=4357 (Inhibition of urate transport by human SCL22A6.) (pIC_50_ 4.9) [http://www.ncbi.nlm.nih.gov/pubmed/12472777?dopt=AbstractPlus]	–	–	–	–	–

### Further reading on Organic anion transporters (OATs)

Burckhardt G *et al*. (2011) In vitro and in vivo evidence of the importance of organic anion transporters (OATs) in drug therapy. *Handb Exp Pharmacol* 29‐104 https://www.ncbi.nlm.nih.gov/pubmed/21103968?dopt=AbstractPlus

Koepsell H. (2013) The SLC22 family with transporters of organic cations, anions and zwitterions. *Mol. Aspects Med*. **34**: 413‐35 https://www.ncbi.nlm.nih.gov/pubmed/23506881?dopt=AbstractPlus

Shen H *et al*. (2017) Organic Anion Transporter 2: An Enigmatic Human Solute Carrier. *Drug Metab. Dispos*. **45**: 228‐236 https://www.ncbi.nlm.nih.gov/pubmed/27872146?dopt=AbstractPlus

Yin J *et al*. (2016) Renal drug transporters and their significance in drug‐drug interactions. *Acta Pharm Sin B* **6**: 363‐373 https://www.ncbi.nlm.nih.gov/pubmed/27709005?dopt=AbstractPlus

## http://www.guidetopharmacology.org/GRAC/FamilyDisplayForward?familyId=199

### Overview

URAT1, a member of the OAT (organic anion transporter) family, is an anion‐exchanging uptake transporter localized to the apical (brush border) membrane of renal proximal tubular cells. It is an anion exchanger that specifically reabsorbs uric acid from the proximal tubule in exchange for monovalent anions such as lactate, nicotinoate, acetoacetate, and hydroxybutyrate [http://www.ncbi.nlm.nih.gov/pubmed/12024214?dopt=AbstractPlus].

**Table:** 

Nomenclature	http://www.guidetopharmacology.org/GRAC/ObjectDisplayForward?objectId=1031
Systematic nomenclature	SLC22A12
Common abbreviation	URAT1
HGNC, UniProt	https://www.genenames.org/data/gene‐symbol‐report/#!/hgnc_id/HGNC:17989, http://www.uniprot.org/uniprot/Q96S37
Endogenous substrates	http://www.guidetopharmacology.org/GRAC/LigandDisplayForward?ligandId=4731 [http://www.ncbi.nlm.nih.gov/pubmed/12024214?dopt=AbstractPlus], http://www.guidetopharmacology.org/GRAC/LigandDisplayForward?ligandId=4690 [http://www.ncbi.nlm.nih.gov/pubmed/12024214?dopt=AbstractPlus]
Stoichiometry	Unknown
Selective inhibitors	http://www.guidetopharmacology.org/GRAC/LigandDisplayForward?ligandId=5826 (pIC_50_ 4) [http://www.ncbi.nlm.nih.gov/pubmed/17325024?dopt=AbstractPlus]
Comments	URAT1 is expressed in the proximal tubule of the kidney and regulates uric acid excretion from the body. Inhibitors of this transporter, such as http://www.guidetopharmacology.org/GRAC/LigandDisplayForward?ligandId=590, find clinical utility in managing hyperuricemia in patients with gout [http://www.ncbi.nlm.nih.gov/pubmed/20719377?dopt=AbstractPlus, http://www.ncbi.nlm.nih.gov/pubmed/18670416?dopt=AbstractPlus].

### Further reading on Urate transporter

Nigam SK *et al*. (2018) The systems biology of uric acid transporters: the role of remote sensing and signaling. *Curr. Opin. Nephrol. Hypertens*. **27**: 305‐313 https://www.ncbi.nlm.nih.gov/pubmed/29847376?dopt=AbstractPlus

## http://www.guidetopharmacology.org/GRAC/FamilyDisplayForward?familyId=859

### Overview

This family of transporters has previously been classified as part of the atypical major facilitator superfamily (MSF) protein superfamily [http://www.ncbi.nlm.nih.gov/pubmed/9529885?dopt=AbstractPlus, http://www.ncbi.nlm.nih.gov/pubmed/28878041?dopt=AbstractPlus, http://www.ncbi.nlm.nih.gov/pubmed/27939446?dopt=AbstractPlus, http://www.ncbi.nlm.nih.gov/pubmed/22458847?dopt=AbstractPlus]. The atypical SLCs share sequence similarities and phylogenetic ancestry with other SLCs, and they have historically been classified in to subfamilies (also referred to as atypical MFS transporter families (AMTF1‐15)) based on phylogenetic, sequence and structural analyses [http://www.ncbi.nlm.nih.gov/pubmed/28878041?dopt=AbstractPlus].

**Table:** 

Nomenclature	http://www.guidetopharmacology.org/GRAC/ObjectDisplayForward?objectId=2634
Systematic nomenclature	SLC22B1
HGNC, UniProt	https://www.genenames.org/data/gene‐symbol‐report/#!/hgnc_id/HGNC:20566, http://www.uniprot.org/uniprot/Q7L0J3
Substrates	Galactose [http://www.ncbi.nlm.nih.gov/pubmed/25326386?dopt=AbstractPlus]
Inhibitors	http://www.guidetopharmacology.org/GRAC/LigandDisplayForward?ligandId=9041 (pIC_50_ 7) [http://www.ncbi.nlm.nih.gov/pubmed/14736235?dopt=AbstractPlus] – Rat, http://www.guidetopharmacology.org/GRAC/LigandDisplayForward?ligandId=6826 (p*K*_i_ 5.8) [http://www.ncbi.nlm.nih.gov/pubmed/8605950?dopt=AbstractPlus] – Rat

### Comments

There are three human synaptic vesicle glycoprotein 2 family members, SV2A, SV2B and SV2C. They have transmembrane transporter activity and can be classified in to the SLC superfamily of solute carriers in subfamily SLC22, as SCL22B1, B2 and B3 respectively. SV2A (SCL22B1) has been identified as the brain binding‐site for the antiepileptic drugs levetiracetam [http://www.ncbi.nlm.nih.gov/pubmed/23484603?dopt=AbstractPlus, http://www.ncbi.nlm.nih.gov/pubmed/27752944?dopt=AbstractPlus] and brivaracetam [http://www.ncbi.nlm.nih.gov/pubmed/26663401?dopt=AbstractPlus].

### Further reading on Atypical SLC22B subfamily

Löscher W *et al*. (2016) Synaptic Vesicle Glycoprotein 2A Ligands in the Treatment of Epilepsy and Beyond. *CNS Drugs* **30**: 1055‐1077 https://www.ncbi.nlm.nih.gov/pubmed/27752944?dopt=AbstractPlus

Mendoza‐Torreblanca JG *et al*. (2013) Synaptic vesicle protein 2A: basic facts and role in synaptic function. *Eur. J. Neurosci*. **38**: 3529‐39 https://www.ncbi.nlm.nih.gov/pubmed/24102679?dopt=AbstractPlus

### Further reading on SLC22 family of organic cation and anion transporters

Burckhardt G. (2012) Drug transport by Organic Anion Transporters (OATs). *Pharmacol. Ther*. **136**: 106‐30 https://www.ncbi.nlm.nih.gov/pubmed/22841915?dopt=AbstractPlus

Hillgren KM *et al*. (2013) Emerging transporters of clinical importance: an update from the International Transporter Consortium. *Clin. Pharmacol. Ther*. **94**: 52‐63 https://www.ncbi.nlm.nih.gov/pubmed/23588305?dopt=AbstractPlus

International Transporter Consortium *et al*. (2010) Membrane transporters in drug development. *Nat Rev Drug Discov* **9**: 215‐36 https://www.ncbi.nlm.nih.gov/pubmed/20190787?dopt=AbstractPlus

Koepsell H. (2013) The SLC22 family with transporters of organic cations, anions and zwitterions. *Mol. Aspects Med*. **34**: 413‐35 https://www.ncbi.nlm.nih.gov/pubmed/23506881?dopt=AbstractPlus

Lozano E *et al*. (2018) Genetic Heterogeneity of SLC22 Family of Transporters in Drug Disposition. *J Pers Med* **8**: https://www.ncbi.nlm.nih.gov/pubmed/29659532?dopt=AbstractPlus

Nigam SK. (2018) The SLC22 Transporter Family: A Paradigm for the Impact of Drug Transporters on Metabolic Pathways, Signaling, and Disease. *Annu. Rev. Pharmacol. Toxicol*. **58**: 663‐687 https://www.ncbi.nlm.nih.gov/pubmed/29309257?dopt=AbstractPlus

Zamek‐Gliszczynski MJ *et al*. (2018) Transporters in Drug Development: 2018 ITC Recommendations for Transporters of Emerging Clinical Importance. *Clin. Pharmacol. Ther*. **104**: 890‐899 https://www.ncbi.nlm.nih.gov/pubmed/30091177?dopt=AbstractPlus

## http://www.guidetopharmacology.org/GRAC/FamilyDisplayForward?familyId=201

### Overview

Predicted to be 12 TM segment proteins, members of this family transport the reduced form of ascorbic acid (while the oxidized form may be handled by members of the http://www.guidetopharmacology.org/GRAC/FamilyDisplayForward?familyId=140 (GLUT1/SLC2A1, GLUT3/SLC2A3 and GLUT4/SLC2A4). http://www.guidetopharmacology.org/GRAC/LigandDisplayForward?ligandId=4285 is considered a non‐selective inhibitor of these transporters, with an affinity in the micromolar range.

**Table:** 

Nomenclature	http://www.guidetopharmacology.org/GRAC/ObjectDisplayForward?objectId=1041	http://www.guidetopharmacology.org/GRAC/ObjectDisplayForward?objectId=1042	http://www.guidetopharmacology.org/GRAC/ObjectDisplayForward?objectId=1043
Systematic nomenclature	SLC23A1	SLC23A2	SLC23A3
Common abbreviation	SVCT1	SVCT2	SVCT3
HGNC, UniProt	https://www.genenames.org/data/gene‐symbol‐report/#!/hgnc_id/HGNC:10974, http://www.uniprot.org/uniprot/Q9UHI7	https://www.genenames.org/data/gene‐symbol‐report/#!/hgnc_id/HGNC:10973, http://www.uniprot.org/uniprot/Q9UGH3	https://www.genenames.org/data/gene‐symbol‐report/#!/hgnc_id/HGNC:20601, http://www.uniprot.org/uniprot/Q6PIS1
Endogenous substrates	http://www.guidetopharmacology.org/GRAC/LigandDisplayForward?ligandId=4781 > http://www.guidetopharmacology.org/GRAC/LigandDisplayForward?ligandId=4651 > http://www.guidetopharmacology.org/GRAC/LigandDisplayForward?ligandId=4733 [http://www.ncbi.nlm.nih.gov/pubmed/10331392?dopt=AbstractPlus]	http://www.guidetopharmacology.org/GRAC/LigandDisplayForward?ligandId=4781 > http://www.guidetopharmacology.org/GRAC/LigandDisplayForward?ligandId=4651 > http://www.guidetopharmacology.org/GRAC/LigandDisplayForward?ligandId=4733 [http://www.ncbi.nlm.nih.gov/pubmed/10331392?dopt=AbstractPlus]	–
Stoichiometry	2 Na^+^: 1 ascorbic acid (in) [http://www.ncbi.nlm.nih.gov/pubmed/10331392?dopt=AbstractPlus]	2 Na^+^: 1 ascorbic acid (in) [http://www.ncbi.nlm.nih.gov/pubmed/10331392?dopt=AbstractPlus]	–
Inhibitors	http://www.guidetopharmacology.org/GRAC/LigandDisplayForward?ligandId=4285 (p*K*_i_ 4.2) [http://www.ncbi.nlm.nih.gov/pubmed/10331392?dopt=AbstractPlus]	–	–
Labelled ligands	http://www.guidetopharmacology.org/GRAC/LigandDisplayForward?ligandId=4532 (Binding) [http://www.ncbi.nlm.nih.gov/pubmed/11895172?dopt=AbstractPlus]	http://www.guidetopharmacology.org/GRAC/LigandDisplayForward?ligandId=4532	–
Comments	–	–	SLC23A3 does not transport ascorbic acid and remains an orphan transporter.

**Table:** 

Nomenclature	http://www.guidetopharmacology.org/GRAC/ObjectDisplayForward?objectId=1044
Systematic nomenclature	SLC23A4
Common abbreviation	SNBT1
HGNC, UniProt	https://www.genenames.org/data/gene‐symbol‐report/#!/hgnc_id/HGNC:20602, –
Substrates	http://www.guidetopharmacology.org/GRAC/LigandDisplayForward?ligandId=4789 [http://www.ncbi.nlm.nih.gov/pubmed/20042597?dopt=AbstractPlus]
Endogenous substrates	http://www.guidetopharmacology.org/GRAC/LigandDisplayForward?ligandId=4560 > http://www.guidetopharmacology.org/GRAC/LigandDisplayForward?ligandId=4581 > http://www.guidetopharmacology.org/GRAC/LigandDisplayForward?ligandId=4556, http://www.guidetopharmacology.org/GRAC/LigandDisplayForward?ligandId=4555 > http://www.guidetopharmacology.org/GRAC/LigandDisplayForward?ligandId=4557, http://www.guidetopharmacology.org/GRAC/LigandDisplayForward?ligandId=4566 [http://www.ncbi.nlm.nih.gov/pubmed/20042597?dopt=AbstractPlus]
Stoichiometry	1 Na^+^ : 1 uracil (in) [http://www.ncbi.nlm.nih.gov/pubmed/20042597?dopt=AbstractPlus]
Comments	SLC23A4/SNBT1 is found in rodents and non‐human primates, but the sequence is truncated in the human genome and named as a pseudogene, SLC23A4P

### Further reading on SLC23 family of ascorbic acid transporters

Bürzle M *et al*. (2013) The sodium‐dependent ascorbic acid transporter family SLC23. *Mol. Aspects Med*. **34**: 436‐54 https://www.ncbi.nlm.nih.gov/pubmed/23506882?dopt=AbstractPlus

May JM. (2011) The SLC23 family of ascorbate transporters: ensuring that you get and keep your daily dose of vitamin *C. Br. J. Pharmacol*. **164**: 1793‐801 https://www.ncbi.nlm.nih.gov/pubmed/21418192?dopt=AbstractPlus

## http://www.guidetopharmacology.org/GRAC/FamilyDisplayForward?familyId=202

### Overview

The sodium/potassium/calcium exchange family of transporters utilize the extracellular sodium gradient to drive calcium and potassium co‐transport out of the cell. As is the case for NCX transporters (http://www.guidetopharmacology.org/GRAC/FamilyDisplayForward?familyId=180), NKCX transporters are thought to be bidirectional, with the possibility of calcium influx following depolarization of the plasma membrane. Topological modeling suggests the presence of 10 TM domains, with a large intracellular loop between the fifth and sixth TM regions.

**Table:** 

Nomenclature	http://www.guidetopharmacology.org/GRAC/ObjectDisplayForward?objectId=1045	http://www.guidetopharmacology.org/GRAC/ObjectDisplayForward?objectId=1050
Systematic nomenclature	SLC24A1	SLC24A6
Common abbreviation	NKCX1	NKCX6
HGNC, UniProt	https://www.genenames.org/data/gene‐symbol‐report/#!/hgnc_id/HGNC:10975, http://www.uniprot.org/uniprot/O60721	https://www.genenames.org/data/gene‐symbol‐report/#!/hgnc_id/HGNC:26175, http://www.uniprot.org/uniprot/Q6J4K2
Stoichiometry	4Na^+^:(1Ca^2+^ + 1K^+^)	–

### Comments

NKCX6 has been proposed to be the sole member of a CAX Na^+^/Ca^2+^ exchanger family, which may be the mitochondrial transporter responsible for calcium accumulation from the cytosol [http://www.ncbi.nlm.nih.gov/pubmed/25998733?dopt=AbstractPlus].

### Further reading on SLC24 family of sodium/potassium/calcium exchangers

Schnetkamp PP. (2013) The SLC24 gene family of Na^+^/Ca^2+^‐K^+^ exchangers: from sight and smell to memory consolidation and skin pigmentation. *Mol. Aspects Med*. **34**: 455‐64 https://www.ncbi.nlm.nih.gov/pubmed/23506883?dopt=AbstractPlus

Schnetkamp PP *et al*. (2014) The SLC24 family of K^+^‐dependent Na^+^‐Ca^2+^ exchangers: structure‐function relationships. *Curr Top Membr* **73**: 263‐87 https://www.ncbi.nlm.nih.gov/pubmed/24745986?dopt=AbstractPlus

Sekler I. (2015) Standing of giants shoulders the story of the mitochondrial Na(+)Ca(2+) exchanger. *Biochem. Biophys. Res. Commun*. **460**: 50‐2 https://www.ncbi.nlm.nih.gov/pubmed/25998733?dopt=AbstractPlus

## http://www.guidetopharmacology.org/GRAC/FamilyDisplayForward?familyId=147

### Overview

Mitochondrial transporters are nuclear‐encoded proteins, which convey solutes across the inner mitochondrial membrane. Topological modelling suggests homodimeric transporters, each with six TM segments and termini in the cytosol.

## http://www.guidetopharmacology.org/GRAC/FamilyDisplayForward?familyId=203

### Overview

Mitochondrial di‐ and tri‐carboxylic acid transporters are grouped on the basis of commonality of substrates and include the citrate transporter which facilitates http://www.guidetopharmacology.org/GRAC/LigandDisplayForward?ligandId=2478 export from the mitochondria to allow the generation of http://www.guidetopharmacology.org/GRAC/LigandDisplayForward?ligandId=5236 and http://www.guidetopharmacology.org/GRAC/LigandDisplayForward?ligandId=3038 through the action of ATP:citrate lyase.

**Table:** 

Nomenclature	http://www.guidetopharmacology.org/GRAC/ObjectDisplayForward?objectId=1051	http://www.guidetopharmacology.org/GRAC/ObjectDisplayForward?objectId=1052	http://www.guidetopharmacology.org/GRAC/ObjectDisplayForward?objectId=1053	http://www.guidetopharmacology.org/GRAC/ObjectDisplayForward?objectId=1057
Systematic nomenclature	SLC25A1	SLC25A10	SLC25A11	SLC25A21
Common abbreviation	CIC	DIC	OGC	ODC
HGNC, UniProt	https://www.genenames.org/data/gene‐symbol‐report/#!/hgnc_id/HGNC:10979, http://www.uniprot.org/uniprot/P53007	https://www.genenames.org/data/gene‐symbol‐report/#!/hgnc_id/HGNC:10980, http://www.uniprot.org/uniprot/Q9UBX3	https://www.genenames.org/data/gene‐symbol‐report/#!/hgnc_id/HGNC:10981, http://www.uniprot.org/uniprot/Q02978	https://www.genenames.org/data/gene‐symbol‐report/#!/hgnc_id/HGNC:14411, http://www.uniprot.org/uniprot/Q9BQT8
Substrates	http://www.guidetopharmacology.org/GRAC/LigandDisplayForward?ligandId=4692, http://www.guidetopharmacology.org/GRAC/LigandDisplayForward?ligandId=2480, http://www.guidetopharmacology.org/GRAC/LigandDisplayForward?ligandId=2478	SO_4_^2‐^, phosphate, S_2_O_3_^2‐^, http://www.guidetopharmacology.org/GRAC/LigandDisplayForward?ligandId=3637, http://www.guidetopharmacology.org/GRAC/LigandDisplayForward?ligandId=2480	http://www.guidetopharmacology.org/GRAC/LigandDisplayForward?ligandId=3636, http://www.guidetopharmacology.org/GRAC/LigandDisplayForward?ligandId=2480	http://www.guidetopharmacology.org/GRAC/LigandDisplayForward?ligandId=3636, http://www.guidetopharmacology.org/GRAC/LigandDisplayForward?ligandId=4657
Stoichiometry	Malate^2‐^ (in) : H‐citrate^2‐^ (out)	PO_3_^4‐^ (in) : malate^2‐^ (out)	Malate^2‐^ (in) : oxoglutarate^2‐^ (out)	Oxoadipate (in) : oxoglutarate (out)
Inhibitors	http://www.guidetopharmacology.org/GRAC/LigandDisplayForward?ligandId=4701	–	–	–

## http://www.guidetopharmacology.org/GRAC/FamilyDisplayForward?familyId=204

### Overview

Mitochondrial amino acid transporters can be subdivided on the basis of their substrates. Mitochondrial ornithine transporters play a role in the http://www.guidetopharmacology.org/GRAC/LigandDisplayForward?ligandId=4539 cycle by exchanging cytosolic ornithine (http://www.guidetopharmacology.org/GRAC/LigandDisplayForward?ligandId=725 and http://www.guidetopharmacology.org/GRAC/LigandDisplayForward?ligandId=4682) for mitochondrial citrulline (http://www.guidetopharmacology.org/GRAC/LigandDisplayForward?ligandId=722 and http://www.guidetopharmacology.org/GRAC/LigandDisplayForward?ligandId=4683) in equimolar amounts. Further members of the family include transporters of S‐adenosylmethionine and carnitine.

**Table:** 

Nomenclature	http://www.guidetopharmacology.org/GRAC/ObjectDisplayForward?objectId=1054	http://www.guidetopharmacology.org/GRAC/ObjectDisplayForward?objectId=1055	http://www.guidetopharmacology.org/GRAC/ObjectDisplayForward?objectId=1056	http://www.guidetopharmacology.org/GRAC/ObjectDisplayForward?objectId=1058	http://www.guidetopharmacology.org/GRAC/ObjectDisplayForward?objectId=1059	http://www.guidetopharmacology.org/GRAC/ObjectDisplayForward?objectId=1060	http://www.guidetopharmacology.org/GRAC/ObjectDisplayForward?objectId=1076
Systematic nomenclature	SLC25A12	SLC25A13	SLC25A18	SLC25A22	SLC25A2	SLC25A15	SLC25A20
Common abbreviation	–	–	GC2	GC1	ORC2	ORC1	CAC
HGNC, UniProt	https://www.genenames.org/data/gene‐symbol‐report/#!/hgnc_id/HGNC:10982, http://www.uniprot.org/uniprot/O75746	https://www.genenames.org/data/gene‐symbol‐report/#!/hgnc_id/HGNC:10983, http://www.uniprot.org/uniprot/Q9UJS0	https://www.genenames.org/data/gene‐symbol‐report/#!/hgnc_id/HGNC:10988, http://www.uniprot.org/uniprot/Q9H1K4	https://www.genenames.org/data/gene‐symbol‐report/#!/hgnc_id/HGNC:19954, http://www.uniprot.org/uniprot/Q9H936	https://www.genenames.org/data/gene‐symbol‐report/#!/hgnc_id/HGNC:22921, http://www.uniprot.org/uniprot/Q9BXI2	https://www.genenames.org/data/gene‐symbol‐report/#!/hgnc_id/HGNC:10985, http://www.uniprot.org/uniprot/Q9Y619	https://www.genenames.org/data/gene‐symbol‐report/#!/hgnc_id/HGNC:1421, http://www.uniprot.org/uniprot/O43772
Substrates	http://www.guidetopharmacology.org/GRAC/LigandDisplayForward?ligandId=1369, http://www.guidetopharmacology.org/GRAC/LigandDisplayForward?ligandId=4695, http://www.guidetopharmacology.org/GRAC/LigandDisplayForward?ligandId=3309	http://www.guidetopharmacology.org/GRAC/LigandDisplayForward?ligandId=4695, http://www.guidetopharmacology.org/GRAC/LigandDisplayForward?ligandId=1369, http://www.guidetopharmacology.org/GRAC/LigandDisplayForward?ligandId=3309	http://www.guidetopharmacology.org/GRAC/LigandDisplayForward?ligandId=1369	http://www.guidetopharmacology.org/GRAC/LigandDisplayForward?ligandId=1369	http://www.guidetopharmacology.org/GRAC/LigandDisplayForward?ligandId=722 [http://www.ncbi.nlm.nih.gov/pubmed/12807890?dopt=AbstractPlus], http://www.guidetopharmacology.org/GRAC/LigandDisplayForward?ligandId=721 [http://www.ncbi.nlm.nih.gov/pubmed/12807890?dopt=AbstractPlus], http://www.guidetopharmacology.org/GRAC/LigandDisplayForward?ligandId=724 [http://www.ncbi.nlm.nih.gov/pubmed/12807890?dopt=AbstractPlus], http://www.guidetopharmacology.org/GRAC/LigandDisplayForward?ligandId=4681 [http://www.ncbi.nlm.nih.gov/pubmed/12807890?dopt=AbstractPlus], http://www.guidetopharmacology.org/GRAC/LigandDisplayForward?ligandId=4680 [http://www.ncbi.nlm.nih.gov/pubmed/12807890?dopt=AbstractPlus], http://www.guidetopharmacology.org/GRAC/LigandDisplayForward?ligandId=4683 [http://www.ncbi.nlm.nih.gov/pubmed/12807890?dopt=AbstractPlus], http://www.guidetopharmacology.org/GRAC/LigandDisplayForward?ligandId=4682 [http://www.ncbi.nlm.nih.gov/pubmed/12807890?dopt=AbstractPlus], http://www.guidetopharmacology.org/GRAC/LigandDisplayForward?ligandId=725 [http://www.ncbi.nlm.nih.gov/pubmed/12807890?dopt=AbstractPlus], http://www.guidetopharmacology.org/GRAC/LigandDisplayForward?ligandId=4679 [http://www.ncbi.nlm.nih.gov/pubmed/12807890?dopt=AbstractPlus], http://www.guidetopharmacology.org/GRAC/LigandDisplayForward?ligandId=3310 [http://www.ncbi.nlm.nih.gov/pubmed/12807890?dopt=AbstractPlus]	http://www.guidetopharmacology.org/GRAC/LigandDisplayForward?ligandId=724 [http://www.ncbi.nlm.nih.gov/pubmed/12807890?dopt=AbstractPlus], http://www.guidetopharmacology.org/GRAC/LigandDisplayForward?ligandId=725 [http://www.ncbi.nlm.nih.gov/pubmed/12807890?dopt=AbstractPlus], http://www.guidetopharmacology.org/GRAC/LigandDisplayForward?ligandId=722 [http://www.ncbi.nlm.nih.gov/pubmed/12807890?dopt=AbstractPlus], http://www.guidetopharmacology.org/GRAC/LigandDisplayForward?ligandId=721 [http://www.ncbi.nlm.nih.gov/pubmed/12807890?dopt=AbstractPlus]	–
Stoichiometry	Aspartate : glutamate H^+^ (bidirectional)	Aspartate : glutamate H^+^ (bidirectional)	Glutamate : H^+^ (bidirectional)	Glutamate : H^+^ (bidirectional)	1 Ornithine (in) :1 citrulline : 1 H^+^ (out)	1 Ornithine (in) :1 citrulline : 1 H^+^ (out)	–
Comments	–	–	–	–	–	–	Exchanges cytosolic acylcarnitine for mitochondrial carnitine

### Comments

Both ornithine transporters are inhibited by the polyamine http://www.guidetopharmacology.org/GRAC/LigandDisplayForward?ligandId=710 [http://www.ncbi.nlm.nih.gov/pubmed/19429682?dopt=AbstractPlus]. Loss‐of‐function mutations in these genes are associated with hyperornithinemia‐hyperammonemia‐homocitrullinuria.

## http://www.guidetopharmacology.org/GRAC/FamilyDisplayForward?familyId=205

### Overview

Mitochondrial phosphate transporters allow the import of inorganic phosphate for http://www.guidetopharmacology.org/GRAC/LigandDisplayForward?ligandId=1713 production.

**Table:** 

Nomenclature	http://www.guidetopharmacology.org/GRAC/ObjectDisplayForward?objectId=1061
Systematic nomenclature	SLC25A3
Common abbreviation	PHC
HGNC, UniProt	https://www.genenames.org/data/gene‐symbol‐report/#!/hgnc_id/HGNC:10989, http://www.uniprot.org/uniprot/Q00325
Stoichiometry	PO_3_^4‐^ (in) : OH^‐^ (out) or PO_3_^4‐^ : H^+^ (in)

## http://www.guidetopharmacology.org/GRAC/FamilyDisplayForward?familyId=206

### Overview

Mitochondrial nucleotide transporters, defined by structural similarlities, include the adenine nucleotide translocator family (SLC25A4, SLC25A5, SLC25A6 and SLC25A31), which under conditions of aerobic metabolism, allow coupling between mitochondrial oxidative phosphorylation and cytosolic energy consumption by exchanging cytosolic http://www.guidetopharmacology.org/GRAC/LigandDisplayForward?ligandId=1712 for mitochondrial http://www.guidetopharmacology.org/GRAC/LigandDisplayForward?ligandId=1713. Further members of the mitochondrial nucleotide transporter subfamily convey diverse substrates including CoA, although not all members have had substrates identified.

**Table:** 

Nomenclature	http://www.guidetopharmacology.org/GRAC/ObjectDisplayForward?objectId=1062	http://www.guidetopharmacology.org/GRAC/ObjectDisplayForward?objectId=1063	http://www.guidetopharmacology.org/GRAC/ObjectDisplayForward?objectId=1064	http://www.guidetopharmacology.org/GRAC/ObjectDisplayForward?objectId=1065	http://www.guidetopharmacology.org/GRAC/ObjectDisplayForward?objectId=1071	http://www.guidetopharmacology.org/GRAC/ObjectDisplayForward?objectId=1072
Systematic nomenclature	SLC25A4	SLC25A5	SLC25A6	SLC25A31	SLC25A16	SLC25A17
Common abbreviation	ANT1	ANT2	ANT3	ANT4	GDC	PMP34
HGNC, UniProt	https://www.genenames.org/data/gene‐symbol‐report/#!/hgnc_id/HGNC:10990, http://www.uniprot.org/uniprot/P12235	https://www.genenames.org/data/gene‐symbol‐report/#!/hgnc_id/HGNC:10991, http://www.uniprot.org/uniprot/P05141	https://www.genenames.org/data/gene‐symbol‐report/#!/hgnc_id/HGNC:10992, http://www.uniprot.org/uniprot/P12236	https://www.genenames.org/data/gene‐symbol‐report/#!/hgnc_id/HGNC:25319, http://www.uniprot.org/uniprot/Q9H0C2	https://www.genenames.org/data/gene‐symbol‐report/#!/hgnc_id/HGNC:10986, http://www.uniprot.org/uniprot/P16260	https://www.genenames.org/data/gene‐symbol‐report/#!/hgnc_id/HGNC:10987, http://www.uniprot.org/uniprot/O43808
Substrates	–	–	–	–	CoA and congeners	http://www.guidetopharmacology.org/GRAC/LigandDisplayForward?ligandId=1712, http://www.guidetopharmacology.org/GRAC/LigandDisplayForward?ligandId=1713, http://www.guidetopharmacology.org/GRAC/LigandDisplayForward?ligandId=2455
Stoichiometry	ADP^3‐^ (in) : ATP^4‐^ (out)	ADP^3‐^ (in) : ATP^4‐^ (out)	ADP^3‐^ (in) : ATP^4‐^ (out)	ADP^3‐^ (in) : ATP^4‐^ (out)	CoA (in)	ATP (in)
Inhibitors	http://www.guidetopharmacology.org/GRAC/LigandDisplayForward?ligandId=4689, http://www.guidetopharmacology.org/GRAC/LigandDisplayForward?ligandId=4572	–	–	–	–	–

**Table:** 

Nomenclature	http://www.guidetopharmacology.org/GRAC/ObjectDisplayForward?objectId=1073	http://www.guidetopharmacology.org/GRAC/ObjectDisplayForward?objectId=1074	http://www.guidetopharmacology.org/GRAC/ObjectDisplayForward?objectId=1077	http://www.guidetopharmacology.org/GRAC/ObjectDisplayForward?objectId=1078	http://www.guidetopharmacology.org/GRAC/ObjectDisplayForward?objectId=1079
Systematic nomenclature	SLC25A19	SLC25A26	SLC25A24	SLC25A23	SLC25A25
Common abbreviation	DNC	SAMC1	APC1	APC2	APC3
HGNC, UniProt	https://www.genenames.org/data/gene‐symbol‐report/#!/hgnc_id/HGNC:14409, http://www.uniprot.org/uniprot/Q9HC21	https://www.genenames.org/data/gene‐symbol‐report/#!/hgnc_id/HGNC:20661, http://www.uniprot.org/uniprot/Q70HW3	https://www.genenames.org/data/gene‐symbol‐report/#!/hgnc_id/HGNC:20662, http://www.uniprot.org/uniprot/Q6NUK1	https://www.genenames.org/data/gene‐symbol‐report/#!/hgnc_id/HGNC:19375, http://www.uniprot.org/uniprot/Q9BV35	https://www.genenames.org/data/gene‐symbol‐report/#!/hgnc_id/HGNC:20663, http://www.uniprot.org/uniprot/Q6KCM7
Substrates	Nucleotide Diphosphates (NDPs), Deoxynucleotide Diphosphates (dNDPs), Dideoxynucleotide Triphosphates (ddNTPs), Deoxynucleotide Triphosphates (dNTPs)	http://www.guidetopharmacology.org/GRAC/LigandDisplayForward?ligandId=4786	–	–	–
Stoichiometry	dNDP (in) : ATP (out)	–	–	–	–

## http://www.guidetopharmacology.org/GRAC/FamilyDisplayForward?familyId=207

### Overview

Mitochondrial uncoupling proteins allow dissipation of the mitochondrial proton gradient associated with thermogenesis and regulation of radical formation.

**Table:** 

Nomenclature	http://www.guidetopharmacology.org/GRAC/ObjectDisplayForward?objectId=1066	http://www.guidetopharmacology.org/GRAC/ObjectDisplayForward?objectId=1067	http://www.guidetopharmacology.org/GRAC/ObjectDisplayForward?objectId=1068	http://www.guidetopharmacology.org/GRAC/ObjectDisplayForward?objectId=1069	http://www.guidetopharmacology.org/GRAC/ObjectDisplayForward?objectId=1070	http://www.guidetopharmacology.org/GRAC/ObjectDisplayForward?objectId=1082
Systematic nomenclature	SLC25A7	SLC25A8	SLC25A9	SLC25A27	SLC25A14	SLC25A30
Common abbreviation	UCP1	UCP2	UCP3	UCP4	UCP5	–
HGNC, UniProt	https://www.genenames.org/data/gene‐symbol‐report/#!/hgnc_id/HGNC:12517, http://www.uniprot.org/uniprot/P25874	https://www.genenames.org/data/gene‐symbol‐report/#!/hgnc_id/HGNC:12518, http://www.uniprot.org/uniprot/P55851	https://www.genenames.org/data/gene‐symbol‐report/#!/hgnc_id/HGNC:12519, http://www.uniprot.org/uniprot/P55916	https://www.genenames.org/data/gene‐symbol‐report/#!/hgnc_id/HGNC:21065, http://www.uniprot.org/uniprot/O95847	https://www.genenames.org/data/gene‐symbol‐report/#!/hgnc_id/HGNC:10984, http://www.uniprot.org/uniprot/O95258	https://www.genenames.org/data/gene‐symbol‐report/#!/hgnc_id/HGNC:27371, http://www.uniprot.org/uniprot/Q5SVS4
Stoichiometry	H^+^ (in)	H^+^ (in)	H^+^ (in)	H^+^ (in)	H^+^ (in)	–

## http://www.guidetopharmacology.org/GRAC/FamilyDisplayForward?familyId=209

### Overview

Many of the transporters identified below have yet to be assigned functions and are currently regarded as orphans.

Information on members of this family may be found in the http://www.guidetopharmacology.org/GRAC/FamilyDisplayForward?familyId=209.

### Further reading on SLC25 family of mitochondrial transporters

Baffy G. (2017) Mitochondrial uncoupling in cancer cells: Liabilities and opportunities. *Biochim. Biophys. Acta* **1858**: 655‐664 https://www.ncbi.nlm.nih.gov/pubmed/28088333?dopt=AbstractPlus

Bertholet AM *et al*. (2017) UCP1: A transporter for H^+^ and fatty acid anions. *Biochimie* **134**: 28‐34 https://www.ncbi.nlm.nih.gov/pubmed/27984203?dopt=AbstractPlus

Clémençon B *et al*. (2013) The mitochondrial ADP/ATP carrier (SLC25 family): pathological implications of its dysfunction. *Mol. Aspects Med*. **34**: 485‐93 https://www.ncbi.nlm.nih.gov/pubmed/23506884?dopt=AbstractPlus

Palmieri F. (2013) The mitochondrial transporter family SLC25: identification, properties and physiopathology. *Mol. Aspects Med*. **34**: 465‐84 https://www.ncbi.nlm.nih.gov/pubmed/23266187?dopt=AbstractPlus

Seifert EL *et al*. (2015) The mitochondrial phosphate carrier: Role in oxidative metabolism, calcium handling and mitochondrial disease. *Biochem. Biophys. Res. Commun*. **464**: 369‐75 https://www.ncbi.nlm.nih.gov/pubmed/26091567?dopt=AbstractPlus

Taylor EB. (2017) Functional Properties of the Mitochondrial Carrier System. *Trends Cell Biol*. **27**: 633‐644 https://www.ncbi.nlm.nih.gov/pubmed/28522206?dopt=AbstractPlus

## http://www.guidetopharmacology.org/GRAC/FamilyDisplayForward?familyId=148

### Overview

Along with the SLC4 family, the SLC26 family acts to allow movement of monovalent and divalent anions across cell membranes. The predicted topology is of 10‐14 TM domains with intracellular C‐ and N‐termini, probably existing as dimers. Within the family, subgroups may be identified on the basis of functional differences, which appear to function as anion exchangers and anion channels (SLC26A7 and SLC26A9).

## http://www.guidetopharmacology.org/GRAC/FamilyDisplayForward?familyId=210

**Table:** 

Nomenclature	http://www.guidetopharmacology.org/GRAC/ObjectDisplayForward?objectId=1097	http://www.guidetopharmacology.org/GRAC/ObjectDisplayForward?objectId=1098
Systematic nomenclature	SLC26A1	SLC26A2
HGNC, UniProt	https://www.genenames.org/data/gene‐symbol‐report/#!/hgnc_id/HGNC:10993, http://www.uniprot.org/uniprot/Q9H2B4	https://www.genenames.org/data/gene‐symbol‐report/#!/hgnc_id/HGNC:10994, http://www.uniprot.org/uniprot/P50443
Substrates	SO_4_^2‐^, http://www.guidetopharmacology.org/GRAC/LigandDisplayForward?ligandId=4538	SO_4_^2‐^
Stoichiometry	SO_4_^2‐^ (in) : anion (out)	1 SO_4_^2‐^ (in) : 2 Cl^‐^ (out)

## http://www.guidetopharmacology.org/GRAC/FamilyDisplayForward?familyId=211

**Table:** 

Nomenclature	http://www.guidetopharmacology.org/GRAC/ObjectDisplayForward?objectId=1099	http://www.guidetopharmacology.org/GRAC/ObjectDisplayForward?objectId=1100	http://www.guidetopharmacology.org/GRAC/ObjectDisplayForward?objectId=1101
Systematic nomenclature	SLC26A3	SLC26A4	SLC26A6
HGNC, UniProt	https://www.genenames.org/data/gene‐symbol‐report/#!/hgnc_id/HGNC:3018, http://www.uniprot.org/uniprot/P40879	https://www.genenames.org/data/gene‐symbol‐report/#!/hgnc_id/HGNC:8818, http://www.uniprot.org/uniprot/O43511	https://www.genenames.org/data/gene‐symbol‐report/#!/hgnc_id/HGNC:14472, http://www.uniprot.org/uniprot/Q9BXS9
Substrates	http://www.guidetopharmacology.org/GRAC/LigandDisplayForward?ligandId=2339	http://www.guidetopharmacology.org/GRAC/LigandDisplayForward?ligandId=4540, HCO_3_^‐^, OH^‐^ , I^‐^ , http://www.guidetopharmacology.org/GRAC/LigandDisplayForward?ligandId=2339	http://www.guidetopharmacology.org/GRAC/LigandDisplayForward?ligandId=4540, http://www.guidetopharmacology.org/GRAC/LigandDisplayForward?ligandId=4538, SO_4_^2‐^, OH^‐^ , http://www.guidetopharmacology.org/GRAC/LigandDisplayForward?ligandId=2339, HCO_3_^‐^, I^‐^
IStoichiometry	2 Cl^‐^ (in) : 1 HCO_3_^‐^ (out) or 2 Cl^‐^ (in) : 1 OH^‐^ (out)	Unknown	1 SO_4_^2‐^ (in) : 2 HCO_3_^‐^ (out) or 1 Cl^‐^ (in) : 2 HCO_3_^‐^ (out)

## http://www.guidetopharmacology.org/GRAC/FamilyDisplayForward?familyId=212

**Table:** 

Nomenclature	http://www.guidetopharmacology.org/GRAC/ObjectDisplayForward?objectId=1102	http://www.guidetopharmacology.org/GRAC/ObjectDisplayForward?objectId=1103
HGNC, UniProt	https://www.genenames.org/data/gene‐symbol‐report/#!/hgnc_id/HGNC:14467, http://www.uniprot.org/uniprot/Q8TE54	https://www.genenames.org/data/gene‐symbol‐report/#!/hgnc_id/HGNC:14469, http://www.uniprot.org/uniprot/Q7LBE3
Substrates	NO_3_^‐^ ≫ http://www.guidetopharmacology.org/GRAC/LigandDisplayForward?ligandId=2339 = Br^‐^ = I^‐^ > SO_4_^2‐^ = http://www.guidetopharmacology.org/GRAC/LigandDisplayForward?ligandId=1369	I^‐^ > Br^‐^ > NO_3_^‐^ > http://www.guidetopharmacology.org/GRAC/LigandDisplayForward?ligandId=2339 > http://www.guidetopharmacology.org/GRAC/LigandDisplayForward?ligandId=1369
Functional Characteristics	Voltage‐ and time‐independent current, linear I‐V relationship [http://www.ncbi.nlm.nih.gov/pubmed/15591059?dopt=AbstractPlus]	Voltage‐ and time‐independent current, linear I‐V relationship [http://www.ncbi.nlm.nih.gov/pubmed/17673510?dopt=AbstractPlus]
Comments	–	SLC26A9 has been suggested to operate in two additional modes as a Cl^‐^‐HCO_3_^‐^exchanger and as a Na^+^‐anion cotransporter [http://www.ncbi.nlm.nih.gov/pubmed/19365592?dopt=AbstractPlus].

## http://www.guidetopharmacology.org/GRAC/FamilyDisplayForward?familyId=213

**Table:** 

Nomenclature	http://www.guidetopharmacology.org/GRAC/ObjectDisplayForward?objectId=1104
Systematic nomenclature	SLC26A5
HGNC, UniProt	https://www.genenames.org/data/gene‐symbol‐report/#!/hgnc_id/HGNC:9359, http://www.uniprot.org/uniprot/P58743
Substrates	HCO_3_^‐^ [http://www.ncbi.nlm.nih.gov/pubmed/22890707?dopt=AbstractPlus], http://www.guidetopharmacology.org/GRAC/LigandDisplayForward?ligandId=2339 [http://www.ncbi.nlm.nih.gov/pubmed/22890707?dopt=AbstractPlus]
Stoichiometry	Unknown
Comments	Prestin has been suggested to function as a molecular motor, rather than a transporter

### Further reading on SLC26 family of anion exchangers

Alper SL *et al*. (2013) The SLC26 gene family of anion transporters and channels. *Mol. Aspects Med*. **34**: 494‐515 https://www.ncbi.nlm.nih.gov/pubmed/23506885?dopt=AbstractPlus

Kato A *et al*. (2011) Regulation of electroneutral NaCl absorption by the small intestine. *Annu. Rev. Physiol*. **73**: 261‐81 https://www.ncbi.nlm.nih.gov/pubmed/21054167?dopt=AbstractPlus

Nofziger C *et al*. (2011) Pendrin function in airway epithelia. *Cell. Physiol. Biochem*. **28**: 571‐8 https://www.ncbi.nlm.nih.gov/pubmed/22116372?dopt=AbstractPlus

Soleimani M. (2013) SLC26 Cl‐/HCO3‐ exchangers in the kidney: roles in health and disease. *Kidney Int*. **84**: 657‐66 https://www.ncbi.nlm.nih.gov/pubmed/23636174?dopt=AbstractPlus

## http://www.guidetopharmacology.org/GRAC/FamilyDisplayForward?familyId=214

### Overview

Fatty acid transporter proteins (FATPs) are a family (SLC27) of six transporters (FATP1‐6). They have at least one, and possibly six [http://www.ncbi.nlm.nih.gov/pubmed/11470793?dopt=AbstractPlus, http://www.ncbi.nlm.nih.gov/pubmed/7954810?dopt=AbstractPlus], transmembrane segments, and are predicted on the basis of structural similarities to form dimers. SLC27 members have several structural domains: integral membrane associated domain, peripheral membrane associated domain, FATP signature, intracellular AMP binding motif, dimerization domain, lipocalin motif, and an ER localization domain (identified in FATP4 only) [http://www.ncbi.nlm.nih.gov/pubmed/9079682?dopt=AbstractPlus, http://www.ncbi.nlm.nih.gov/pubmed/17062637?dopt=AbstractPlus, http://www.ncbi.nlm.nih.gov/pubmed/17065791?dopt=AbstractPlus]. These transporters are unusual in that they appear to express intrinsic very longchain acyl‐CoA synthetase (http://www.genome.jp/kegg‐bin/search_brite?option=‐a&search_string=6.2.1.‐ , http://www.genome.jp/kegg‐bin/search_brite?option=‐a&search_string=6.2.1.7) enzyme activity. Within the cell, these transporters may associate with plasma and peroxisomal membranes. FATP1‐4 and ‐6 transport long‐ and very long‐chain fatty acids, while FATP5 transports long‐chain fatty acids as well as bile acids [http://www.ncbi.nlm.nih.gov/pubmed/11980911?dopt=AbstractPlus, http://www.ncbi.nlm.nih.gov/pubmed/7954810?dopt=AbstractPlus].

**Table:** 

Nomenclature	http://www.guidetopharmacology.org/GRAC/ObjectDisplayForward?objectId=1108	http://www.guidetopharmacology.org/GRAC/ObjectDisplayForward?objectId=1109	http://www.guidetopharmacology.org/GRAC/ObjectDisplayForward?objectId=1110	http://www.guidetopharmacology.org/GRAC/ObjectDisplayForward?objectId=1111	http://www.guidetopharmacology.org/GRAC/ObjectDisplayForward?objectId=1112	http://www.guidetopharmacology.org/GRAC/ObjectDisplayForward?objectId=1113
Systematic nomenclature	SLC27A1	SLC27A2	SLC27A3	SLC27A4	SLC27A5	SLC27A6
Common abbreviation	FATP1	FATP2	FATP3	FATP4	FATP5	FATP6
HGNC, UniProt	https://www.genenames.org/data/gene‐symbol‐report/#!/hgnc_id/HGNC:10995, http://www.uniprot.org/uniprot/Q6PCB7	https://www.genenames.org/data/gene‐symbol‐report/#!/hgnc_id/HGNC:10996, http://www.uniprot.org/uniprot/O14975	https://www.genenames.org/data/gene‐symbol‐report/#!/hgnc_id/HGNC:10997, http://www.uniprot.org/uniprot/Q5K4L6	https://www.genenames.org/data/gene‐symbol‐report/#!/hgnc_id/HGNC:10998, http://www.uniprot.org/uniprot/Q6P1M0	https://www.genenames.org/data/gene‐symbol‐report/#!/hgnc_id/HGNC:10999, http://www.uniprot.org/uniprot/Q9Y2P5	https://www.genenames.org/data/gene‐symbol‐report/#!/hgnc_id/HGNC:11000, http://www.uniprot.org/uniprot/Q9Y2P4
Endogenous substrates	http://www.guidetopharmacology.org/GRAC/LigandDisplayForward?ligandId=1055 > http://www.guidetopharmacology.org/GRAC/LigandDisplayForward?ligandId=1054 > http://www.guidetopharmacology.org/GRAC/LigandDisplayForward?ligandId=4710 > http://www.guidetopharmacology.org/GRAC/LigandDisplayForward?ligandId=4585 [http://www.ncbi.nlm.nih.gov/pubmed/12556534?dopt=AbstractPlus] http://www.guidetopharmacology.org/GRAC/LigandDisplayForward?ligandId=2391 > http://www.guidetopharmacology.org/GRAC/LigandDisplayForward?ligandId=1055 > http://www.guidetopharmacology.org/GRAC/LigandDisplayForward?ligandId=1054 > http://www.guidetopharmacology.org/GRAC/LigandDisplayForward?ligandId=1059 [http://www.ncbi.nlm.nih.gov/pubmed/7954810?dopt=AbstractPlus]	–	–	http://www.guidetopharmacology.org/GRAC/LigandDisplayForward?ligandId=1055, http://www.guidetopharmacology.org/GRAC/LigandDisplayForward?ligandId=1054 > http://www.guidetopharmacology.org/GRAC/LigandDisplayForward?ligandId=4710 > http://www.guidetopharmacology.org/GRAC/LigandDisplayForward?ligandId=4585 [http://www.ncbi.nlm.nih.gov/pubmed/12556534?dopt=AbstractPlus] http://www.guidetopharmacology.org/GRAC/LigandDisplayForward?ligandId=1055 > http://www.guidetopharmacology.org/GRAC/LigandDisplayForward?ligandId=1054 > http://www.guidetopharmacology.org/GRAC/LigandDisplayForward?ligandId=1059, http://www.guidetopharmacology.org/GRAC/LigandDisplayForward?ligandId=4710 > http://www.guidetopharmacology.org/GRAC/LigandDisplayForward?ligandId=2391 [http://www.ncbi.nlm.nih.gov/pubmed/10518211?dopt=AbstractPlus]	–	http://www.guidetopharmacology.org/GRAC/LigandDisplayForward?ligandId=1055 > http://www.guidetopharmacology.org/GRAC/LigandDisplayForward?ligandId=1054 > http://www.guidetopharmacology.org/GRAC/LigandDisplayForward?ligandId=4710 > http://www.guidetopharmacology.org/GRAC/LigandDisplayForward?ligandId=4585 [http://www.ncbi.nlm.nih.gov/pubmed/12556534?dopt=AbstractPlus]
Inhibitors	–	–	–	http://www.guidetopharmacology.org/GRAC/LigandDisplayForward?ligandId=8810 (pIC_50_ 7.1) [http://www.ncbi.nlm.nih.gov/pubmed/16644217?dopt=AbstractPlus]	–	–
Comments	–	–	–	FATP4 is genetically linked to http://www.omim.org/entry/275210?search=275210&highlight=275210.	–	–

### Comments

Although the stoichiometry of fatty acid transport is unclear, it has been proposed to be facilitated by the coupling of fatty acid transport to conjugation with http://www.guidetopharmacology.org/GRAC/LigandDisplayForward?ligandId=3044 to form fatty acyl CoA esters. Small molecule inhibitors of FATP2 [http://www.ncbi.nlm.nih.gov/pubmed/17928635?dopt=AbstractPlus, http://www.ncbi.nlm.nih.gov/pubmed/19913517?dopt=AbstractPlus] and FATP4 [http://www.ncbi.nlm.nih.gov/pubmed/16644217?dopt=AbstractPlus, http://www.ncbi.nlm.nih.gov/pubmed/20448275?dopt=AbstractPlus], as well as bile acid inhibitors of FATP5 [http://www.ncbi.nlm.nih.gov/pubmed/20448275?dopt=AbstractPlus], have been described; analysis of the mechanism of action of some of these inhibitors suggests that transport may be selectively inhibited without altering enzymatic activity of the FATP.

http://www.guidetopharmacology.org/GRAC/LigandDisplayForward?ligandId=5496 accumulation has been used as a non‐selective index of fatty acid transporter activity.

FATP2 has two variants: Variant 1 encodes the full‐length protein, while Variant 2 encodes a shorter isoform missing an internal protein segment. FATP6 also has two variants: Variant 2 encodes the same protein as Variant 1 but has an additional segment in the 5′ UTR.

### Further reading on SLC27 family of fatty acid transporters

Anderson CM *et al*. (2013) SLC27 fatty acid transport proteins. *Mol. Aspects Med*. **34**: 516–28 https://www.ncbi.nlm.nih.gov/pubmed/23506886?dopt=AbstractPlus

Dourlen P *et al*. (2015) Fatty acid transport proteins in disease: New insights from invertebrate models. *Prog. Lipid Res*. **60**: 30–40 https://www.ncbi.nlm.nih.gov/pubmed/26416577?dopt=AbstractPlus

Schwenk RW *et al*. (2010) Fatty acid transport across the cell membrane: regulation by fatty acid transporters. *Prostaglandins Leukot. Essent. Fatty Acids* **82**: 149–54 https://www.ncbi.nlm.nih.gov/pubmed/20206486?dopt=AbstractPlus

## http://www.guidetopharmacology.org/GRAC/FamilyDisplayForward?familyId=149

### Overview

Nucleoside transporters are divided into two families, the sodium‐dependent, concentrative solute carrier family 28 (SLC28) and the equilibrative, solute carrier family 29 (SLC29). The endogenous substrates are typically nucleosides, although some family members can also transport nucleobases and organic cations.

## http://www.guidetopharmacology.org/GRAC/FamilyDisplayForward?familyId=215

### Overview

SLC28 family membersappear to have 13 TM segments with cytoplasmic N‐termini and extracellular C‐termini, and function as concentrative nucleoside transporters.

**Table:** 

Nomenclature	http://www.guidetopharmacology.org/GRAC/ObjectDisplayForward?objectId=1114	http://www.guidetopharmacology.org/GRAC/ObjectDisplayForward?objectId=1115	http://www.guidetopharmacology.org/GRAC/ObjectDisplayForward?objectId=1116
Systematic nomenclature	SLC28A1	SLC28A2	SLC28A3
Common abbreviation	CNT1	CNT2	CNT3
HGNC, UniProt	https://www.genenames.org/data/gene‐symbol‐report/#!/hgnc_id/HGNC:11001, http://www.uniprot.org/uniprot/O00337	https://www.genenames.org/data/gene‐symbol‐report/#!/hgnc_id/HGNC:11002, http://www.uniprot.org/uniprot/O43868	https://www.genenames.org/data/gene‐symbol‐report/#!/hgnc_id/HGNC:16484, http://www.uniprot.org/uniprot/Q9HAS3
Substrates	http://www.guidetopharmacology.org/GRAC/LigandDisplayForward?ligandId=6842 [http://www.ncbi.nlm.nih.gov/pubmed/25011570?dopt=AbstractPlus], http://www.guidetopharmacology.org/GRAC/LigandDisplayForward?ligandId=4793 [http://www.ncbi.nlm.nih.gov/pubmed/22644860?dopt=AbstractPlus], http://www.guidetopharmacology.org/GRAC/LigandDisplayForward?ligandId=4828, http://www.guidetopharmacology.org/GRAC/LigandDisplayForward?ligandId=4825	http://www.guidetopharmacology.org/GRAC/LigandDisplayForward?ligandId=4799 [http://www.ncbi.nlm.nih.gov/pubmed/16840788?dopt=AbstractPlus], http://www.guidetopharmacology.org/GRAC/LigandDisplayForward?ligandId=4833, http://www.guidetopharmacology.org/GRAC/LigandDisplayForward?ligandId=4806, http://www.guidetopharmacology.org/GRAC/LigandDisplayForward?ligandId=4802 [http://www.ncbi.nlm.nih.gov/pubmed/16837649?dopt=AbstractPlus], http://www.guidetopharmacology.org/GRAC/LigandDisplayForward?ligandId=4737 [http://www.ncbi.nlm.nih.gov/pubmed/16837649?dopt=AbstractPlus]	http://www.guidetopharmacology.org/GRAC/LigandDisplayForward?ligandId=4828, http://www.guidetopharmacology.org/GRAC/LigandDisplayForward?ligandId=4737, http://www.guidetopharmacology.org/GRAC/LigandDisplayForward?ligandId=4799, http://www.guidetopharmacology.org/GRAC/LigandDisplayForward?ligandId=4614, http://www.guidetopharmacology.org/GRAC/LigandDisplayForward?ligandId=4801, http://www.guidetopharmacology.org/GRAC/LigandDisplayForward?ligandId=4833, http://www.guidetopharmacology.org/GRAC/LigandDisplayForward?ligandId=4825, http://www.guidetopharmacology.org/GRAC/LigandDisplayForward?ligandId=4729, http://www.guidetopharmacology.org/GRAC/LigandDisplayForward?ligandId=4793
Endogenous substrates	http://www.guidetopharmacology.org/GRAC/LigandDisplayForward?ligandId=2844, http://www.guidetopharmacology.org/GRAC/LigandDisplayForward?ligandId=4566, http://www.guidetopharmacology.org/GRAC/LigandDisplayForward?ligandId=4728, http://www.guidetopharmacology.org/GRAC/LigandDisplayForward?ligandId=4718	http://www.guidetopharmacology.org/GRAC/LigandDisplayForward?ligandId=2844, http://www.guidetopharmacology.org/GRAC/LigandDisplayForward?ligandId=4567, http://www.guidetopharmacology.org/GRAC/LigandDisplayForward?ligandId=4554, http://www.guidetopharmacology.org/GRAC/LigandDisplayForward?ligandId=4718	http://www.guidetopharmacology.org/GRAC/LigandDisplayForward?ligandId=2844, http://www.guidetopharmacology.org/GRAC/LigandDisplayForward?ligandId=4566, http://www.guidetopharmacology.org/GRAC/LigandDisplayForward?ligandId=4567, http://www.guidetopharmacology.org/GRAC/LigandDisplayForward?ligandId=4718, http://www.guidetopharmacology.org/GRAC/LigandDisplayForward?ligandId=4554, http://www.guidetopharmacology.org/GRAC/LigandDisplayForward?ligandId=4728
Stoichiometry	1 Na^+^ : 1 nucleoside (in)	1 Na^+^ : 1 nucleoside (in)	2 Na^+^/H^+^
Inhibitors	–	–	http://www.guidetopharmacology.org/GRAC/LigandDisplayForward?ligandId=8837 (p*K*_i_ 5.5) [http://www.ncbi.nlm.nih.gov/pubmed/19097778?dopt=AbstractPlus]
Comments	–	–	CNT3 forms cyclic homotrimers [http://www.ncbi.nlm.nih.gov/pubmed/28661652?dopt=AbstractPlus]. Genetic variants of *SLC28A3* are associated with increased risk of anthracycline‐induced cardiomyopathy [http://www.ncbi.nlm.nih.gov/pubmed/30351207?dopt=AbstractPlus].

### Further reading on SLC28 family

Johnson ZL *et al*. (2014) Structural basis of nucleoside and nucleoside drug selectivity by concentrative nucleoside transporters. *Elife* **3**: e03604 https://www.ncbi.nlm.nih.gov/pubmed/25082345?dopt=AbstractPlus

Pastor‐Anglada M *et al*. (2008) SLC28 genes and concentrative nucleoside transporter (CNT) proteins. *Xenobiotica* **38**: 972–94 https://www.ncbi.nlm.nih.gov/pubmed/18668436?dopt=AbstractPlus

Pastor‐Anglada M *et al*. (2015) Nucleoside transporter proteins as biomarkers of drug responsiveness and drug targets. *Front Pharmacol* **6**: 13 https://www.ncbi.nlm.nih.gov/pubmed/25713533?dopt=AbstractPlus

Pastor‐Anglada M *et al*. (2018) Who Is Who in Adenosine Transport. *Front Pharmacol* **9**: 627 https://www.ncbi.nlm.nih.gov/pubmed/29962948?dopt=AbstractPlus

Young JD *et al*. (2013) The human concentrative and equilibrative nucleoside transporter families, SLC28 and SLC29. *Mol. Aspects Med*. **34**: 529–47 https://www.ncbi.nlm.nih.gov/pubmed/23506887?dopt=AbstractPlus

## http://www.guidetopharmacology.org/GRAC/FamilyDisplayForward?familyId=216

### Overview

SLC29 family members appear to be composed of 11 TM segments with cytoplasmic N‐termini and extracellular C‐termini. ENT1, ENT2 and ENT4 are cell‐surface transporters, while ENT3 is intracellular, possibly lysosomal [http://www.ncbi.nlm.nih.gov/pubmed/15701636?dopt=AbstractPlus]. ENT1‐3 are described as broad‐spectrum equilibrative nucleoside transporters, while ENT4 is primarily a polyspecific organic cation transporter at neutral pH [http://www.ncbi.nlm.nih.gov/pubmed/21816955?dopt=AbstractPlus].

**Table:** 

Nomenclature	http://www.guidetopharmacology.org/GRAC/ObjectDisplayForward?objectId=1117	http://www.guidetopharmacology.org/GRAC/ObjectDisplayForward?objectId=1118
Systematic nomenclature	SLC29A1	SLC29A2
Common abbreviation	ENT1	ENT2
HGNC, UniProt	https://www.genenames.org/data/gene‐symbol‐report/#!/hgnc_id/HGNC:11003, http://www.uniprot.org/uniprot/Q99808	https://www.genenames.org/data/gene‐symbol‐report/#!/hgnc_id/HGNC:11004, http://www.uniprot.org/uniprot/Q14542
Endogenous substrates in order of increasing Km:	http://www.guidetopharmacology.org/GRAC/LigandDisplayForward?ligandId=2844 < http://www.guidetopharmacology.org/GRAC/LigandDisplayForward?ligandId=4554 < http://www.guidetopharmacology.org/GRAC/LigandDisplayForward?ligandId=4566 < http://www.guidetopharmacology.org/GRAC/LigandDisplayForward?ligandId=4567 < http://www.guidetopharmacology.org/GRAC/LigandDisplayForward?ligandId=4728 < http://www.guidetopharmacology.org/GRAC/LigandDisplayForward?ligandId=4555 < http://www.guidetopharmacology.org/GRAC/LigandDisplayForward?ligandId=4788 < http://www.guidetopharmacology.org/GRAC/LigandDisplayForward?ligandId=4581	–
Substrates	abacavir [http://www.ncbi.nlm.nih.gov/pubmed/30097436?dopt=AbstractPlus], http://www.guidetopharmacology.org/GRAC/LigandDisplayForward?ligandId=548 [http://www.ncbi.nlm.nih.gov/pubmed/28089688?dopt=AbstractPlus], http://www.guidetopharmacology.org/GRAC/LigandDisplayForward?ligandId=4805, http://www.guidetopharmacology.org/GRAC/LigandDisplayForward?ligandId=4806, http://www.guidetopharmacology.org/GRAC/LigandDisplayForward?ligandId=4793, http://www.guidetopharmacology.org/GRAC/LigandDisplayForward?ligandId=372, http://www.guidetopharmacology.org/GRAC/LigandDisplayForward?ligandId=4827, http://www.guidetopharmacology.org/GRAC/LigandDisplayForward?ligandId=4828, http://www.guidetopharmacology.org/GRAC/LigandDisplayForward?ligandId=4833, http://www.guidetopharmacology.org/GRAC/LigandDisplayForward?ligandId=4755, http://www.guidetopharmacology.org/GRAC/LigandDisplayForward?ligandId=4737, http://www.guidetopharmacology.org/GRAC/LigandDisplayForward?ligandId=4799, http://www.guidetopharmacology.org/GRAC/LigandDisplayForward?ligandId=6842 [http://www.ncbi.nlm.nih.gov/pubmed/25011570?dopt=AbstractPlus], http://www.guidetopharmacology.org/GRAC/LigandDisplayForward?ligandId=4801	http://www.guidetopharmacology.org/GRAC/LigandDisplayForward?ligandId=4737, http://www.guidetopharmacology.org/GRAC/LigandDisplayForward?ligandId=372, http://www.guidetopharmacology.org/GRAC/LigandDisplayForward?ligandId=4827, http://www.guidetopharmacology.org/GRAC/LigandDisplayForward?ligandId=4755, http://www.guidetopharmacology.org/GRAC/LigandDisplayForward?ligandId=4799, http://www.guidetopharmacology.org/GRAC/LigandDisplayForward?ligandId=4793, http://www.guidetopharmacology.org/GRAC/LigandDisplayForward?ligandId=4806, http://www.guidetopharmacology.org/GRAC/LigandDisplayForward?ligandId=4825
Endogenous substrates	http://www.guidetopharmacology.org/GRAC/LigandDisplayForward?ligandId=4581 [http://www.ncbi.nlm.nih.gov/pubmed/21795683?dopt=AbstractPlus], http://www.guidetopharmacology.org/GRAC/LigandDisplayForward?ligandId=4567 [http://www.ncbi.nlm.nih.gov/pubmed/21795683?dopt=AbstractPlus], http://www.guidetopharmacology.org/GRAC/LigandDisplayForward?ligandId=4555 [http://www.ncbi.nlm.nih.gov/pubmed/21795683?dopt=AbstractPlus], http://www.guidetopharmacology.org/GRAC/LigandDisplayForward?ligandId=4566 [http://www.ncbi.nlm.nih.gov/pubmed/21795683?dopt=AbstractPlus], http://www.guidetopharmacology.org/GRAC/LigandDisplayForward?ligandId=4554 [http://www.ncbi.nlm.nih.gov/pubmed/21795683?dopt=AbstractPlus], http://www.guidetopharmacology.org/GRAC/LigandDisplayForward?ligandId=4788 [http://www.ncbi.nlm.nih.gov/pubmed/21795683?dopt=AbstractPlus], http://www.guidetopharmacology.org/GRAC/LigandDisplayForward?ligandId=4728 [http://www.ncbi.nlm.nih.gov/pubmed/21795683?dopt=AbstractPlus], http://www.guidetopharmacology.org/GRAC/LigandDisplayForward?ligandId=4718 [http://www.ncbi.nlm.nih.gov/pubmed/21795683?dopt=AbstractPlus], http://www.guidetopharmacology.org/GRAC/LigandDisplayForward?ligandId=2844 [http://www.ncbi.nlm.nih.gov/pubmed/21795683?dopt=AbstractPlus]	http://www.guidetopharmacology.org/GRAC/LigandDisplayForward?ligandId=2844, http://www.guidetopharmacology.org/GRAC/LigandDisplayForward?ligandId=4556, http://www.guidetopharmacology.org/GRAC/LigandDisplayForward?ligandId=4581, http://www.guidetopharmacology.org/GRAC/LigandDisplayForward?ligandId=4566, http://www.guidetopharmacology.org/GRAC/LigandDisplayForward?ligandId=4567, http://www.guidetopharmacology.org/GRAC/LigandDisplayForward?ligandId=4555, http://www.guidetopharmacology.org/GRAC/LigandDisplayForward?ligandId=4554, http://www.guidetopharmacology.org/GRAC/LigandDisplayForward?ligandId=4718, http://www.guidetopharmacology.org/GRAC/LigandDisplayForward?ligandId=8490
Stoichiometry	Equilibrative	Equilibrative
Inhibitors	http://www.guidetopharmacology.org/GRAC/LigandDisplayForward?ligandId=4512 (p*K*_i_ 9.7), http://www.guidetopharmacology.org/GRAC/LigandDisplayForward?ligandId=4590 (p*K*_i_ 9.6) [http://www.ncbi.nlm.nih.gov/pubmed/10763851?dopt=AbstractPlus], http://www.guidetopharmacology.org/GRAC/LigandDisplayForward?ligandId=4734 (p*K*_i_ 9.4) [http://www.ncbi.nlm.nih.gov/pubmed/14634039?dopt=AbstractPlus], http://www.guidetopharmacology.org/GRAC/LigandDisplayForward?ligandId=4513 (p*K*_i_ 9.3), http://www.guidetopharmacology.org/GRAC/LigandDisplayForward?ligandId=4717 (p*K*_i_ 9), http://www.guidetopharmacology.org/GRAC/LigandDisplayForward?ligandId=4807 (p*K*_i_ 8.8) [http://www.ncbi.nlm.nih.gov/pubmed/14634039?dopt=AbstractPlus], http://www.guidetopharmacology.org/GRAC/LigandDisplayForward?ligandId=1765 (p*K*_i_ 7.3) [http://www.ncbi.nlm.nih.gov/pubmed/24414167?dopt=AbstractPlus]	–
Labelled ligands	http://www.guidetopharmacology.org/GRAC/LigandDisplayForward?ligandId=4511 ^3^H]nitrobenzylmercaptopurine ribonucleoside (p*K*_d_ 9.3)	–
Comments	ENT1 has 100‐1000‐fold lower affinity for nucleobases as compared with nucleosides [http://www.ncbi.nlm.nih.gov/pubmed/21795683?dopt=AbstractPlus]. The affinities of http://www.guidetopharmacology.org/GRAC/LigandDisplayForward?ligandId=4590, http://www.guidetopharmacology.org/GRAC/LigandDisplayForward?ligandId=4717, http://www.guidetopharmacology.org/GRAC/LigandDisplayForward?ligandId=4734 and http://www.guidetopharmacology.org/GRAC/LigandDisplayForward?ligandId=4807 at ENT1 transporters are species dependent, exhibiting lower affinity at rat transporters than at human transporters [http://www.ncbi.nlm.nih.gov/pubmed/14634039?dopt=AbstractPlus, http://www.ncbi.nlm.nih.gov/pubmed/9705281?dopt=AbstractPlus]. The loss of ENT1 activity in ENT1‐null mice has been associated with a hypermineralization disorder similar to human diffuse idiopathic skeletal hyperostosis [http://www.ncbi.nlm.nih.gov/pubmed/23184610?dopt=AbstractPlus]. Lack of ENT1 also results in the Augustine‐null blood type [http://www.ncbi.nlm.nih.gov/pubmed/25896650?dopt=AbstractPlus].	–

**Table:** 

Nomenclature	http://www.guidetopharmacology.org/GRAC/ObjectDisplayForward?objectId=1119	http://www.guidetopharmacology.org/GRAC/ObjectDisplayForward?objectId=1120
Systematic nomenclature	SLC29A3	SLC29A4
Common abbreviation	ENT3	PMAT
HGNC, UniProt	https://www.genenames.org/data/gene‐symbol‐report/#!/hgnc_id/HGNC:23096, http://www.uniprot.org/uniprot/Q9BZD2	https://www.genenames.org/data/gene‐symbol‐report/#!/hgnc_id/HGNC:23097, http://www.uniprot.org/uniprot/Q7RTT9
Substrates	http://www.guidetopharmacology.org/GRAC/LigandDisplayForward?ligandId=4825 [http://www.ncbi.nlm.nih.gov/pubmed/15701636?dopt=AbstractPlus], http://www.guidetopharmacology.org/GRAC/LigandDisplayForward?ligandId=4828 [http://www.ncbi.nlm.nih.gov/pubmed/15701636?dopt=AbstractPlus], http://www.guidetopharmacology.org/GRAC/LigandDisplayForward?ligandId=4833 [http://www.ncbi.nlm.nih.gov/pubmed/15701636?dopt=AbstractPlus], http://www.guidetopharmacology.org/GRAC/LigandDisplayForward?ligandId=4802 [http://www.ncbi.nlm.nih.gov/pubmed/15701636?dopt=AbstractPlus], http://www.guidetopharmacology.org/GRAC/LigandDisplayForward?ligandId=4630 [http://www.ncbi.nlm.nih.gov/pubmed/15701636?dopt=AbstractPlus], http://www.guidetopharmacology.org/GRAC/LigandDisplayForward?ligandId=4801 [http://www.ncbi.nlm.nih.gov/pubmed/15701636?dopt=AbstractPlus], http://www.guidetopharmacology.org/GRAC/LigandDisplayForward?ligandId=4799 [http://www.ncbi.nlm.nih.gov/pubmed/15701636?dopt=AbstractPlus], http://www.guidetopharmacology.org/GRAC/LigandDisplayForward?ligandId=4755 [http://www.ncbi.nlm.nih.gov/pubmed/15701636?dopt=AbstractPlus], http://www.guidetopharmacology.org/GRAC/LigandDisplayForward?ligandId=4729 [http://www.ncbi.nlm.nih.gov/pubmed/15701636?dopt=AbstractPlus]	http://www.guidetopharmacology.org/GRAC/LigandDisplayForward?ligandId=2343 [http://www.ncbi.nlm.nih.gov/pubmed/16099839?dopt=AbstractPlus, http://www.ncbi.nlm.nih.gov/pubmed/27506881?dopt=AbstractPlus], http://www.guidetopharmacology.org/GRAC/LigandDisplayForward?ligandId=4568 [http://www.ncbi.nlm.nih.gov/pubmed/16099839?dopt=AbstractPlus, http://www.ncbi.nlm.nih.gov/pubmed/27506881?dopt=AbstractPlus], http://www.guidetopharmacology.org/GRAC/LigandDisplayForward?ligandId=4779 [http://www.ncbi.nlm.nih.gov/pubmed/17600084?dopt=AbstractPlus], http://www.guidetopharmacology.org/GRAC/LigandDisplayForward?ligandId=548 [http://www.ncbi.nlm.nih.gov/pubmed/28089688?dopt=AbstractPlus]
Endogenous substrates	http://www.guidetopharmacology.org/GRAC/LigandDisplayForward?ligandId=2844 [http://www.ncbi.nlm.nih.gov/pubmed/15701636?dopt=AbstractPlus], http://www.guidetopharmacology.org/GRAC/LigandDisplayForward?ligandId=4554 [http://www.ncbi.nlm.nih.gov/pubmed/15701636?dopt=AbstractPlus], http://www.guidetopharmacology.org/GRAC/LigandDisplayForward?ligandId=4566 [http://www.ncbi.nlm.nih.gov/pubmed/15701636?dopt=AbstractPlus], http://www.guidetopharmacology.org/GRAC/LigandDisplayForward?ligandId=4718 [http://www.ncbi.nlm.nih.gov/pubmed/15701636?dopt=AbstractPlus], http://www.guidetopharmacology.org/GRAC/LigandDisplayForward?ligandId=4567 [http://www.ncbi.nlm.nih.gov/pubmed/15701636?dopt=AbstractPlus], http://www.guidetopharmacology.org/GRAC/LigandDisplayForward?ligandId=4788 [http://www.ncbi.nlm.nih.gov/pubmed/15701636?dopt=AbstractPlus]	http://www.guidetopharmacology.org/GRAC/LigandDisplayForward?ligandId=1204 [http://www.ncbi.nlm.nih.gov/pubmed/16099839?dopt=AbstractPlus, http://www.ncbi.nlm.nih.gov/pubmed/27506881?dopt=AbstractPlus], http://www.guidetopharmacology.org/GRAC/LigandDisplayForward?ligandId=2150 [http://www.ncbi.nlm.nih.gov/pubmed/16099839?dopt=AbstractPlus, http://www.ncbi.nlm.nih.gov/pubmed/27506881?dopt=AbstractPlus], http://www.guidetopharmacology.org/GRAC/LigandDisplayForward?ligandId=2844 [http://www.ncbi.nlm.nih.gov/pubmed/20592246?dopt=AbstractPlus], http://www.guidetopharmacology.org/GRAC/LigandDisplayForward?ligandId=5 [http://www.ncbi.nlm.nih.gov/pubmed/16099839?dopt=AbstractPlus, http://www.ncbi.nlm.nih.gov/pubmed/27506881?dopt=AbstractPlus], http://www.guidetopharmacology.org/GRAC/LigandDisplayForward?ligandId=940 [http://www.ncbi.nlm.nih.gov/pubmed/16099839?dopt=AbstractPlus, http://www.ncbi.nlm.nih.gov/pubmed/27506881?dopt=AbstractPlus]
Stoichiometry	Equilibrative	Equilibrative
Inhibitors	–	http://www.guidetopharmacology.org/GRAC/LigandDisplayForward?ligandId=8482 (p*K*_i_ 7) [http://www.ncbi.nlm.nih.gov/pubmed/16099839?dopt=AbstractPlus, http://www.ncbi.nlm.nih.gov/pubmed/27506881?dopt=AbstractPlus], http://www.guidetopharmacology.org/GRAC/LigandDisplayForward?ligandId=4607 (p*K*_i_ 6) [http://www.ncbi.nlm.nih.gov/pubmed/16099839?dopt=AbstractPlus, http://www.ncbi.nlm.nih.gov/pubmed/27506881?dopt=AbstractPlus], http://www.guidetopharmacology.org/GRAC/LigandDisplayForward?ligandId=4807 (p*K*_i_ 5.9) [http://www.ncbi.nlm.nih.gov/pubmed/24021350?dopt=AbstractPlus], http://www.guidetopharmacology.org/GRAC/LigandDisplayForward?ligandId=2406 (p*K*_i_ 4.7) [http://www.ncbi.nlm.nih.gov/pubmed/16099839?dopt=AbstractPlus, http://www.ncbi.nlm.nih.gov/pubmed/27506881?dopt=AbstractPlus], http://www.guidetopharmacology.org/GRAC/LigandDisplayForward?ligandId=203 (p*K*_i_ 4.6) [http://www.ncbi.nlm.nih.gov/pubmed/16099839?dopt=AbstractPlus, http://www.ncbi.nlm.nih.gov/pubmed/27506881?dopt=AbstractPlus], http://www.guidetopharmacology.org/GRAC/LigandDisplayForward?ligandId=2342 (p*K*_i_ 4.6) [http://www.ncbi.nlm.nih.gov/pubmed/16099839?dopt=AbstractPlus, http://www.ncbi.nlm.nih.gov/pubmed/27506881?dopt=AbstractPlus], http://www.guidetopharmacology.org/GRAC/LigandDisplayForward?ligandId=2510 (p*K*_i_ 4.6) [http://www.ncbi.nlm.nih.gov/pubmed/16099839?dopt=AbstractPlus, http://www.ncbi.nlm.nih.gov/pubmed/27506881?dopt=AbstractPlus], http://www.guidetopharmacology.org/GRAC/LigandDisplayForward?ligandId=2399 (p*K*_i_ 4.5) [http://www.ncbi.nlm.nih.gov/pubmed/16099839?dopt=AbstractPlus, http://www.ncbi.nlm.nih.gov/pubmed/27506881?dopt=AbstractPlus], http://www.guidetopharmacology.org/GRAC/LigandDisplayForward?ligandId=1231 (p*K*_i_ <3.3) [http://www.ncbi.nlm.nih.gov/pubmed/16099839?dopt=AbstractPlus, http://www.ncbi.nlm.nih.gov/pubmed/27506881?dopt=AbstractPlus]
Comments	Defects in *SLC29A3* have been implicated in histiocytosis‐lymphadenopathy plus syndrome (http://omim.org/entry/602782) and lysosomal storage diseases [http://www.ncbi.nlm.nih.gov/pubmed/22174130?dopt=AbstractPlus, http://www.ncbi.nlm.nih.gov/pubmed/20595384?dopt=AbstractPlus].	Uptake of substrates by PMAT is pH dependent, with greater uptake observed at acidic extracellular pH [http://www.ncbi.nlm.nih.gov/pubmed/16873718?dopt=AbstractPlus, http://www.ncbi.nlm.nih.gov/pubmed/17600084?dopt=AbstractPlus].

### Further reading on SLC29 family

Boswell‐Casteel RC *et al*. (2017) Equilibrative nucleoside transporters‐A review. *Nucleosides Nucleotides Nucleic Acids* **36**: 7–30 https://www.ncbi.nlm.nih.gov/pubmed/27759477?dopt=AbstractPlus

Pastor‐Anglada M *et al*. (2018) Who Is Who in Adenosine Transport. *Front Pharmacol* **9**: 627 https://www.ncbi.nlm.nih.gov/pubmed/29962948?dopt=AbstractPlus

Wang J. (2016) The plasma membranemonoamine transporter (PMAT): Structure, function, and role in organic cation disposition. *Clin. Pharmacol. Ther*. **100**: 489–499 https://www.ncbi.nlm.nih.gov/pubmed/27506881?dopt=AbstractPlus

### Further reading on SLC28 and SLC29 families of nucleoside transporters

Boswell‐Casteel RC *et al*. (2017) Equilibrative nucleoside transporters‐A review. *Nucleosides Nucleotides Nucleic Acids* **36**: 7–30 https://www.ncbi.nlm.nih.gov/pubmed/27759477?dopt=AbstractPlus

Pastor‐Anglada M *et al*. (2015) Nucleoside transporter proteins as biomarkers of drug responsiveness and drug targets. *Front Pharmacol* **6**: 13 https://www.ncbi.nlm.nih.gov/pubmed/25713533?dopt=AbstractPlus

Young JD. (2016) The SLC28 (CNT) and SLC29 (ENT) nucleoside transporter families: a 30‐year collaborative odyssey. *Biochem. Soc. Trans*. **44**: 869–76 https://www.ncbi.nlm.nih.gov/pubmed/27284054?dopt=AbstractPlus

Young JD *et al*. (2013) The human concentrative and equilibrative nucleoside transporter families, SLC28 and SLC29. *Mol. Aspects Med*. **34**: 529–47 https://www.ncbi.nlm.nih.gov/pubmed/23506887?dopt=AbstractPlus

## http://www.guidetopharmacology.org/GRAC/FamilyDisplayForward?familyId=217

### Overview

Along with the http://www.guidetopharmacology.org/GRAC/FamilyDisplayForward?familyId=228, SLC30 transporters regulate the movement of zinc ions around the cell. In particular, these transporters remove zinc ions from the cytosol, allowing accumulation into intracellular compartments or efflux through the plasma membrane. ZnT1 is thought to be placed on the plasma membrane extruding zinc, while ZnT3 is associated with synaptic vesicles and ZnT4 and ZnT5 are linked with secretory granules. Membrane topology predictions suggest a multimeric assembly, potentially heteromultimeric [http://www.ncbi.nlm.nih.gov/pubmed/15994300?dopt=AbstractPlus], with subunits having six TM domains, and both termini being cytoplasmic. Dityrosine covalent linking has been suggested as a mechanism for dimerisation, particularly for ZnT3 [http://www.ncbi.nlm.nih.gov/pubmed/19521526?dopt=AbstractPlus]. The mechanism for zinc transport is unknown.

Information on members of this family may be found in the http://www.guidetopharmacology.org/GRAC/FamilyDisplayForward?familyId=217.

### Comments

ZnT8/SLC30A8 is described as a type 1 diabetes susceptibility gene.

Zinc fluxes may be monitored through the use of radioisotopic Zn‐65 or the fluorescent dye FluoZin 3.

### Further reading on SLC30 zinc transporter family

Bouron A *et al*. (2014) Contribution of calcium‐conducting channels to the transport of zinc ions. *Pflugers Arch*. **466**: 381–7 https://www.ncbi.nlm.nih.gov/pubmed/23719866?dopt=AbstractPlus

Hojyo S *et al*. (2016) Zinc transporters and signaling in physiology and pathogenesis. *Arch. Biochem. Biophys*. **611**: 43–50 https://www.ncbi.nlm.nih.gov/pubmed/27394923?dopt=AbstractPlus

Huang L *et al*. (2013) The SLC30 family of zinc transporters ‐ a review of current understanding of their biological and pathophysiological roles. *Mol. Aspects Med*. **34**: 548‐60 https://www.ncbi.nlm.nih.gov/pubmed/23506888?dopt=AbstractPlus

Kambe T *et al*. (2014) Current understanding of ZIP and ZnT zinc transporters in human health and diseases. *Cell. Mol. Life Sci*. **71**: 3281–95 https://www.ncbi.nlm.nih.gov/pubmed/24710731?dopt=AbstractPlus

Kambe T *et al*. (2015) The Physiological, Biochemical, and Molecular Roles of Zinc Transporters in Zinc Homeostasis and Metabolism. *Physiol. Rev*. **95**: 749–784 https://www.ncbi.nlm.nih.gov/pubmed/26084690?dopt=AbstractPlus

## http://www.guidetopharmacology.org/GRAC/FamilyDisplayForward?familyId=218

### Overview

SLC31 family members, alongside the http://www.guidetopharmacology.org/GRAC/FamilyDisplayForward?familyId=138#Cu2+‐ATPase are involved in the regulation of cellular copper levels. The CTR1 transporter is a cell‐surface transporter to allow monovalent copper accumulation into cells, while CTR2 appears to be a vacuolar/vesicular transporter [http://www.ncbi.nlm.nih.gov/pubmed/15494390?dopt=AbstractPlus]. Functional copper transporters appear to be trimeric with each subunit having three TM regions and an extracellular N‐terminus. CTR1 is considered to be a higher affinity copper transporter compared to CTR2. The stoichiometry of copper accumulation is unclear, but appears to be energy‐independent [http://www.ncbi.nlm.nih.gov/pubmed/11734551?dopt=AbstractPlus].

**Table:** 

Nomenclature	http://www.guidetopharmacology.org/GRAC/ObjectDisplayForward?objectId=1131	http://www.guidetopharmacology.org/GRAC/ObjectDisplayForward?objectId=1132
Systematic nomenclature	SLC31A1	SLC31A2
Common abbreviation	CTR1	CTR2
HGNC, UniProt	https://www.genenames.org/data/gene‐symbol‐report/#!/hgnc_id/HGNC:11016, http://www.uniprot.org/uniprot/O15431	https://www.genenames.org/data/gene‐symbol‐report/#!/hgnc_id/HGNC:11017, http://www.uniprot.org/uniprot/O15432
Substrates	http://www.guidetopharmacology.org/GRAC/LigandDisplayForward?ligandId=5343 [http://www.ncbi.nlm.nih.gov/pubmed/12370430?dopt=AbstractPlus]	http://www.guidetopharmacology.org/GRAC/LigandDisplayForward?ligandId=5343 [http://www.ncbi.nlm.nih.gov/pubmed/19509135?dopt=AbstractPlus]
Endogenous substrates	copper [http://www.ncbi.nlm.nih.gov/pubmed/11734551?dopt=AbstractPlus]	copper
Stoichiometry	Unknown	Unknown

### Comments

Copper accumulation through CTR1 is sensitive to silver ions, but not divalent cations [http://www.ncbi.nlm.nih.gov/pubmed/11734551?dopt=AbstractPlus].

### Further reading on SLC31 family of copper transporters

Howell SB *et al*. (2010) Copper transporters and the cellular pharmacology of the platinumcontaining cancer drugs. *Mol. Pharmacol*. **77**: 887–94 https://www.ncbi.nlm.nih.gov/pubmed/20159940?dopt=AbstractPlus

Kaplan JH *et al*. (2016) How Mammalian Cells Acquire Copper: An Essential but Potentially Toxic Metal. *Biophys. J*. **110**: 7–13 https://www.ncbi.nlm.nih.gov/pubmed/26745404?dopt=AbstractPlus

Kim H *et al*. (2013) SLC31 (CTR) family of copper transporters in health and disease. *Mol. Aspects Med*. **34**: 561–70 https://www.ncbi.nlm.nih.gov/pubmed/23506889?dopt=AbstractPlus

Monne M *et al*. (2014) Antiporters of the mitochondrial carrier family. *Curr Top Membr* **73**: 289–320 https://www.ncbi.nlm.nih.gov/pubmed/24745987?dopt=AbstractPlus

## http://www.guidetopharmacology.org/GRAC/FamilyDisplayForward?familyId=219

### Overview

The vesicular inhibitory amino acid transporter, VIAAT (also termed the vesicular GABA transporter VGAT), which is the sole representative of the SLC32 family, transports http://www.guidetopharmacology.org/GRAC/LigandDisplayForward?ligandId=1067, or http://www.guidetopharmacology.org/GRAC/LigandDisplayForward?ligandId=727, into synaptic vesicles [http://www.ncbi.nlm.nih.gov/pubmed/12750892?dopt=AbstractPlus, http://www.ncbi.nlm.nih.gov/pubmed/10865121?dopt=AbstractPlus], and is a member of the structurally‐defined amino acid‐polyamineorganocation/ APC clan composed of SLC32, SLC36 and SLC38 transporter families (see [http://www.ncbi.nlm.nih.gov/pubmed/23506890?dopt=AbstractPlus]). VIAAT was originally suggested to be composed of 10 TM segments with cytoplasmic N‐ and C‐termini [http://www.ncbi.nlm.nih.gov/pubmed/9349821?dopt=AbstractPlus]. However, an alternative 9TM structure with the N terminus facing the cytoplasm and the C terminus residing in the synaptic vesicle lumen has subsequently been reported [http://www.ncbi.nlm.nih.gov/pubmed/19052203?dopt=AbstractPlus]. VI‐AAT acts as an antiporter for inhibitory amino acids and protons. The accumulation ofGABA and glycine within vesicles is driven by both the chemical (ΔpH) and electrical (Δψ) components of the proton electrochemical gradient (Δμ_H_^+^) established by a vacuolar H^+^‐ATPase [http://www.ncbi.nlm.nih.gov/pubmed/9349821?dopt=AbstractPlus]. However, one study, [http://www.ncbi.nlm.nih.gov/pubmed/19843525?dopt=AbstractPlus], presented evidence that VIAAT is instead a Cl^‐^/GABA co‐transporter. VIAAT co‐exists with http://www.guidetopharmacology.org/GRAC/FamilyDisplayForward?familyId=145#show_object_1007 (SLC17A7), or http://www.guidetopharmacology.org/GRAC/FamilyDisplayForward?familyId=145#show_object_1008 (SLC17A6), in the synaptic vesicles of selected nerve terminals [http://www.ncbi.nlm.nih.gov/pubmed/19627441?dopt=AbstractPlus, http://www.ncbi.nlm.nih.gov/pubmed/20519538?dopt=AbstractPlus]. VIAAT knock out mice die between embryonic day 18.5 and birth [http://www.ncbi.nlm.nih.gov/pubmed/16701208?dopt=AbstractPlus]. In cultures of spinal cord neurones established from earlier embryos, the corelease of of http://www.guidetopharmacology.org/GRAC/LigandDisplayForward?ligandId=1067 and http://www.guidetopharmacology.org/GRAC/LigandDisplayForward?ligandId=727 from synaptic vesicles is drastically reduced, providing direct evidence for the role of VIAAT in the sequestration of both transmitters [http://www.ncbi.nlm.nih.gov/pubmed/21190592?dopt=AbstractPlus, http://www.ncbi.nlm.nih.gov/pubmed/16701208?dopt=AbstractPlus].

**Table:** 

Nomenclature	http://www.guidetopharmacology.org/GRAC/ObjectDisplayForward?objectId=1133
Systematic nomenclature	SLC32A1
Common abbreviation	VIAAT
HGNC, UniProt	https://www.genenames.org/data/gene‐symbol‐report/#!/hgnc_id/HGNC:11018, http://www.uniprot.org/uniprot/Q9H598
Endogenous substrates	β‐alanine, γ‐hydroxybutyric acid, GABA (*K*_m_ 5×10^‐3^M) [http://www.ncbi.nlm.nih.gov/pubmed/9349821?dopt=AbstractPlus], http://www.guidetopharmacology.org/GRAC/LigandDisplayForward?ligandId=727
Stoichiometry	1 amino acid (in): 1 H^+^ (out) [http://www.ncbi.nlm.nih.gov/pubmed/12750892?dopt=AbstractPlus] or 1 amino acid: 2Cl^‐^ (in) [http://www.ncbi.nlm.nih.gov/pubmed/19843525?dopt=AbstractPlus]
Inhibitors	http://www.guidetopharmacology.org/GRAC/LigandDisplayForward?ligandId=4821 (pIC_50_ 2.1) [http://www.ncbi.nlm.nih.gov/pubmed/9349821?dopt=AbstractPlus]

### Further reading on SLC32 vesicular inhibitory amino acid transporter

Anne C *et al*. (2014) Vesicular neurotransmitter transporters: mechanistic aspects. *Curr Top Membr* **73**: 149‐74 https://www.ncbi.nlm.nih.gov/pubmed/24745982?dopt=AbstractPlus

Schiöth HB *et al*. (2013) Evolutionary origin of amino acid transporter families SLC32, SLC36 and SLC38 and physiological, pathological and therapeutic aspects. *Mol. Aspects Med*. **34**: 571‐85 https://www.ncbi.nlm.nih.gov/pubmed/23506890?dopt=AbstractPlus

## http://www.guidetopharmacology.org/GRAC/FamilyDisplayForward?familyId=220

### Overview

Acetylation of proteins is a post‐translational modification mediated by specific acetyltransferases, using the donor http://www.guidetopharmacology.org/GRAC/LigandDisplayForward?ligandId=3038. SLC33A1/AT1 is a putative 11 TM transporter present on the endoplasmic reticulum, expressed in all tissues, but particularly abundant in the pancreas [http://www.ncbi.nlm.nih.gov/pubmed/9096318?dopt=AbstractPlus], which imports cytosolic http://www.guidetopharmacology.org/GRAC/LigandDisplayForward?ligandId=3038 into these intracellular organelles.

**Table:** 

Nomenclature	http://www.guidetopharmacology.org/GRAC/ObjectDisplayForward?objectId=1134
Systematic nomenclature	SLC33A1
Common abbreviation	ACATN1
HGNC, UniProt	https://www.genenames.org/data/gene‐symbol‐report/#!/hgnc_id/HGNC:95, http://www.uniprot.org/uniprot/O00400
Endogenous substrates	http://www.guidetopharmacology.org/GRAC/LigandDisplayForward?ligandId=3038
Stoichiometry	Unknown
Labelled ligands	http://www.guidetopharmacology.org/GRAC/LigandDisplayForward?ligandId=4541 (Binding)

### Comments

In heterologous expression studies, http://www.guidetopharmacology.org/GRAC/LigandDisplayForward?ligandId=3038 transport through AT1 was inhibited by http://www.guidetopharmacology.org/GRAC/LigandDisplayForward?ligandId=3044, but not http://www.guidetopharmacology.org/GRAC/LigandDisplayForward?ligandId=1058, http://www.guidetopharmacology.org/GRAC/LigandDisplayForward?ligandId=1713 or http://www.guidetopharmacology.org/GRAC/LigandDisplayForward?ligandId=1782[http://www.ncbi.nlm.nih.gov/pubmed/20826464?dopt=AbstractPlus]. A loss‐of‐function mutation in ACATN1/SLC33A1 has been associated with spastic paraplegia (SPG42, [http://www.ncbi.nlm.nih.gov/pubmed/19061983?dopt=AbstractPlus]), although this observation could not be replicated in a subsequent study [http://www.ncbi.nlm.nih.gov/pubmed/20461110?dopt=AbstractPlus].

### Further reading on SLC33 acetylCoA transporter

Hirabayashi Y *et al*. (2004) The acetyl‐CoA transporter family SLC33. *Pflugers Arch*. **447**: 760‐2 https://www.ncbi.nlm.nih.gov/pubmed/12739170?dopt=AbstractPlus

Hirabayashi Y *et al*. (2013) The acetyl‐CoA transporter family SLC33. *Mol. Aspects Med*. **34**: 586‐9 https://www.ncbi.nlm.nih.gov/pubmed/23506891?dopt=AbstractPlus

## http://www.guidetopharmacology.org/GRAC/FamilyDisplayForward?familyId=221

### Overview

The SLC34 family are sometimes referred to as Type II sodium‐phosphate co‐transporters, alongside Type I (http://www.guidetopharmacology.org/GRAC/FamilyDisplayForward?familyId=145) and Type III (http://www.guidetopharmacology.org/GRAC/FamilyDisplayForward?familyId=195) transporters. Topological modelling suggests eight TM domains with C‐ and N‐ termini in the cytoplasm, and a re‐entrant loop at TM7/8. SLC34 family members are expressed on the apical surfaces of epithelia in the intestine and kidneys to regulate body phosphate levels, principally NaPi‐IIa and NaPi‐IIb, respectively. NaPi‐IIa and NaPi‐IIb are electrogenic, while NaPiIIc is electroneutral [http://www.ncbi.nlm.nih.gov/pubmed/18989094?dopt=AbstractPlus].

**Table:** 

Nomenclature	http://www.guidetopharmacology.org/GRAC/ObjectDisplayForward?objectId=1135	http://www.guidetopharmacology.org/GRAC/ObjectDisplayForward?objectId=1136	http://www.guidetopharmacology.org/GRAC/ObjectDisplayForward?objectId=1137
Systematic nomenclature	SLC34A1	SLC34A2	SLC34A3
Common abbreviation	NaPi‐IIa	NaPi‐IIb	NaPi‐IIc
HGNC, UniProt	https://www.genenames.org/data/gene‐symbol‐report/#!/hgnc_id/HGNC:11019, http://www.uniprot.org/uniprot/Q06495	https://www.genenames.org/data/gene‐symbol‐report/#!/hgnc_id/HGNC:11020, http://www.uniprot.org/uniprot/O95436	https://www.genenames.org/data/gene‐symbol‐report/#!/hgnc_id/HGNC:20305, http://www.uniprot.org/uniprot/Q8N130
Stoichiometry	3 Na^+^ : 1 HPO_4_^2‐^ (in) [http://www.ncbi.nlm.nih.gov/pubmed/10198426?dopt=AbstractPlus]	3 Na^+^ : 1 HPO_4_^2‐^ (in) [http://www.ncbi.nlm.nih.gov/pubmed/18989094?dopt=AbstractPlus]	2 Na^+^ : 1 HPO_4_^2‐^ (in) [http://www.ncbi.nlm.nih.gov/pubmed/18989094?dopt=AbstractPlus]
Antibodies	–	http://www.guidetopharmacology.org/GRAC/LigandDisplayForward?ligandId=8405 (Binding) [146]	–

### Comments

These transporters can be inhibited by http://www.guidetopharmacology.org/GRAC/LigandDisplayForward?ligandId=5497, in contrast to type III sodium‐phosphate cotransporters, the http://www.guidetopharmacology.org/GRAC/FamilyDisplayForward?familyId=195.

### Further reading on SLC34 family of sodium phosphate co‐transporters

Biber J *et al*. (2013) Phosphate transporters and their function. *Annu. Rev. Physiol*. **75**: 535‐50 https://www.ncbi.nlm.nih.gov/pubmed/23398154?dopt=AbstractPlus

Forster IC *et al*. (2013) Phosphate transporters of the SLC20 and SLC34 families. *Mol. Aspects Med*. **34**: 386‐95 https://www.ncbi.nlm.nih.gov/pubmed/23506879?dopt=AbstractPlus

Shobeiri N *et al*. (2014) Phosphate: an old bone molecule but new cardiovascular risk factor. *Br J Clin Pharmacol* **77**: 39‐54 https://www.ncbi.nlm.nih.gov/pubmed/23506202?dopt=AbstractPlus

Wagner CA *et al*. (2014) The SLC34 family of sodium‐dependent phosphate transporters. *Pflugers Arch*. **466**: 139‐53 https://www.ncbi.nlm.nih.gov/pubmed/24352629?dopt=AbstractPlus

## http://www.guidetopharmacology.org/GRAC/FamilyDisplayForward?familyId=222

### Overview

Glycoprotein formation in the Golgi and endoplasmic reticulum relies on the accumulation of nucleotide‐conjugated sugars via the SLC35 family of transporters. These transporters have a predicted topology of 10 TM domains, with cytoplasmic termini, and function as exchangers, swopping nucleoside monophosphates for the corresponding nucleoside diphosphate conjugated sugar. Five subfamilies of transporters have been identified on the basis of sequence similarity, namely SLC35A1, SLC35A2, SLC35A3, SLC35A4 and SLC35A5; SLC35B1, SLC35B2, SLC35B3 and SLC35B4; SLC35C1 and SLC35C2; SLC35D1, SL35D1, SLC35D2 and SLC35D3, and the subfamily of orphan SLC35 transporters, SLC35E1‐4 and SLC35F1‐5.

**Table:** 

Nomenclature	http://www.guidetopharmacology.org/GRAC/ObjectDisplayForward?objectId=1138	http://www.guidetopharmacology.org/GRAC/ObjectDisplayForward?objectId=1139	http://www.guidetopharmacology.org/GRAC/ObjectDisplayForward?objectId=1140	http://www.guidetopharmacology.org/GRAC/ObjectDisplayForward?objectId=1144	http://www.guidetopharmacology.org/GRAC/ObjectDisplayForward?objectId=1145
Systematic nomenclature	SLC35A1	SLC35A2	SLC35A3	SLC35B2	SLC35B3
HGNC, UniProt	https://www.genenames.org/data/gene‐symbol‐report/#!/hgnc_id/HGNC:11021, http://www.uniprot.org/uniprot/P78382	https://www.genenames.org/data/gene‐symbol‐report/#!/hgnc_id/HGNC:11022, http://www.uniprot.org/uniprot/P78381	https://www.genenames.org/data/gene‐symbol‐report/#!/hgnc_id/HGNC:11023, http://www.uniprot.org/uniprot/Q9Y2D2	https://www.genenames.org/data/gene‐symbol‐report/#!/hgnc_id/HGNC:16872, http://www.uniprot.org/uniprot/Q8TB61	https://www.genenames.org/data/gene‐symbol‐report/#!/hgnc_id/HGNC:21601, http://www.uniprot.org/uniprot/Q9H1N7
Substrates	http://www.guidetopharmacology.org/GRAC/LigandDisplayForward?ligandId=4663 [http://www.ncbi.nlm.nih.gov/pubmed/9644260?dopt=AbstractPlus]	http://www.guidetopharmacology.org/GRAC/LigandDisplayForward?ligandId=1782 [http://www.ncbi.nlm.nih.gov/pubmed/9010752?dopt=AbstractPlus, http://www.ncbi.nlm.nih.gov/pubmed/8889805?dopt=AbstractPlus], http://www.guidetopharmacology.org/GRAC/LigandDisplayForward?ligandId=1779 [http://www.ncbi.nlm.nih.gov/pubmed/9010752?dopt=AbstractPlus, http://www.ncbi.nlm.nih.gov/pubmed/8889805?dopt=AbstractPlus]	http://www.guidetopharmacology.org/GRAC/LigandDisplayForward?ligandId=1779 [http://www.ncbi.nlm.nih.gov/pubmed/10393322?dopt=AbstractPlus]	http://www.guidetopharmacology.org/GRAC/LigandDisplayForward?ligandId=1719 [http://www.ncbi.nlm.nih.gov/pubmed/12716889?dopt=AbstractPlus]	http://www.guidetopharmacology.org/GRAC/LigandDisplayForward?ligandId=1719 [http://www.ncbi.nlm.nih.gov/pubmed/16492677?dopt=AbstractPlus]

**Table:** 

Nomenclature	http://www.guidetopharmacology.org/GRAC/ObjectDisplayForward?objectId=1146	http://www.guidetopharmacology.org/GRAC/ObjectDisplayForward?objectId=1147	http://www.guidetopharmacology.org/GRAC/ObjectDisplayForward?objectId=1149	http://www.guidetopharmacology.org/GRAC/ObjectDisplayForward?objectId=1150
Systematic nomenclature	SLC35B4	SLC35C1	SLC35D1	SLC35D2
HGNC, UniProt	https://www.genenames.org/data/gene‐symbol‐report/#!/hgnc_id/HGNC:20584, http://www.uniprot.org/uniprot/Q969S0	https://www.genenames.org/data/gene‐symbol‐report/#!/hgnc_id/HGNC:20197, http://www.uniprot.org/uniprot/Q96A29	https://www.genenames.org/data/gene‐symbol‐report/#!/hgnc_id/HGNC:20800, http://www.uniprot.org/uniprot/Q9NTN3	https://www.genenames.org/data/gene‐symbol‐report/#!/hgnc_id/HGNC:20799, http://www.uniprot.org/uniprot/Q76EJ3
Substrates	http://www.guidetopharmacology.org/GRAC/LigandDisplayForward?ligandId=4730 [http://www.ncbi.nlm.nih.gov/pubmed/15911612?dopt=AbstractPlus], http://www.guidetopharmacology.org/GRAC/LigandDisplayForward?ligandId=1779 [http://www.ncbi.nlm.nih.gov/pubmed/15911612?dopt=AbstractPlus]	http://www.guidetopharmacology.org/GRAC/LigandDisplayForward?ligandId=4578 [http://www.ncbi.nlm.nih.gov/pubmed/11326279?dopt=AbstractPlus]	http://www.guidetopharmacology.org/GRAC/LigandDisplayForward?ligandId=4741 [http://www.ncbi.nlm.nih.gov/pubmed/11322953?dopt=AbstractPlus], http://www.guidetopharmacology.org/GRAC/LigandDisplayForward?ligandId=1784 [http://www.ncbi.nlm.nih.gov/pubmed/11322953?dopt=AbstractPlus]	http://www.guidetopharmacology.org/GRAC/LigandDisplayForward?ligandId=4741 [http://www.ncbi.nlm.nih.gov/pubmed/15607426?dopt=AbstractPlus]

### Further reading on SLC35 family of nucleotide sugar transporters

Ishida N *et al*. (2004) Molecular physiology and pathology of the nucleotide sugar transporter family (SLC35). *Pflugers Arch*. **447**: 768‐75 https://www.ncbi.nlm.nih.gov/pubmed/12759756?dopt=AbstractPlus

Orellana A *et al*. (2016) Overview of Nucleotide Sugar Transporter Gene Family Functions Across Multiple Species. *J. Mol. Biol*. **428**: 3150‐3165 https://www.ncbi.nlm.nih.gov/pubmed/27261257?dopt=AbstractPlus

Song Z. (2013) Roles of the nucleotide sugar transporters (SLC35 family) in health and disease. *Mol. Aspects Med*. **34**: 590‐600 https://www.ncbi.nlm.nih.gov/pubmed/23506892?dopt=AbstractPlus

## http://www.guidetopharmacology.org/GRAC/FamilyDisplayForward?familyId=223

### Overview

Members of the SLC36 family of proton‐coupled amino acid transporters are involved in membrane transport of amino acids and derivatives. The four transporters show variable tissue expression patterns and are expressed in various cell types at the plasma‐membrane and in intracellular organelles. PAT1 is expressed at the luminal surface of the small intestine and absorbs amino acids and derivatives [3]. In lysosomes, PAT1 functions as an effluxmechanism for amino acids produced during intralysosomal proteolysis [http://www.ncbi.nlm.nih.gov/pubmed/12761825?dopt=AbstractPlus, http://www.ncbi.nlm.nih.gov/pubmed/11390972?dopt=AbstractPlus]. PAT2 is expressed at the apical membrane of the renal proximal tubule [http://www.ncbi.nlm.nih.gov/pubmed/19033659?dopt=AbstractPlus] and at the plasma‐membrane in brown/beige adipocytes [http://www.ncbi.nlm.nih.gov/pubmed/25080478?dopt=AbstractPlus]. PAT1 and PAT4 are involved in regulation of the mTORC1 pathway [http://www.ncbi.nlm.nih.gov/pubmed/29971004?dopt=AbstractPlus]. More comprehensive lists of substrates can be found within the reviews under Further Reading and in the references.

**Table:** 

Nomenclature	http://www.guidetopharmacology.org/GRAC/ObjectDisplayForward?objectId=1161	http://www.guidetopharmacology.org/GRAC/ObjectDisplayForward?objectId=1162
Systematic nomenclature	SLC36A1	SLC36A2
Common abbreviation	PAT1	PAT2
HGNC, UniProt	https://www.genenames.org/data/gene‐symbol‐report/#!/hgnc_id/HGNC:18761, http://www.uniprot.org/uniprot/Q7Z2H8	https://www.genenames.org/data/gene‐symbol‐report/#!/hgnc_id/HGNC:18762, http://www.uniprot.org/uniprot/Q495M3
Substrates	http://www.guidetopharmacology.org/GRAC/LigandDisplayForward?ligandId=4259 [http://www.ncbi.nlm.nih.gov/pubmed/21501141?dopt=AbstractPlus], http://www.guidetopharmacology.org/GRAC/LigandDisplayForward?ligandId=9487 [http://www.ncbi.nlm.nih.gov/pubmed/21501141?dopt=AbstractPlus], http://www.guidetopharmacology.org/GRAC/LigandDisplayForward?ligandId=4550 [http://www.ncbi.nlm.nih.gov/pubmed/21501141?dopt=AbstractPlus], http://www.guidetopharmacology.org/GRAC/LigandDisplayForward?ligandId=9488 [http://www.ncbi.nlm.nih.gov/pubmed/21501141?dopt=AbstractPlus], http://www.guidetopharmacology.org/GRAC/LigandDisplayForward?ligandId=4784 [http://www.ncbi.nlm.nih.gov/pubmed/21501141?dopt=AbstractPlus], http://www.guidetopharmacology.org/GRAC/LigandDisplayForward?ligandId=4322 [http://www.ncbi.nlm.nih.gov/pubmed/19594759?dopt=AbstractPlus, http://www.ncbi.nlm.nih.gov/pubmed/21501141?dopt=AbstractPlus], http://www.guidetopharmacology.org/GRAC/LigandDisplayForward?ligandId=4707 [http://www.ncbi.nlm.nih.gov/pubmed/21501141?dopt=AbstractPlus], http://www.guidetopharmacology.org/GRAC/LigandDisplayForward?ligandId=9489 [http://www.ncbi.nlm.nih.gov/pubmed/21501141?dopt=AbstractPlus], http://www.guidetopharmacology.org/GRAC/LigandDisplayForward?ligandId=4697 [http://www.ncbi.nlm.nih.gov/pubmed/21501141?dopt=AbstractPlus], http://www.guidetopharmacology.org/GRAC/LigandDisplayForward?ligandId=4821 [http://www.ncbi.nlm.nih.gov/pubmed/16331283?dopt=AbstractPlus, http://www.ncbi.nlm.nih.gov/pubmed/21501141?dopt=AbstractPlus], http://www.guidetopharmacology.org/GRAC/LigandDisplayForward?ligandId=4686 [http://www.ncbi.nlm.nih.gov/pubmed/21501141?dopt=AbstractPlus], http://www.guidetopharmacology.org/GRAC/LigandDisplayForward?ligandId=4727 [http://www.ncbi.nlm.nih.gov/pubmed/21501141?dopt=AbstractPlus]	http://www.guidetopharmacology.org/GRAC/LigandDisplayForward?ligandId=4697 [http://www.ncbi.nlm.nih.gov/pubmed/12727219?dopt=AbstractPlus], http://www.guidetopharmacology.org/GRAC/LigandDisplayForward?ligandId=9489, http://www.guidetopharmacology.org/GRAC/LigandDisplayForward?ligandId=9488, http://www.guidetopharmacology.org/GRAC/LigandDisplayForward?ligandId=4686 [http://www.ncbi.nlm.nih.gov/pubmed/15644866?dopt=AbstractPlus]
Endogenous substrates	http://www.guidetopharmacology.org/GRAC/LigandDisplayForward?ligandId=1067 [http://www.ncbi.nlm.nih.gov/pubmed/21501141?dopt=AbstractPlus], http://www.guidetopharmacology.org/GRAC/LigandDisplayForward?ligandId=720 [http://www.ncbi.nlm.nih.gov/pubmed/21501141?dopt=AbstractPlus], http://www.guidetopharmacology.org/GRAC/LigandDisplayForward?ligandId=2365 [http://www.ncbi.nlm.nih.gov/pubmed/21501141?dopt=AbstractPlus], http://www.guidetopharmacology.org/GRAC/LigandDisplayForward?ligandId=2379 [http://www.ncbi.nlm.nih.gov/pubmed/21501141?dopt=AbstractPlus], http://www.guidetopharmacology.org/GRAC/LigandDisplayForward?ligandId=4171 [http://www.ncbi.nlm.nih.gov/pubmed/21501141?dopt=AbstractPlus], http://www.guidetopharmacology.org/GRAC/LigandDisplayForward?ligandId=4676 [http://www.ncbi.nlm.nih.gov/pubmed/21501141?dopt=AbstractPlus], http://www.guidetopharmacology.org/GRAC/LigandDisplayForward?ligandId=4704 [http://www.ncbi.nlm.nih.gov/pubmed/21501141?dopt=AbstractPlus], http://www.guidetopharmacology.org/GRAC/LigandDisplayForward?ligandId=4713 [http://www.ncbi.nlm.nih.gov/pubmed/21501141?dopt=AbstractPlus], http://www.guidetopharmacology.org/GRAC/LigandDisplayForward?ligandId=4537 [http://www.ncbi.nlm.nih.gov/pubmed/21501141?dopt=AbstractPlus], http://www.guidetopharmacology.org/GRAC/LigandDisplayForward?ligandId=727 [http://www.ncbi.nlm.nih.gov/pubmed/21501141?dopt=AbstractPlus], http://www.guidetopharmacology.org/GRAC/LigandDisplayForward?ligandId=4678 [http://www.ncbi.nlm.nih.gov/pubmed/21501141?dopt=AbstractPlus]	http://www.guidetopharmacology.org/GRAC/LigandDisplayForward?ligandId=4713, http://www.guidetopharmacology.org/GRAC/LigandDisplayForward?ligandId=3314, http://www.guidetopharmacology.org/GRAC/LigandDisplayForward?ligandId=727, http://www.guidetopharmacology.org/GRAC/LigandDisplayForward?ligandId=720, http://www.guidetopharmacology.org/GRAC/LigandDisplayForward?ligandId=4704
Stoichiometry	1 H^+^ : 1 amino acid (symport)	1 H^+^ : 1 amino acid (symport)
Inhibitors	http://www.guidetopharmacology.org/GRAC/LigandDisplayForward?ligandId=4671 (p*K*_i_ 3) [http://www.ncbi.nlm.nih.gov/pubmed/16126914?dopt=AbstractPlus], http://www.guidetopharmacology.org/GRAC/LigandDisplayForward?ligandId=717 (p*K*_i_ 2.3) [http://www.ncbi.nlm.nih.gov/pubmed/16126914?dopt=AbstractPlus], http://www.guidetopharmacology.org/GRAC/LigandDisplayForward?ligandId=4709 (p*K*_i_ 2.3) [http://www.ncbi.nlm.nih.gov/pubmed/16126914?dopt=AbstractPlus], http://www.guidetopharmacology.org/GRAC/LigandDisplayForward?ligandId=5 (p*K*_i_ 2.2) [http://www.ncbi.nlm.nih.gov/pubmed/16126914?dopt=AbstractPlus]	http://www.guidetopharmacology.org/GRAC/LigandDisplayForward?ligandId=4671 (pIC_50_ 2.8) [http://www.ncbi.nlm.nih.gov/pubmed/20691150?dopt=AbstractPlus], http://www.guidetopharmacology.org/GRAC/LigandDisplayForward?ligandId=4693 (pIC_50_ 2.5) [http://www.ncbi.nlm.nih.gov/pubmed/20691150?dopt=AbstractPlus]
Comments	[^3^H] or [^14^C] labelled substrates as listed above are used as probes. PAT1 can also function as an electroneutral transport system for protons and fatty acids including acetic acid, propanoic acid and butyric acid [http://www.ncbi.nlm.nih.gov/pubmed/15345686?dopt=AbstractPlus]. In addition, forskolin, phosphodiesterase inhibitors, amiloride analogues and SLC9A3 (NHE3) selective inhibitors all reduce PAT1 activity indirectly (in intact mammalian intestinal epithelia such as human intestinal Caco‐2 cells) by inhibiting the Na^+^/H^+^ exchanger NHE3 which is required to maintain the H^+^‐electrochemical gradient driving force for H^+^/amino acid cotransport [http://www.ncbi.nlm.nih.gov/pubmed/15521011?dopt=AbstractPlus, http://www.ncbi.nlm.nih.gov/pubmed/15754324?dopt=AbstractPlus, http://www.ncbi.nlm.nih.gov/pubmed/21501141?dopt=AbstractPlus].	[^3^H] or [^14^C] labelled substrates as listed above are used as probes. Loss‐of‐function mutations in PAT2 lead to iminoglycinuria and hyperglycinuria in man [http://www.ncbi.nlm.nih.gov/pubmed/19033659?dopt=AbstractPlus]. PAT2 can also function as an electroneutral transport system for protons and fatty acids including acetic acid, propanoic acid and butyric acid [http://www.ncbi.nlm.nih.gov/pubmed/15345686?dopt=AbstractPlus]. Replacement of a Phe residue in transmembrane domain 3 with Cys (that has a smaller side‐chain) broadens substrate specificity to include larger substrates (*e.g*. methionine, leucine) [http://www.ncbi.nlm.nih.gov/pubmed/29058016?dopt=AbstractPlus].

**Table:** 

Nomenclature	http://www.guidetopharmacology.org/GRAC/ObjectDisplayForward?objectId=1163	http://www.guidetopharmacology.org/GRAC/ObjectDisplayForward?objectId=1164
Systematic nomenclature	SLC36A3	SLC36A4
Common abbreviation	PAT3	PAT4
HGNC, UniProt	https://www.genenames.org/data/gene‐symbol‐report/#!/hgnc_id/HGNC:19659, http://www.uniprot.org/uniprot/Q495N2	https://www.genenames.org/data/gene‐symbol‐report/#!/hgnc_id/HGNC:19660, http://www.uniprot.org/uniprot/Q6YBV0
Endogenous substrates	–	http://www.guidetopharmacology.org/GRAC/LigandDisplayForward?ligandId=717 [http://www.ncbi.nlm.nih.gov/pubmed/21097500?dopt=AbstractPlus], http://www.guidetopharmacology.org/GRAC/LigandDisplayForward?ligandId=3314 [http://www.ncbi.nlm.nih.gov/pubmed/21097500?dopt=AbstractPlus]
Stoichiometry	Unknown	Unknown
Comments	The function of the testes‐specific PAT3 remains unknown.	PAT4 is not proton‐coupled and functions by facilitated diffusion in an electroneutral, Na^+^‐independent, manner [http://www.ncbi.nlm.nih.gov/pubmed/21097500?dopt=AbstractPlus]. PAT4 is expressed ubiquitously and is predominantly associated with the Golgi [http://www.ncbi.nlm.nih.gov/pubmed/26434594?dopt=AbstractPlus].. High PAT4 expression is associated with reduced relapse‐free survival after colorectal cancer surgery [http://www.ncbi.nlm.nih.gov/pubmed/26434594?dopt=AbstractPlus].

### Further reading on SLC36 family of proton‐coupled amino acid transporters

Schiöth HB *et al*. (2013) Evolutionary origin of amino acid transporter families SLC32, SLC36 and SLC38 and physiological, pathological and therapeutic aspects. *Mol. Aspects Med*. **34**: 571‐85 https://www.ncbi.nlm.nih.gov/pubmed/23506890?dopt=AbstractPlus

Thwaites DT *et al*. (2011) The SLC36 family of proton‐coupled amino acid transporters and their potential role in drug transport. *Br. J. Pharmacol*. **164**: 1802‐16 https://www.ncbi.nlm.nih.gov/pubmed/21501141?dopt=AbstractPlus

Thwaites DT *et al*. (2007) Deciphering the mechanisms of intestinal imino (and amino) acid transport: the redemption of SLC36A1. *Biochim. Biophys. Acta* **1768**: 179‐97 https://www.ncbi.nlm.nih.gov/pubmed/17123464?dopt=AbstractPlus

## http://www.guidetopharmacology.org/GRAC/FamilyDisplayForward?familyId=224

### Overview

The family of sugar‐phosphate exchangers pass particular phosphorylated sugars across intracellular membranes, exchanging for inorganic phosphate. Of the family of sugar phosphate transporters, most information is available on SPX4, the glucose‐6‐phosphate transporter. This is a 10 TM domain protein with cytoplasmic termini and is associated with the endoplasmic reticulum, with tissue‐specific splice variation.

**Table:** 

Nomenclature	http://www.guidetopharmacology.org/GRAC/ObjectDisplayForward?objectId=1165	http://www.guidetopharmacology.org/GRAC/ObjectDisplayForward?objectId=1166	http://www.guidetopharmacology.org/GRAC/ObjectDisplayForward?objectId=1168
Systematic nomenclature	SLC37A1	SLC37A2	SLC37A4
Common abbreviation	SPX1	SPX2	SPX4
HGNC, UniProt	https://www.genenames.org/data/gene‐symbol‐report/#!/hgnc_id/HGNC:11024, http://www.uniprot.org/uniprot/P57057	https://www.genenames.org/data/gene‐symbol‐report/#!/hgnc_id/HGNC:20644, http://www.uniprot.org/uniprot/Q8TED4	https://www.genenames.org/data/gene‐symbol‐report/#!/hgnc_id/HGNC:4061, http://www.uniprot.org/uniprot/O43826
Endogenous substrates	http://www.guidetopharmacology.org/GRAC/LigandDisplayForward?ligandId=4740, http://www.guidetopharmacology.org/GRAC/LigandDisplayForward?ligandId=4647	http://www.guidetopharmacology.org/GRAC/LigandDisplayForward?ligandId=4647	http://www.guidetopharmacology.org/GRAC/LigandDisplayForward?ligandId=4647
Stoichiometry	Glucose 6‐phosphate (in): phosphate (out) [http://www.ncbi.nlm.nih.gov/pubmed/21949678?dopt=AbstractPlus].	Glucose 6‐phosphate (in): phosphate (out) [http://www.ncbi.nlm.nih.gov/pubmed/21949678?dopt=AbstractPlus].	Glucose 6‐phosphate (in): phosphate (out) [http://www.ncbi.nlm.nih.gov/pubmed/18337460?dopt=AbstractPlus].
Inhibitors	–	–	http://www.guidetopharmacology.org/GRAC/LigandDisplayForward?ligandId=8845 (pIC_50_ 8.7) [101] – Rat
Comments	–	–	Multiple polymorphisms have been described for the SLC37A4 gene, some of which associate with a glycogen storage disease [http://www.ncbi.nlm.nih.gov/pubmed/15260472?dopt=AbstractPlus].

### Further reading on SLC37 family of phosphosugar/phosphate exchangers

Chou JY *et al*. (2014) The SLC37 family of sugar‐phosphate/phosphate exchangers. *Curr Top Membr* **73**: 357–82 https://www.ncbi.nlm.nih.gov/pubmed/24745989?dopt=AbstractPlus

Chou JY *et al*. (2013) The SLC37 family of phosphate‐linked sugar phosphate antiporters. *Mol. Aspects Med*. **34**: 601–11 https://www.ncbi.nlm.nih.gov/pubmed/23506893?dopt=AbstractPlus

## http://www.guidetopharmacology.org/GRAC/FamilyDisplayForward?familyId=150

### Overview

The SLC38 family of transporters appears to be responsible for the functionally‐defined system A and system N mechanisms of amino acid transport and are mostly expressed in the CNS. Two distinct subfamilies are identifiable within the SLC38 transporters. SNAT1, SNAT2 and SNAT4 appear to resemble system A transporters in accumulating neutral amino acids under the influence of the sodium gradient. SNAT3 and SNAT5 appear to resemble system N transporters in utilizing proton co‐transport to accumulate amino acids. The predicted membrane topology is of 11 TM domains with an extracellular C‐terminus and intracellular N‐terminus [http://www.ncbi.nlm.nih.gov/pubmed/23506890?dopt=AbstractPlus].

## http://www.guidetopharmacology.org/GRAC/FamilyDisplayForward?familyId=225

**Table:** 

Nomenclature	http://www.guidetopharmacology.org/GRAC/ObjectDisplayForward?objectId=1169	http://www.guidetopharmacology.org/GRAC/ObjectDisplayForward?objectId=1170	http://www.guidetopharmacology.org/GRAC/ObjectDisplayForward?objectId=1171
Systematic nomenclature	SLC38A1	SLC38A2	SLC38A4
Common abbreviation	SNAT1	SNAT2	SNAT4
HGNC, UniProt	https://www.genenames.org/data/gene‐symbol‐report/#!/hgnc_id/HGNC:13447, http://www.uniprot.org/uniprot/Q9H2H9	https://www.genenames.org/data/gene‐symbol‐report/#!/hgnc_id/HGNC:13448, http://www.uniprot.org/uniprot/Q96QD8	https://www.genenames.org/data/gene‐symbol‐report/#!/hgnc_id/HGNC:14679, http://www.uniprot.org/uniprot/Q969I6
Substrates	http://www.guidetopharmacology.org/GRAC/LigandDisplayForward?ligandId=4697 http://www.guidetopharmacology.org/GRAC/LigandDisplayForward?ligandId=720 > http://www.guidetopharmacology.org/GRAC/LigandDisplayForward?ligandId=726, http://www.guidetopharmacology.org/GRAC/LigandDisplayForward?ligandId=723, http://www.guidetopharmacology.org/GRAC/LigandDisplayForward?ligandId=4533, http://www.guidetopharmacology.org/GRAC/LigandDisplayForward?ligandId=3310, http://www.guidetopharmacology.org/GRAC/LigandDisplayForward?ligandId=4782, http://www.guidetopharmacology.org/GRAC/LigandDisplayForward?ligandId=4814 > http://www.guidetopharmacology.org/GRAC/LigandDisplayForward?ligandId=727, http://www.guidetopharmacology.org/GRAC/LigandDisplayForward?ligandId=4785, http://www.guidetopharmacology.org/GRAC/LigandDisplayForward?ligandId=3314, http://www.guidetopharmacology.org/GRAC/LigandDisplayForward?ligandId=4791, http://www.guidetopharmacology.org/GRAC/LigandDisplayForward?ligandId=4794 [http://www.ncbi.nlm.nih.gov/pubmed/11692272?dopt=AbstractPlus]	http://www.guidetopharmacology.org/GRAC/LigandDisplayForward?ligandId=4697 http://www.guidetopharmacology.org/GRAC/LigandDisplayForward?ligandId=720, http://www.guidetopharmacology.org/GRAC/LigandDisplayForward?ligandId=4814 > http://www.guidetopharmacology.org/GRAC/LigandDisplayForward?ligandId=4533, http://www.guidetopharmacology.org/GRAC/LigandDisplayForward?ligandId=723, http://www.guidetopharmacology.org/GRAC/LigandDisplayForward?ligandId=726, http://www.guidetopharmacology.org/GRAC/LigandDisplayForward?ligandId=3314, http://www.guidetopharmacology.org/GRAC/LigandDisplayForward?ligandId=727 > http://www.guidetopharmacology.org/GRAC/LigandDisplayForward?ligandId=4785, http://www.guidetopharmacology.org/GRAC/LigandDisplayForward?ligandId=3312, http://www.guidetopharmacology.org/GRAC/LigandDisplayForward?ligandId=3313 [http://www.ncbi.nlm.nih.gov/pubmed/10930503?dopt=AbstractPlus]	http://www.guidetopharmacology.org/GRAC/LigandDisplayForward?ligandId=4697 http://www.guidetopharmacology.org/GRAC/LigandDisplayForward?ligandId=3310 > http://www.guidetopharmacology.org/GRAC/LigandDisplayForward?ligandId=721, http://www.guidetopharmacology.org/GRAC/LigandDisplayForward?ligandId=720, http://www.guidetopharmacology.org/GRAC/LigandDisplayForward?ligandId=4533, http://www.guidetopharmacology.org/GRAC/LigandDisplayForward?ligandId=724 > http://www.guidetopharmacology.org/GRAC/LigandDisplayForward?ligandId=727, http://www.guidetopharmacology.org/GRAC/LigandDisplayForward?ligandId=723, http://www.guidetopharmacology.org/GRAC/LigandDisplayForward?ligandId=726, http://www.guidetopharmacology.org/GRAC/LigandDisplayForward?ligandId=3314, http://www.guidetopharmacology.org/GRAC/LigandDisplayForward?ligandId=3312, http://www.guidetopharmacology.org/GRAC/LigandDisplayForward?ligandId=3313 [http://www.ncbi.nlm.nih.gov/pubmed/11342143?dopt=AbstractPlus]
Stoichiometry	1 Na^+^ : 1 amino acid (in) [http://www.ncbi.nlm.nih.gov/pubmed/11692272?dopt=AbstractPlus]	1 Na^+^ : 1 amino acid (in) [http://www.ncbi.nlm.nih.gov/pubmed/10930503?dopt=AbstractPlus]	1 Na^+^ : 1 neutral amino acid (in) [http://www.ncbi.nlm.nih.gov/pubmed/11342143?dopt=AbstractPlus]
Labelled ligands	http://www.guidetopharmacology.org/GRAC/LigandDisplayForward?ligandId=4542, http://www.guidetopharmacology.org/GRAC/LigandDisplayForward?ligandId=4543	http://www.guidetopharmacology.org/GRAC/LigandDisplayForward?ligandId=4542, http://www.guidetopharmacology.org/GRAC/LigandDisplayForward?ligandId=4543	http://www.guidetopharmacology.org/GRAC/LigandDisplayForward?ligandId=4542, http://www.guidetopharmacology.org/GRAC/LigandDisplayForward?ligandId=4635, http://www.guidetopharmacology.org/GRAC/LigandDisplayForward?ligandId=4543, http://www.guidetopharmacology.org/GRAC/LigandDisplayForward?ligandId=4084
Comments	–	–	Transport of cationic amino acids by SNAT4 was sodium‐independent [http://www.ncbi.nlm.nih.gov/pubmed/11342143?dopt=AbstractPlus].

## http://www.guidetopharmacology.org/GRAC/FamilyDisplayForward?familyId=226

**Table:** 

Nomenclature	http://www.guidetopharmacology.org/GRAC/ObjectDisplayForward?objectId=1172	http://www.guidetopharmacology.org/GRAC/ObjectDisplayForward?objectId=1173
Systematic nomenclature	SLC38A3	SLC38A5
Common abbreviation	SNAT3	SNAT5
HGNC, UniProt	https://www.genenames.org/data/gene‐symbol‐report/#!/hgnc_id/HGNC:18044, http://www.uniprot.org/uniprot/Q99624	https://www.genenames.org/data/gene‐symbol‐report/#!/hgnc_id/HGNC:18070, http://www.uniprot.org/uniprot/Q8WUX1
Substrates	http://www.guidetopharmacology.org/GRAC/LigandDisplayForward?ligandId=4697 http://www.guidetopharmacology.org/GRAC/LigandDisplayForward?ligandId=3310 , http://www.guidetopharmacology.org/GRAC/LigandDisplayForward?ligandId=723 > http://www.guidetopharmacology.org/GRAC/LigandDisplayForward?ligandId=4533, http://www.guidetopharmacology.org/GRAC/LigandDisplayForward?ligandId=720 > http://www.guidetopharmacology.org/GRAC/LigandDisplayForward?ligandId=1369 [http://www.ncbi.nlm.nih.gov/pubmed/10823827?dopt=AbstractPlus]	http://www.guidetopharmacology.org/GRAC/LigandDisplayForward?ligandId=4697 http://www.guidetopharmacology.org/GRAC/LigandDisplayForward?ligandId=4533, http://www.guidetopharmacology.org/GRAC/LigandDisplayForward?ligandId=726, http://www.guidetopharmacology.org/GRAC/LigandDisplayForward?ligandId=3310, http://www.guidetopharmacology.org/GRAC/LigandDisplayForward?ligandId=723 > http://www.guidetopharmacology.org/GRAC/LigandDisplayForward?ligandId=727, http://www.guidetopharmacology.org/GRAC/LigandDisplayForward?ligandId=720 [http://www.ncbi.nlm.nih.gov/pubmed/11243884?dopt=AbstractPlus]
Stoichiometry	1 Na^+^ : 1 amino acid (in) : 1 H^+^ (out) [http://www.ncbi.nlm.nih.gov/pubmed/11850497?dopt=AbstractPlus]	1 Na^+^ : 1 amino acid (in) : 1 H^+^ (out) [http://www.ncbi.nlm.nih.gov/pubmed/11243884?dopt=AbstractPlus]
Labelled ligands	http://www.guidetopharmacology.org/GRAC/LigandDisplayForward?ligandId=4633, http://www.guidetopharmacology.org/GRAC/LigandDisplayForward?ligandId=4634	http://www.guidetopharmacology.org/GRAC/LigandDisplayForward?ligandId=4623, http://www.guidetopharmacology.org/GRAC/LigandDisplayForward?ligandId=4670

## http://www.guidetopharmacology.org/GRAC/FamilyDisplayForward?familyId=227

**Table:** 

Nomenclature	http://www.guidetopharmacology.org/GRAC/ObjectDisplayForward?objectId=1175
Systematic nomenclature	SLC38A7
Common abbreviation	SNAT7
HGNC, UniProt	https://www.genenames.org/data/gene‐symbol‐report/#!/hgnc_id/HGNC:25582, http://www.uniprot.org/uniprot/Q9NVC3
Comments	SNAT7/SLC38A7 has been described to be a system N‐like transporter allowing preferential accumulation of glutamine (*e.g*. http://www.guidetopharmacology.org/GRAC/LigandDisplayForward?ligandId=723), histidine (*e.g*. http://www.guidetopharmacology.org/GRAC/LigandDisplayForward?ligandId=3310) and asparagine (*e.g*. http://www.guidetopharmacology.org/GRAC/LigandDisplayForward?ligandId=4533) [http://www.ncbi.nlm.nih.gov/pubmed/21511949?dopt=AbstractPlus].

### Further reading on SLC38 family of sodium‐dependent neutral amino acid transporters

Bhutia YD *et al*. (2016) Glutamine transporters inmammalian cells and their functions in physiology and cancer. *Biochim. Biophys. Acta* **1863**: 2531–9 https://www.ncbi.nlm.nih.gov/pubmed/26724577?dopt=AbstractPlus

Bröer S. (2014) The SLC38 family of sodium‐amino acid co‐transporters. *Pflugers Arch*. **466**: 155–72 https://www.ncbi.nlm.nih.gov/pubmed/24193407?dopt=AbstractPlus

Bröer S *et al.* (2011) The role of amino acid transporters in inherited and acquired diseases. *Biochem. J*. **436**: 193–211 https://www.ncbi.nlm.nih.gov/pubmed/21568940?dopt=AbstractPlus

Hägglund MG *et al*. (2011) Identification of SLC38A7 (SNAT7) protein as a glutamine transporter expressed in neurons. *J. Biol. Chem*. **286**: 20500–11 https://www.ncbi.nlm.nih.gov/pubmed/21511949?dopt=AbstractPlus

Schiöth HB *et al*. (2013) Evolutionary origin of amino acid transporter families SLC32, SLC36 and SLC38 and physiological, pathological and therapeutic aspects. *Mol. Aspects Med*. **34**: 571–85 https://www.ncbi.nlm.nih.gov/pubmed/23506890?dopt=AbstractPlus

## http://www.guidetopharmacology.org/GRAC/FamilyDisplayForward?familyId=228

### Overview

Along with the http://www.guidetopharmacology.org/GRAC/FamilyDisplayForward?familyId=217, SLC39 family members regulate zinc movement in cells. SLC39 metal ion transporters accumulate zinc into the cytosol. Membrane topology modelling suggests the presence of eight TM regions with both termini extracellular or in the lumen of intracellular organelles. The mechanism for zinc transport for many members is unknown but appears to involve co‐transport of bicarbonate ions [http://www.ncbi.nlm.nih.gov/pubmed/18270315?dopt=AbstractPlus, http://www.ncbi.nlm.nih.gov/pubmed/18037372?dopt=AbstractPlus].

**Table:** 

Nomenclature	http://www.guidetopharmacology.org/GRAC/ObjectDisplayForward?objectId=1187	http://www.guidetopharmacology.org/GRAC/ObjectDisplayForward?objectId=1193
Systematic nomenclature	SLC39A8	SLC39A14
Common abbreviation	ZIP8	ZIP14
HGNC, UniProt	https://www.genenames.org/data/gene‐symbol‐report/#!/hgnc_id/HGNC:20862, http://www.uniprot.org/uniprot/Q9C0K1	https://www.genenames.org/data/gene‐symbol‐report/#!/hgnc_id/HGNC:20858, http://www.uniprot.org/uniprot/Q15043
Substrates	http://www.guidetopharmacology.org/GRAC/LigandDisplayForward?ligandId=2440 [http://www.ncbi.nlm.nih.gov/pubmed/15722412?dopt=AbstractPlus, http://www.ncbi.nlm.nih.gov/pubmed/18037372?dopt=AbstractPlus]	http://www.guidetopharmacology.org/GRAC/LigandDisplayForward?ligandId=2440 [http://www.ncbi.nlm.nih.gov/pubmed/18270315?dopt=AbstractPlus], Mn^2+^ [http://www.ncbi.nlm.nih.gov/pubmed/18270315?dopt=AbstractPlus], Fe^2+^ [http://www.ncbi.nlm.nih.gov/pubmed/16950869?dopt=AbstractPlus]
Stoichiometry	1 Zn^2+^ (in) : 2 HCO_3_^‐^(in) [http://www.ncbi.nlm.nih.gov/pubmed/18037372?dopt=AbstractPlus]	–

### Comments

Zinc fluxes may be monitored through the use of radioisotopic Zn‐65 or the fluorescent dye FluoZin 3. The bicarbonate transport inhibitor http://www.guidetopharmacology.org/GRAC/LigandDisplayForward?ligandId=4177 has been reported to inhibit cation accumulation through ZIP14 [http://www.ncbi.nlm.nih.gov/pubmed/18270315?dopt=AbstractPlus].

### Further reading on SLC39 family of metal ion transporters

Hojyo S *et al*. (2016) Zinc transporters and signaling in physiology and pathogenesis. *Arch. Biochem. Biophys*. **611**: 43–50 https://www.ncbi.nlm.nih.gov/pubmed/27394923?dopt=AbstractPlus

Jeong J *et al*. (2013) The SLC39 family of zinc transporters. *Mol. Aspects Med*. **34**: 612–9 https://www.ncbi.nlm.nih.gov/pubmed/23506894?dopt=AbstractPlus

Kambe T *et al*. (2014) Current understanding of ZIP and ZnT zinc transporters in human health and diseases. Cell. *Mol. Life Sci*. **71**: 3281–95 https://www.ncbi.nlm.nih.gov/pubmed/24710731?dopt=AbstractPlus

Kambe T *et al*. (2015) The Physiological, Biochemical, and Molecular Roles of Zinc Transporters in Zinc Homeostasis and Metabolism. *Physiol. Rev*. **95**: 749–784 https://www.ncbi.nlm.nih.gov/pubmed/26084690?dopt=AbstractPlus

Marger L *et al*. (2014) Zinc: an underappreciated modulatory factor of brain function. *Biochem. Pharmacol*. **91**: 426–35 https://www.ncbi.nlm.nih.gov/pubmed/25130547?dopt=AbstractPlus

## http://www.guidetopharmacology.org/GRAC/FamilyDisplayForward?familyId=229

### Overview

Alongside the http://www.guidetopharmacology.org/GRAC/FamilyDisplayForward?familyId=183 of proton‐coupled metal transporters, ferroportin allows the accumulation of iron from the diet. Whilst SLC11A2 functions on the apical membrane, ferroportin acts on the basolateral side of the enterocyte, as well as regulating macrophage and placental iron levels. The predicted topology is of 12 TM domains, with intracellular termini [http://www.ncbi.nlm.nih.gov/pubmed/19150361?dopt=AbstractPlus], with the functional transporter potentially a dimeric arrangement [http://www.ncbi.nlm.nih.gov/pubmed/15667655?dopt=AbstractPlus, http://www.ncbi.nlm.nih.gov/pubmed/17077321?dopt=AbstractPlus]. Ferroportin is essential for iron homeostasis [http://www.ncbi.nlm.nih.gov/pubmed/16054062?dopt=AbstractPlus]. Ferroportin is expressed on the surface of cells that store and transport iron, such as duodenal enterocytes, hepatocytes, adipocytes and reticuloendothelial macrophages. Levels of ferroportin are regulated by its association with (binding to) hepcidin, a 25 amino acid hormone responsive to circulating iron levels (amongst other signals). Hepcidin binding targets ferroportin for internalisation and degradation, lowering the levels of iron export to the blood. Novel therapeutic agents which stabilise ferroportin or protect it from hepcidin‐induced degradation are being developed as antianemia agents. Anti‐ferroportin monoclonal antibodies are such an agent.

**Table:** 

Nomenclature	http://www.guidetopharmacology.org/GRAC/ObjectDisplayForward?objectId=1194
Systematic nomenclature	SLC40A1
Common abbreviation	IREG1
HGNC, UniProt	https://www.genenames.org/data/gene‐symbol‐report/#!/hgnc_id/HGNC:10909, http://www.uniprot.org/uniprot/Q9NP59
Endogenous substrates	Fe^2+^
Stoichiometry	Unknown
Antibodies	http://www.guidetopharmacology.org/GRAC/LigandDisplayForward?ligandId=8416 (Binding) [395]

### Comments

Hepcidin (https://www.genenames.org/data/gene‐symbol‐report/#!/hgnc_id/HGNC:15598, http://www.uniprot.org/uniprot/P81172), cleaved into http://www.guidetopharmacology.org/GRAC/LigandDisplayForward?ligandId=5378 (https://www.genenames.org/data/gene‐symbol‐report/#!/hgnc_id/HGNC:15598, http://www.uniprot.org/uniprot/P81172) and http://www.guidetopharmacology.org/GRAC/LigandDisplayForward?ligandId=5379 (https://www.genenames.org/data/gene‐symbol‐report/#!/hgnc_id/HGNC:15598, http://www.uniprot.org/uniprot/P81172), is a small protein that increases upon inflammation, binds to ferroportin to regulate its cellular distribution and degradation. Gene disruption in mice results in embryonic lethality [http://www.ncbi.nlm.nih.gov/pubmed/16054062?dopt=AbstractPlus], while loss‐of‐function mutations in man are associated with haemochromatosis [http://www.ncbi.nlm.nih.gov/pubmed/15956209?dopt=AbstractPlus].

### Further reading on SLC40 iron transporter

McKie AT *et al*. (2004) The SLC40 basolateral iron transporter family (IREG1/ferroportin/MTP1). *Pflugers Arch*. **447**: 801–6 https://www.ncbi.nlm.nih.gov/pubmed/12836025?dopt=AbstractPlus

Montalbetti N *et al*. (2013) Mammalian iron transporters: families SLC11 and SLC40. *Mol. Aspects Med*. **34**: 270–87 https://www.ncbi.nlm.nih.gov/pubmed/23506870?dopt=AbstractPlus

## http://www.guidetopharmacology.org/GRAC/FamilyDisplayForward?familyId=230

### Overview

By analogy with bacterial orthologues, this family is probably magnesium transporters. The prokaryote orthologue, MgtE, is responsible for uptake of divalent cations, while the heterologous expression studies of mammalian proteins suggest Mg^2+^ efflux [http://www.ncbi.nlm.nih.gov/pubmed/22031603?dopt=AbstractPlus], possibly as a result of co‐expression of particular protein partners (see [http://www.ncbi.nlm.nih.gov/pubmed/23506895?dopt=AbstractPlus]). Topological modelling suggests 10 TM domains with cytoplasmic C‐ and N‐ termini.

**Table:** 

Nomenclature	http://www.guidetopharmacology.org/GRAC/ObjectDisplayForward?objectId=1195	http://www.guidetopharmacology.org/GRAC/ObjectDisplayForward?objectId=1196
Systematic nomenclature	SLC41A1	SLC41A2
Common abbreviation	MgtE	–
HGNC, UniProt	https://www.genenames.org/data/gene‐symbol‐report/#!/hgnc_id/HGNC:19429, http://www.uniprot.org/uniprot/Q8IVJ1	https://www.genenames.org/data/gene‐symbol‐report/#!/hgnc_id/HGNC:31045, http://www.uniprot.org/uniprot/Q96JW4
Substrates	http://www.guidetopharmacology.org/GRAC/LigandDisplayForward?ligandId=4160 [http://www.ncbi.nlm.nih.gov/pubmed/15713785?dopt=AbstractPlus], http://www.guidetopharmacology.org/GRAC/LigandDisplayForward?ligandId=4164 [http://www.ncbi.nlm.nih.gov/pubmed/15713785?dopt=AbstractPlus], http://www.guidetopharmacology.org/GRAC/LigandDisplayForward?ligandId=2344 [http://www.ncbi.nlm.nih.gov/pubmed/15713785?dopt=AbstractPlus], http://www.guidetopharmacology.org/GRAC/LigandDisplayForward?ligandId=2440 [http://www.ncbi.nlm.nih.gov/pubmed/15713785?dopt=AbstractPlus], http://www.guidetopharmacology.org/GRAC/LigandDisplayForward?ligandId=566 [http://www.ncbi.nlm.nih.gov/pubmed/15713785?dopt=AbstractPlus], http://www.guidetopharmacology.org/GRAC/LigandDisplayForward?ligandId=708 [http://www.ncbi.nlm.nih.gov/pubmed/15713785?dopt=AbstractPlus], Sr^2+^ [http://www.ncbi.nlm.nih.gov/pubmed/15713785?dopt=AbstractPlus], Fe^2+^ [http://www.ncbi.nlm.nih.gov/pubmed/15713785?dopt=AbstractPlus]	http://www.guidetopharmacology.org/GRAC/LigandDisplayForward?ligandId=2344 [http://www.ncbi.nlm.nih.gov/pubmed/15809054?dopt=AbstractPlus], http://www.guidetopharmacology.org/GRAC/LigandDisplayForward?ligandId=708 [http://www.ncbi.nlm.nih.gov/pubmed/15809054?dopt=AbstractPlus], http://www.guidetopharmacology.org/GRAC/LigandDisplayForward?ligandId=4160 [http://www.ncbi.nlm.nih.gov/pubmed/15809054?dopt=AbstractPlus], http://www.guidetopharmacology.org/GRAC/LigandDisplayForward?ligandId=2476 [http://www.ncbi.nlm.nih.gov/pubmed/15809054?dopt=AbstractPlus], Mn^2+^ [http://www.ncbi.nlm.nih.gov/pubmed/15809054?dopt=AbstractPlus], Fe^2+^ [http://www.ncbi.nlm.nih.gov/pubmed/15809054?dopt=AbstractPlus]
Stoichiometry	Unknown	Unknown

### Further reading on SLC41 family of divalent cation transporters

Payandeh J *et al*. (2013) The structure and regulation of magnesium selective ion channels. *Biochim. Biophys. Acta* **1828**: 2778‐92 https://www.ncbi.nlm.nih.gov/pubmed/23954807?dopt=AbstractPlus

Sahni J *et al*. (2013) The SLC41 family of MgtE‐like magnesium transporters. *Mol. Aspects Med*. **34**: 620‐8 https://www.ncbi.nlm.nih.gov/pubmed/23506895?dopt=AbstractPlus

Schweigel‐Röntgen M *et al*. (2014) SLC41 transporters–molecular identification and functional role. *Curr Top Membr* **73**: 383‐410 https://www.ncbi.nlm.nih.gov/pubmed/24745990?dopt=AbstractPlus

## http://www.guidetopharmacology.org/GRAC/FamilyDisplayForward?familyId=231

### Overview

Rhesus is commonly defined as a ’factor’ that determines, in part, blood type, and whether neonates suffer from haemolytic disease of the newborn. These glycoprotein antigens derive from two genes, https://www.genenames.org/data/gene‐symbol‐report/#!/hgnc_id/HGNC:10008 (http://www.uniprot.org/uniprot/P18577) and https://www.genenames.org/data/gene‐symbol‐report/#!/hgnc_id/HGNC:10009 (http://www.uniprot.org/uniprot/Q02161), expressed on the surface of erythrocytes. On erythrocytes, RhAG associates with these antigens and functions as an ammonium transporter. RhBG and RhBG are non‐erythroid related sequences associated with epithelia. Topological modelling suggests the presence of 12TM with cytoplasmic N‐ and C‐ termini. The majority of information on these transporters derives from orthologues in yeast, plants and bacteria. More recent evidence points to family members being permeable to carbon dioxide, leading to the term gas channels.

**Table:** 

Nomenclature	http://www.guidetopharmacology.org/GRAC/ObjectDisplayForward?objectId=1198	http://www.guidetopharmacology.org/GRAC/ObjectDisplayForward?objectId=1199	http://www.guidetopharmacology.org/GRAC/ObjectDisplayForward?objectId=1200
Systematic nomenclature	SLC42A1	SLC42A2	SLC42A3
Common abbreviation	RhAG	RhBG	RhCG
HGNC, UniProt	https://www.genenames.org/data/gene‐symbol‐report/#!/hgnc_id/HGNC:10006, http://www.uniprot.org/uniprot/Q02094	https://www.genenames.org/data/gene‐symbol‐report/#!/hgnc_id/HGNC:14572, http://www.uniprot.org/uniprot/Q9H310	https://www.genenames.org/data/gene‐symbol‐report/#!/hgnc_id/HGNC:18140, http://www.uniprot.org/uniprot/Q9UBD6
Substrates	NH_4_^+^ [http://www.ncbi.nlm.nih.gov/pubmed/11861637?dopt=AbstractPlus], NH_3_ [http://www.ncbi.nlm.nih.gov/pubmed/15572441?dopt=AbstractPlus], CO_2_ [http://www.ncbi.nlm.nih.gov/pubmed/17712059?dopt=AbstractPlus]	–	NH_3_ [http://www.ncbi.nlm.nih.gov/pubmed/19553567?dopt=AbstractPlus]
Stoichiometry	Unknown	Unknown	Unknown
Labelled ligands	http://www.guidetopharmacology.org/GRAC/LigandDisplayForward?ligandId=4624 (Binding) [http://www.ncbi.nlm.nih.gov/pubmed/12846905?dopt=AbstractPlus]	–	http://www.guidetopharmacology.org/GRAC/LigandDisplayForward?ligandId=4624 (Binding) [http://www.ncbi.nlm.nih.gov/pubmed/16131648?dopt=AbstractPlus] – Mouse

### Further reading on SLC42 family of Rhesus glycoprotein ammonium transporters

Nakhoul NL *et al*. (2013) Characteristics of mammalian Rh glycoproteins (SLC42 transporters) and their role in acid‐base transport. *Mol. Aspects Med*. **34**: 629‐37 https://www.ncbi.nlm.nih.gov/pubmed/23506896?dopt=AbstractPlus

Weiner ID *et al*. (2011) Role of NH_3_ and NH_4_^+^ transporters in renal acid‐base transport. *Am. J. Physiol. Renal Physiol*. **300**: F11‐23 https://www.ncbi.nlm.nih.gov/pubmed/21048022?dopt=AbstractPlus

Weiner ID *et al*. (2014) Ammonia transport in the kidney by Rhesus glycoproteins. *Am. J. Physiol. Renal Physiol*. **306**: F1107‐20 https://www.ncbi.nlm.nih.gov/pubmed/24647713?dopt=AbstractPlus

## http://www.guidetopharmacology.org/GRAC/FamilyDisplayForward?familyId=232

### Overview

LAT3 (SLC43A1) and LAT4 (SLC43A2) are transporters with system L amino acid transporter activity, along with the structurally and functionally distinct transporters LAT1 and LAT2 that are members of the http://www.guidetopharmacology.org/GRAC/FamilyDisplayForward?familyId=141#SLC7 family. LAT3 and LAT4 contain 12 put.ative TM domains with both N and C termini located intracellularly. They transport neutral amino acids in a manner independent of Na^+^ and Cl^‐^ and with two kinetic components [http://www.ncbi.nlm.nih.gov/pubmed/12930836?dopt=AbstractPlus, http://www.ncbi.nlm.nih.gov/pubmed/15659399?dopt=AbstractPlus]. LAT3/SLC43A1 is expressed in human tissues at high levels in the pancreas, liver, skeletal muscle and fetal liver [http://www.ncbi.nlm.nih.gov/pubmed/12930836?dopt=AbstractPlus] whereas LAT4/SLC43A2 is primarily expressed in the placenta, kidney and peripheral blood leukocytes [http://www.ncbi.nlm.nih.gov/pubmed/15659399?dopt=AbstractPlus]. SLC43A3 is expressed in vascular endothelial cells [http://www.ncbi.nlm.nih.gov/pubmed/18483404?dopt=AbstractPlus] but remains to be characterised.

**Table:** 

Nomenclature	http://www.guidetopharmacology.org/GRAC/ObjectDisplayForward?objectId=1201	http://www.guidetopharmacology.org/GRAC/ObjectDisplayForward?objectId=1202
Systematic nomenclature	SLC43A1	SLC43A2
Common abbreviation	LAT3	LAT4
HGNC, UniProt	https://www.genenames.org/data/gene‐symbol‐report/#!/hgnc_id/HGNC:9225, http://www.uniprot.org/uniprot/O75387	https://www.genenames.org/data/gene‐symbol‐report/#!/hgnc_id/HGNC:23087, http://www.uniprot.org/uniprot/Q8N370
Substrates	http://www.guidetopharmacology.org/GRAC/LigandDisplayForward?ligandId=3311 [http://www.ncbi.nlm.nih.gov/pubmed/12930836?dopt=AbstractPlus], http://www.guidetopharmacology.org/GRAC/LigandDisplayForward?ligandId=4750 [http://www.ncbi.nlm.nih.gov/pubmed/12930836?dopt=AbstractPlus], http://www.guidetopharmacology.org/GRAC/LigandDisplayForward?ligandId=4751 [http://www.ncbi.nlm.nih.gov/pubmed/12930836?dopt=AbstractPlus], http://www.guidetopharmacology.org/GRAC/LigandDisplayForward?ligandId=4752 [http://www.ncbi.nlm.nih.gov/pubmed/12930836?dopt=AbstractPlus], http://www.guidetopharmacology.org/GRAC/LigandDisplayForward?ligandId=3312 [http://www.ncbi.nlm.nih.gov/pubmed/12930836?dopt=AbstractPlus], http://www.guidetopharmacology.org/GRAC/LigandDisplayForward?ligandId=3313 [http://www.ncbi.nlm.nih.gov/pubmed/12930836?dopt=AbstractPlus], http://www.guidetopharmacology.org/GRAC/LigandDisplayForward?ligandId=4794 [http://www.ncbi.nlm.nih.gov/pubmed/12930836?dopt=AbstractPlus], http://www.guidetopharmacology.org/GRAC/LigandDisplayForward?ligandId=4814 [http://www.ncbi.nlm.nih.gov/pubmed/12930836?dopt=AbstractPlus]	http://www.guidetopharmacology.org/GRAC/LigandDisplayForward?ligandId=3311, http://www.guidetopharmacology.org/GRAC/LigandDisplayForward?ligandId=4750, http://www.guidetopharmacology.org/GRAC/LigandDisplayForward?ligandId=4751, http://www.guidetopharmacology.org/GRAC/LigandDisplayForward?ligandId=3312, http://www.guidetopharmacology.org/GRAC/LigandDisplayForward?ligandId=3313, http://www.guidetopharmacology.org/GRAC/LigandDisplayForward?ligandId=4794, http://www.guidetopharmacology.org/GRAC/LigandDisplayForward?ligandId=4814
Stoichiometry	Operates by facilitative diffusion	Operates by facilitative diffusion

### Comments

Covalent modification of LAT3 by http://www.guidetopharmacology.org/GRAC/LigandDisplayForward?ligandId=5335 inhibits its function [http://www.ncbi.nlm.nih.gov/pubmed/12930836?dopt=AbstractPlus] and at LAT4 inhibits the low‐, but not high‐affinity component of transport [http://www.ncbi.nlm.nih.gov/pubmed/15659399?dopt=AbstractPlus].

### Further reading on SLC43 family of large neutral amino acid transporters

Bodoy S *et al*. (2013) The small SLC43 family: facilitator system l amino acid transporters and the orphan EEG1. *Mol. Aspects Med*. **34**: 638‐45 https://www.ncbi.nlm.nih.gov/pubmed/23268354?dopt=AbstractPlus

## http://www.guidetopharmacology.org/GRAC/FamilyDisplayForward?familyId=233

### Overview

Members of the choline transporter‐like family are encoded by five genes (CTL1‐CTL5) with further diversity occurring through alternative splicing of CTL1, 4 and 5 [http://www.ncbi.nlm.nih.gov/pubmed/15715662?dopt=AbstractPlus]. CTL family members are putative 10TM domain proteins with extracellular termini that mediate Na^+^‐independent transport of http://www.guidetopharmacology.org/GRAC/LigandDisplayForward?ligandId=4551 with an affinity that is intermediate to that of the high affinity choline transporter CHT1 (http://www.guidetopharmacology.org/GRAC/FamilyDisplayForward?familyId=143#show_object_914) and the low affinity organiccation transporters [OCT1 (http://www.guidetopharmacology.org/GRAC/FamilyDisplayForward?familyId=146#show_object_1019) andOCT2 (http://www.guidetopharmacology.org/GRAC/FamilyDisplayForward?familyId=146#show_object_1020)] [http://www.ncbi.nlm.nih.gov/pubmed/16636297?dopt=AbstractPlus]. CLT1 is expressed almost ubiquitously in human tissues [http://www.ncbi.nlm.nih.gov/pubmed/11698453?dopt=AbstractPlus] and mediates http://www.guidetopharmacology.org/GRAC/LigandDisplayForward?ligandId=4551 transport across the plasma and mitochondrial membranes [http://www.ncbi.nlm.nih.gov/pubmed/19357133?dopt=AbstractPlus]. Transport of http://www.guidetopharmacology.org/GRAC/LigandDisplayForward?ligandId=4551 by CTL2, which in rodents is expressed as two isoforms (CTL2P1 and CLTP2; [http://www.ncbi.nlm.nih.gov/pubmed/20665236?dopt=AbstractPlus]) in lung, colon, inner ear and spleen and to a lesser extent in brain, tongue, liver, and kidney, has only recently been demonstrated [http://www.ncbi.nlm.nih.gov/pubmed/20665236?dopt=AbstractPlus, http://www.ncbi.nlm.nih.gov/pubmed/20410607?dopt=AbstractPlus]. CTL3‐5 remain to be characterized functionally.

**Table:** 

Nomenclature	http://www.guidetopharmacology.org/GRAC/ObjectDisplayForward?objectId=1204
Systematic nomenclature	SLC44A1
Common abbreviation	CTL1
HGNC, UniProt	https://www.genenames.org/data/gene‐symbol‐report/#!/hgnc_id/HGNC:18798, http://www.uniprot.org/uniprot/Q8WWI5
Substrates	http://www.guidetopharmacology.org/GRAC/LigandDisplayForward?ligandId=4551
Stoichiometry	Unknown: uptake enhanced in the absence of extracellular Na^+^, reduced by membrane depolarization, extracellular acidification and collapse of plasma membrane H^+^ electrochemical gradient
Inhibitors	http://www.guidetopharmacology.org/GRAC/LigandDisplayForward?ligandId=4494 (p*K*_i_ 3.5–4.5)

### Comments

Data tabulated are features observed for CLT1 endogenous to: rat astrocytes [http://www.ncbi.nlm.nih.gov/pubmed/16000150?dopt=AbstractPlus]; rat renal tubule epithelial cells [http://www.ncbi.nlm.nih.gov/pubmed/19236841?dopt=AbstractPlus]; human colon carcinoma cells [http://www.ncbi.nlm.nih.gov/pubmed/19135976?dopt=AbstractPlus]; human keratinocytes [http://www.ncbi.nlm.nih.gov/pubmed/19122366?dopt=AbstractPlus] and human neuroblastoma cells [http://www.ncbi.nlm.nih.gov/pubmed/21185344?dopt=AbstractPlus]. Choline uptake by CLT1 is inhibited by numerous organic cations (*e.g*. [http://www.ncbi.nlm.nih.gov/pubmed/16000150?dopt=AbstractPlus, http://www.ncbi.nlm.nih.gov/pubmed/19236841?dopt=AbstractPlus, http://www.ncbi.nlm.nih.gov/pubmed/21185344?dopt=AbstractPlus]). In the guinea‐pig, CTL2 is a target for antibody‐induced hearing loss [http://www.ncbi.nlm.nih.gov/pubmed/14973250?dopt=AbstractPlus] and in man, a polymorphism in CTL2 constitutes the human neutrophil alloantigen‐3a (HNA‐3a; [http://www.ncbi.nlm.nih.gov/pubmed/20037594?dopt=AbstractPlus]).

### Further reading on SLC44 choline transporter‐like family

Inazu M. (2014) Choline transporter‐like proteins CTLs/SLC44 family as a novel molecular target for cancer therapy. *Biopharm Drug Dispos* **35**: 431‐49 https://www.ncbi.nlm.nih.gov/pubmed/24532461?dopt=AbstractPlus

Traiffort E *et al*. (2013) The choline transporter‐like family SLC44: properties and roles in human diseases. *Mol. Aspects Med*. **34**: 646‐54 https://www.ncbi.nlm.nih.gov/pubmed/23506897?dopt=AbstractPlus

## http://www.guidetopharmacology.org/GRAC/FamilyDisplayForward?familyId=234

### Overview

Members of the SLC45 family remain to be fully characterised. SLC45A1 was initially identified in the rat brain, particularly predominant in the hindbrain, as a proton‐associated sugar transport, induced by hypercapnia [http://www.ncbi.nlm.nih.gov/pubmed/12417639?dopt=AbstractPlus]. The protein is predicted to have 12TM domains, with intracellular termini. The *SLC45A2* gene is thought to encode a transporter protein that mediates http://www.guidetopharmacology.org/GRAC/LigandDisplayForward?ligandId=5415 synthesis. Mutations in SLC45A2 are a cause of oculocutaneous albinism type 4 (*e.g*. [http://www.ncbi.nlm.nih.gov/pubmed/11574907?dopt=AbstractPlus]), and polymorphisms in this gene are associated with variations in skin and hair color (*e.g*. [http://www.ncbi.nlm.nih.gov/pubmed/15714523?dopt=AbstractPlus]).

**Table:** 

Nomenclature	http://www.guidetopharmacology.org/GRAC/ObjectDisplayForward?objectId=1209
Systematic nomenclature	SLC45A1
HGNC, UniProt	https://www.genenames.org/data/gene‐symbol‐report/#!/hgnc_id/HGNC:17939, http://www.uniprot.org/uniprot/Q9Y2W3
Substrates	http://www.guidetopharmacology.org/GRAC/LigandDisplayForward?ligandId=4719 [http://www.ncbi.nlm.nih.gov/pubmed/12417639?dopt=AbstractPlus], Galactose [http://www.ncbi.nlm.nih.gov/pubmed/12417639?dopt=AbstractPlus]
Stoichiometry	Unknown; increased at acid pH [http://www.ncbi.nlm.nih.gov/pubmed/12417639?dopt=AbstractPlus].

### Further reading on SLC45 family of putative sugar transporters

Bartölke R *et al*. (2014) Proton‐associated sucrose transport of mammalian solute carrier family 45: an analysis in Saccharomyces cerevisiae. *Biochem. J*. **464**: 193‐201 https://www.ncbi.nlm.nih.gov/pubmed/25164149?dopt=AbstractPlus

Vitavska O *et al*. (2013) The SLC45 gene family of putative sugar transporters. *Mol. Aspects Med*. **34**: 655‐60 https://www.ncbi.nlm.nih.gov/pubmed/23506898?dopt=AbstractPlus

## http://www.guidetopharmacology.org/GRAC/FamilyDisplayForward?familyId=235

### Overview

Based on the proptypicalmember of this family, PCFT, this family includes proton‐driven transporters with 11 TMsegments. SLC46A1 has been described to act as an intestinal proton‐coupled high‐affinity http://www.guidetopharmacology.org/GRAC/LigandDisplayForward?ligandId=4563 transporter [http://www.ncbi.nlm.nih.gov/pubmed/17129779?dopt=AbstractPlus], with lower affinity for http://www.guidetopharmacology.org/GRAC/LigandDisplayForward?ligandId=4349. http://www.guidetopharmacology.org/GRAC/LigandDisplayForward?ligandId=4563 accumulation is independent of Na^+^ or K^+^ ion concentrations, but driven by extracellular protons with an as yet undefined stoichiometry.

**Table:** 

Nomenclature	http://www.guidetopharmacology.org/GRAC/ObjectDisplayForward?objectId=1213
Systematic nomenclature	SLC46A1
Common abbreviation	PCFT
HGNC, UniProt	https://www.genenames.org/data/gene‐symbol‐report/#!/hgnc_id/HGNC:30521, http://www.uniprot.org/uniprot/Q96NT5
Substrates	pemetrexed, N‐formyltetrahydrofolate, http://www.guidetopharmacology.org/GRAC/LigandDisplayForward?ligandId=4815 [http://www.ncbi.nlm.nih.gov/pubmed/17129779?dopt=AbstractPlus] http://www.guidetopharmacology.org/GRAC/LigandDisplayForward?ligandId=4563 (1.3μM) > http://www.guidetopharmacology.org/GRAC/LigandDisplayForward?ligandId=4349 (>100 μM) [http://www.ncbi.nlm.nih.gov/pubmed/17475902?dopt=AbstractPlus]
Endogenous substrates	N^5^‐methyltetrafolate [http://www.ncbi.nlm.nih.gov/pubmed/17129779?dopt=AbstractPlus]
Labelled ligands	http://www.guidetopharmacology.org/GRAC/LigandDisplayForward?ligandId=6689 (Binding), http://www.guidetopharmacology.org/GRAC/LigandDisplayForward?ligandId=4562, http://www.guidetopharmacology.org/GRAC/LigandDisplayForward?ligandId=6690 (Binding), http://www.guidetopharmacology.org/GRAC/LigandDisplayForward?ligandId=4674, http://www.guidetopharmacology.org/GRAC/LigandDisplayForward?ligandId=6688 (Binding)
Comments	Loss‐of‐function mutations in PCFT (SLC46A1) are the molecular basis for hereditary folate maladsorption [http://www.ncbi.nlm.nih.gov/pubmed/21346251?dopt=AbstractPlus].

### Further reading on SLC46 family of folate transporters

Hou Z *et al*. (2014) Biology of the major facilitative folate transporters SLC19A1 and SLC46A1. *Curr Top Membr* **73**: 175‐204 https://www.ncbi.nlm.nih.gov/pubmed/24745983?dopt=AbstractPlus

Matherly LH *et al*. (2014) The major facilitative folate transporters solute carrier 19A1 and solute carrier 46A1: biology and role in antifolate chemotherapy of cancer. *Drug Metab. Dispos*. **42**: 632‐49 https://www.ncbi.nlm.nih.gov/pubmed/24396145?dopt=AbstractPlus

Wilson MR *et al*. (2015) Structural determinants of human proton‐coupled folate transporter oligomerization: role of GXXXG motifs and identification of oligomeric interfaces at transmembrane domains 3 and 6. *Biochem. J*. **469**: 33‐44 https://www.ncbi.nlm.nih.gov/pubmed/25877470?dopt=AbstractPlus

Zhao R *et al*. (2011) Mechanisms of membrane transport of folates into cells and across epithelia. *Annu. Rev. Nutr*. **31**: 177‐201 https://www.ncbi.nlm.nih.gov/pubmed/21568705?dopt=AbstractPlus

Zhao R *et al*. (2013) Folate and thiamine transporters mediated by facilitative carriers (SLC19A1‐3 and SLC46A1) and folate receptors. *Mol. Aspects Med*. **34**: 373‐85 https://www.ncbi.nlm.nih.gov/pubmed/23506878?dopt=AbstractPlus

## http://www.guidetopharmacology.org/GRAC/FamilyDisplayForward?familyId=236

### Overview

These proton:organic cation exchangers are predicted to have 13 TM segments [http://www.ncbi.nlm.nih.gov/pubmed/19515813?dopt=AbstractPlus] and are suggested to be responsible for excretion of many drugs in the liver and kidneys.

**Table:** 

Nomenclature	http://www.guidetopharmacology.org/GRAC/ObjectDisplayForward?objectId=1216	http://www.guidetopharmacology.org/GRAC/ObjectDisplayForward?objectId=1217
Systematic nomenclature	SLC47A1	SLC47A2
Common abbreviation	MATE1	MATE2‐K
HGNC, UniProt	https://www.genenames.org/data/gene‐symbol‐report/#!/hgnc_id/HGNC:25588, http://www.uniprot.org/uniprot/Q96FL8	https://www.genenames.org/data/gene‐symbol‐report/#!/hgnc_id/HGNC:26439, http://www.uniprot.org/uniprot/Q86VL8
Substrates	http://www.guidetopharmacology.org/GRAC/LigandDisplayForward?ligandId=2342 [http://www.ncbi.nlm.nih.gov/pubmed/17509534?dopt=AbstractPlus], http://www.guidetopharmacology.org/GRAC/LigandDisplayForward?ligandId=4830 [http://www.ncbi.nlm.nih.gov/pubmed/17509534?dopt=AbstractPlus], http://www.guidetopharmacology.org/GRAC/LigandDisplayForward?ligandId=4779 (*K*_m_ 7.8×10^‐4^M) [http://www.ncbi.nlm.nih.gov/pubmed/17509534?dopt=AbstractPlus], http://www.guidetopharmacology.org/GRAC/LigandDisplayForward?ligandId=4832 [http://www.ncbi.nlm.nih.gov/pubmed/17509534?dopt=AbstractPlus], http://www.guidetopharmacology.org/GRAC/LigandDisplayForward?ligandId=1231 (*K*_m_ 1.7×10^‐4^M) [http://www.ncbi.nlm.nih.gov/pubmed/16928787?dopt=AbstractPlus, http://www.ncbi.nlm.nih.gov/pubmed/17509534?dopt=AbstractPlus], http://www.guidetopharmacology.org/GRAC/LigandDisplayForward?ligandId=4552 [http://www.ncbi.nlm.nih.gov/pubmed/17495125?dopt=AbstractPlus]	http://www.guidetopharmacology.org/GRAC/LigandDisplayForward?ligandId=4783 [http://www.ncbi.nlm.nih.gov/pubmed/17509534?dopt=AbstractPlus], http://www.guidetopharmacology.org/GRAC/LigandDisplayForward?ligandId=4811 [http://www.ncbi.nlm.nih.gov/pubmed/16807400?dopt=AbstractPlus], http://www.guidetopharmacology.org/GRAC/LigandDisplayForward?ligandId=4779 (*K*_m_ 1.9×10^‐3^M) [http://www.ncbi.nlm.nih.gov/pubmed/16807400?dopt=AbstractPlus, http://www.ncbi.nlm.nih.gov/pubmed/17509534?dopt=AbstractPlus], http://www.guidetopharmacology.org/GRAC/LigandDisplayForward?ligandId=4829 [http://www.ncbi.nlm.nih.gov/pubmed/17509534?dopt=AbstractPlus], http://www.guidetopharmacology.org/GRAC/LigandDisplayForward?ligandId=4568 [http://www.ncbi.nlm.nih.gov/pubmed/16807400?dopt=AbstractPlus], http://www.guidetopharmacology.org/GRAC/LigandDisplayForward?ligandId=1231 (*K*_m_ 1.2×10^‐4^M) [http://www.ncbi.nlm.nih.gov/pubmed/16807400?dopt=AbstractPlus, http://www.ncbi.nlm.nih.gov/pubmed/17509534?dopt=AbstractPlus], http://www.guidetopharmacology.org/GRAC/LigandDisplayForward?ligandId=4658 [http://www.ncbi.nlm.nih.gov/pubmed/16807400?dopt=AbstractPlus]
Endogenous substrates	http://www.guidetopharmacology.org/GRAC/LigandDisplayForward?ligandId=4629 [http://www.ncbi.nlm.nih.gov/pubmed/17509534?dopt=AbstractPlus], http://www.guidetopharmacology.org/GRAC/LigandDisplayForward?ligandId=4496 [http://www.ncbi.nlm.nih.gov/pubmed/17509534?dopt=AbstractPlus]	http://www.guidetopharmacology.org/GRAC/LigandDisplayForward?ligandId=4496 [http://www.ncbi.nlm.nih.gov/pubmed/17509534?dopt=AbstractPlus], http://www.guidetopharmacology.org/GRAC/LigandDisplayForward?ligandId=4629 [http://www.ncbi.nlm.nih.gov/pubmed/17509534?dopt=AbstractPlus]
Sub/family‐selective inhibitors	http://www.guidetopharmacology.org/GRAC/LigandDisplayForward?ligandId=4800 (p*K*_i_ 7.1) [http://www.ncbi.nlm.nih.gov/pubmed/20065018?dopt=AbstractPlus], http://www.guidetopharmacology.org/GRAC/LigandDisplayForward?ligandId=1231 (p*K*_i_ 6) [http://www.ncbi.nlm.nih.gov/pubmed/19164462?dopt=AbstractPlus]	http://www.guidetopharmacology.org/GRAC/LigandDisplayForward?ligandId=4800 (p*K*_i_ 6.3) [http://www.ncbi.nlm.nih.gov/pubmed/20065018?dopt=AbstractPlus] – Mouse, http://www.guidetopharmacology.org/GRAC/LigandDisplayForward?ligandId=1231 (p*K*_i_ 5.1) [http://www.ncbi.nlm.nih.gov/pubmed/19164462?dopt=AbstractPlus]
Labelled ligands	http://www.guidetopharmacology.org/GRAC/LigandDisplayForward?ligandId=4575 [http://www.ncbi.nlm.nih.gov/pubmed/16330770?dopt=AbstractPlus, http://www.ncbi.nlm.nih.gov/pubmed/16850272?dopt=AbstractPlus], http://www.guidetopharmacology.org/GRAC/LigandDisplayForward?ligandId=4503 [http://www.ncbi.nlm.nih.gov/pubmed/17509534?dopt=AbstractPlus, http://www.ncbi.nlm.nih.gov/pubmed/16850272?dopt=AbstractPlus]	http://www.guidetopharmacology.org/GRAC/LigandDisplayForward?ligandId=4575 [http://www.ncbi.nlm.nih.gov/pubmed/17509534?dopt=AbstractPlus], http://www.guidetopharmacology.org/GRAC/LigandDisplayForward?ligandId=4503 [http://www.ncbi.nlm.nih.gov/pubmed/17509534?dopt=AbstractPlus]

### Comments

DAPI has been used to allow quantification of MATE1 and MATE2‐mediated transport activity [http://www.ncbi.nlm.nih.gov/pubmed/20047987?dopt=AbstractPlus]. MATE2 and MATE2‐B are inactive splice variants of MATE2‐K [http://www.ncbi.nlm.nih.gov/pubmed/16807400?dopt=AbstractPlus].

### Further reading on SLC47 family of multidrug and toxin extrusion transporters

Damme K *et al*. (2011) Mammalian MATE (SLC47A) transport proteins: impact on efflux of endogenous substrates and xenobiotics. *Drug Metab. Rev*. **43**: 499‐523 https://www.ncbi.nlm.nih.gov/pubmed/21923552?dopt=AbstractPlus

Motohashi H *et al*. (2013) Multidrug and toxin extrusion family SLC47: physiological, pharmacokinetic and toxicokinetic importance of MATE1 and MATE2‐K. *Mol. Aspects Med*. **34**: 661‐8 https://www.ncbi.nlm.nih.gov/pubmed/23506899?dopt=AbstractPlus

Nies AT *et al*. (2016) Structure and function of multidrug and toxin extrusion proteins (MATEs) and their relevance to drug therapy and personalized medicine. *Arch. Toxicol*. **90**: 1555‐84 https://www.ncbi.nlm.nih.gov/pubmed/27165417?dopt=AbstractPlus

Wagner DJ *et al*. (2016) Polyspecific organic cation transporters and their impact on drug intracellular levels and pharmacodynamics. *Pharmacol. Res*. **111**: 237‐246 https://www.ncbi.nlm.nih.gov/pubmed/27317943?dopt=AbstractPlus

Yonezawa A *et al*. (2011) Importance of the multidrug and toxin extrusion MATE/SLC47A family to pharmacokinetics, pharmacodynamics/toxicodynamics and pharmacogenomics. *Br. J. Pharmacol*. **164**: 1817‐25 https://www.ncbi.nlm.nih.gov/pubmed/21457222?dopt=AbstractPlus

## http://www.guidetopharmacology.org/GRAC/FamilyDisplayForward?familyId=237

### Overview

HRG1 has been identified as a cell surface and lysosomal heme transporter [http://www.ncbi.nlm.nih.gov/pubmed/18418376?dopt=AbstractPlus]. In addition, evidence suggests this 4TM‐containing protein associates with the http://www.guidetopharmacology.org/GRAC/FamilyDisplayForward?familyId=137#V‐type ATPase in lysosomes [http://www.ncbi.nlm.nih.gov/pubmed/19875448?dopt=AbstractPlus]. Recent studies confirm its lysosomal location and demonstrate that it has an important physiological function in macrophages ingesting senescent red blood cells (erythrophagocytosis), recycling heme (released from the red cell hemoglobin) from the phagolysosome into the cytosol, where the heme is subsequently catabolized to recycle the iron [http://www.ncbi.nlm.nih.gov/pubmed/23395172?dopt=AbstractPlus].

**Table:** 

Nomenclature	http://www.guidetopharmacology.org/GRAC/ObjectDisplayForward?objectId=1218
Systematic nomenclature	SLC48A1
Common abbreviation	HRG1
HGNC, UniProt	https://www.genenames.org/data/gene‐symbol‐report/#!/hgnc_id/HGNC:26035, http://www.uniprot.org/uniprot/Q6P1K1

### Further reading on SLC48 heme transporter

Khan AA *et al*. (2013) Heme and FLVCR‐related transporter families SLC48 and SLC49. *Mol. Aspects Med*. **34**: 669‐82 https://www.ncbi.nlm.nih.gov/pubmed/23506900?dopt=AbstractPlus

## http://www.guidetopharmacology.org/GRAC/FamilyDisplayForward?familyId=335

### Overview

FLVCR1 was initially identified as a cell‐surface attachment site for feline leukemia virus subgroup C [http://www.ncbi.nlm.nih.gov/pubmed/10400745?dopt=AbstractPlus], and later identified as a cell surface accumulation which exports heme from the cytosol [http://www.ncbi.nlm.nih.gov/pubmed/15369674?dopt=AbstractPlus]. A recent study indicates that an isoform of FLVCR1 is located in the mitochondria, the site of the final steps of heme synthesis, and appears to transport heme into the cytosol [http://www.ncbi.nlm.nih.gov/pubmed/23187127?dopt=AbstractPlus]. FLVCR‐mediated heme transport is essential for erythropoiesis. Flvcr1 gene mutations have been identified as the cause of PCARP (http://www.omim.org/entry/609033?search=609033&highlight=609033 (PCARP) [http://www.ncbi.nlm.nih.gov/pubmed/21070897?dopt=AbstractPlus].There are three paralogs of FLVCR1 in the human genome.

FLVCR2, most similar to FLVCR1 [http://www.ncbi.nlm.nih.gov/pubmed/11943475?dopt=AbstractPlus], has been reported to function as a heme importer [http://www.ncbi.nlm.nih.gov/pubmed/20823265?dopt=AbstractPlus]. In addition, a congenital syndrome of proliferative vasculopathy and hydranencephaly, also known as Fowler&apos;s syndrome, is associated with a loss‐of‐function mutation in FLVCR2 [http://www.ncbi.nlm.nih.gov/pubmed/20206334?dopt=AbstractPlus].

The functions of the other two members of the SLC49 family, MFSD7 and DIRC2, are unknown, although DIRC2 has been implicated in hereditary renal carcinomas [http://www.ncbi.nlm.nih.gov/pubmed/11912179?dopt=AbstractPlus].

**Table:** 

Nomenclature	http://www.guidetopharmacology.org/GRAC/ObjectDisplayForward?objectId=1910	http://www.guidetopharmacology.org/GRAC/ObjectDisplayForward?objectId=1911
Systematic nomenclature	SLC49A1	SLC49A2
Common abbreviation	FLVCR1	FLVCR2
HGNC, UniProt	https://www.genenames.org/data/gene‐symbol‐report/#!/hgnc_id/HGNC:24682, http://www.uniprot.org/uniprot/Q9Y5Y0	https://www.genenames.org/data/gene‐symbol‐report/#!/hgnc_id/HGNC:20105, http://www.uniprot.org/uniprot/Q9UPI3
Substrates	http://www.guidetopharmacology.org/GRAC/LigandDisplayForward?ligandId=4349 [http://www.ncbi.nlm.nih.gov/pubmed/15369674?dopt=AbstractPlus]	http://www.guidetopharmacology.org/GRAC/LigandDisplayForward?ligandId=4349 [http://www.ncbi.nlm.nih.gov/pubmed/20823265?dopt=AbstractPlus]
Stoichiometry	Unknown	Unknown

### Comments

Non‐functional splice alternatives of FLVCR1 have been implicated as a cause of a congenital red cell aplasia, http://omim.org/entry/105650 [http://www.ncbi.nlm.nih.gov/pubmed/18815190?dopt=AbstractPlus].

### Further reading on SLC49 family of FLVCR‐related heme transporters

Khan AA *et al*. (2013) Heme and FLVCR‐related transporter families SLC48 and SLC49. *Mol. Aspects Med*. **34**: 669‐82 https://www.ncbi.nlm.nih.gov/pubmed/23506900?dopt=AbstractPlus

Khan AA *et al*. (2011) Control of intracellular heme levels: heme transporters and heme oxygenases. *Biochim. Biophys. Acta* **1813**: 668‐82 https://www.ncbi.nlm.nih.gov/pubmed/21238504?dopt=AbstractPlus

## http://www.guidetopharmacology.org/GRAC/FamilyDisplayForward?familyId=336

### Overview

A mouse stromal cell cDNA library was used to clone C2.3 [http://www.ncbi.nlm.nih.gov/pubmed/8630032?dopt=AbstractPlus], later termed Rag1‐activating protein 1, with a sequence homology predictive of a 4TM topology. The plant orthologues, termed SWEETs, appear to be 7 TM proteins, with extracellular N‐termini, and the capacity for bidirectional flux of http://www.guidetopharmacology.org/GRAC/LigandDisplayForward?ligandId=4536 [http://www.ncbi.nlm.nih.gov/pubmed/21107422?dopt=AbstractPlus]. Expression of mouse SWEET in the mammary gland was suggestive of a role in Golgi lactose synthesis [http://www.ncbi.nlm.nih.gov/pubmed/21107422?dopt=AbstractPlus].

**Table:** 

Nomenclature	http://www.guidetopharmacology.org/GRAC/ObjectDisplayForward?objectId=1914
Systematic nomenclature	SLC50A1
Common abbreviation	RAG1AP1
HGNC, UniProt	https://www.genenames.org/data/gene‐symbol‐report/#!/hgnc_id/HGNC:30657, http://www.uniprot.org/uniprot/Q9BRV3

### Further reading on SLC50 sugar transporter

Wright EM. (2013) Glucose transport families SLC5 and SLC50. *Mol. Aspects Med*. **34**: 183‐96 https://www.ncbi.nlm.nih.gov/pubmed/23506865?dopt=AbstractPlus

Wright EM *et al*. (2011) Biology of human sodium glucose transporters. *Physiol. Rev*. **91**: 733‐94 https://www.ncbi.nlm.nih.gov/pubmed/21527736?dopt=AbstractPlus

## http://www.guidetopharmacology.org/GRAC/FamilyDisplayForward?familyId=337

### Overview

The SLC51 organic solute transporter family of transporters is a pair of heterodimeric proteins which regulate bile salt movements in the small intestine, bile duct, and liver, as part of the enterohepatic circulation [http://www.ncbi.nlm.nih.gov/pubmed/16317684?dopt=AbstractPlus, http://www.ncbi.nlm.nih.gov/pubmed/15563450?dopt=AbstractPlus]. OSTα/OSTβ is also expressed in steroidogenic cells of the brain and adrenal gland, where it may contribute to steroid movement [http://www.ncbi.nlm.nih.gov/pubmed/20649839?dopt=AbstractPlus]. Bile acid transport is suggested to be facilitative and independent of sodium, potassium, chloride ions or protons [http://www.ncbi.nlm.nih.gov/pubmed/16317684?dopt=AbstractPlus, http://www.ncbi.nlm.nih.gov/pubmed/15563450?dopt=AbstractPlus]. OSTα/OSTβ heterodimers have been shown to transport http://www.guidetopharmacology.org/GRAC/LigandDisplayForward?ligandId=4546, http://www.guidetopharmacology.org/GRAC/LigandDisplayForward?ligandId=5577, http://www.guidetopharmacology.org/GRAC/LigandDisplayForward?ligandId=4748, http://www.guidetopharmacology.org/GRAC/LigandDisplayForward?ligandId=6504 and http://www.guidetopharmacology.org/GRAC/LigandDisplayForward?ligandId=5577 [http://www.ncbi.nlm.nih.gov/pubmed/16317684?dopt=AbstractPlus, http://www.ncbi.nlm.nih.gov/pubmed/15563450?dopt=AbstractPlus, http://www.ncbi.nlm.nih.gov/pubmed/20649839?dopt=AbstractPlus]. OSTα/OSTβ‐mediated transport of bile salts is inhibited by http://www.guidetopharmacology.org/GRAC/LigandDisplayForward?ligandId=9184 [http://www.ncbi.nlm.nih.gov/pubmed/29675448?dopt=AbstractPlus]. OSTα is suggested to be a seven TM protein, while OSTβ is a single TM ‘ancillary’ protein, both of which are thought to have intracellular C‐termini [http://www.ncbi.nlm.nih.gov/pubmed/17650074?dopt=AbstractPlus]. Both proteins function in solute transport and bimolecular fluorescence complementation studies suggest the possibility of OSTα homooligomers, as well as OSTα/OSTβ hetero‐oligomers [http://www.ncbi.nlm.nih.gov/pubmed/22535958?dopt=AbstractPlus, http://www.ncbi.nlm.nih.gov/pubmed/17650074?dopt=AbstractPlus]. An inherited mutation in OSTβ is associated with congenital diarrhea in children [http://www.ncbi.nlm.nih.gov/pubmed/28898457?dopt=AbstractPlus].

**Table:** 

Nomenclature	http://www.guidetopharmacology.org/GRAC/ObjectDisplayForward?objectId=1915	http://www.guidetopharmacology.org/GRAC/ObjectDisplayForward?objectId=1916
Systematic nomenclature	SLC51A1	SLC51B
Common abbreviation	OSTα	OSTβ
HGNC, UniProt	https://www.genenames.org/data/gene‐symbol‐report/#!/hgnc_id/HGNC:29955, http://www.uniprot.org/uniprot/Q86UW1	https://www.genenames.org/data/gene‐symbol‐report/#!/hgnc_id/HGNC:29956, http://www.uniprot.org/uniprot/Q86UW2

### Further reading on SLC51 family of steroid‐derived molecule transporters

Ballatori N. (2011) Pleiotropic functions of the organic solute transporter Ostα‐Ostβ. *Dig Dis* **29**: 13‐7 https://www.ncbi.nlm.nih.gov/pubmed/21691099?dopt=AbstractPlus

Ballatori N *et al*. (2013) The heteromeric organic solute transporter, OSTα‐OSTβ/SLC51: a transporter for steroid‐derived molecules. *Mol. Aspects Med*. **34**: 683‐92 https://www.ncbi.nlm.nih.gov/pubmed/23506901?dopt=AbstractPlus

Dawson PA. (2011) Role of the intestinal bile acid transporters in bile acid and drug disposition. *Handb Exp Pharmacol* 169‐203 https://www.ncbi.nlm.nih.gov/pubmed/21103970?dopt=AbstractPlus

## http://www.guidetopharmacology.org/GRAC/FamilyDisplayForward?familyId=786

### Overview

http://www.guidetopharmacology.org/GRAC/LigandDisplayForward?ligandId=6578, also known as vitamin B2, is a precursor of the enzyme cofactors http://www.guidetopharmacology.org/GRAC/LigandDisplayForward?ligandId=5185 (FMN) and http://www.guidetopharmacology.org/GRAC/LigandDisplayForward?ligandId=5184 (FAD). Riboflavin transporters are predicted to possess 10 or 11 TM segments.

**Table:** 

Nomenclature	http://www.guidetopharmacology.org/GRAC/ObjectDisplayForward?objectId=2571	http://www.guidetopharmacology.org/GRAC/ObjectDisplayForward?objectId=2572	http://www.guidetopharmacology.org/GRAC/ObjectDisplayForward?objectId=2573
Systematic nomenclature	SLC52A1	SLC52A2	SLC52A3
Common abbreviation	RFVT1	RFVT2	RFVT3
HGNC, UniProt	https://www.genenames.org/data/gene‐symbol‐report/#!/hgnc_id/HGNC:30225, http://www.uniprot.org/uniprot/Q9NWF4	https://www.genenames.org/data/gene‐symbol‐report/#!/hgnc_id/HGNC:30224, http://www.uniprot.org/uniprot/Q9HAB3	https://www.genenames.org/data/gene‐symbol‐report/#!/hgnc_id/HGNC:16187, http://www.uniprot.org/uniprot/Q9NQ40
Endogenous substrates	http://www.guidetopharmacology.org/GRAC/LigandDisplayForward?ligandId=6578 (*K*_m_ 1.3×10^‐3^M) [http://www.ncbi.nlm.nih.gov/pubmed/20463145?dopt=AbstractPlus]	http://www.guidetopharmacology.org/GRAC/LigandDisplayForward?ligandId=6578 (*K*_m_ 9.8×10^‐4^M) [http://www.ncbi.nlm.nih.gov/pubmed/20463145?dopt=AbstractPlus]	http://www.guidetopharmacology.org/GRAC/LigandDisplayForward?ligandId=6578 (*K*_m_ 3.3×10^‐4^M) [http://www.ncbi.nlm.nih.gov/pubmed/20463145?dopt=AbstractPlus]
Stoichiometry	Unknown	Unknown	H^+^‐dependent

### Comments

Although expressed elsewhere, RFVT3 is found on the luminal surface of intestinal epithelium and is thought to mediate uptake of dietary riboflavin, while RFVT1 and RFVT2 are thought to allow movement from the epithelium into the blood.

### Further reading on SLC52 family of riboflavin transporters

Yonezawa A *et al*. (2013) Novel riboflavin transporter family RFVT/SLC52: identification, nomenclature, functional characterization and genetic diseases of RFVT/SLC52. *Mol. Aspects Med*. **34**: 693‐701 [https://www.ncbi.nlm.nih.gov/pubmed/23506902?dopt=AbstractPlus]

## http://www.guidetopharmacology.org/GRAC/FamilyDisplayForward?familyId=1005

**Table:** 

Nomenclature	http://www.guidetopharmacology.org/GRAC/ObjectDisplayForward?objectId=3021
Systematic nomenclature	SLC53A1
HGNC, UniProt	https://www.genenames.org/data/gene‐symbol‐report/#!/hgnc_id/HGNC:12827, http://www.uniprot.org/uniprot/Q9UBH6
Substrates	Phosphate [http://www.ncbi.nlm.nih.gov/pubmed/23791524?dopt=AbstractPlus]
Comments	XPR1/SLC53A1 is a phosphate carrier which appears to play a role in bone and tooth mineralization. It is ubiquitously expressed [http://www.ncbi.nlm.nih.gov/pubmed/9990033?dopt=AbstractPlus, http://www.ncbi.nlm.nih.gov/pubmed/9927670?dopt=AbstractPlus]. The pathological consequences of defective SLC53A1 expression in the brain [http://www.ncbi.nlm.nih.gov/pubmed/25938945?dopt=AbstractPlus] and kidney [http://www.ncbi.nlm.nih.gov/pubmed/27799484?dopt=AbstractPlus] have been reported.

## http://www.guidetopharmacology.org/GRAC/FamilyDisplayForward?familyId=1006

**Table:** 

Nomenclature	http://www.guidetopharmacology.org/GRAC/ObjectDisplayForward?objectId=3022	http://www.guidetopharmacology.org/GRAC/ObjectDisplayForward?objectId=3023	http://www.guidetopharmacology.org/GRAC/ObjectDisplayForward?objectId=3024
Systematic nomenclature	SLC54A1	SLC54A2	SLC54A3
HGNC, UniProt	https://www.genenames.org/data/gene‐symbol‐report/#!/hgnc_id/HGNC:21606, http://www.uniprot.org/uniprot/Q9Y5U8	https://www.genenames.org/data/gene‐symbol‐report/#!/hgnc_id/HGNC:24515, http://www.uniprot.org/uniprot/O95563	https://www.genenames.org/data/gene‐symbol‐report/#!/hgnc_id/HGNC:44205, http://www.uniprot.org/uniprot/P0DKB6
Substrates	Pyruvate [http://www.ncbi.nlm.nih.gov/pubmed/22628558?dopt=AbstractPlus, http://www.ncbi.nlm.nih.gov/pubmed/22628554?dopt=AbstractPlus]	Pyruvate [http://www.ncbi.nlm.nih.gov/pubmed/22628558?dopt=AbstractPlus]	Pyruvate [http://www.ncbi.nlm.nih.gov/pubmed/27317664?dopt=AbstractPlus]
Comments	SLC54A1 is ubiquitously expressed [http://www.ncbi.nlm.nih.gov/pubmed/27317664?dopt=AbstractPlus].	SLC54A2 is ubiquitously expressed [http://www.ncbi.nlm.nih.gov/pubmed/27317664?dopt=AbstractPlus].	SLC54A3 is expressed in testis, postmeiotic spermatids and sperm cells [http://www.ncbi.nlm.nih.gov/pubmed/27317664?dopt=AbstractPlus].

### Comments

SLC54 family transporters appear to function as mechanisms for accumulating pyruvate into mitochondria to link glycolysis with oxidative phosphorylation.

## http://www.guidetopharmacology.org/GRAC/FamilyDisplayForward?familyId=1007

**Table:** 

Nomenclature	http://www.guidetopharmacology.org/GRAC/ObjectDisplayForward?objectId=3025	http://www.guidetopharmacology.org/GRAC/ObjectDisplayForward?objectId=3026	http://www.guidetopharmacology.org/GRAC/ObjectDisplayForward?objectId=3027
Systematic nomenclature	SLC55A1	SLC55A2	SLC55A3
HGNC, UniProt	https://www.genenames.org/data/gene‐symbol‐report/#!/hgnc_id/HGNC:6556, http://www.uniprot.org/uniprot/O95202	https://www.genenames.org/data/gene‐symbol‐report/#!/hgnc_id/HGNC:14648, http://www.uniprot.org/uniprot/Q2VYF4	https://www.genenames.org/data/gene‐symbol‐report/#!/hgnc_id/HGNC:24241, http://www.uniprot.org/uniprot/Q6P1Q0
Transport type	Exchanger / Ca^2+^:H^+^ [http://www.ncbi.nlm.nih.gov/pubmed/19797662?dopt=AbstractPlus, http://www.ncbi.nlm.nih.gov/pubmed/27669901?dopt=AbstractPlus] Exchanger / K^+^:H^+^ [http://www.ncbi.nlm.nih.gov/pubmed/17925330?dopt=AbstractPlus, http://www.ncbi.nlm.nih.gov/pubmed/15138253?dopt=AbstractPlus]	–	–
Substrates	Ca^2+^, K^+^, H^+^ [http://www.ncbi.nlm.nih.gov/pubmed/17925330?dopt=AbstractPlus, http://www.ncbi.nlm.nih.gov/pubmed/15138253?dopt=AbstractPlus, http://www.ncbi.nlm.nih.gov/pubmed/17541427?dopt=AbstractPlus, http://www.ncbi.nlm.nih.gov/pubmed/20197279?dopt=AbstractPlus]	–	–
Comments	SLC55A1 is ubiquitously expressed [http://www.ncbi.nlm.nih.gov/pubmed/10486213?dopt=AbstractPlus]. Arguments against SLC55A1&apos;s role as a Ca^2+^ transporter are outlined by Zotova *et al*. (2010) [http://www.ncbi.nlm.nih.gov/pubmed/20197279?dopt=AbstractPlus].	–	–

### Comments

The family of SLC55 mitochondrial transporters appear to regulate ion fluxes and to maintain tubular networks.

## http://www.guidetopharmacology.org/GRAC/FamilyDisplayForward?familyId=1008

**Table:** 

Nomenclature	http://www.guidetopharmacology.org/GRAC/ObjectDisplayForward?objectId=3028	http://www.guidetopharmacology.org/GRAC/ObjectDisplayForward?objectId=3029	http://www.guidetopharmacology.org/GRAC/ObjectDisplayForward?objectId=3030	http://www.guidetopharmacology.org/GRAC/ObjectDisplayForward?objectId=3031	http://www.guidetopharmacology.org/GRAC/ObjectDisplayForward?objectId=3032
Systematic nomenclature	SLC56A1	SLC56A2	SLC56A3	SLC56A4	SLC56A5
HGNC, UniProt	https://www.genenames.org/data/gene‐symbol‐report/#!/hgnc_id/HGNC:16085, http://www.uniprot.org/uniprot/Q9H9B4	https://www.genenames.org/data/gene‐symbol‐report/#!/hgnc_id/HGNC:16086, http://www.uniprot.org/uniprot/Q96NB2	https://www.genenames.org/data/gene‐symbol‐report/#!/hgnc_id/HGNC:16087, http://www.uniprot.org/uniprot/Q9BWM7	–	https://www.genenames.org/data/gene‐symbol‐report/#!/hgnc_id/HGNC:16073, http://www.uniprot.org/uniprot/Q8TD22
Comments	Sideroflexin 1 (SFXN1/SLC56A1) was probably falsely identified as a tricarboxylate carrier in the 1993 article by Azzi *et al*. [http://www.ncbi.nlm.nih.gov/pubmed/8132491?dopt=AbstractPlus], as discussed several years later in [http://www.ncbi.nlm.nih.gov/pubmed/11274051?dopt=AbstractPlus]. SFXN1 likely transports pyridoxin or another heme precursor or the 5′‐aminolevulinate synthase 2 (https://www.genenames.org/data/gene‐symbol‐report/#!/hgnc_id/HGNC:397; https://www.uniprot.org/uniprot/P22557) cofactor [http://www.ncbi.nlm.nih.gov/pubmed/11274051?dopt=AbstractPlus, http://www.ncbi.nlm.nih.gov/pubmed/12670026?dopt=AbstractPlus]. SFXN1 has recently been suggested to be a mitochondrial serine transporter [http://www.ncbi.nlm.nih.gov/pubmed/30442778?dopt=AbstractPlus]. It is mainly expressed in adult kidney and liver (mouse) [http://www.ncbi.nlm.nih.gov/pubmed/11274051?dopt=AbstractPlus].	In mice sideroflexin 2 expression is mainly detected in adult kidney and liver [http://www.ncbi.nlm.nih.gov/pubmed/11274051?dopt=AbstractPlus]. In human tissues it is detected at highest levels in kidney, liver and pancreas [http://www.ncbi.nlm.nih.gov/pubmed/12670026?dopt=AbstractPlus].	Sideroflexin 3 is ubiquitously expressed in mouse tissues [http://www.ncbi.nlm.nih.gov/pubmed/11274051?dopt=AbstractPlus].	Sideroflexin 4 is expressed in mouse kidney, brain and heart [http://www.ncbi.nlm.nih.gov/pubmed/11274051?dopt=AbstractPlus]. The SFXN4a isoform is most highly expressed in human kidney and pancreas, and the SFXN4b isoform is barely detectable in brain [http://www.ncbi.nlm.nih.gov/pubmed/14756423?dopt=AbstractPlus].	Sideroflexin 5 is expressed in mouse brain and liver [http://www.ncbi.nlm.nih.gov/pubmed/11274051?dopt=AbstractPlus].

### Comments

These are a family of incompletely‐characterised mitochondrial transporters.

## http://www.guidetopharmacology.org/GRAC/FamilyDisplayForward?familyId=1009

**Table:** 

Nomenclature	http://www.guidetopharmacology.org/GRAC/ObjectDisplayForward?objectId=3033	http://www.guidetopharmacology.org/GRAC/ObjectDisplayForward?objectId=3034	http://www.guidetopharmacology.org/GRAC/ObjectDisplayForward?objectId=3035	http://www.guidetopharmacology.org/GRAC/ObjectDisplayForward?objectId=3037
Systematic nomenclature	SLC57A1	SLC57A2	SLC57A3	SLC57A5
HGNC, UniProt	https://www.genenames.org/data/gene‐symbol‐report/#!/hgnc_id/HGNC:17043, http://www.uniprot.org/uniprot/Q7RTP0	https://www.genenames.org/data/gene‐symbol‐report/#!/hgnc_id/HGNC:17044, http://www.uniprot.org/uniprot/Q8N8Q9	https://www.genenames.org/data/gene‐symbol‐report/#!/hgnc_id/HGNC:27194, http://www.uniprot.org/uniprot/Q6NVV3	https://www.genenames.org/data/gene‐symbol‐report/#!/hgnc_id/HGNC:25233, http://www.uniprot.org/uniprot/Q6P499
Substrates	Sr^2+^, Fe^2+^ and Co^2+^ to a lesser extent [http://www.ncbi.nlm.nih.gov/pubmed/18667602?dopt=AbstractPlus], Mg^2+^ [http://www.ncbi.nlm.nih.gov/pubmed/17166836?dopt=AbstractPlus]	Mg^2+^ [http://www.ncbi.nlm.nih.gov/pubmed/18667602?dopt=AbstractPlus]	Mg^2+^, Sr^2+^, Ba^2+^, Fe^2+^, Cu^2+^ [http://www.ncbi.nlm.nih.gov/pubmed/18667602?dopt=AbstractPlus]	–
Comments	Human tissue expression: Constitutively expressed at low levels, with significant enrichment in the brain [http://www.ncbi.nlm.nih.gov/pubmed/14508710?dopt=AbstractPlus]. Mouse tissue expression: Widely expressed, including in the heart, kidney, liver, colon, less in the brain, and not in the small intestine [http://www.ncbi.nlm.nih.gov/pubmed/17166836?dopt=AbstractPlus].	–	–	–

## http://www.guidetopharmacology.org/GRAC/FamilyDisplayForward?familyId=1010

**Table:** 

Nomenclature	http://www.guidetopharmacology.org/GRAC/ObjectDisplayForward?objectId=3039	http://www.guidetopharmacology.org/GRAC/ObjectDisplayForward?objectId=3040
Systematic nomenclature	SLC58A1	SLC58A2
HGNC, UniProt	https://www.genenames.org/data/gene‐symbol‐report/#!/hgnc_id/HGNC:28880, http://www.uniprot.org/uniprot/Q9H0U3	https://www.genenames.org/data/gene‐symbol‐report/#!/hgnc_id/HGNC:30242, http://www.uniprot.org/uniprot/Q13454
Transport type	Channel‐like [http://www.ncbi.nlm.nih.gov/pubmed/19940067?dopt=AbstractPlus]	–
Substrates	Mg^2+^ [http://www.ncbi.nlm.nih.gov/pubmed/15804357?dopt=AbstractPlus]	Mg^2+^, Fe^2+^, Cu^2+^, Mn^2+^ [http://www.ncbi.nlm.nih.gov/pubmed/18667602?dopt=AbstractPlus, http://www.ncbi.nlm.nih.gov/pubmed/19940067?dopt=AbstractPlus]
Comments	Expressed in kidney, colon, heart and liver (the latter only at the mRNA level) [http://www.ncbi.nlm.nih.gov/pubmed/15804357?dopt=AbstractPlus]; universally expressed [http://www.ncbi.nlm.nih.gov/pubmed/19717468?dopt=AbstractPlus].	Expressed in placenta, pancreas, testis, ovary, heart, and prostate [http://www.ncbi.nlm.nih.gov/pubmed/8661104?dopt=AbstractPlus].

## http://www.guidetopharmacology.org/GRAC/FamilyDisplayForward?familyId=1011

**Table:** 

Nomenclature	http://www.guidetopharmacology.org/GRAC/ObjectDisplayForward?objectId=3041	http://www.guidetopharmacology.org/GRAC/ObjectDisplayForward?objectId=3042
Systematic nomenclature	SLC59A1	SLC59A2
HGNC, UniProt	https://www.genenames.org/data/gene‐symbol‐report/#!/hgnc_id/HGNC:25897, http://www.uniprot.org/uniprot/Q8NA29	https://www.genenames.org/data/gene‐symbol‐report/#!/hgnc_id/HGNC:37207, http://www.uniprot.org/uniprot/A6NFX1
Transport type	Co‐transporter: LPC:Na^+^, uptake	–
Substrates	LPC (lysophosphatidylcholine) form of DHA (docosahexaenoic acid) [http://www.ncbi.nlm.nih.gov/pubmed/24828044?dopt=AbstractPlus]	–
Comments	MFSD2B/SLC59A has been suggested to be a sphingosine 1‐phosphate transporter in erythropoietic cells [http://www.ncbi.nlm.nih.gov/pubmed/29563527?dopt=AbstractPlus]. It is expressed in brain, intestine, kidney, liver, lung, mammary gland, and prostate [http://www.ncbi.nlm.nih.gov/pubmed/18694395?dopt=AbstractPlus]; relatively low expression in BAT (brown adipose tissue), but upregulated during cold‐induced thermogenesis [http://www.ncbi.nlm.nih.gov/pubmed/18694395?dopt=AbstractPlus]. Subcellular locations: plasma membrane [http://www.ncbi.nlm.nih.gov/pubmed/26747400?dopt=AbstractPlus] and ER [http://www.ncbi.nlm.nih.gov/pubmed/18694395?dopt=AbstractPlus].	Expressed in the spleen, lung, testis and subcellularly in the ER [http://www.ncbi.nlm.nih.gov/pubmed/18694395?dopt=AbstractPlus].

## http://www.guidetopharmacology.org/GRAC/FamilyDisplayForward?familyId=1012

**Table:** 

Nomenclature	http://www.guidetopharmacology.org/GRAC/ObjectDisplayForward?objectId=3043	http://www.guidetopharmacology.org/GRAC/ObjectDisplayForward?objectId=3044
Systematic nomenclature	SLC60A1	SLC60A2
HGNC, UniProt	https://www.genenames.org/data/gene‐symbol‐report/#!/hgnc_id/HGNC:25433, http://www.uniprot.org/uniprot/Q8N468	https://www.genenames.org/data/gene‐symbol‐report/#!/hgnc_id/HGNC:21053, http://www.uniprot.org/uniprot/Q5TF39
Transport type	–	Co‐transporter / Na^+^ (1:1) uptake (Rat) [http://www.ncbi.nlm.nih.gov/pubmed/12590146?dopt=AbstractPlus]
Substrates	‐	α‐Me‐glucose, D‐glucose [http://www.ncbi.nlm.nih.gov/pubmed/12590146?dopt=AbstractPlus]
Inhibitors	–	http://www.guidetopharmacology.org/GRAC/LigandDisplayForward?ligandId=4285 [http://www.ncbi.nlm.nih.gov/pubmed/12590146?dopt=AbstractPlus] – Rat, http://www.guidetopharmacology.org/GRAC/LigandDisplayForward?ligandId=4757 [http://www.ncbi.nlm.nih.gov/pubmed/12590146?dopt=AbstractPlus] – Rat, http://www.guidetopharmacology.org/GRAC/LigandDisplayForward?ligandId=4539 [http://www.ncbi.nlm.nih.gov/pubmed/26423860?dopt=AbstractPlus] – Rat
Comments	–	Expressed in rat kidney (cortex and medulla), brain, liver and lung [http://www.ncbi.nlm.nih.gov/pubmed/12590146?dopt=AbstractPlus].

## http://www.guidetopharmacology.org/GRAC/FamilyDisplayForward?familyId=1013

**Table:** 

Nomenclature	http://www.guidetopharmacology.org/GRAC/ObjectDisplayForward?objectId=3045
Systematic nomenclature	SLC61A1
HGNC, UniProt	https://www.genenames.org/data/gene‐symbol‐report/#!/hgnc_id/HGNC:28156, http://www.uniprot.org/uniprot/Q6N075
Substrates	molybdate [http://www.ncbi.nlm.nih.gov/pubmed/21464289?dopt=AbstractPlus]
Comments	MFSD5/SLC61 is a putative 12TM cell‐surface protein which appears to allow the accumulation of molybdate, and where the neural expression appears to respond to changes in the diet. It is expressed in cervix, stomach, nerve and skin [http://www.ncbi.nlm.nih.gov/pubmed/21464289?dopt=AbstractPlus]; ubiquitous but higher in skeletal muscle, olfactory bulb [http://www.ncbi.nlm.nih.gov/pubmed/18948099?dopt=AbstractPlus]; blood, cortex, hypothalamus, cerebellum and spinal cord (mouse) [http://www.ncbi.nlm.nih.gov/pubmed/27272503?dopt=AbstractPlus].

## http://www.guidetopharmacology.org/GRAC/FamilyDisplayForward?familyId=1014

**Table:** 

Nomenclature	http://www.guidetopharmacology.org/GRAC/ObjectDisplayForward?objectId=3046
Systematic nomenclature	SLC62A1
HGNC, UniProt	https://www.genenames.org/data/gene‐symbol‐report/#!/hgnc_id/HGNC:15492, Qhttp://www.uniprot.org/uniprot/Q9HCJ1
Substrates	Pyrophosphate [http://www.ncbi.nlm.nih.gov/pubmed/10894769?dopt=AbstractPlus]
Comments	ANKH/SLC62 is a putative 8TM membrane protein, also known as progressive ankylosis protein homolog. Mutations in this protein are associated with bone and joint abnormalities. It is expressed in kidney and bone [http://www.ncbi.nlm.nih.gov/pubmed/19910700?dopt=AbstractPlus].

## http://www.guidetopharmacology.org/GRAC/FamilyDisplayForward?familyId=1015

### Overview

The SLC63 family of transporters has roles inside the cell (SLC63A1/SPNS1) or on the cell surface (SLC63A2/SPNS2) in sphingolipid transport.

**Table:** 

Nomenclature	http://www.guidetopharmacology.org/GRAC/ObjectDisplayForward?objectId=3047
Systematic nomenclature	SLC63A1
HGNC, UniProt	https://www.genenames.org/data/gene‐symbol‐report/#!/hgnc_id/HGNC:30621, http://www.uniprot.org/uniprot/Q9H2V7
Comments	Expressed in mitochondria [http://www.ncbi.nlm.nih.gov/pubmed/12815463?dopt=AbstractPlus].

## http://www.guidetopharmacology.org/GRAC/FamilyDisplayForward?familyId=1016

**Table:** 

Nomenclature	http://www.guidetopharmacology.org/GRAC/ObjectDisplayForward?objectId=3050
Systematic nomenclature	SLC64A1
HGNC, UniProt	https://www.genenames.org/data/gene‐symbol‐report/#!/hgnc_id/HGNC:30760, http://www.uniprot.org/uniprot/Q9HC07
Transport type	Exchanger/ Ca^2+^:H^+^
Substrates	Mn^2+^ [http://www.ncbi.nlm.nih.gov/pubmed/28270545?dopt=AbstractPlus, http://www.ncbi.nlm.nih.gov/pubmed/27008884?dopt=AbstractPlus], Ca^2+^, H^+^ [http://www.ncbi.nlm.nih.gov/pubmed/23569283?dopt=AbstractPlus]
Comments	TMEM165/SLC64 is a putative 6TM intracellular membrane protein. Mutations in the protein are associated with congenital disorder of glycosylation. It has been suggested to be essential for milk production in the mammary gland [http://www.ncbi.nlm.nih.gov/pubmed/30622138?dopt=AbstractPlus]. TMEM165 deficiency (*via* siRNA knockdown) causes Golgi glycosylation defects in transfected HEK cells [http://www.ncbi.nlm.nih.gov/pubmed/22683087?dopt=AbstractPlus].

## http://www.guidetopharmacology.org/GRAC/FamilyDisplayForward?familyId=1017

### Overview

The SLC65 family of intracellular cholesterol transporters are 13TM membrane proteins. NPC1/SLC65A1 is an intracellular cholesterol transporter, which together with NPC2 (Uniprot ID https://www.uniprot.org/uniprot/P61916), allows the accumulation into the cytosol of cholesterol acquired from low density lipoproteins.

**Table:** 

Nomenclature	http://www.guidetopharmacology.org/GRAC/ObjectDisplayForward?objectId=3051	http://www.guidetopharmacology.org/GRAC/ObjectDisplayForward?objectId=2629
Systematic nomenclature	SLC65A1	SLC65A2
HGNC, UniProt	https://www.genenames.org/data/gene‐symbol‐report/#!/hgnc_id/HGNC:7897, http://www.uniprot.org/uniprot/O15118	https://www.genenames.org/data/gene‐symbol‐report/#!/hgnc_id/HGNC:7898, http://www.uniprot.org/uniprot/Q9UHC9
Substrates	Cholesterol [http://www.ncbi.nlm.nih.gov/pubmed/17989073?dopt=AbstractPlus, http://www.ncbi.nlm.nih.gov/pubmed/17989072?dopt=AbstractPlus, http://www.ncbi.nlm.nih.gov/pubmed/27410046?dopt=AbstractPlus]	Cholesterol [http://www.ncbi.nlm.nih.gov/pubmed/14976318?dopt=AbstractPlus]
Selective antagonists	–	http://www.guidetopharmacology.org/GRAC/LigandDisplayForward?ligandId=6816 (Inhibition) (p*K*_d_ 6.7) [http://www.ncbi.nlm.nih.gov/pubmed/15928087?dopt=AbstractPlus]
Comments	Expression is ubiquitous [http://www.ncbi.nlm.nih.gov/pubmed/14976318?dopt=AbstractPlus], with highest levels detected in liver, lung, and pancreas [http://www.ncbi.nlm.nih.gov/pubmed/10783261?dopt=AbstractPlus]. NPC1 plays a critical role in the regulation of intracellular cholesterol trafficking [http://www.ncbi.nlm.nih.gov/pubmed/9211849?dopt=AbstractPlus]. Mutations in the NPC1 gene have been identified in patients with the lipid storage disorder Niemann‐Pick disease type C1 [http://www.ncbi.nlm.nih.gov/pubmed/12554680?dopt=AbstractPlus, http://www.ncbi.nlm.nih.gov/pubmed/9211849?dopt=AbstractPlus, http://www.ncbi.nlm.nih.gov/pubmed/10521290?dopt=AbstractPlus, http://www.ncbi.nlm.nih.gov/pubmed/10480349?dopt=AbstractPlus].	Expressed in small intestine, gallbladder, liver, testis and stomach [http://www.ncbi.nlm.nih.gov/pubmed/14976318?dopt=AbstractPlus].

## http://www.guidetopharmacology.org/GRAC/FamilyDisplayForward?familyId=238

### Overview

The SLCO superfamily is comprised of the organic anion transporting polypeptides (OATPs). The 11 human OATPs are divided into 6 families and ten subfamilies based on amino acid identity. These proteins are located on the plasma membrane of cells throughout the body. They have 12 TM domains and intracellular termini, with multiple putative glycosylation sites. OATPs mediate the sodium‐independent uptake of a wide range of amphiphilic substrates, including many drugs and toxins. Due to the multispecificity of these proteins, this guide lists classes of substrates and inhibitors for each family member. More comprehensive lists of substrates, inhibitors, and their relative affinities may be found in the review articles listed below.

**Table:** 

Nomenclature	http://www.guidetopharmacology.org/GRAC/ObjectDisplayForward?objectId=1219	http://www.guidetopharmacology.org/GRAC/ObjectDisplayForward?objectId=1220	http://www.guidetopharmacology.org/GRAC/ObjectDisplayForward?objectId=1221	http://www.guidetopharmacology.org/GRAC/ObjectDisplayForward?objectId=1222
Systematic nomenclature	SLCO1A2	SLCO1B1	SLCO1B3	SLCO1C1
HGNC, UniProt	https://www.genenames.org/data/gene‐symbol‐report/#!/hgnc_id/HGNC:10956, http://www.uniprot.org/uniprot/P46721	https://www.genenames.org/data/gene‐symbol‐report/#!/hgnc_id/HGNC:10959, http://www.uniprot.org/uniprot/Q9Y6L6	https://www.genenames.org/data/gene‐symbol‐report/#!/hgnc_id/HGNC:10961, http://www.uniprot.org/uniprot/Q9NPD5	https://www.genenames.org/data/gene‐symbol‐report/#!/hgnc_id/HGNC:13819, http://www.uniprot.org/uniprot/Q9NYB5
Substrates	fluoroquinolones, beta blockers, http://www.guidetopharmacology.org/GRAC/LigandDisplayForward?ligandId=1615, http://www.guidetopharmacology.org/GRAC/LigandDisplayForward?ligandId=2954, http://www.guidetopharmacology.org/GRAC/LigandDisplayForward?ligandId=4819, http://www.guidetopharmacology.org/GRAC/LigandDisplayForward?ligandId=4506, anticancer drugs, antibiotics, HIV protease inhibitors, http://www.guidetopharmacology.org/GRAC/LigandDisplayForward?ligandId=4664, http://www.guidetopharmacology.org/GRAC/LigandDisplayForward?ligandId=4826, http://www.guidetopharmacology.org/GRAC/LigandDisplayForward?ligandId=4735 [http://www.ncbi.nlm.nih.gov/pubmed/15737679?dopt=AbstractPlus]	statins, opioids, β‐lactam antibiotics, bile acid derivatives and conjugates, http://www.guidetopharmacology.org/GRAC/LigandDisplayForward?ligandId=4506, anticancer drugs, HIV protease inhibitors, http://www.guidetopharmacology.org/GRAC/LigandDisplayForward?ligandId=4819, antifungals, ACE inhibitors, http://www.guidetopharmacology.org/GRAC/LigandDisplayForward?ligandId=2765, endothelin receptor antagonists, sartans	http://www.guidetopharmacology.org/GRAC/LigandDisplayForward?ligandId=2765, opioids, sartans, statins, http://www.guidetopharmacology.org/GRAC/LigandDisplayForward?ligandId=4726, anticancer drugs, http://www.guidetopharmacology.org/GRAC/LigandDisplayForward?ligandId=4506, bile acid derivatives and conjugates, β‐lactam antibiotics, http://www.guidetopharmacology.org/GRAC/LigandDisplayForward?ligandId=4826, http://www.guidetopharmacology.org/GRAC/LigandDisplayForward?ligandId=4632, http://www.guidetopharmacology.org/GRAC/LigandDisplayForward?ligandId=4813, http://www.guidetopharmacology.org/GRAC/LigandDisplayForward?ligandId=4819, http://www.guidetopharmacology.org/GRAC/LigandDisplayForward?ligandId=1456, http://www.guidetopharmacology.org/GRAC/LigandDisplayForward?ligandId=4736	statins, http://www.guidetopharmacology.org/GRAC/LigandDisplayForward?ligandId=4506
Endogenous substrates	bile acids, thyroid hormones, steroid conjugates, http://www.guidetopharmacology.org/GRAC/LigandDisplayForward?ligandId=4577, http://www.guidetopharmacology.org/GRAC/LigandDisplayForward?ligandId=1883	leukotrienes, steroid conjugates, thyroid hormones, bile acids, http://www.guidetopharmacology.org/GRAC/LigandDisplayForward?ligandId=4577	steroid conjugates, thyroid hormones, bile acids, http://www.guidetopharmacology.org/GRAC/LigandDisplayForward?ligandId=864 (https://www.genenames.org/data/gene‐symbol‐report/#!/hgnc_id/HGNC:1569, http://www.uniprot.org/uniprot/P06307), http://www.guidetopharmacology.org/GRAC/LigandDisplayForward?ligandId=4577, http://www.guidetopharmacology.org/GRAC/LigandDisplayForward?ligandId=3354	thyroid hormones, steroid conjugates
Ligands	–	http://www.guidetopharmacology.org/GRAC/LigandDisplayForward?ligandId=2953 (Binding)	–	–
Inhibitors	http://www.guidetopharmacology.org/GRAC/LigandDisplayForward?ligandId=4570 (p*K*_i_ 5) [http://www.ncbi.nlm.nih.gov/pubmed/12085361?dopt=AbstractPlus], http://www.guidetopharmacology.org/GRAC/LigandDisplayForward?ligandId=2765 (p*K*_i_ 4.3) [http://www.ncbi.nlm.nih.gov/pubmed/12085361?dopt=AbstractPlus], http://www.guidetopharmacology.org/GRAC/LigandDisplayForward?ligandId=4738 [http://www.ncbi.nlm.nih.gov/pubmed/17301733?dopt=AbstractPlus]	http://www.guidetopharmacology.org/GRAC/LigandDisplayForward?ligandId=1024 (p*K*_i_ 7.3) [http://www.ncbi.nlm.nih.gov/pubmed/14530907?dopt=AbstractPlus, http://www.ncbi.nlm.nih.gov/pubmed/22541068?dopt=AbstractPlus], http://www.guidetopharmacology.org/GRAC/LigandDisplayForward?ligandId=4749 (pIC_50_ 7.2) [http://www.ncbi.nlm.nih.gov/pubmed/20448812?dopt=AbstractPlus], http://www.guidetopharmacology.org/GRAC/LigandDisplayForward?ligandId=2765 (p*K*_i_ 6) [http://www.ncbi.nlm.nih.gov/pubmed/22541068?dopt=AbstractPlus], http://www.guidetopharmacology.org/GRAC/LigandDisplayForward?ligandId=4570 (p*K*_i_ 5.7) [http://www.ncbi.nlm.nih.gov/pubmed/12085361?dopt=AbstractPlus], http://www.guidetopharmacology.org/GRAC/LigandDisplayForward?ligandId=3439 [http://www.ncbi.nlm.nih.gov/pubmed/17470528?dopt=AbstractPlus], http://www.guidetopharmacology.org/GRAC/LigandDisplayForward?ligandId=4688, http://www.guidetopharmacology.org/GRAC/LigandDisplayForward?ligandId=4844	http://www.guidetopharmacology.org/GRAC/LigandDisplayForward?ligandId=1024 (pIC_50_ 6.1) [http://www.ncbi.nlm.nih.gov/pubmed/22541068?dopt=AbstractPlus, http://www.ncbi.nlm.nih.gov/pubmed/17496208?dopt=AbstractPlus], http://www.guidetopharmacology.org/GRAC/LigandDisplayForward?ligandId=4743 (pIC_50_ 6.1) [http://www.ncbi.nlm.nih.gov/pubmed/17496208?dopt=AbstractPlus], http://www.guidetopharmacology.org/GRAC/LigandDisplayForward?ligandId=2765 (pIC_50_ 5.8) [http://www.ncbi.nlm.nih.gov/pubmed/22541068?dopt=AbstractPlus, http://www.ncbi.nlm.nih.gov/pubmed/17496208?dopt=AbstractPlus], http://www.guidetopharmacology.org/GRAC/LigandDisplayForward?ligandId=3439, http://www.guidetopharmacology.org/GRAC/LigandDisplayForward?ligandId=4688, http://www.guidetopharmacology.org/GRAC/LigandDisplayForward?ligandId=4570	http://www.guidetopharmacology.org/GRAC/LigandDisplayForward?ligandId=1608, http://www.guidetopharmacology.org/GRAC/LigandDisplayForward?ligandId=4357, http://www.guidetopharmacology.org/GRAC/LigandDisplayForward?ligandId=4547
Labelled ligands	http://www.guidetopharmacology.org/GRAC/LigandDisplayForward?ligandId=4505, http://www.guidetopharmacology.org/GRAC/LigandDisplayForward?ligandId=3819, http://www.guidetopharmacology.org/GRAC/LigandDisplayForward?ligandId=4748	http://www.guidetopharmacology.org/GRAC/LigandDisplayForward?ligandId=4687, http://www.guidetopharmacology.org/GRAC/LigandDisplayForward?ligandId=4748	http://www.guidetopharmacology.org/GRAC/LigandDisplayForward?ligandId=4505, http://www.guidetopharmacology.org/GRAC/LigandDisplayForward?ligandId=4765, http://www.guidetopharmacology.org/GRAC/LigandDisplayForward?ligandId=4687	http://www.guidetopharmacology.org/GRAC/LigandDisplayForward?ligandId=4627, http://www.guidetopharmacology.org/GRAC/LigandDisplayForward?ligandId=4505, http://www.guidetopharmacology.org/GRAC/LigandDisplayForward?ligandId=4748
Comments	Although rat and mouse OATP1A4 are considered the orthologs of human OATP1A2 we do not cross‐link to gene or protein databases for these since in reality there are five genes in rodents that arose through gene duplication in this family and it is not clear which one of these is the "true" ortholog.	Other inhibitors include, fibrates, flavonoids, glitazones and macrolide antibiotics. http://www.guidetopharmacology.org/GRAC/LigandDisplayForward?ligandId=4749 or the drug substrates http://www.guidetopharmacology.org/GRAC/LigandDisplayForward?ligandId=2949, http://www.guidetopharmacology.org/GRAC/LigandDisplayForward?ligandId=2953 and http://www.guidetopharmacology.org/GRAC/LigandDisplayForward?ligandId=2954 are used as a probe.	Other inhibitors include, HIV protease inhibitors, glitazones and macrolide antibiotics. CCK‐8 is used as an OATP1B3‐selective probe.	–

**Table:** 

Nomenclature	http://www.guidetopharmacology.org/GRAC/ObjectDisplayForward?objectId=1223	http://www.guidetopharmacology.org/GRAC/ObjectDisplayForward?objectId=1224	http://www.guidetopharmacology.org/GRAC/ObjectDisplayForward?objectId=1225	http://www.guidetopharmacology.org/GRAC/ObjectDisplayForward?objectId=1226	http://www.guidetopharmacology.org/GRAC/ObjectDisplayForward?objectId=1227
Systematic nomenclature	SLCO2A1	SLCO2B1	SLCO3A1	SLCO4A1	SLCO4C1
HGNC, UniProt	https://www.genenames.org/data/gene‐symbol‐report/#!/hgnc_id/HGNC:10955, http://www.uniprot.org/uniprot/Q92959	https://www.genenames.org/data/gene‐symbol‐report/#!/hgnc_id/HGNC:10962, http://www.uniprot.org/uniprot/O94956	https://www.genenames.org/data/gene‐symbol‐report/#!/hgnc_id/HGNC:10952, http://www.uniprot.org/uniprot/Q9UIG8	https://www.genenames.org/data/gene‐symbol‐report/#!/hgnc_id/HGNC:10953, http://www.uniprot.org/uniprot/Q96BD0	https://www.genenames.org/data/gene‐symbol‐report/#!/hgnc_id/HGNC:23612, http://www.uniprot.org/uniprot/Q6ZQN7
Substrates	synthetic prostaglandin derivatives	http://www.guidetopharmacology.org/GRAC/LigandDisplayForward?ligandId=2566, http://www.guidetopharmacology.org/GRAC/LigandDisplayForward?ligandId=4506, statins, http://www.guidetopharmacology.org/GRAC/LigandDisplayForward?ligandId=2414, http://www.guidetopharmacology.org/GRAC/LigandDisplayForward?ligandId=4812, http://www.guidetopharmacology.org/GRAC/LigandDisplayForward?ligandId=4819, http://www.guidetopharmacology.org/GRAC/LigandDisplayForward?ligandId=4664, http://www.guidetopharmacology.org/GRAC/LigandDisplayForward?ligandId=3494, http://www.guidetopharmacology.org/GRAC/LigandDisplayForward?ligandId=592	–	http://www.guidetopharmacology.org/GRAC/LigandDisplayForward?ligandId=4796	dipeptidyl peptidase‐4 inhibitors, anticancer drugs, cardiac glycosides
Endogenous substrates	prostaglandins, eicosanoids	http://www.guidetopharmacology.org/GRAC/LigandDisplayForward?ligandId=4749, http://www.guidetopharmacology.org/GRAC/LigandDisplayForward?ligandId=4528, http://www.guidetopharmacology.org/GRAC/LigandDisplayForward?ligandId=2635	http://www.guidetopharmacology.org/GRAC/LigandDisplayForward?ligandId=997, http://www.guidetopharmacology.org/GRAC/LigandDisplayForward?ligandId=2168 (https://www.genenames.org/data/gene‐symbol‐report/#!/hgnc_id/HGNC:894, http://www.uniprot.org/uniprot/P01185), thyroid hormones, prostaglandins	thyroid hormones, prostaglandins, bile acids, steroid conjugates	thyroid hormones, http://www.guidetopharmacology.org/GRAC/LigandDisplayForward?ligandId=2352, steroid conjugates
Inhibitors	http://www.guidetopharmacology.org/GRAC/LigandDisplayForward?ligandId=4530 (Inhibition of PGF_2α_ uptake in PGT‐expressing HeLa cells) (p*K*_i_ 5.4) [http://www.ncbi.nlm.nih.gov/pubmed/7754369?dopt=AbstractPlus] – Rat, http://www.guidetopharmacology.org/GRAC/LigandDisplayForward?ligandId=4506 (Inhibition of PGF_2α_ uptake in PGT‐expressing HeLa cells) (p*K*_i_ 5.2) [http://www.ncbi.nlm.nih.gov/pubmed/7754369?dopt=AbstractPlus] – Rat	http://www.guidetopharmacology.org/GRAC/LigandDisplayForward?ligandId=4920 (p*K*_i_ 6.3) [http://www.ncbi.nlm.nih.gov/pubmed/22541068?dopt=AbstractPlus], http://www.guidetopharmacology.org/GRAC/LigandDisplayForward?ligandId=6193 (p*K*_i_ 5.6) [http://www.ncbi.nlm.nih.gov/pubmed/22541068?dopt=AbstractPlus], http://www.guidetopharmacology.org/GRAC/LigandDisplayForward?ligandId=3439, http://www.guidetopharmacology.org/GRAC/LigandDisplayForward?ligandId=2414, http://www.guidetopharmacology.org/GRAC/LigandDisplayForward?ligandId=4570, http://www.guidetopharmacology.org/GRAC/LigandDisplayForward?ligandId=4743 [http://www.ncbi.nlm.nih.gov/pubmed/17496208?dopt=AbstractPlus]	–	–	–
Labelled ligands	http://www.guidetopharmacology.org/GRAC/LigandDisplayForward?ligandId=1916 (Binding) [http://www.ncbi.nlm.nih.gov/pubmed/9506966?dopt=AbstractPlus]	http://www.guidetopharmacology.org/GRAC/LigandDisplayForward?ligandId=4505, http://www.guidetopharmacology.org/GRAC/LigandDisplayForward?ligandId=4748	http://www.guidetopharmacology.org/GRAC/LigandDisplayForward?ligandId=1916, http://www.guidetopharmacology.org/GRAC/LigandDisplayForward?ligandId=4748	http://www.guidetopharmacology.org/GRAC/LigandDisplayForward?ligandId=4748	http://www.guidetopharmacology.org/GRAC/LigandDisplayForward?ligandId=4725
Comments	Other inhibitors include NSAIDs	Other inhibitors include glitazones and citrus juices	–	–	–

### Further reading on SLCO family of organic anion transporting polypeptides

Hagenbuch B *et al*. (2013) The SLCO (former SLC21) superfamily of transporters. *Mol. Aspects Med*. **34**: 396‐412 https://www.ncbi.nlm.nih.gov/pubmed/23506880?dopt=AbstractPlus

Hillgren KM *et al*. (2013) Emerging transporters of clinical importance: an update from the International Transporter Consortium. *Clin. Pharmacol. Ther*. **94**: 52‐63 https://www.ncbi.nlm.nih.gov/pubmed/23588305?dopt=AbstractPlus

International Transporter Consortium *et al*. (2010) Membrane transporters in drug development. *Nat Rev Drug Discov* **9**: 215‐36 https://www.ncbi.nlm.nih.gov/pubmed/20190787?dopt=AbstractPlus

Lee HH *et al*. (2017) Interindividual and interethnic variability in drug disposition: polymorphisms in organic anion transporting polypeptide 1B1 (OATP1B1; SLCO1B1). *Br J Clin Pharmacol* **83**: 1176‐1184 https://www.ncbi.nlm.nih.gov/pubmed/27936281?dopt=AbstractPlus

Roth M *et al*. (2012) OATPs, OATs and OCTs: the organic anion and cation transporters of the SLCO and SLC22A gene superfamilies. *Br. J. Pharmacol*. **165**: 1260‐87 https://www.ncbi.nlm.nih.gov/pubmed/22013971?dopt=AbstractPlus

Zamek‐Gliszczynski MJ *et al*. (2018) Transporters in Drug Development: 2018 ITC Recommendations for Transporters of Emerging Clinical Importance. *Clin. Pharmacol. Ther*. **104**: 890‐899 https://www.ncbi.nlm.nih.gov/pubmed/30091177?dopt=AbstractPlus

### Further reading on SLC superfamily of solute carriers

Bhutia YD *et al*. (2016) SLC transporters as a novel class of tumour suppressors: identity, function and molecular mechanisms. *Biochem. J*. **473**: 1113‐24 https://www.ncbi.nlm.nih.gov/pubmed/27118869?dopt=AbstractPlus

Colas C *et al*. (2016) SLC Transporters: Structure, Function, and Drug Discovery. *Medchemcomm* **7**: 1069‐1081 https://www.ncbi.nlm.nih.gov/pubmed/27672436?dopt=AbstractPlus

César‐Razquin A *et al*. (2015) A Call for Systematic Research on Solute Carriers. *Cell* **162**: 478‐87 https://www.ncbi.nlm.nih.gov/pubmed/26232220?dopt=AbstractPlus

Lin L *et al*. (2015) SLC transporters as therapeutic targets: emerging opportunities. *Nat Rev Drug Discov* **14**: 543‐60 https://www.ncbi.nlm.nih.gov/pubmed/26111766?dopt=AbstractPlus

Na^a^|cz KA. (2017) Solute Carriers in the Blood‐Brain Barier: Safety in Abundance. *Neurochem. Res*. **42**: 795‐809 https://www.ncbi.nlm.nih.gov/pubmed/27503090?dopt=AbstractPlus

Neul C *et al*. (2016) Impact of Membrane Drug Transporters on Resistance to Small‐Molecule Tyrosine Kinase Inhibitors. *Trends Pharmacol. Sci*. **37**: 904‐932 https://www.ncbi.nlm.nih.gov/pubmed/27659854?dopt=AbstractPlus

Nigam SK. (2015) What do drug transporters really do? *Nat Rev Drug Discov* **14**: 29‐44 https://www.ncbi.nlm.nih.gov/pubmed/25475361?dopt=AbstractPlus

Pedersen NB *et al*. (2016) Glycosylation of solute carriers: mechanisms and functional consequences. *Pflugers Arch*. **468**: 159‐76 https://www.ncbi.nlm.nih.gov/pubmed/26383868?dopt=AbstractPlus

Perland E *et al*. (2017) Classification Systems of Secondary Active Transporters. *Trends Pharmacol. Sci*. **38**: 305‐315 https://www.ncbi.nlm.nih.gov/pubmed/27939446?dopt=AbstractPlus

Rives ML *et al*. (2017) Potentiating SLC transporter activity: Emerging drug discovery opportunities. *Biochem. Pharmacol*. **135**: 1‐11 https://www.ncbi.nlm.nih.gov/pubmed/28214518?dopt=AbstractPlus

## References

[1] Abbot EL *et al* (2006) http://www.ncbi.nlm.nih.gov/pubmed/16331283?dopt=AbstractPlus

[2] Abramson J *et al* (2009) http://www.ncbi.nlm.nih.gov/pubmed/19631523?dopt=AbstractPlus

[3] Agu R *et al* (2011) http://www.ncbi.nlm.nih.gov/pubmed/21366347?dopt=AbstractPlus

[4] Aguirre P *et al* (2005) http://www.ncbi.nlm.nih.gov/pubmed/15667655?dopt=AbstractPlus

[5] Agulhon C *et al* (2003) http://www.ncbi.nlm.nih.gov/pubmed/12761825?dopt=AbstractPlus

[6] Akazawa T *et al* (2018) http://www.ncbi.nlm.nih.gov/pubmed/30135242?dopt=AbstractPlus

[7] Albers A *et al* (2001) http://www.ncbi.nlm.nih.gov/pubmed/11692272?dopt=AbstractPlus

[8] Albrecht C *et al* (2007) http://www.ncbi.nlm.nih.gov/pubmed/16586097?dopt=AbstractPlus

[9] Almqvist J *et al* (2004) http://www.ncbi.nlm.nih.gov/pubmed/15260472?dopt=AbstractPlus

[10] Altmann SW *et al* (2004) http://www.ncbi.nlm.nih.gov/pubmed/14976318?dopt=AbstractPlus

[11] Amara SG *et al* (1993) http://www.ncbi.nlm.nih.gov/pubmed/8103691?dopt=AbstractPlus

[12] Anand BS *et al* (2003) http://www.ncbi.nlm.nih.gov/pubmed/12538834?dopt=AbstractPlus

[13] Anderson CM *et al* (2008) http://www.ncbi.nlm.nih.gov/pubmed/18599538?dopt=AbstractPlus

[14] Anderson CM *et al* (2004) http://www.ncbi.nlm.nih.gov/pubmed/15521011?dopt=AbstractPlus

[15] Anderson CM *et al* (2009) http://www.ncbi.nlm.nih.gov/pubmed/19074966?dopt=AbstractPlus

[16] Anderson CM *et al* (2010) http://www.ncbi.nlm.nih.gov/pubmed/19789362?dopt=AbstractPlus

[17] Anderson CM *et al* (2005) http://www.ncbi.nlm.nih.gov/pubmed/15754324?dopt=AbstractPlus

[18] Andrini O *et al* (2008) http://www.ncbi.nlm.nih.gov/pubmed/18989094?dopt=AbstractPlus

[19] Angers M *et al* (2008) http://www.ncbi.nlm.nih.gov/pubmed/18694395?dopt=AbstractPlus

[20] Ansermet C *et al* (2017) http://www.ncbi.nlm.nih.gov/pubmed/27799484?dopt=AbstractPlus

[21] Aouameur R *et al* (2007) http://www.ncbi.nlm.nih.gov/pubmed/17932225?dopt=AbstractPlus

[22] Apparsundaram S *et al* (2000) http://www.ncbi.nlm.nih.gov/pubmed/11027560?dopt=AbstractPlus

[23] Apricò K *et al* (2004) http://www.ncbi.nlm.nih.gov/pubmed/14994336?dopt=AbstractPlus

[24] Apricò K *et al* (2007) http://www.ncbi.nlm.nih.gov/pubmed/17590480?dopt=AbstractPlus

[25] Apricó K *et al* (2001) http://www.ncbi.nlm.nih.gov/pubmed/11389172?dopt=AbstractPlus

[26] Armstrong D *et al* (2014) http://www.ncbi.nlm.nih.gov/pubmed/24414167?dopt=AbstractPlus

[27] Arriza JL *et al* (1993) http://www.ncbi.nlm.nih.gov/pubmed/8101838?dopt=AbstractPlus

[28] Ashikov A *et al* (2005) http://www.ncbi.nlm.nih.gov/pubmed/15911612?dopt=AbstractPlus

[29] Assaraf YG *et al* (1998) http://www.ncbi.nlm.nih.gov/pubmed/9525913?dopt=AbstractPlus

[30] Aubrey KR *et al* (2000) http://www.ncbi.nlm.nih.gov/pubmed/10860934?dopt=AbstractPlus

[31] Auerbach SS *et al*. National Toxicology Program: Dept of Health and Human Services.

[32] Azzi A *et al* (1993) http://www.ncbi.nlm.nih.gov/pubmed/8132491?dopt=AbstractPlus

[33] Babu E *et al* (2003) http://www.ncbi.nlm.nih.gov/pubmed/12930836?dopt=AbstractPlus

[34] Bagrov AY *et al* (2009) http://www.ncbi.nlm.nih.gov/pubmed/19325075?dopt=AbstractPlus

[35] Bailey CG *et al* (2011) http://www.ncbi.nlm.nih.gov/pubmed/21123949?dopt=AbstractPlus

[36] Bailey DG *et al* (2007) http://www.ncbi.nlm.nih.gov/pubmed/17301733?dopt=AbstractPlus

[37] Bakos E *et al* (2007) http://www.ncbi.nlm.nih.gov/pubmed/17187268?dopt=AbstractPlus

[38] Baldwin SA *et al* (2005) http://www.ncbi.nlm.nih.gov/pubmed/15701636?dopt=AbstractPlus

[39] Balimane P *et al* (2000) http://www.ncbi.nlm.nih.gov/pubmed/11180195?dopt=AbstractPlus

[40] Balimane PV *et al* (1998) http://www.ncbi.nlm.nih.gov/pubmed/9753615?dopt=AbstractPlus

[41] Ballatori N *et al* (2005) http://www.ncbi.nlm.nih.gov/pubmed/16317684?dopt=AbstractPlus

[42] Banerjee A *et al* (2006) http://www.ncbi.nlm.nih.gov/pubmed/16411770?dopt=AbstractPlus

[43] Barnes K *et al* (2006) http://www.ncbi.nlm.nih.gov/pubmed/16873718?dopt=AbstractPlus

[44] Battini JL *et al* (1999) http://www.ncbi.nlm.nih.gov/pubmed/9990033?dopt=AbstractPlus

[45] Bayeva M *et al* (2013) http://www.ncbi.nlm.nih.gov/pubmed/23720443?dopt=AbstractPlus

[46] Beart PM *et al* (2007) http://www.ncbi.nlm.nih.gov/pubmed/17088867?dopt=AbstractPlus

[47] Belanger AM *et al* (2018) http://www.ncbi.nlm.nih.gov/pubmed/30046012?dopt=AbstractPlus

[48] Bellocchio EE *et al* (2000) http://www.ncbi.nlm.nih.gov/pubmed/10938000?dopt=AbstractPlus

[49] Ben-Daniel R *et al* (2008) http://www.ncbi.nlm.nih.gov/pubmed/18487050?dopt=AbstractPlus

[50] Bergeron R *et al* (1998) http://www.ncbi.nlm.nih.gov/pubmed/9861038?dopt=AbstractPlus

[51] Betz H *et al* (2006) http://www.ncbi.nlm.nih.gov/pubmed/16417482?dopt=AbstractPlus

[52] Bhardwaj RK *et al* (2006) http://www.ncbi.nlm.nih.gov/pubmed/16289537?dopt=AbstractPlus

[53] Bhardwaj RK *et al* (2005) http://www.ncbi.nlm.nih.gov/pubmed/15901802?dopt=AbstractPlus

[54] Bhat BG *et al* (2003) http://www.ncbi.nlm.nih.gov/pubmed/12810816?dopt=AbstractPlus

[55] Bianchi J *et al* (1986) http://www.ncbi.nlm.nih.gov/pubmed/3945643?dopt=AbstractPlus

[56] Biegel A *et al* (2005) http://www.ncbi.nlm.nih.gov/pubmed/15974593?dopt=AbstractPlus

[57] Biegel A *et al* (2006) http://www.ncbi.nlm.nih.gov/pubmed/16868651?dopt=AbstractPlus

[58] Bissonnette P *et al* (2004) http://www.ncbi.nlm.nih.gov/pubmed/15181167?dopt=AbstractPlus

[59] Bizhanova A *et al* (2009) http://www.ncbi.nlm.nih.gov/pubmed/19196800?dopt=AbstractPlus

[60] Blackburn C *et al* (2006) http://www.ncbi.nlm.nih.gov/pubmed/16644217?dopt=AbstractPlus

[61] Blair BG *et al* (2009) http://www.ncbi.nlm.nih.gov/pubmed/19509135?dopt=AbstractPlus

[62] Blom TS *et al* (2003) http://www.ncbi.nlm.nih.gov/pubmed/12554680?dopt=AbstractPlus

[63] Bodmer D *et al* (2002) http://www.ncbi.nlm.nih.gov/pubmed/11912179?dopt=AbstractPlus

[64] Bodoy S *et al* (2005) http://www.ncbi.nlm.nih.gov/pubmed/15659399?dopt=AbstractPlus

[65] Borden LA *et al* (1994) http://www.ncbi.nlm.nih.gov/pubmed/7874447?dopt=AbstractPlus

[66] Borden LA *et al* (1994) http://www.ncbi.nlm.nih.gov/pubmed/7851497?dopt=AbstractPlus

[67] Borst P *et al* (2007) http://www.ncbi.nlm.nih.gov/pubmed/16586096?dopt=AbstractPlus

[68] Boudker O *et al* (2007) http://www.ncbi.nlm.nih.gov/pubmed/17230192?dopt=AbstractPlus

[69] Boulay D *et al* (2008) http://www.ncbi.nlm.nih.gov/pubmed/18621075?dopt=AbstractPlus

[70] Bourgeois F *et al* (2005) http://www.ncbi.nlm.nih.gov/pubmed/15613375?dopt=AbstractPlus

[71] Bravo DT *et al* (2005) http://www.ncbi.nlm.nih.gov/pubmed/15979764?dopt=AbstractPlus

[72] Bravo DT *et al* (2004) http://www.ncbi.nlm.nih.gov/pubmed/15485505?dopt=AbstractPlus

[73] Bricker DK *et al* (2012) http://www.ncbi.nlm.nih.gov/pubmed/22628558?dopt=AbstractPlus

[74] Brown A *et al* (2001) http://www.ncbi.nlm.nih.gov/pubmed/11454468?dopt=AbstractPlus

[75] Bröer A *et al* (2002) http://www.ncbi.nlm.nih.gov/pubmed/11850497?dopt=AbstractPlus

[76] Bröer A *et al* (2009) http://www.ncbi.nlm.nih.gov/pubmed/19657969?dopt=AbstractPlus

[77] Bröer A *et al* (1999) http://www.ncbi.nlm.nih.gov/pubmed/10537079?dopt=AbstractPlus

[78] Bröer A *et al* (2006) http://www.ncbi.nlm.nih.gov/pubmed/16185194?dopt=AbstractPlus

[79] Bröer A *et al* (2000) http://www.ncbi.nlm.nih.gov/pubmed/10698697?dopt=AbstractPlus

[80] Bröer S. (2006) http://www.ncbi.nlm.nih.gov/pubmed/16540203?dopt=AbstractPlus

[81] Bröer S. (2008) http://www.ncbi.nlm.nih.gov/pubmed/18400692?dopt=AbstractPlus

[82] Bröer S *et al* (2008) http://www.ncbi.nlm.nih.gov/pubmed/19033659?dopt=AbstractPlus

[83] Burant CF *et al* (1992) http://www.ncbi.nlm.nih.gov/pubmed/1634504?dopt=AbstractPlus

[84] Burger S *et al* (2011) http://www.ncbi.nlm.nih.gov/pubmed/21742018?dopt=AbstractPlus

[85] Burns CM *et al* (2011) http://www.ncbi.nlm.nih.gov/pubmed/20719377?dopt=AbstractPlus

[86] Busch AE *et al* (1996) http://www.ncbi.nlm.nih.gov/pubmed/8643577?dopt=AbstractPlus

[87] Buyse M *et al* (2001) http://www.ncbi.nlm.nih.gov/pubmed/11714740?dopt=AbstractPlus

[88] Buyse M *et al* (2003) http://www.ncbi.nlm.nih.gov/pubmed/14578196?dopt=AbstractPlus

[89] Byrne JA *et al* (2002) http://www.ncbi.nlm.nih.gov/pubmed/12404239?dopt=AbstractPlus

[90] Böhmer C *et al* (2005) http://www.ncbi.nlm.nih.gov/pubmed/15804236?dopt=AbstractPlus

[91] Carland JE *et al* (2013) http://www.ncbi.nlm.nih.gov/pubmed/22978602?dopt=AbstractPlus

[92] Carr G *et al* (2009) http://www.ncbi.nlm.nih.gov/pubmed/19910700?dopt=AbstractPlus

[93] Carstea ED *et al* (1997) http://www.ncbi.nlm.nih.gov/pubmed/9211849?dopt=AbstractPlus

[94] Caulfield MJ *et al* (2008) http://www.ncbi.nlm.nih.gov/pubmed/18842065?dopt=AbstractPlus

[95] Caulfield WL *et al* (2001) http://www.ncbi.nlm.nih.gov/pubmed/11495577?dopt=AbstractPlus

[96] Cerveny L *et al* (2018) http://www.ncbi.nlm.nih.gov/pubmed/30097436?dopt=AbstractPlus

[97] Cha SH *et al* (2000) http://www.ncbi.nlm.nih.gov/pubmed/10660625?dopt=AbstractPlus

[98] Chan BS *et al* (1998) http://www.ncbi.nlm.nih.gov/pubmed/9506966?dopt=AbstractPlus

[99] Chang MH *et al* (2009) http://www.ncbi.nlm.nih.gov/pubmed/19365592?dopt=AbstractPlus

[100] Chao EC *et al* (2010) http://www.ncbi.nlm.nih.gov/pubmed/20508640?dopt=AbstractPlus

[101] Charkoudian LK *et al* (2012) Natural product inhibitors of glucose-6-phosphate translocase. MedChemComm. 3: 926-931

[102] Charrier L *et al* (2006) http://www.ncbi.nlm.nih.gov/pubmed/16568107?dopt=AbstractPlus

[103] Chavan H *et al* (2015) http://www.ncbi.nlm.nih.gov/pubmed/25623066?dopt=AbstractPlus

[104] Cheeseman C et al. (2008) http://www.ncbi.nlm.nih.gov/pubmed/18477702?dopt=AbstractPlus

[105] Chen LQ *et al* (2010) http://www.ncbi.nlm.nih.gov/pubmed/21107422?dopt=AbstractPlus

[106] Chen NH *et al* (2004) http://www.ncbi.nlm.nih.gov/pubmed/12719981?dopt=AbstractPlus

[107] Chen SY *et al* (2008) http://www.ncbi.nlm.nih.gov/pubmed/18337460?dopt=AbstractPlus

[108] Chen Y *et al* (2007) http://www.ncbi.nlm.nih.gov/pubmed/17495125?dopt=AbstractPlus

[109] Chen Z *et al* (2003) http://www.ncbi.nlm.nih.gov/pubmed/12727219?dopt=AbstractPlus

[110] Chen ZQ *et al* (2006) http://www.ncbi.nlm.nih.gov/pubmed/16421098?dopt=AbstractPlus

[111] Chen ZS *et al* (1999) http://www.ncbi.nlm.nih.gov/pubmed/10570049?dopt=AbstractPlus

[112] Cheng Q *et al* (2017) http://www.ncbi.nlm.nih.gov/pubmed/28176326?dopt=AbstractPlus

[113] Chiabrando D *et al* (2012) http://www.ncbi.nlm.nih.gov/pubmed/23187127?dopt=AbstractPlus

[114] Choi MK. (2012) http://www.ncbi.nlm.nih.gov/pubmed/22644860?dopt=AbstractPlus

[115] Choi MK *et al* (2015) http://www.ncbi.nlm.nih.gov/pubmed/25011570?dopt=AbstractPlus

[116] Chong X *et al* (1992) http://www.ncbi.nlm.nih.gov/pubmed/1417961?dopt=AbstractPlus

[117] Christian WV *et al* (2012) http://www.ncbi.nlm.nih.gov/pubmed/22535958?dopt=AbstractPlus

[118] Chu XY *et al* (2001) http://www.ncbi.nlm.nih.gov/pubmed/11602669?dopt=AbstractPlus

[119] Clausen RP *et al* (2006) http://www.ncbi.nlm.nih.gov/pubmed/17175818?dopt=AbstractPlus

[120] Coady MJ *et al* (2007) http://www.ncbi.nlm.nih.gov/pubmed/17526579?dopt=AbstractPlus

[121] Coady MJ *et al* (2002) http://www.ncbi.nlm.nih.gov/pubmed/12133831?dopt=AbstractPlus

[122] Coelho D *et al* (2012) http://www.ncbi.nlm.nih.gov/pubmed/22922874?dopt=AbstractPlus

[123] Cohen-Kfir E *et al* (2005) http://www.ncbi.nlm.nih.gov/pubmed/15829583?dopt=AbstractPlus

[124] Colleoni S *et al* (2008) http://www.ncbi.nlm.nih.gov/pubmed/18451317?dopt=AbstractPlus

[125] Colton CK *et al* (2010) http://www.ncbi.nlm.nih.gov/pubmed/20508255?dopt=AbstractPlus

[126] Coon *et al* (2004) Abstract Viewer/Itinerary Planner, Program No. 168.10. Society for Neuroscience:

[127] Counillon L *et al* (1993) http://www.ncbi.nlm.nih.gov/pubmed/8246907?dopt=AbstractPlus

[128] Covitz KM *et al* (1996) http://www.ncbi.nlm.nih.gov/pubmed/8956326?dopt=AbstractPlus

[129] Craddock AL *et al* (1998) http://www.ncbi.nlm.nih.gov/pubmed/9458785?dopt=AbstractPlus

[130] Cuboni S *et al* (2014) http://www.ncbi.nlm.nih.gov/pubmed/25318072?dopt=AbstractPlus

[131] Dai W *et al* (1999) http://www.ncbi.nlm.nih.gov/pubmed/9882430?dopt=AbstractPlus

[132] Dalton TP *et al* (2005) http://www.ncbi.nlm.nih.gov/pubmed/15722412?dopt=AbstractPlus

[133] Daniels G *et al* (2015) http://www.ncbi.nlm.nih.gov/pubmed/25896650?dopt=AbstractPlus

[134] Danthi SJ *et al* (2019) http://www.ncbi.nlm.nih.gov/pubmed/30589598?dopt=AbstractPlus

[135] Darcel NP *et al* (2005) http://www.ncbi.nlm.nih.gov/pubmed/15930458?dopt=AbstractPlus 10.1093/jn/135.6.1491 15930458

[136] Davies JP *et al* (2000) http://www.ncbi.nlm.nih.gov/pubmed/10783261?dopt=AbstractPlus

[137] Dawson PA *et al* (2005) http://www.ncbi.nlm.nih.gov/pubmed/15563450?dopt=AbstractPlus

[138] Dawson PA *et al* (2009) http://www.ncbi.nlm.nih.gov/pubmed/19498215?dopt=AbstractPlus

[139] de Carvalho FD *et al* (2011) http://www.ncbi.nlm.nih.gov/pubmed/20980265?dopt=AbstractPlus

[140] De Domenico I *et al* (2007) http://www.ncbi.nlm.nih.gov/pubmed/17077321?dopt=AbstractPlus

[141] De Domenico I *et al* (2005) http://www.ncbi.nlm.nih.gov/pubmed/15956209?dopt=AbstractPlus

[142] Dean M *et al* (2001) http://www.ncbi.nlm.nih.gov/pubmed/11441126?dopt=AbstractPlus

[143] Delpire E *et al* (2009) http://www.ncbi.nlm.nih.gov/pubmed/19279215?dopt=AbstractPlus

[144] Demaegd D *et al* (2013) http://www.ncbi.nlm.nih.gov/pubmed/23569283?dopt=AbstractPlus

[145] Demirel Ö et al et al. (2012) http://www.ncbi.nlm.nih.gov/pubmed/22641697?dopt=AbstractPlus

[146] Dennis M *et al* (2013) Compositions and methods for the diagnosis and treatment of tumor. Patent number: US8535675 B2.

[147] DeStefano GM *et al* (2014) http://www.ncbi.nlm.nih.gov/pubmed/24831815?dopt=AbstractPlus

[148] Dhar TG *et al* (1994) http://www.ncbi.nlm.nih.gov/pubmed/8057281?dopt=AbstractPlus

[149] Dhar TGM *et al* (1996) On the bioactive conformation of the GABA uptake inhibitor SK&F 89976-A. Bioorganic and medicinal chemistry letters 6: 1535-1540

[150] Di Daniel E *et al* (2009) http://www.ncbi.nlm.nih.gov/pubmed/19607714?dopt=AbstractPlus

[151] Diaz GA *et al* (1999) http://www.ncbi.nlm.nih.gov/pubmed/10391223?dopt=AbstractPlus

[152] Dieck ST *et al* (1999) http://www.ncbi.nlm.nih.gov/pubmed/9888294?dopt=AbstractPlus

[153] Diez-Sampedro A *et al* (2003) http://www.ncbi.nlm.nih.gov/pubmed/13130073?dopt=AbstractPlus

[154] Dimmer KS *et al* (2008) http://www.ncbi.nlm.nih.gov/pubmed/17925330?dopt=AbstractPlus

[155] Dodd JR *et al* (2007) http://www.ncbi.nlm.nih.gov/pubmed/17400549?dopt=AbstractPlus

[156] Doege H *et al* (2001) http://www.ncbi.nlm.nih.gov/pubmed/11583593?dopt=AbstractPlus

[157] Dohán O *et al* (2007) http://www.ncbi.nlm.nih.gov/pubmed/18077370?dopt=AbstractPlus

[158] Dong H *et al* (2002) http://www.ncbi.nlm.nih.gov/pubmed/11916852?dopt=AbstractPlus

[159] Dong Z *et al* (2013) http://www.ncbi.nlm.nih.gov/pubmed/23339484?dopt=AbstractPlus

[160] Donovan A *et al* (2005) http://www.ncbi.nlm.nih.gov/pubmed/16054062?dopt=AbstractPlus

[161] Dorwart MR *et al* (2007) http://www.ncbi.nlm.nih.gov/pubmed/17673510?dopt=AbstractPlus

[162] Draoui N *et al* (2013) http://www.ncbi.nlm.nih.gov/pubmed/24095010?dopt=AbstractPlus

[163] Duffy SP *et al* (2010) http://www.ncbi.nlm.nih.gov/pubmed/20823265?dopt=AbstractPlus

[164] Dunlop J. (2006) http://www.ncbi.nlm.nih.gov/pubmed/16368269?dopt=AbstractPlus

[165] Dunlop J *et al* (2006) http://www.ncbi.nlm.nih.gov/pubmed/17017964?dopt=AbstractPlus

[166] Dunlop J *et al* (2003) http://www.ncbi.nlm.nih.gov/pubmed/14517179?dopt=AbstractPlus

[167] Dunlop J *et al* (2005) http://www.ncbi.nlm.nih.gov/pubmed/16014807?dopt=AbstractPlus

[168] Dutta B *et al* (1999) http://www.ncbi.nlm.nih.gov/pubmed/10542220?dopt=AbstractPlus

[169] Döring F *et al* (1998) http://www.ncbi.nlm.nih.gov/pubmed/9637710?dopt=AbstractPlus

[170] Edington AR *et al* (2009) http://www.ncbi.nlm.nih.gov/pubmed/19875446?dopt=AbstractPlus

[171] Edwards N *et al* (2011) http://www.ncbi.nlm.nih.gov/pubmed/20691150?dopt=AbstractPlus

[172] Edwards N *et al* (2018) http://www.ncbi.nlm.nih.gov/pubmed/29058016?dopt=AbstractPlus

[173] Efange SM *et al* (1995) http://www.ncbi.nlm.nih.gov/pubmed/7702637?dopt=AbstractPlus

[174] Eiden LE *et al* (2004) http://www.ncbi.nlm.nih.gov/pubmed/12827358?dopt=AbstractPlus

[175] Eiden LE *et al* (2011) http://www.ncbi.nlm.nih.gov/pubmed/21272013?dopt=AbstractPlus

[176] Eliasof S *et al* (2001) http://www.ncbi.nlm.nih.gov/pubmed/11299317?dopt=AbstractPlus

[177] Elliott AM *et al* (2009) http://www.ncbi.nlm.nih.gov/pubmed/19147539?dopt=AbstractPlus

[178] Endele S *et al* (1999) http://www.ncbi.nlm.nih.gov/pubmed/10486213?dopt=AbstractPlus

[179] Endeward V *et al* (2008) http://www.ncbi.nlm.nih.gov/pubmed/17712059?dopt=AbstractPlus

[180] Engel K *et al* (2005) http://www.ncbi.nlm.nih.gov/pubmed/16099839?dopt=AbstractPlus

[181] Enomoto A *et al* (2002) http://www.ncbi.nlm.nih.gov/pubmed/12024214?dopt=AbstractPlus

[182] Erickson JD *et al* (1993) http://www.ncbi.nlm.nih.gov/pubmed/8245983?dopt=AbstractPlus

[183] Erickson JD *et al* (1996) http://www.ncbi.nlm.nih.gov/pubmed/8643547?dopt=AbstractPlus

[184] Erickson JD *et al* (1994) http://www.ncbi.nlm.nih.gov/pubmed/8071310?dopt=AbstractPlus

[185] Eskandari S *et al* (1997) http://www.ncbi.nlm.nih.gov/pubmed/9341168?dopt=AbstractPlus

[186] Esslinger CS *et al* (2005) http://www.ncbi.nlm.nih.gov/pubmed/16183084?dopt=AbstractPlus

[187] Esslinger CS *et al* (2005) http://www.ncbi.nlm.nih.gov/pubmed/15670919?dopt=AbstractPlus

[188] Etoga JL *et al* (2010) http://www.ncbi.nlm.nih.gov/pubmed/20303751?dopt=AbstractPlus

[189] Eulenburg V *et al* (2005) http://www.ncbi.nlm.nih.gov/pubmed/15950877?dopt=AbstractPlus

[190] Faergeman NJ *et al* (1997) http://www.ncbi.nlm.nih.gov/pubmed/9079682?dopt=AbstractPlus

[191] Fan SJ *et al* (2018) http://www.ncbi.nlm.nih.gov/pubmed/29971004?dopt=AbstractPlus

[192] Fan SJ *et al* (2016) http://www.ncbi.nlm.nih.gov/pubmed/26434594?dopt=AbstractPlus

[193] Fang F *et al* (2010) http://www.ncbi.nlm.nih.gov/pubmed/20649839?dopt=AbstractPlus

[194] Fattorini G *et al* (2009) http://www.ncbi.nlm.nih.gov/pubmed/19627441?dopt=AbstractPlus

[195] Favari E *et al* (2004) http://www.ncbi.nlm.nih.gov/pubmed/15514211?dopt=AbstractPlus

[196] Fehrenbach T *et al* (2003) http://www.ncbi.nlm.nih.gov/pubmed/14530907?dopt=AbstractPlus

[197] Fei YJ *et al* (2000) http://www.ncbi.nlm.nih.gov/pubmed/10823827?dopt=AbstractPlus

[198] Ferdinandusse S *et al* (2015) http://www.ncbi.nlm.nih.gov/pubmed/25168382?dopt=AbstractPlus

[199] Ferguson SM *et al* (2004) http://www.ncbi.nlm.nih.gov/pubmed/15173594?dopt=AbstractPlus

[200] Ferguson SM *et al* (2004) http://www.ncbi.nlm.nih.gov/pubmed/14993474?dopt=AbstractPlus

[201] Fernandes CF *et al* (2007) http://www.ncbi.nlm.nih.gov/pubmed/17632081?dopt=AbstractPlus

[202] Fiermonte G *et al* (2003) http://www.ncbi.nlm.nih.gov/pubmed/12807890?dopt=AbstractPlus

[203] Fiermonte G *et al* (2009) http://www.ncbi.nlm.nih.gov/pubmed/19429682?dopt=AbstractPlus

[204] Fischer WJ *et al* (2005) http://www.ncbi.nlm.nih.gov/pubmed/15737679?dopt=AbstractPlus

[205] Fleming MD *et al* (2001) http://www.ncbi.nlm.nih.gov/pubmed/11274051?dopt=AbstractPlus

[206] Foltz M *et al* (2004) http://www.ncbi.nlm.nih.gov/pubmed/15345686?dopt=AbstractPlus

[207] Fontana AC *et al* (2007) http://www.ncbi.nlm.nih.gov/pubmed/17646426?dopt=AbstractPlus

[208] Fontana AC *et al* (2003) http://www.ncbi.nlm.nih.gov/pubmed/12890709?dopt=AbstractPlus

[209] Forrest LR *et al* (2009) http://www.ncbi.nlm.nih.gov/pubmed/19996368?dopt=AbstractPlus

[210] Forster IC *et al* (1999) http://www.ncbi.nlm.nih.gov/pubmed/10198426?dopt=AbstractPlus

[211] Foulquier F *et al* (2012) http://www.ncbi.nlm.nih.gov/pubmed/22683087?dopt=AbstractPlus

[212] Fredriksson R *et al* (2008) http://www.ncbi.nlm.nih.gov/pubmed/18948099?dopt=AbstractPlus

[213] Friesema EC *et al* (2006) http://www.ncbi.nlm.nih.gov/pubmed/16887882?dopt=AbstractPlus

[214] Froimowitz M *et al* (2007) http://www.ncbi.nlm.nih.gov/pubmed/17228864?dopt=AbstractPlus

[215] Fu Y *et al* (2013) http://www.ncbi.nlm.nih.gov/pubmed/23931754?dopt=AbstractPlus

[216] Fujinami K *et al* (2015) http://www.ncbi.nlm.nih.gov/pubmed/25312043?dopt=AbstractPlus

[217] Fujita T *et al* (2004) http://www.ncbi.nlm.nih.gov/pubmed/14715149?dopt=AbstractPlus

[218] Fülep GH *et al* (2006) http://www.ncbi.nlm.nih.gov/pubmed/16766089?dopt=AbstractPlus

[219] Gabernet L *et al* (2005) http://www.ncbi.nlm.nih.gov/pubmed/15555781?dopt=AbstractPlus

[220] Gameiro A *et al* (2011) http://www.ncbi.nlm.nih.gov/pubmed/21641307?dopt=AbstractPlus

[221] Ganapathy ME *et al* (1995) http://www.ncbi.nlm.nih.gov/pubmed/7592745?dopt=AbstractPlus

[222] Ganapathy ME *et al* (1998) http://www.ncbi.nlm.nih.gov/pubmed/9610386?dopt=AbstractPlus

[223] Ganapathy ME *et al* (1997) http://www.ncbi.nlm.nih.gov/pubmed/9092716?dopt=AbstractPlus

[224] Ganapathy V *et al* (2008) http://www.ncbi.nlm.nih.gov/pubmed/18446519?dopt=AbstractPlus

[225] Ganapathy V *et al* (2009) http://www.ncbi.nlm.nih.gov/pubmed/18992769?dopt=AbstractPlus

[226] Ganel R *et al* (2006) http://www.ncbi.nlm.nih.gov/pubmed/16274998?dopt=AbstractPlus

[227] Garcia-Calvo M *et al* (2005) http://www.ncbi.nlm.nih.gov/pubmed/15928087?dopt=AbstractPlus

[228] Garzel B *et al* (2014) http://www.ncbi.nlm.nih.gov/pubmed/24335466?dopt=AbstractPlus

[229] Gasnier B. (2004) http://www.ncbi.nlm.nih.gov/pubmed/12750892?dopt=AbstractPlus

[230] Gasnier B. (2000) http://www.ncbi.nlm.nih.gov/pubmed/10865121?dopt=AbstractPlus

[231] Gebhardt FM *et al* (2010) http://www.ncbi.nlm.nih.gov/pubmed/20688910?dopt=AbstractPlus

[232] Gendreau S *et al* (2004) http://www.ncbi.nlm.nih.gov/pubmed/15265858?dopt=AbstractPlus

[233] Gengo PJ *et al* (2005) Monoaminergic transporter binding and inhibition profile of dapoxetine, a medication for the treatment of premature ejaculation [abstract]. J Urol 173: Abstract 878

[234] Geyer J *et al* (2007) http://www.ncbi.nlm.nih.gov/pubmed/17491011?dopt=AbstractPlus

[235] Geyer J *et al* (2008) http://www.ncbi.nlm.nih.gov/pubmed/18355966?dopt=AbstractPlus

[236] Geyer J *et al* (2004) http://www.ncbi.nlm.nih.gov/pubmed/15020217?dopt=AbstractPlus

[237] Gillberg P‐G *et al* (2013) Ibat inhibitors for the treatment of liver diseases Patent number: US20130225511A1.

[238] Gimeno RE *et al* (2003) http://www.ncbi.nlm.nih.gov/pubmed/12556534?dopt=AbstractPlus

[239] Giovannini D *et al* (2013) http://www.ncbi.nlm.nih.gov/pubmed/23791524?dopt=AbstractPlus

[240] Girijashanker K *et al* (2008) http://www.ncbi.nlm.nih.gov/pubmed/18270315?dopt=AbstractPlus

[241] Godoy JR *et al* (2007) http://www.ncbi.nlm.nih.gov/pubmed/17628207?dopt=AbstractPlus

[242] Gomeza J *et al* (2006) http://www.ncbi.nlm.nih.gov/pubmed/16722246?dopt=AbstractPlus

[243] Gomeza J *et al* (2003) http://www.ncbi.nlm.nih.gov/pubmed/14622582?dopt=AbstractPlus

[244] Gomeza J *et al* (2003) http://www.ncbi.nlm.nih.gov/pubmed/14622583?dopt=AbstractPlus

[245] Gong Y *et al* (2017) http://www.ncbi.nlm.nih.gov/pubmed/28943923?dopt=AbstractPlus

[246] Gopal E *et al* (2005) http://www.ncbi.nlm.nih.gov/pubmed/15651982?dopt=AbstractPlus

[247] Gorboulev V *et al* (1997) http://www.ncbi.nlm.nih.gov/pubmed/9260930?dopt=AbstractPlus

[248] Goursaud S *et al* (2011) http://www.ncbi.nlm.nih.gov/pubmed/21730107?dopt=AbstractPlus

[249] Goytain A *et al* (2007) http://www.ncbi.nlm.nih.gov/pubmed/17166836?dopt=AbstractPlus

[250] Goytain A *et al* (2008) http://www.ncbi.nlm.nih.gov/pubmed/18667602?dopt=AbstractPlus

[251] Goytain A *et al* (2005) http://www.ncbi.nlm.nih.gov/pubmed/15804357?dopt=AbstractPlus

[252] Goytain A *et al* (2005) http://www.ncbi.nlm.nih.gov/pubmed/15713785?dopt=AbstractPlus

[253] Goytain A *et al* (2005) http://www.ncbi.nlm.nih.gov/pubmed/15809054?dopt=AbstractPlus

[254] Graf J *et al* (2005) http://www.ncbi.nlm.nih.gov/pubmed/15714523?dopt=AbstractPlus

[255] Greer WL *et al* (1999) http://www.ncbi.nlm.nih.gov/pubmed/10521290?dopt=AbstractPlus

[256] Greinacher A *et al* (2010) http://www.ncbi.nlm.nih.gov/pubmed/20037594?dopt=AbstractPlus

[257] Grewer C *et al* (2005) http://www.ncbi.nlm.nih.gov/pubmed/16128593?dopt=AbstractPlus

[258] Grewer C *et al* (2004) http://www.ncbi.nlm.nih.gov/pubmed/15107471?dopt=AbstractPlus

[259] Grewer C *et al* (2005) http://www.ncbi.nlm.nih.gov/pubmed/15834685?dopt=AbstractPlus

[260] Groneberg DA *et al* (2001) http://www.ncbi.nlm.nih.gov/pubmed/11518682?dopt=AbstractPlus

[261] Grozio A *et al* (2019) Slc12a8 is a nicotinamide mononucleotide transporter Nature Metabolism 1: 47-57 10.1038/s42255-018-0009-4 PMC6530925 31131364

[262] Grunewald M *et al* (2000) http://www.ncbi.nlm.nih.gov/pubmed/10734120?dopt=AbstractPlus

[263] Gründemann D *et al* (1999) http://www.ncbi.nlm.nih.gov/pubmed/10385678?dopt=AbstractPlus

[264] Gründemann D *et al* (1998) http://www.ncbi.nlm.nih.gov/pubmed/10196521?dopt=AbstractPlus

[265] Gu H *et al* (1994) http://www.ncbi.nlm.nih.gov/pubmed/8125921?dopt=AbstractPlus

[266] Gu HH *et al* (1996) http://www.ncbi.nlm.nih.gov/pubmed/8636118?dopt=AbstractPlus

[267] Gui C *et al* (2010) http://www.ncbi.nlm.nih.gov/pubmed/20448812?dopt=AbstractPlus

[268] Guile SD *et al* (2006) http://www.ncbi.nlm.nih.gov/pubmed/16455256?dopt=AbstractPlus

[269] Gunshin H *et al* (1997) http://www.ncbi.nlm.nih.gov/pubmed/9242408?dopt=AbstractPlus

[270] Guo A *et al* (1999) http://www.ncbi.nlm.nih.gov/pubmed/10087037?dopt=AbstractPlus

[271] Gupta N *et al* (2006) http://www.ncbi.nlm.nih.gov/pubmed/16375929?dopt=AbstractPlus

[272] Gupte A *et al* (2009) http://www.ncbi.nlm.nih.gov/pubmed/19097778?dopt=AbstractPlus

[273] Hager K *et al* (1995) http://www.ncbi.nlm.nih.gov/pubmed/7537337?dopt=AbstractPlus

[274] Hallén S *et al* (1999) http://www.ncbi.nlm.nih.gov/pubmed/10471288?dopt=AbstractPlus

[275] Hamada T *et al* (2008) http://www.ncbi.nlm.nih.gov/pubmed/18670416?dopt=AbstractPlus

[276] Hammond JR. (2000) http://www.ncbi.nlm.nih.gov/pubmed/10763851?dopt=AbstractPlus

[277] Hammond JR *et al* (2004) http://www.ncbi.nlm.nih.gov/pubmed/14634039?dopt=AbstractPlus

[278] Han H *et al* (1998) http://www.ncbi.nlm.nih.gov/pubmed/9706043?dopt=AbstractPlus

[279] Han X *et al* (2006) http://www.ncbi.nlm.nih.gov/pubmed/16734743?dopt=AbstractPlus

[280] Hannaert P *et al* (2002) http://www.ncbi.nlm.nih.gov/pubmed/11882915?dopt=AbstractPlus

[281] Harvey RJ *et al* (2008) http://www.ncbi.nlm.nih.gov/pubmed/18707791?dopt=AbstractPlus

[282] Hatanaka T *et al* (2001) http://www.ncbi.nlm.nih.gov/pubmed/11342143?dopt=AbstractPlus

[283] Hatanaka T *et al* (2000) http://www.ncbi.nlm.nih.gov/pubmed/10930503?dopt=AbstractPlus

[284] Hatanaka T *et al* (2001) http://www.ncbi.nlm.nih.gov/pubmed/11306607?dopt=AbstractPlus

[285] Helias V *et al* (2012) http://www.ncbi.nlm.nih.gov/pubmed/22246506?dopt=AbstractPlus

[286] Hemker MB *et al* (2003) http://www.ncbi.nlm.nih.gov/pubmed/12846905?dopt=AbstractPlus

[287] Herdon HJ *et al* (2010) http://www.ncbi.nlm.nih.gov/pubmed/20691713?dopt=AbstractPlus

[288] Herrera-Ruiz D *et al* (2004) http://www.ncbi.nlm.nih.gov/pubmed/15832510?dopt=AbstractPlus

[289] Herzig S *et al* (2012) http://www.ncbi.nlm.nih.gov/pubmed/22628554?dopt=AbstractPlus

[290] Hiasa M *et al* (2014) http://www.ncbi.nlm.nih.gov/pubmed/25355561?dopt=AbstractPlus

[291] Hirschfield GM *et al* (2013) http://www.ncbi.nlm.nih.gov/pubmed/23583734?dopt=AbstractPlus

[292] Ho AM *et al* (2000) http://www.ncbi.nlm.nih.gov/pubmed/10894769?dopt=AbstractPlus

[293] Ho HT *et al* (2011) http://www.ncbi.nlm.nih.gov/pubmed/21816955?dopt=AbstractPlus

[294] Hollingworth P *et al* (2011) http://www.ncbi.nlm.nih.gov/pubmed/21460840?dopt=AbstractPlus

[295] Horiba N *et al* (2003) http://www.ncbi.nlm.nih.gov/pubmed/12590146?dopt=AbstractPlus

[296] Hsu CL *et al* (2012) http://www.ncbi.nlm.nih.gov/pubmed/22174130?dopt=AbstractPlus

[297] Hu Y *et al* (2018) http://www.ncbi.nlm.nih.gov/pubmed/29784761?dopt=AbstractPlus

[298] Hu Y *et al* (2014) http://www.ncbi.nlm.nih.gov/pubmed/24548120?dopt=AbstractPlus

[299] Hu Z *et al* (2000) http://www.ncbi.nlm.nih.gov/pubmed/10615129?dopt=AbstractPlus

[300] Huang S *et al* (2009) http://www.ncbi.nlm.nih.gov/pubmed/19074430?dopt=AbstractPlus

[301] Hummel CS *et al* (2011) http://www.ncbi.nlm.nih.gov/pubmed/20980548?dopt=AbstractPlus

[302] Hägglund MG *et al* (2011) http://www.ncbi.nlm.nih.gov/pubmed/21511949?dopt=AbstractPlus

[303] Ibberson M *et al* (2000) http://www.ncbi.nlm.nih.gov/pubmed/10671487?dopt=AbstractPlus

[304] Ichida K *et al* (2003) http://www.ncbi.nlm.nih.gov/pubmed/12472777?dopt=AbstractPlus

[305] Ichikawa Y *et al* (2012) http://www.ncbi.nlm.nih.gov/pubmed/22375032?dopt=AbstractPlus

[306] Iharada M *et al* (2010) http://www.ncbi.nlm.nih.gov/pubmed/20566650?dopt=AbstractPlus

[307] Inagaki N *et al* (1995) http://www.ncbi.nlm.nih.gov/pubmed/7502040?dopt=AbstractPlus

[308] Inazu M *et al* (2005) http://www.ncbi.nlm.nih.gov/pubmed/16000150?dopt=AbstractPlus

[309] Infante RE *et al* (2008) http://www.ncbi.nlm.nih.gov/pubmed/17989073?dopt=AbstractPlus

[310] Infante RE *et al* (2008) http://www.ncbi.nlm.nih.gov/pubmed/17989072?dopt=AbstractPlus

[311] Inoue T *et al* (2017) http://www.ncbi.nlm.nih.gov/pubmed/28410751?dopt=AbstractPlus

[312] Irie M *et al* (2001) http://www.ncbi.nlm.nih.gov/pubmed/11454935?dopt=AbstractPlus

[313] Ishida N *et al* (1998) http://www.ncbi.nlm.nih.gov/pubmed/9644260?dopt=AbstractPlus

[314] Ishida N *et al* (2005) http://www.ncbi.nlm.nih.gov/pubmed/15607426?dopt=AbstractPlus

[315] Ishida N *et al* (1996) http://www.ncbi.nlm.nih.gov/pubmed/9010752?dopt=AbstractPlus

[316] Ishida N *et al* (1999) http://www.ncbi.nlm.nih.gov/pubmed/10393322?dopt=AbstractPlus

[317] Ishida S *et al* (2002) http://www.ncbi.nlm.nih.gov/pubmed/12370430?dopt=AbstractPlus

[318] Ismair MG *et al* (2006) http://www.ncbi.nlm.nih.gov/pubmed/17487240?dopt=AbstractPlus

[319] Itagaki S *et al* (2006) http://www.ncbi.nlm.nih.gov/pubmed/16729224?dopt=AbstractPlus

[320] Ito S *et al* (2010) http://www.ncbi.nlm.nih.gov/pubmed/20065018?dopt=AbstractPlus

[321] Ito Y *et al* (2001) http://www.ncbi.nlm.nih.gov/pubmed/11527541?dopt=AbstractPlus

[322] Iwamoto H *et al* (2006) http://www.ncbi.nlm.nih.gov/pubmed/17005849?dopt=AbstractPlus

[323] Iwamoto T *et al* (2006) http://www.ncbi.nlm.nih.gov/pubmed/16973719?dopt=AbstractPlus

[324] Iwao T *et al* (2014) http://www.ncbi.nlm.nih.gov/pubmed/23822979?dopt=AbstractPlus

[325] Jappar D *et al* (2010) http://www.ncbi.nlm.nih.gov/pubmed/20660104?dopt=AbstractPlus

[326] Jensen AA *et al* (2009) http://www.ncbi.nlm.nih.gov/pubmed/19161278?dopt=AbstractPlus

[327] Jeong HJ *et al* (2010) http://www.ncbi.nlm.nih.gov/pubmed/20860669?dopt=AbstractPlus

[328] Jiang D *et al* (2009) http://www.ncbi.nlm.nih.gov/pubmed/19797662?dopt=AbstractPlus

[329] Jiang J *et al* (2011) http://www.ncbi.nlm.nih.gov/pubmed/20708631?dopt=AbstractPlus

[330] Jonas MC *et al* (2010) http://www.ncbi.nlm.nih.gov/pubmed/20826464?dopt=AbstractPlus

[331] Jost N *et al* (2013) http://www.ncbi.nlm.nih.gov/pubmed/23647096?dopt=AbstractPlus

[332] Ju P *et al* (2004) http://www.ncbi.nlm.nih.gov/pubmed/15031290?dopt=AbstractPlus

[333] Juge N *et al* (2010) http://www.ncbi.nlm.nih.gov/pubmed/20920794?dopt=AbstractPlus

[334] Juge N *et al* (2009) http://www.ncbi.nlm.nih.gov/pubmed/19843525?dopt=AbstractPlus

[335] Kamiyama S *et al* (2006) http://www.ncbi.nlm.nih.gov/pubmed/16492677?dopt=AbstractPlus

[336] Kamiyama S *et al* (2003) http://www.ncbi.nlm.nih.gov/pubmed/12716889?dopt=AbstractPlus

[337] Kanai N *et al* (1995) http://www.ncbi.nlm.nih.gov/pubmed/7754369?dopt=AbstractPlus

[338] Kanai Y *et al* (2004) http://www.ncbi.nlm.nih.gov/pubmed/14530974?dopt=AbstractPlus

[339] Kanai Y *et al* (2003) http://www.ncbi.nlm.nih.gov/pubmed/14612154?dopt=AbstractPlus

[340] Kanai Y *et al* (1994) http://www.ncbi.nlm.nih.gov/pubmed/8282810?dopt=AbstractPlus

[341] Kanamori A *et al* (1997) http://www.ncbi.nlm.nih.gov/pubmed/9096318?dopt=AbstractPlus

[342] Kang N *et al* (2010) http://www.ncbi.nlm.nih.gov/pubmed/20595384?dopt=AbstractPlus

[343] Kang SY *et al* (2010) http://www.ncbi.nlm.nih.gov/pubmed/20637636?dopt=AbstractPlus

[344] Karlgren M *et al* (2012) http://www.ncbi.nlm.nih.gov/pubmed/22541068?dopt=AbstractPlus

[345] Karunakaran S *et al* (2008) http://www.ncbi.nlm.nih.gov/pubmed/18522536?dopt=AbstractPlus

[346] Kato Y *et al* (2017) Identification of a vesicular ATP release inhibitor for the treatment of neuropathic and inflammatory pain Proceedings of the National Academy of Sciences of the United States of America 10.1073/pnas.1704847114 PMC5547629 28720702

[347] Kemp S *et al* (2016) http://www.ncbi.nlm.nih.gov/pubmed/27312864?dopt=AbstractPlus

[348] Kemp S *et al* (2011) http://www.ncbi.nlm.nih.gov/pubmed/21488864?dopt=AbstractPlus

[349] Kenda BM *et al* (2004) http://www.ncbi.nlm.nih.gov/pubmed/14736235?dopt=AbstractPlus

[350] Kennedy DJ *et al* (2005) http://www.ncbi.nlm.nih.gov/pubmed/15644866?dopt=AbstractPlus

[351] Kerr ID *et al* (2011) http://www.ncbi.nlm.nih.gov/pubmed/21175590?dopt=AbstractPlus

[352] Khare P *et al* (2010) http://www.ncbi.nlm.nih.gov/pubmed/20225888?dopt=AbstractPlus

[353] Kim K *et al* (2011) http://www.ncbi.nlm.nih.gov/pubmed/21792905?dopt=AbstractPlus

[354] Kim KH *et al* (2005) http://www.ncbi.nlm.nih.gov/pubmed/15591059?dopt=AbstractPlus

[355] Kim RB *et al* (1999) http://www.ncbi.nlm.nih.gov/pubmed/10565843?dopt=AbstractPlus

[356] Kimura H *et al* (2002) http://www.ncbi.nlm.nih.gov/pubmed/11907186?dopt=AbstractPlus

[357] Klaassen CD *et al* (2010) http://www.ncbi.nlm.nih.gov/pubmed/20103563?dopt=AbstractPlus

[358] Klitgaard H *et al* (2007) http://www.ncbi.nlm.nih.gov/pubmed/23484603?dopt=AbstractPlus

[359] Knutsen LJ *et al* (1999) http://www.ncbi.nlm.nih.gov/pubmed/10479278?dopt=AbstractPlus

[360] Knütter I *et al* (2004) http://www.ncbi.nlm.nih.gov/pubmed/14706812?dopt=AbstractPlus

[361] Knütter I *et al* (2009) http://www.ncbi.nlm.nih.gov/pubmed/18824524?dopt=AbstractPlus

[362] Knütter I *et al* (2001) http://www.ncbi.nlm.nih.gov/pubmed/11284702?dopt=AbstractPlus

[363] Knütter I *et al* (2008) http://www.ncbi.nlm.nih.gov/pubmed/18713951?dopt=AbstractPlus

[364] Kobayashi N *et al* (2018) http://www.ncbi.nlm.nih.gov/pubmed/29563527?dopt=AbstractPlus

[365] Kobayashi T *et al* (2014) http://www.ncbi.nlm.nih.gov/pubmed/25238095?dopt=AbstractPlus

[366] Koch HP *et al* (2007) http://www.ncbi.nlm.nih.gov/pubmed/17360917?dopt=AbstractPlus

[367] Koch HP *et al* (1999) http://www.ncbi.nlm.nih.gov/pubmed/10570036?dopt=AbstractPlus

[368] Koepsell H. (2013) http://www.ncbi.nlm.nih.gov/pubmed/23506881?dopt=AbstractPlus

[369] Kolisek M *et al* (2012) http://www.ncbi.nlm.nih.gov/pubmed/22031603?dopt=AbstractPlus

[370] Kommareddi PK *et al* (2010) http://www.ncbi.nlm.nih.gov/pubmed/20665236?dopt=AbstractPlus

[371] Kory N *et al* (2018) http://www.ncbi.nlm.nih.gov/pubmed/30442778?dopt=AbstractPlus

[372] Kottra G *et al* (2013) http://www.ncbi.nlm.nih.gov/pubmed/24744852?dopt=AbstractPlus

[373] Kouji H *et al* (2009) http://www.ncbi.nlm.nih.gov/pubmed/19135976?dopt=AbstractPlus

[374] Krishnamurthy PC *et al* (2006) http://www.ncbi.nlm.nih.gov/pubmed/17006453?dopt=AbstractPlus

[375] Krishnaswamy A *et al* (2009) http://www.ncbi.nlm.nih.gov/pubmed/19186169?dopt=AbstractPlus

[376] Kristensen AS *et al* (2011) http://www.ncbi.nlm.nih.gov/pubmed/21752877?dopt=AbstractPlus

[377] Ksander BR *et al* (2014) http://www.ncbi.nlm.nih.gov/pubmed/25030174?dopt=AbstractPlus

[378] Kung MP *et al* (1994) http://www.ncbi.nlm.nih.gov/pubmed/7855735?dopt=AbstractPlus

[379] Kusuhara H *et al* (1999) http://www.ncbi.nlm.nih.gov/pubmed/10224140?dopt=AbstractPlus

[380] Kvist T *et al* (2009) http://www.ncbi.nlm.nih.gov/pubmed/19275529?dopt=AbstractPlus

[381] Lapinsky DJ *et al* (2011) http://www.ncbi.nlm.nih.gov/pubmed/21129986?dopt=AbstractPlus

[382] Larráyoz IM *et al* (2006) http://www.ncbi.nlm.nih.gov/pubmed/16837649?dopt=AbstractPlus

[383] Larsen M *et al* (2009) http://www.ncbi.nlm.nih.gov/pubmed/19594759?dopt=AbstractPlus

[384] Lau CL *et al* (2011) http://www.ncbi.nlm.nih.gov/pubmed/21309758?dopt=AbstractPlus

[385] Leary GP *et al* (2007) http://www.ncbi.nlm.nih.gov/pubmed/17360916?dopt=AbstractPlus

[386] Lee A *et al* (2010) http://www.ncbi.nlm.nih.gov/pubmed/20883814?dopt=AbstractPlus

[387] Lee J *et al* (2002) http://www.ncbi.nlm.nih.gov/pubmed/11734551?dopt=AbstractPlus

[388] Lee J *et al* (2009) http://www.ncbi.nlm.nih.gov/pubmed/19570976?dopt=AbstractPlus

[389] Lee JY *et al* (2016) http://www.ncbi.nlm.nih.gov/pubmed/27144356?dopt=AbstractPlus

[390] Lee SG *et al* (2008) http://www.ncbi.nlm.nih.gov/pubmed/18326497?dopt=AbstractPlus

[391] Lee SH *et al* (2008) http://www.ncbi.nlm.nih.gov/pubmed/18269914?dopt=AbstractPlus

[392] Lee YC *et al* (2010) http://www.ncbi.nlm.nih.gov/pubmed/20639396?dopt=AbstractPlus

[393] Legati A *et al* (2015) http://www.ncbi.nlm.nih.gov/pubmed/25938945?dopt=AbstractPlus

[394] Leier I *et al* (1994) http://www.ncbi.nlm.nih.gov/pubmed/7961706?dopt=AbstractPlus

[395] Leung DDM *et al* (2010) Anti-ferroportin monoclonal antibodies and uses thereof. Patent number: WO2010065496 A1.

[396] Levy LM *et al* (1998) http://www.ncbi.nlm.nih.gov/pubmed/9822723?dopt=AbstractPlus

[397] Lewis SE *et al* (2001) http://www.ncbi.nlm.nih.gov/pubmed/11470793?dopt=AbstractPlus

[398] Li H *et al* (2008) http://www.ncbi.nlm.nih.gov/pubmed/17928635?dopt=AbstractPlus

[399] Li M *et al* (2006) http://www.ncbi.nlm.nih.gov/pubmed/16434549?dopt=AbstractPlus

[400] Li N *et al* (2007) http://www.ncbi.nlm.nih.gov/pubmed/17650074?dopt=AbstractPlus

[401] Lin P *et al* (2008) http://www.ncbi.nlm.nih.gov/pubmed/19061983?dopt=AbstractPlus

[402] Lin X *et al* (2009) http://www.ncbi.nlm.nih.gov/pubmed/19032932?dopt=AbstractPlus

[403] Lipovich L *et al* (2002) http://www.ncbi.nlm.nih.gov/pubmed/11943475?dopt=AbstractPlus

[404] Liu H *et al* (2008) http://www.ncbi.nlm.nih.gov/pubmed/18983139?dopt=AbstractPlus

[405] Liu W *et al* (1995) http://www.ncbi.nlm.nih.gov/pubmed/7756356?dopt=AbstractPlus

[406] Liu Y *et al* (2013) http://www.ncbi.nlm.nih.gov/pubmed/23597791?dopt=AbstractPlus

[407] Liu Z *et al* (2008) http://www.ncbi.nlm.nih.gov/pubmed/18037372?dopt=AbstractPlus

[408] Liuzzi JP *et al* (2006) http://www.ncbi.nlm.nih.gov/pubmed/16950869?dopt=AbstractPlus

[409] Longo N *et al* (2016) http://www.ncbi.nlm.nih.gov/pubmed/26828774?dopt=AbstractPlus

[410] Lowe 3rd JA *et al* (2003) http://www.ncbi.nlm.nih.gov/pubmed/12657266?dopt=AbstractPlus

[411] Lowe 3rd JA *et al* (2009) http://www.ncbi.nlm.nih.gov/pubmed/19410451?dopt=AbstractPlus

[412] Lu X *et al* (2016) http://www.ncbi.nlm.nih.gov/pubmed/26494147?dopt=AbstractPlus

[413] Luckner P *et al* (2005) http://www.ncbi.nlm.nih.gov/pubmed/15567297?dopt=AbstractPlus 10.1016/j.ejpb.2004.07.008 15567297

[414] Lytton J *et al* (1991) http://www.ncbi.nlm.nih.gov/pubmed/1832668?dopt=AbstractPlus

[415] Löscher W *et al* (2016) http://www.ncbi.nlm.nih.gov/pubmed/27752944?dopt=AbstractPlus

[416] Lühn K *et al* (2001) http://www.ncbi.nlm.nih.gov/pubmed/11326279?dopt=AbstractPlus

[417] MacDonald L *et al* (2002) http://www.ncbi.nlm.nih.gov/pubmed/11895172?dopt=AbstractPlus

[418] MacGrogan D *et al* (1996) http://www.ncbi.nlm.nih.gov/pubmed/8661104?dopt=AbstractPlus

[419] Machtens JP *et al* (2011) http://www.ncbi.nlm.nih.gov/pubmed/21572047?dopt=AbstractPlus

[420] Maciver B *et al* (2008) http://www.ncbi.nlm.nih.gov/pubmed/18256317?dopt=AbstractPlus

[421] Madeo M *et al* (2014) http://www.ncbi.nlm.nih.gov/pubmed/25326386?dopt=AbstractPlus

[422] Madsen KK *et al* (2010) http://www.ncbi.nlm.nih.gov/pubmed/20026354?dopt=AbstractPlus

[423] Mak DO *et al* (2006) http://www.ncbi.nlm.nih.gov/pubmed/16131648?dopt=AbstractPlus

[424] Mallorga PJ *et al* (2003) http://www.ncbi.nlm.nih.gov/pubmed/12941372?dopt=AbstractPlus

[425] Mandal A *et al* (2016) http://www.ncbi.nlm.nih.gov/pubmed/27543355?dopt=AbstractPlus

[426] Manolescu AR *et al* (2007) http://www.ncbi.nlm.nih.gov/pubmed/17710649?dopt=AbstractPlus

[427] Martens H *et al* (2008) http://www.ncbi.nlm.nih.gov/pubmed/19052203?dopt=AbstractPlus

[428] Masuda S *et al* (2006) http://www.ncbi.nlm.nih.gov/pubmed/16807400?dopt=AbstractPlus

[429] McIntire SL *et al* (1997) http://www.ncbi.nlm.nih.gov/pubmed/9349821?dopt=AbstractPlus

[430] Meier PJ *et al* (1997) http://www.ncbi.nlm.nih.gov/pubmed/9398014?dopt=AbstractPlus

[431] Meredith D *et al* (1998) http://www.ncbi.nlm.nih.gov/pubmed/9882198?dopt=AbstractPlus

[432] Merlin D *et al* (2001) http://www.ncbi.nlm.nih.gov/pubmed/11375948?dopt=AbstractPlus

[433] Merlin D *et al* (1998) http://www.ncbi.nlm.nih.gov/pubmed/9835627?dopt=AbstractPlus

[434] Metzner L *et al* (2005) http://www.ncbi.nlm.nih.gov/pubmed/16126914?dopt=AbstractPlus

[435] Meyer E *et al* (2010) http://www.ncbi.nlm.nih.gov/pubmed/20206334?dopt=AbstractPlus

[436] Mezler M *et al* (2008) http://www.ncbi.nlm.nih.gov/pubmed/18815213?dopt=AbstractPlus

[437] Michel V *et al* (2009) http://www.ncbi.nlm.nih.gov/pubmed/19357133?dopt=AbstractPlus

[438] Michel V *et al* (2006) http://www.ncbi.nlm.nih.gov/pubmed/16636297?dopt=AbstractPlus

[439] Mihalik SJ *et al* (2002) http://www.ncbi.nlm.nih.gov/pubmed/11980911?dopt=AbstractPlus

[440] Miki T *et al* (1999) http://www.ncbi.nlm.nih.gov/pubmed/10194514?dopt=AbstractPlus

[441] Milger K *et al* (2006) http://www.ncbi.nlm.nih.gov/pubmed/17062637?dopt=AbstractPlus

[442] Mimura Y *et al* (2017) http://www.ncbi.nlm.nih.gov/pubmed/28089688?dopt=AbstractPlus

[443] Mistrík P *et al* (2012) http://www.ncbi.nlm.nih.gov/pubmed/22890707?dopt=AbstractPlus

[444] Mitsuoka K *et al* (2008) http://www.ncbi.nlm.nih.gov/pubmed/18344442?dopt=AbstractPlus

[445] Miura N *et al* (1996) http://www.ncbi.nlm.nih.gov/pubmed/8889805?dopt=AbstractPlus

[446] Miyaji T *et al* (2011) http://www.ncbi.nlm.nih.gov/pubmed/21781115?dopt=AbstractPlus

[447] Miyauchi S *et al* (2004) http://www.ncbi.nlm.nih.gov/pubmed/14966140?dopt=AbstractPlus

[448] Miyazaki E *et al* (2001) http://www.ncbi.nlm.nih.gov/pubmed/11641397?dopt=AbstractPlus

[449] Mladenova G *et al* (2012) http://www.ncbi.nlm.nih.gov/pubmed/22420844?dopt=AbstractPlus

[450] Morgan RE *et al* (2010) http://www.ncbi.nlm.nih.gov/pubmed/20829430?dopt=AbstractPlus

[451] Murakami Y *et al* (2005) http://www.ncbi.nlm.nih.gov/pubmed/16174808?dopt=AbstractPlus

[452] Muraoka M *et al* (2001) http://www.ncbi.nlm.nih.gov/pubmed/11322953?dopt=AbstractPlus

[453] Nabulsi NB *et al* (2005) http://www.ncbi.nlm.nih.gov/pubmed/15781409?dopt=AbstractPlus

[454] Nair TS *et al* (2004) http://www.ncbi.nlm.nih.gov/pubmed/14973250?dopt=AbstractPlus

[455] Nakai Y *et al* (2007) http://www.ncbi.nlm.nih.gov/pubmed/17475902?dopt=AbstractPlus

[456] Nakamura N *et al* (2014) http://www.ncbi.nlm.nih.gov/pubmed/24695226?dopt=AbstractPlus

[457] Nakamura N *et al* (2005) http://www.ncbi.nlm.nih.gov/pubmed/15522866?dopt=AbstractPlus

[458] Nakamura T *et al* (2010) http://www.ncbi.nlm.nih.gov/pubmed/20410607?dopt=AbstractPlus

[459] Nakanishi T *et al* (2001) http://www.ncbi.nlm.nih.gov/pubmed/11243884?dopt=AbstractPlus

[460] Nawata CM *et al* (2015) http://www.ncbi.nlm.nih.gov/pubmed/26423860?dopt=AbstractPlus

[461] Neumann J *et al* (2003) http://www.ncbi.nlm.nih.gov/pubmed/12649372?dopt=AbstractPlus

[462] Neumann J *et al* (2004) http://www.ncbi.nlm.nih.gov/pubmed/15128310?dopt=AbstractPlus

[463] Newton JM *et al* (2001) http://www.ncbi.nlm.nih.gov/pubmed/11574907?dopt=AbstractPlus

[464] Nguyen LN *et al* (2014) http://www.ncbi.nlm.nih.gov/pubmed/24828044?dopt=AbstractPlus

[465] Nicolas JM *et al* (2016) http://www.ncbi.nlm.nih.gov/pubmed/26663401?dopt=AbstractPlus

[466] Nigam SK *et al* (2018) http://www.ncbi.nlm.nih.gov/pubmed/29847376?dopt=AbstractPlus

[467] Nothmann D *et al* (2011) http://www.ncbi.nlm.nih.gov/pubmed/21127051?dopt=AbstractPlus

[468] Nowikovsky K *et al* (2004) http://www.ncbi.nlm.nih.gov/pubmed/15138253?dopt=AbstractPlus

[469] Nowikovsky K *et al* (2007) http://www.ncbi.nlm.nih.gov/pubmed/17541427?dopt=AbstractPlus

[470] Noyer M *et al* (1995) http://www.ncbi.nlm.nih.gov/pubmed/8605950?dopt=AbstractPlus

[471] Noé J *et al* (2007) http://www.ncbi.nlm.nih.gov/pubmed/17470528?dopt=AbstractPlus

[472] Numata M *et al* (2001) http://www.ncbi.nlm.nih.gov/pubmed/11279194?dopt=AbstractPlus

[473] Núñez E *et al* (2000) http://www.ncbi.nlm.nih.gov/pubmed/10694221?dopt=AbstractPlus

[474] O'Callaghan KM *et al* (2010) http://www.ncbi.nlm.nih.gov/pubmed/19875448?dopt=AbstractPlus

[475] Ocheltree SM *et al* (2004) http://www.ncbi.nlm.nih.gov/pubmed/14600253?dopt=AbstractPlus

[476] Oda K *et al* (2010) http://www.ncbi.nlm.nih.gov/pubmed/19900191?dopt=AbstractPlus

[477] Ohta KY *et al* (2006) http://www.ncbi.nlm.nih.gov/pubmed/16928787?dopt=AbstractPlus

[478] Okuda T *et al* (2003) http://www.ncbi.nlm.nih.gov/pubmed/12675135?dopt=AbstractPlus

[479] Okuda T *et al* (2000) http://www.ncbi.nlm.nih.gov/pubmed/11068039?dopt=AbstractPlus

[480] Oppedisano F *et al* (2010) http://www.ncbi.nlm.nih.gov/pubmed/20599776?dopt=AbstractPlus

[481] Ordovás L *et al* (2006) http://www.ncbi.nlm.nih.gov/pubmed/17065791?dopt=AbstractPlus

[482] Otsuka M *et al* (2005) http://www.ncbi.nlm.nih.gov/pubmed/16330770?dopt=AbstractPlus

[483] Otter M *et al* (2017) http://www.ncbi.nlm.nih.gov/pubmed/27903454?dopt=AbstractPlus

[484] Oude Elferink RP *et al* (2007) http://www.ncbi.nlm.nih.gov/pubmed/16622704?dopt=AbstractPlus

[485] Owen RP *et al* (2006) http://www.ncbi.nlm.nih.gov/pubmed/16840788?dopt=AbstractPlus

[486] Ozvegy C *et al* (2001) http://www.ncbi.nlm.nih.gov/pubmed/11437380?dopt=AbstractPlus

[487] Palacín M *et al* (1998) http://www.ncbi.nlm.nih.gov/pubmed/9790568?dopt=AbstractPlus

[488] Pan CJ *et al* (2011) http://www.ncbi.nlm.nih.gov/pubmed/21949678?dopt=AbstractPlus

[489] Pao SS *et al* (1998) http://www.ncbi.nlm.nih.gov/pubmed/9529885?dopt=AbstractPlus

[490] Paytubi S *et al* (2009) http://www.ncbi.nlm.nih.gov/pubmed/19570978?dopt=AbstractPlus

[491] Pearlman RJ *et al* (2003) http://www.ncbi.nlm.nih.gov/pubmed/12558979?dopt=AbstractPlus

[492] Perland E *et al* (2017) http://www.ncbi.nlm.nih.gov/pubmed/28878041?dopt=AbstractPlus

[493] Perland E *et al* (2017) http://www.ncbi.nlm.nih.gov/pubmed/27939446?dopt=AbstractPlus

[494] Perland E *et al* (2016) http://www.ncbi.nlm.nih.gov/pubmed/27272503?dopt=AbstractPlus

[495] Pestov NB *et al* (2006) http://www.ncbi.nlm.nih.gov/pubmed/16525125?dopt=AbstractPlus

[496] Pfeffer SR. (2016) http://www.ncbi.nlm.nih.gov/pubmed/27410046?dopt=AbstractPlus

[497] Pillai SM *et al* (2011) http://www.ncbi.nlm.nih.gov/pubmed/21097500?dopt=AbstractPlus

[498] Pinard E *et al* (2010) http://www.ncbi.nlm.nih.gov/pubmed/20491477?dopt=AbstractPlus

[499] Pinilla-Tenas J *et al* (2003) http://www.ncbi.nlm.nih.gov/pubmed/14502423?dopt=AbstractPlus 10.1007/s00232-003-2041-9 14502423

[500] Pochini L *et al* (2014) http://www.ncbi.nlm.nih.gov/pubmed/24704252?dopt=AbstractPlus

[501] Pondarre C *et al* (2007) http://www.ncbi.nlm.nih.gov/pubmed/17192398?dopt=AbstractPlus

[502] Pondarré C *et al* (2006) http://www.ncbi.nlm.nih.gov/pubmed/16467350?dopt=AbstractPlus

[503] Potelle S *et al* (2017) http://www.ncbi.nlm.nih.gov/pubmed/28270545?dopt=AbstractPlus

[504] Potelle S *et al* (2016) http://www.ncbi.nlm.nih.gov/pubmed/27008884?dopt=AbstractPlus

[505] Prasad PD *et al* (1995) http://www.ncbi.nlm.nih.gov/pubmed/7826387?dopt=AbstractPlus

[506] Prasad PD *et al* (2000) http://www.ncbi.nlm.nih.gov/pubmed/10772912?dopt=AbstractPlus

[507] Priebe W *et al* (1998) http://www.ncbi.nlm.nih.gov/pubmed/9647783?dopt=AbstractPlus

[508] Pristupa ZB *et al* (1994) http://www.ncbi.nlm.nih.gov/pubmed/8302271?dopt=AbstractPlus

[509] Pérez-Siles G *et al* (2011) http://www.ncbi.nlm.nih.gov/pubmed/21574997?dopt=AbstractPlus

[510] Qiu A *et al* (2006) http://www.ncbi.nlm.nih.gov/pubmed/17129779?dopt=AbstractPlus

[511] Quamme GA. (2010) http://www.ncbi.nlm.nih.gov/pubmed/19940067?dopt=AbstractPlus

[512] Quazi F *et al* (2014) http://www.ncbi.nlm.nih.gov/pubmed/24707049?dopt=AbstractPlus

[513] Quigley JG *et al* (2004) http://www.ncbi.nlm.nih.gov/pubmed/15369674?dopt=AbstractPlus

[514] Raffel DM *et al* (2004) http://www.ncbi.nlm.nih.gov/pubmed/15300361?dopt=AbstractPlus

[515] Rainier S *et al* (2003) http://www.ncbi.nlm.nih.gov/pubmed/14508710?dopt=AbstractPlus

[516] Rajadhyaksha AM *et al* (2010) http://www.ncbi.nlm.nih.gov/pubmed/21070897?dopt=AbstractPlus

[517] Rajagopal A *et al* (2008) http://www.ncbi.nlm.nih.gov/pubmed/18418376?dopt=AbstractPlus

[518] Rajgopal A *et al* (2001) http://www.ncbi.nlm.nih.gov/pubmed/11731220?dopt=AbstractPlus

[519] Ravera S *et al* (2007) http://www.ncbi.nlm.nih.gov/pubmed/17494632?dopt=AbstractPlus

[520] Reddy VS *et al* (2012) http://www.ncbi.nlm.nih.gov/pubmed/22458847?dopt=AbstractPlus

[521] Rees EM *et al* (2004) http://www.ncbi.nlm.nih.gov/pubmed/15494390?dopt=AbstractPlus

[522] Rehmann H. (2012) http://www.ncbi.nlm.nih.gov/pubmed/22260657?dopt=AbstractPlus

[523] Reid G *et al* (2003) http://www.ncbi.nlm.nih.gov/pubmed/12835412?dopt=AbstractPlus

[524] Reith ME *et al* (1996) http://www.ncbi.nlm.nih.gov/pubmed/8878059?dopt=AbstractPlus

[525] Rey MA *et al* (2008) http://www.ncbi.nlm.nih.gov/pubmed/18815190?dopt=AbstractPlus

[526] Reyes N *et al* (2009) http://www.ncbi.nlm.nih.gov/pubmed/19924125?dopt=AbstractPlus

[527] Rice AE *et al* (2009) http://www.ncbi.nlm.nih.gov/pubmed/19150361?dopt=AbstractPlus

[528] Ripoche P *et al* (2004) http://www.ncbi.nlm.nih.gov/pubmed/15572441?dopt=AbstractPlus

[529] Rodriguez L *et al* (2006) http://www.ncbi.nlm.nih.gov/pubmed/16632403?dopt=AbstractPlus

[530] Rogers S *et al* (2003) http://www.ncbi.nlm.nih.gov/pubmed/12914765?dopt=AbstractPlus

[531] Romano A *et al* (2010) http://www.ncbi.nlm.nih.gov/pubmed/19913073?dopt=AbstractPlus

[532] Romera C *et al* (2007) http://www.ncbi.nlm.nih.gov/pubmed/17213861?dopt=AbstractPlus

[533] Rose EM *et al* (2009) http://www.ncbi.nlm.nih.gov/pubmed/19553454?dopt=AbstractPlus

[534] Rosowsky A *et al* (2004) http://www.ncbi.nlm.nih.gov/pubmed/15615544?dopt=AbstractPlus

[535] Rotella DP *et al* (2009) http://www.ncbi.nlm.nih.gov/pubmed/19720528?dopt=AbstractPlus

[536] Rothstein JD *et al* (2005) http://www.ncbi.nlm.nih.gov/pubmed/15635412?dopt=AbstractPlus

[537] Rousseau F *et al* (2008) http://www.ncbi.nlm.nih.gov/pubmed/18815261?dopt=AbstractPlus

[538] Ryan RM *et al* (2007) http://www.ncbi.nlm.nih.gov/pubmed/17435767?dopt=AbstractPlus

[539] Ryan RM *et al* (2004) http://www.ncbi.nlm.nih.gov/pubmed/14982939?dopt=AbstractPlus

[540] Rühl A *et al* (2005) http://www.ncbi.nlm.nih.gov/pubmed/16041713?dopt=AbstractPlus

[541] Sager G *et al* (2012) http://www.ncbi.nlm.nih.gov/pubmed/22380603?dopt=AbstractPlus

[542] Sagné C *et al* (2001) http://www.ncbi.nlm.nih.gov/pubmed/11390972?dopt=AbstractPlus

[543] Sahni J *et al* (2013) http://www.ncbi.nlm.nih.gov/pubmed/23506895?dopt=AbstractPlus

[544] Said HM. (2009) http://www.ncbi.nlm.nih.gov/pubmed/19056639?dopt=AbstractPlus

[545] Said HM *et al* (1989) http://www.ncbi.nlm.nih.gov/pubmed/2911998?dopt=AbstractPlus

[546] Saier MH *et al* (2009) http://www.ncbi.nlm.nih.gov/pubmed/19022853?dopt=AbstractPlus

[547] Saito K *et al* (2010) http://www.ncbi.nlm.nih.gov/pubmed/21190592?dopt=AbstractPlus

[548] Sakata K *et al* (2001) http://www.ncbi.nlm.nih.gov/pubmed/11336635?dopt=AbstractPlus

[549] Sala-Rabanal M *et al* (2006) http://www.ncbi.nlm.nih.gov/pubmed/16627568?dopt=AbstractPlus

[550] Sala-Rabanal M *et al* (2008) http://www.ncbi.nlm.nih.gov/pubmed/18367661?dopt=AbstractPlus

[551] Salazar G *et al* (2009) http://www.ncbi.nlm.nih.gov/pubmed/19521526?dopt=AbstractPlus

[552] Salojin KV *et al* (2011) http://www.ncbi.nlm.nih.gov/pubmed/21346251?dopt=AbstractPlus

[553] Sandoval A *et al* (2010) http://www.ncbi.nlm.nih.gov/pubmed/19913517?dopt=AbstractPlus

[554] Sasawatari S *et al* (2011) http://www.ncbi.nlm.nih.gov/pubmed/21277849?dopt=AbstractPlus

[555] Sawada K *et al* (2008) http://www.ncbi.nlm.nih.gov/pubmed/18375752?dopt=AbstractPlus

[556] Sawada K *et al* (1999) http://www.ncbi.nlm.nih.gov/pubmed/10578127?dopt=AbstractPlus

[557] Schaffer JE *et al* (1994) http://www.ncbi.nlm.nih.gov/pubmed/7954810?dopt=AbstractPlus

[558] Schirmer SU *et al* (2011) http://www.ncbi.nlm.nih.gov/pubmed/21482687?dopt=AbstractPlus

[559] Schiöth HB *et al* (2013) http://www.ncbi.nlm.nih.gov/pubmed/23506890?dopt=AbstractPlus

[560] Schlipf NA *et al* (2010) http://www.ncbi.nlm.nih.gov/pubmed/20461110?dopt=AbstractPlus

[561] Schousboe A *et al* (2011) http://www.ncbi.nlm.nih.gov/pubmed/21428813?dopt=AbstractPlus

[562] Schousboe A *et al* (2004) http://www.ncbi.nlm.nih.gov/pubmed/15451399?dopt=AbstractPlus

[563] Secondo A *et al* (2015) http://www.ncbi.nlm.nih.gov/pubmed/25942323?dopt=AbstractPlus

[564] Seidler NW *et al* (1989) http://www.ncbi.nlm.nih.gov/pubmed/2530215?dopt=AbstractPlus

[565] Sekler I. (2015) http://www.ncbi.nlm.nih.gov/pubmed/25998733?dopt=AbstractPlus

[566] Semyanov A *et al* (2004) http://www.ncbi.nlm.nih.gov/pubmed/15111008?dopt=AbstractPlus

[567] Sethi AA *et al* (2008) http://www.ncbi.nlm.nih.gov/pubmed/18805791?dopt=AbstractPlus

[568] Seyffer F *et al* (2015) http://www.ncbi.nlm.nih.gov/pubmed/24923865?dopt=AbstractPlus

[569] Shao J *et al* (2016) http://www.ncbi.nlm.nih.gov/pubmed/27669901?dopt=AbstractPlus

[570] Shigeri Y *et al* (2001) http://www.ncbi.nlm.nih.gov/pubmed/11677257?dopt=AbstractPlus

[571] Shimamoto K *et al* (1998) http://www.ncbi.nlm.nih.gov/pubmed/9463476?dopt=AbstractPlus

[572] Shimamoto K *et al* (2007) http://www.ncbi.nlm.nih.gov/pubmed/17047096?dopt=AbstractPlus

[573] Shimamoto K *et al* (2000) http://www.ncbi.nlm.nih.gov/pubmed/11078189?dopt=AbstractPlus

[574] Shimokawa N *et al* (2002) http://www.ncbi.nlm.nih.gov/pubmed/12417639?dopt=AbstractPlus

[575] Shintre CA *et al* (2013) http://www.ncbi.nlm.nih.gov/pubmed/23716676?dopt=AbstractPlus

[576] Shu Y *et al* (2007) http://www.ncbi.nlm.nih.gov/pubmed/17476361?dopt=AbstractPlus

[577] Sievert MK *et al* (1997) http://www.ncbi.nlm.nih.gov/pubmed/9325342?dopt=AbstractPlus

[578] Singer D *et al* (2009) http://www.ncbi.nlm.nih.gov/pubmed/19478081?dopt=AbstractPlus

[579] Singh N *et al* (2010) http://www.ncbi.nlm.nih.gov/pubmed/20601425?dopt=AbstractPlus

[580] Singh SK *et al* (2007) http://www.ncbi.nlm.nih.gov/pubmed/17687333?dopt=AbstractPlus

[581] Sloan JL *et al* (1999) http://www.ncbi.nlm.nih.gov/pubmed/10446133?dopt=AbstractPlus

[582] Snyder NA *et al* (2019) http://www.ncbi.nlm.nih.gov/pubmed/30622138?dopt=AbstractPlus

[583] Song F *et al* (2017) http://www.ncbi.nlm.nih.gov/pubmed/27836942?dopt=AbstractPlus

[584] Song F *et al* (2018) http://www.ncbi.nlm.nih.gov/pubmed/29224352?dopt=AbstractPlus

[585] Sreedharan S *et al* (2011) http://www.ncbi.nlm.nih.gov/pubmed/21044875?dopt=AbstractPlus

[586] Stahl A *et al* (1999) http://www.ncbi.nlm.nih.gov/pubmed/10518211?dopt=AbstractPlus

[587] Stansberry WM *et al* (2018) http://www.ncbi.nlm.nih.gov/pubmed/30351207?dopt=AbstractPlus 10.1089/gtmb.2018.0194 PMC6249667 30351207

[588] Stecula A *et al* (2017) http://www.ncbi.nlm.nih.gov/pubmed/28661652?dopt=AbstractPlus

[589] Stewart G. (2011) http://www.ncbi.nlm.nih.gov/pubmed/21449978?dopt=AbstractPlus

[590] Stieger B. (2009) http://www.ncbi.nlm.nih.gov/pubmed/19684528?dopt=AbstractPlus

[591] Sultan M *et al* (2018) http://www.ncbi.nlm.nih.gov/pubmed/28898457?dopt=AbstractPlus

[592] Sun D *et al* (2013) http://www.ncbi.nlm.nih.gov/pubmed/23442152?dopt=AbstractPlus

[593] Sundaram M *et al* (1998) http://www.ncbi.nlm.nih.gov/pubmed/9705281?dopt=AbstractPlus

[594] Supplisson S *et al* (2002) http://www.ncbi.nlm.nih.gov/pubmed/12354619?dopt=AbstractPlus

[595] Suzuki H *et al* (1998) http://www.ncbi.nlm.nih.gov/pubmed/9875554?dopt=AbstractPlus

[596] Suzuki T *et al* (2005) http://www.ncbi.nlm.nih.gov/pubmed/15994300?dopt=AbstractPlus

[597] Swaan PW *et al* (2008) http://www.ncbi.nlm.nih.gov/pubmed/18474668?dopt=AbstractPlus

[598] Tagoh H *et al* (1996) http://www.ncbi.nlm.nih.gov/pubmed/8630032?dopt=AbstractPlus

[599] Tai W *et al* (2013) http://www.ncbi.nlm.nih.gov/pubmed/22950754?dopt=AbstractPlus

[600] Tailor CS *et al* (1999) http://www.ncbi.nlm.nih.gov/pubmed/9927670?dopt=AbstractPlus

[601] Tailor CS *et al* (1999) http://www.ncbi.nlm.nih.gov/pubmed/10400745?dopt=AbstractPlus

[602] Takeuchi T *et al* (2017) http://www.ncbi.nlm.nih.gov/pubmed/28082679?dopt=AbstractPlus

[603] Talvenheimo J *et al* (1983) http://www.ncbi.nlm.nih.gov/pubmed/6853478?dopt=AbstractPlus

[604] Tamai I *et al* (1997) http://www.ncbi.nlm.nih.gov/pubmed/9379359?dopt=AbstractPlus

[605] Tamarappoo BK *et al* (1996) http://www.ncbi.nlm.nih.gov/pubmed/8603078?dopt=AbstractPlus

[606] Tang L *et al* (2012) http://www.ncbi.nlm.nih.gov/pubmed/22085049?dopt=AbstractPlus

[607] Tanihara Y *et al* (2007) http://www.ncbi.nlm.nih.gov/pubmed/17509534?dopt=AbstractPlus

[608] Tatsumi M *et al* (1997) http://www.ncbi.nlm.nih.gov/pubmed/9537821?dopt=AbstractPlus

[609] Taylor NMI *et al* (2017) http://www.ncbi.nlm.nih.gov/pubmed/28554189?dopt=AbstractPlus

[610] Tejada-Jiménez M *et al* (2011) http://www.ncbi.nlm.nih.gov/pubmed/21464289?dopt=AbstractPlus

[611] Terada T *et al* (2006) http://www.ncbi.nlm.nih.gov/pubmed/16850272?dopt=AbstractPlus

[612] Terada T *et al* (1997) http://www.ncbi.nlm.nih.gov/pubmed/9374833?dopt=AbstractPlus

[613] Terada T *et al* (1996) http://www.ncbi.nlm.nih.gov/pubmed/8843163?dopt=AbstractPlus

[614] Terada T *et al* (2000) http://www.ncbi.nlm.nih.gov/pubmed/10748266?dopt=AbstractPlus

[615] Thangaraju M *et al* (2006) http://www.ncbi.nlm.nih.gov/pubmed/16873376?dopt=AbstractPlus

[616] Thangaraju M *et al* (2006) http://www.ncbi.nlm.nih.gov/pubmed/17178845?dopt=AbstractPlus

[617] Theis S *et al* (2002) http://www.ncbi.nlm.nih.gov/pubmed/11752223?dopt=AbstractPlus

[618] Thomsen C *et al* (1997) http://www.ncbi.nlm.nih.gov/pubmed/9134205?dopt=AbstractPlus

[619] Thwaites DT *et al* (2011) http://www.ncbi.nlm.nih.gov/pubmed/21501141?dopt=AbstractPlus

[620] Tollefson MB *et al* (2003) http://www.ncbi.nlm.nih.gov/pubmed/14552767?dopt=AbstractPlus

[621] Torres-Salazar D *et al* (2007) http://www.ncbi.nlm.nih.gov/pubmed/17908688?dopt=AbstractPlus

[622] Traiffort E *et al* (2005) http://www.ncbi.nlm.nih.gov/pubmed/15715662?dopt=AbstractPlus

[623] Treiber A *et al* (2007) http://www.ncbi.nlm.nih.gov/pubmed/17496208?dopt=AbstractPlus

[624] Tsai G *et al* (2004) http://www.ncbi.nlm.nih.gov/pubmed/15159536?dopt=AbstractPlus

[625] Tse CM *et al* (1993) http://www.ncbi.nlm.nih.gov/pubmed/8415663?dopt=AbstractPlus

[626] Tse CM *et al* (1993) http://www.ncbi.nlm.nih.gov/pubmed/7685025?dopt=AbstractPlus

[627] Tsuda M *et al* (2009) http://www.ncbi.nlm.nih.gov/pubmed/19164462?dopt=AbstractPlus

[628] Tsukaguchi H *et al* (1999) http://www.ncbi.nlm.nih.gov/pubmed/10331392?dopt=AbstractPlus

[629] Tóth A *et al* (2002) http://www.ncbi.nlm.nih.gov/pubmed/12054538?dopt=AbstractPlus

[630] Uchida Y *et al* (2009) http://www.ncbi.nlm.nih.gov/pubmed/19122366?dopt=AbstractPlus

[631] Uldry M *et al* (2002) http://www.ncbi.nlm.nih.gov/pubmed/12135767?dopt=AbstractPlus

[632] Umapathy NS *et al* (2004) http://www.ncbi.nlm.nih.gov/pubmed/15290873?dopt=AbstractPlus

[633] Ussar S *et al* (2014) http://www.ncbi.nlm.nih.gov/pubmed/25080478?dopt=AbstractPlus

[634] Utsunomiya-Tate N *et al* (1996) http://www.ncbi.nlm.nih.gov/pubmed/8662767?dopt=AbstractPlus

[635] van de Wiel SMW *et al* (2018) http://www.ncbi.nlm.nih.gov/pubmed/29675448?dopt=AbstractPlus

[636] van Leeuwen EM *et al* (2015) http://www.ncbi.nlm.nih.gov/pubmed/25751400?dopt=AbstractPlus

[637] van Roermund CW *et al* (2008) http://www.ncbi.nlm.nih.gov/pubmed/18757502?dopt=AbstractPlus

[638] van Roermund CW *et al.* (2011) http://www.ncbi.nlm.nih.gov/pubmed/21145416?dopt=AbstractPlus

[639] Vandenberg RJ *et al* (2004) http://www.ncbi.nlm.nih.gov/pubmed/15324920?dopt=AbstractPlus

[640] Vandenberg RJ *et al* (1997) http://www.ncbi.nlm.nih.gov/pubmed/9145919?dopt=AbstractPlus

[641] Vandenberg RJ *et al* (2007) http://www.ncbi.nlm.nih.gov/pubmed/17383967?dopt=AbstractPlus

[642] Vanderperre B *et al* (2016) http://www.ncbi.nlm.nih.gov/pubmed/27317664?dopt=AbstractPlus

[643] Vanslambrouck JM *et al* (2010) http://www.ncbi.nlm.nih.gov/pubmed/20377526?dopt=AbstractPlus

[644] Varoqui H *et al* (1996) http://www.ncbi.nlm.nih.gov/pubmed/8910293?dopt=AbstractPlus

[645] Vavricka SR *et al* (2004) http://www.ncbi.nlm.nih.gov/pubmed/15521010?dopt=AbstractPlus

[646] Vavricka SR *et al* (2002) http://www.ncbi.nlm.nih.gov/pubmed/12085361?dopt=AbstractPlus

[647] Verheijen FW *et al* (1999) http://www.ncbi.nlm.nih.gov/pubmed/10581036?dopt=AbstractPlus

[648] Veruki ML *et al* (2006) http://www.ncbi.nlm.nih.gov/pubmed/17041592?dopt=AbstractPlus

[649] Visser WE *et al* (2010) http://www.ncbi.nlm.nih.gov/pubmed/19682536?dopt=AbstractPlus

[650] Voss AA *et al* (2007) http://www.ncbi.nlm.nih.gov/pubmed/17110502?dopt=AbstractPlus

[651] Wallgard E *et al* (2008) http://www.ncbi.nlm.nih.gov/pubmed/18483404?dopt=AbstractPlus

[652] Wang C *et al* (2013) http://www.ncbi.nlm.nih.gov/pubmed/24021350?dopt=AbstractPlus

[653] Wang D *et al* (2003) http://www.ncbi.nlm.nih.gov/pubmed/14634667?dopt=AbstractPlus

[654] Wang H *et al* (1999) http://www.ncbi.nlm.nih.gov/pubmed/10329687?dopt=AbstractPlus

[655] Wang J. (2016) http://www.ncbi.nlm.nih.gov/pubmed/27506881?dopt=AbstractPlus

[656] Wang JZ *et al* (2016) http://www.ncbi.nlm.nih.gov/pubmed/26747400?dopt=AbstractPlus

[657] Wang Q *et al* (2006) http://www.ncbi.nlm.nih.gov/pubmed/16707723?dopt=AbstractPlus

[658] Wang XX *et al* (2017) http://www.ncbi.nlm.nih.gov/pubmed/27845049?dopt=AbstractPlus

[659] Wang XX *et al* (2018) http://www.ncbi.nlm.nih.gov/pubmed/29305823?dopt=AbstractPlus

[660] Wang Y *et al* (2018) http://www.ncbi.nlm.nih.gov/pubmed/29305856?dopt=AbstractPlus

[661] Warraich S *et al* (2013) http://www.ncbi.nlm.nih.gov/pubmed/23184610?dopt=AbstractPlus

[662] Weinman SA *et al* (1998) http://www.ncbi.nlm.nih.gov/pubmed/9856990?dopt=AbstractPlus

[663] Wenzel U *et al* (1998) http://www.ncbi.nlm.nih.gov/pubmed/9843719?dopt=AbstractPlus

[664] Wenzel U *et al* (1996) http://www.ncbi.nlm.nih.gov/pubmed/8627565?dopt=AbstractPlus

[665] Westhoff CM *et al* (2002) http://www.ncbi.nlm.nih.gov/pubmed/11861637?dopt=AbstractPlus

[666] White C *et al* (2013) http://www.ncbi.nlm.nih.gov/pubmed/23395172?dopt=AbstractPlus

[667] White HS *et al* (2005) http://www.ncbi.nlm.nih.gov/pubmed/15550575?dopt=AbstractPlus

[668] Wiles AL *et al* (2006) http://www.ncbi.nlm.nih.gov/pubmed/16899062?dopt=AbstractPlus

[669] Wille S *et al* (2001) http://www.ncbi.nlm.nih.gov/pubmed/11698453?dopt=AbstractPlus

[670] Wilson BJ *et al* (2014) http://www.ncbi.nlm.nih.gov/pubmed/24934811?dopt=AbstractPlus

[671] Wojcik SM *et al* (2006) http://www.ncbi.nlm.nih.gov/pubmed/16701208?dopt=AbstractPlus

[672] Wolf S *et al* (2002) http://www.ncbi.nlm.nih.gov/pubmed/12049641?dopt=AbstractPlus

[673] Wong EH *et al* (2000) http://www.ncbi.nlm.nih.gov/pubmed/10812041?dopt=AbstractPlus

[674] Wright EM *et al* (2011) http://www.ncbi.nlm.nih.gov/pubmed/21527736?dopt=AbstractPlus

[675] Wright EM *et al* (2004) http://www.ncbi.nlm.nih.gov/pubmed/12748858?dopt=AbstractPlus

[676] Wu CA *et al* (2004) http://www.ncbi.nlm.nih.gov/pubmed/15140889?dopt=AbstractPlus

[677] Wu SP *et al* (2013) http://www.ncbi.nlm.nih.gov/pubmed/23259992?dopt=AbstractPlus

[678] Wu X *et al* (2002) http://www.ncbi.nlm.nih.gov/pubmed/12504846?dopt=AbstractPlus

[679] Wu Y *et al* (2013) http://www.ncbi.nlm.nih.gov/pubmed/23678871?dopt=AbstractPlus

[680] Wängler B *et al* (2004) http://www.ncbi.nlm.nih.gov/pubmed/15380228?dopt=AbstractPlus

[681] Xiang J *et al* (2006) http://www.ncbi.nlm.nih.gov/pubmed/17034769?dopt=AbstractPlus

[682] Xu X *et al* (2018) http://www.ncbi.nlm.nih.gov/pubmed/29615471?dopt=AbstractPlus

[683] Yabuki M *et al* (2009) http://www.ncbi.nlm.nih.gov/pubmed/19236841?dopt=AbstractPlus

[684] Yamada T *et al* (2011) http://www.ncbi.nlm.nih.gov/pubmed/21185344?dopt=AbstractPlus

[685] Yamamoto S *et al* (2010) http://www.ncbi.nlm.nih.gov/pubmed/20042597?dopt=AbstractPlus

[686] Yamamoto T *et al* (1999) http://www.ncbi.nlm.nih.gov/pubmed/10480349?dopt=AbstractPlus

[687] Yamashita A *et al* (2005) http://www.ncbi.nlm.nih.gov/pubmed/16041361?dopt=AbstractPlus

[688] Yamashita K *et al* (2016) http://www.ncbi.nlm.nih.gov/pubmed/27480939?dopt=AbstractPlus

[689] Yamashita T *et al* (1997) http://www.ncbi.nlm.nih.gov/pubmed/9092568?dopt=AbstractPlus

[690] Yanagisawa H *et al* (2003) http://www.ncbi.nlm.nih.gov/pubmed/12815463?dopt=AbstractPlus

[691] Yao SY *et al* (2011) http://www.ncbi.nlm.nih.gov/pubmed/21795683?dopt=AbstractPlus

[692] Yao Y *et al* (2010) http://www.ncbi.nlm.nih.gov/pubmed/20463145?dopt=AbstractPlus

[693] Yasujima T *et al* (2010) http://www.ncbi.nlm.nih.gov/pubmed/20047987?dopt=AbstractPlus

[694] Ye X *et al* (2003) http://www.ncbi.nlm.nih.gov/pubmed/12670026?dopt=AbstractPlus

[695] Yee BK *et al* (2006) http://www.ncbi.nlm.nih.gov/pubmed/16554468?dopt=AbstractPlus

[696] Yernool D *et al* (2004) http://www.ncbi.nlm.nih.gov/pubmed/15483603?dopt=AbstractPlus

[697] Youngblood GL *et al* (2004) http://www.ncbi.nlm.nih.gov/pubmed/15068970?dopt=AbstractPlus

[698] Yu XC *et al* (2009) http://www.ncbi.nlm.nih.gov/pubmed/19159658?dopt=AbstractPlus

[699] Yu Z *et al* (2007) http://www.ncbi.nlm.nih.gov/pubmed/17325024?dopt=AbstractPlus

[700] Zaia KA *et al* (2009) http://www.ncbi.nlm.nih.gov/pubmed/19147495?dopt=AbstractPlus

[701] Zander JF *et al* (2010) http://www.ncbi.nlm.nih.gov/pubmed/20519538?dopt=AbstractPlus

[702] Zelcer N *et al* (2003) http://www.ncbi.nlm.nih.gov/pubmed/12523936?dopt=AbstractPlus

[703] Zeng Z *et al* (2008) http://www.ncbi.nlm.nih.gov/pubmed/18355687?dopt=AbstractPlus

[704] Zerangue N *et al* (1996) http://www.ncbi.nlm.nih.gov/pubmed/8910405?dopt=AbstractPlus

[705] Zerangue N *et al* (1996) http://www.ncbi.nlm.nih.gov/pubmed/8857541?dopt=AbstractPlus

[706] Zerangue N *et al* (1996) http://www.ncbi.nlm.nih.gov/pubmed/8782106?dopt=AbstractPlus

[707] Zhang HX *et al* (2009) http://www.ncbi.nlm.nih.gov/pubmed/19433577?dopt=AbstractPlus

[708] Zhang L *et al* (1998) http://www.ncbi.nlm.nih.gov/pubmed/9655880?dopt=AbstractPlus

[709] Zhang X *et al* (2009) http://www.ncbi.nlm.nih.gov/pubmed/19515813?dopt=AbstractPlus

[710] Zhang Z *et al* (2012) http://www.ncbi.nlm.nih.gov/pubmed/22749870?dopt=AbstractPlus

[711] Zhao D *et al* (2007) http://www.ncbi.nlm.nih.gov/pubmed/17506977?dopt=AbstractPlus

[712] Zhao R *et al* (2002) http://www.ncbi.nlm.nih.gov/pubmed/11997266?dopt=AbstractPlus

[713] Zheng H *et al* (2003) http://www.ncbi.nlm.nih.gov/pubmed/14756423?dopt=AbstractPlus

[714] Zhou H *et al* (2009) http://www.ncbi.nlm.nih.gov/pubmed/19717468?dopt=AbstractPlus

[715] Zhou LM *et al* (1997) http://www.ncbi.nlm.nih.gov/pubmed/8996224?dopt=AbstractPlus

[716] Zhou M *et al* (2010) http://www.ncbi.nlm.nih.gov/pubmed/20592246?dopt=AbstractPlus

[717] Zhou M *et al* (2007) http://www.ncbi.nlm.nih.gov/pubmed/17600084?dopt=AbstractPlus

[718] Zhou W *et al* (2010) http://www.ncbi.nlm.nih.gov/pubmed/20448275?dopt=AbstractPlus

[719] Zhu HJ *et al* (2010) http://www.ncbi.nlm.nih.gov/pubmed/20402963?dopt=AbstractPlus

[720] Zhu L *et al* (2009) http://www.ncbi.nlm.nih.gov/pubmed/19632829?dopt=AbstractPlus

[721] Zidi-Yahiaoui N *et al* (2009) http://www.ncbi.nlm.nih.gov/pubmed/19553567?dopt=AbstractPlus

[722] Zimmermann M *et al* (2010) http://www.ncbi.nlm.nih.gov/pubmed/20868728?dopt=AbstractPlus

[723] Zimmermann M *et al* (2010) http://www.ncbi.nlm.nih.gov/pubmed/19612975?dopt=AbstractPlus

[724] Zipp GG *et al* (2014) http://www.ncbi.nlm.nih.gov/pubmed/25037917?dopt=AbstractPlus

[725] Zotova L *et al* (2010) http://www.ncbi.nlm.nih.gov/pubmed/20197279?dopt=AbstractPlus

[726] Zou S *et al* (2011) http://www.ncbi.nlm.nih.gov/pubmed/21426345?dopt=AbstractPlus

